# Research on the Mechanism of Liuwei Dihuang Decoction for Osteoporosis Based on Systematic Biological Strategies

**DOI:** 10.1155/2022/7017610

**Published:** 2022-09-22

**Authors:** Zhi-yong Long, Jia-min Wu, Wang Xiang, Meng-xia Yuan, Yong-he Wu, Jun Li, Gan-peng Yu, Tiejun Yang

**Affiliations:** ^1^Department of Rehabilitation Medicine, Guangzhou Panyu Central Hospital, Guangzhou, China; ^2^Hunan University of Chinese Medicine, Changsha, Hunan, China; ^3^People's Hospital of Ningxiang City, Ningxiang 410600, Hunan, China

## Abstract

**Background:**

Osteoporosis is an important health problem worldwide. Liuwei Dihuang Decoction (LDD) and its main ingredients may have a good clinical effect on osteoporosis. Meanwhile, its mechanism for treating osteoporosis needs to be further revealed in order to provide a basis for future drug development.

**Methods:**

A systematic biological methodology was utilized to construct and analyze the LDD-osteoporosis network. After that, the human transcription data of LDD intervention in patients with osteoporosis and protein arrays data of LDD intervention in osteoporosis rats were collected. The human transcription data analysis, protein arrays data analysis, and molecular docking were performed to validate the findings of the prediction network (LDD-osteoporosis PPI network). Finally, animal experiments were conducted to verify the prediction results of systematic pharmacology.

**Results:**

(1) LDD-osteoporosis PPI network shows the potential compounds, potential targets (such as ALB, IGF1, SRC, and ESR1), clusters, biological processes (such as positive regulation of calmodulin 1-monooxygenase activity, estrogen metabolism, and endothelial cell proliferation), and signaling and Reactome pathways (such as JAK-STAT signaling pathway, osteoclast differentiation, and degradation of the extracellular matrix) of LDD intervention in osteoporosis. (2) Human transcriptomics data and protein arrays data validated the findings of the LDD-osteoporosis PPI network. (3) The animal experiments showed that LDD can improve bone mineral density (BMD), increase serum estradiol (E2) and alkaline phosphatase (ALP) levels, and upregulate Wnt3a and *β*-catenin mRNA expression (*P* < 0.05). (4) Molecular docking results showed that alisol A, dioscin, loganin, oleanolic acid, pachymic acid, and ursolic acid may stably bind to JAK2, ESR1, and CTNNB1.

**Conclusion:**

LDD may have a therapeutic effect on osteoporosis through regulating the targets (such as ALB, IGF1, SRC, and ESR1), biological processes (such as positive regulation of calmodulin 1-monooxygenase activity, estrogen metabolism, and endothelial cell proliferation), and pathways (such as JAK-STAT signaling pathway, osteoclast differentiation, and degradation of the extracellular matrix) found in this research.

## 1. Introduction

Osteoporosis is a common systemic metabolic bone disease with a decrease in bone density and bone quality and bone microstructural damage caused by various reasons [1]. The serious clinical outcome of osteoporosis is osteoporotic fractures (fragility fractures), which lead to a significant increase in morbidity and mortality in patients with osteoporosis [2]. The treatments for osteoporosis include the following: (1) basic prevention, such as lifestyle adjustment (diet and outdoor sports) and basic bone health supplements (calcium and vitamin D); (2) drug interventions, such as antibone resorption drugs (bisphosphonates, calcitonin, selective estrogen receptor modulators (SERMs), and estrogen); (3) drugs that promote bone formation, such as targeted drugs [2–4]. However, recent studies showed that the preventive and therapeutic effects of the above drugs are still controversial, such as vitamin D [5], while antibone resorption drugs increase the burden of medical resources and reduce patient compliance due to their high price [6]. Natural plant products have become the direction of new drug development due to their multicompound, multitarget features and cheap price [7, 8].

Traditional Chinese medicine (TCM), as a traditional medicine applied for thousands of years, has gradually highlighted its therapeutic advantages in osteoporosis through long medical clinical practice [8]. Liuwei Dihuang Decoction (LDD) as a representative of TCM for the treatment of osteoporosis, comes from Jingyue Quanshu. This formula is composed of *Rehmannia glutinosa (*Gaertn.) DC (Rehmanniae Radix Praeparata, Dihuang), *Cornus Officinalis* Sieb. et Zucc. (Shanzhuyu), *Paeonia × suffruticosa* Andrews (Cortex Moutan, Mudanpi), *Dioscorea oppositifolia* L. (*Rhizoma Dioscoreae*, Shanyao), *Poria cocos* (Schw.) Wolf. (Fuling), and *Alisma plantago-aquatica* L. (*Alisma orientale* (Sam.) Juz., Zexie). Current clinical studies showed that LDD alone or in combination with other antiosteoporosis drugs (alendronate, salmon calcitonin) for osteoporosis has a certain effect: it can effectively improve the patient's bone density (in the lumbar spine, the femoral neck, the forearm, the distal third of the junction, and the tibia bone density) and the clinical efficacy rate [9, 10]. A systematic review and meta-analysis also showed that LDD can increase the bone mineral density of the hip, lumbar spine, ulna, and radius, and it has a good effect in improving the effective rate of clinical treatment of postmenopausal osteoporosis and reducing the pain caused by osteoporosis [11]. Its mechanism may be related to the regulation of hormone levels and oxidative stress through the Wnt/*β*-catenin signaling pathway [12, 13]. In addition, current pharmacological studies showed that LDD has the effect of inhibiting aging and prolonging the lifespan of *C. elegans* life-sustaining and natural aging mice [14]. However, its mechanism for treating osteoporosis needs to be further revealed in order to provide a basis for future drug development.

**Figure 1:**
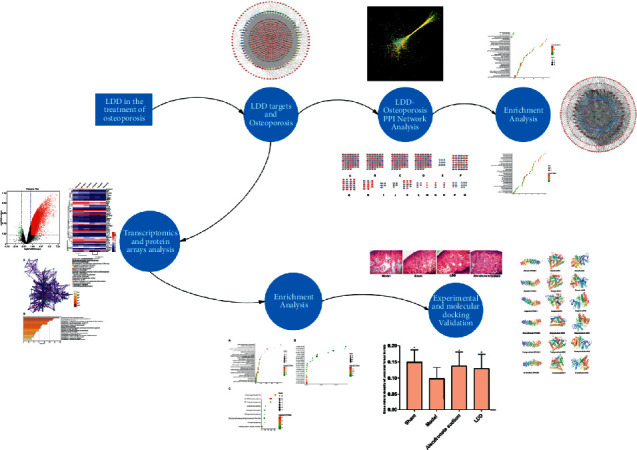
The processes of this study.

Systemic pharmacology is a discipline that studies the effects of drugs on disease at a system level, which combines multidisciplinary technologies such as bioinformatics and network pharmacology to bring new strategies for analyzing the mechanism of drugs [15, 16]. This method can resolve multicompound and multitarget drugs from microscopic (molecular and biochemical network level) to macroscopic (tissue, organ, and overall level) levels [15, 16]. Meanwhile, at present, researchers have explored the therapeutic mechanisms of natural products for disease through systematic pharmacology [17–20]. Therefore, in this research, the systematic pharmacological methodology would be utilized to uncover the mechanism of LDD on osteoporosis. The process of this study is shown in Figure 1.

## 2. Materials and Methods

### 2.1. LDD's Compounds Prediction

The TCM Database@Taiwan [21] (https://tcm.cmu.edu.tw/zh-tw/) and the TCMSP database [22] (https://tcmspw.com/tcmsp.php) were utilized to collect the compounds of LDD. Then, in order to predict the bioactive compounds of LDD, oral bioavailability (OB), Caco-2 permeability, and drug-likeness (DL) were applied [17, 18]. The standard was OB ≥30%, Caco-2 >−0.4, and DL ≥0.18 [21]. The components meeting this standard was included. Meanwhile, due to the limitations of prediction methods based on pharmacokinetic parameters [23], in order to avoid losing potential active components, we searched a large amount of literature [24–28] to collect compounds that are orally absorbable and biologically active. The pharmacokinetic parameters of the components are shown in Table 1.

### 2.2. LDD's Potential Target Prediction and Osteoporosis Genes Collection

The molecular structures of each compound were collected from the PubChem (https://pubchem.ncbi.nlm.nih.gov/) and the SciFinder (https://scifinder.cas.org) and saved as “sdf” file format. These “sdf” files of each compound were input into PharmMapper (https://lilab-ecust.cn/pharmmapper/) to predict the potential targets [29]. The name of the target protein was imported into UniProtKB (https://www.uniprot.org/), and the species was limited to “*Homo sapiens*,” so that the name of the protein was corrected to their official symbols (see Table S1 in Supplementary Materials). Meanwhile, the OMIM database (https://omim.org/) and GeneCards (https://www.genecards.org) were utilized to collect the osteoporosis-related disease genes and targets with the keyword “Osteoporosis” [17, 18]. The osteoporosis-related genes and their relevance scores are shown in Table S2.

### 2.3. Human Transcriptome Data and Protein Arrays Data Collection

Transcriptome data come from GEO (https://www.ncbi.nlm.nih.gov/geo/). The data on LDD treating osteoporosis were obtained from GSE57273. The protein arrays data of LDD intervention in osteoporosis rats come from [30]. Differential expression analysis was performed using the R software package.

### 2.4. Network Construction and Analysis Methods

The protein-protein interaction (PPI) data were collected from String database (https://string-db.org/) with medium confidence >0.4 and the IntAct database (https://www.ebi.ac.uk/intact/) [31, 32]. The LDD targets, osteoporosis genes, and their PPI data were input into Cytoscape 3.7.0 (https://cytoscape.org/) for network construction and analysis [33]. In the PPI network, there are dense areas of some molecular complexes, which are defined as clusters [33]. Clusters can be viewed as functional modules, and drugs may treat diseases by regulating these functional modules. The cluster was obtained by analyzing the network using MCODE, a plug-in of Cytoscape software.

### 2.5. Gene Ontology (GO), Signaling Pathway, and Reactome Pathway Enrichment Analysis

The LDD targets, osteoporosis genes, human transcriptome data, and protein arrays data were input into the DAVID database ver. 6.8 (https://david-d.ncifcrf.gov) for GO enrichment analysis and Kyoto Encyclopedia of Genes and Genomes (KEGG) signaling pathway enrichment analysis [34]. GO enrichment analysis includes biological processes, cell components, and molecular function. Meanwhile, those data were input into the Reactome Pathway Database (https://reactome.org/) for Reactome pathway enrichment analysis [35].

### 2.6. Experimental Materials

#### 2.6.1. Instruments and Reagents

LDD (Approval number: National Pharmaceutical Standard Z11020056) was purchased from Beijing Tongrentang Technology Development Co., Ltd. Alendronate sodium (Approval no. National Pharmaceutical Standard J20130085) was purchased from Hangzhou Merck Pharmaceutical Co., Ltd. Estradiol (E2), alkaline phosphatase (ALP), enzyme-linked immunosorbent assay (ELISA) kit were purchased from Beijing Northern Biotechnology Research Institute Co., Ltd. (lot number: 180820, 180416). Dual-energy X-ray absorptiometry was purchased from General Electric Company. Wnt3a, *β*-catenin, and *β*-actin primers were synthesized by Shanghai Shengong Biotechnology Co., Ltd.

#### 2.6.2. Experimental Animal

In total, 40 healthy SD female rats, 180–240 days old, were provided by Hunan Slake Jingda Experimental Animal Co., Ltd. [Animal License Number: SYXK(Xiang)2018-0002] and were raised in the Experimental Animal Center of Hunan University of Chinese Medicine. The animals were weighed, numbered, and divided into 4 groups according to the random sequence method: sham operation group (10 rats), model group (10 rats), LDD group (10 rats), and positive control group (10 rats). Animal experiments have been approved by the Animal Ethics Committee of Hunan University of Chinese Medicine (Approval no. HUTCM-LL20199306) and performed in accordance with the guidelines for the care and use of experimental animals.

### 2.7. Experimental Methods

#### 2.7.1. Animal Modeling and Intervention

After 1 week of adaptive feeding, rats underwent bilateral ovarian resection under anesthesia (3% pentobarbital sodium 30 mg/kg intraperitoneal injection) to create postmenopausal osteoporosis (PMOP) model. On the 7th day after the operation, the rats began to administer the medicine by gavage daily. The sham operation group and the model group were intragastrically administered with 10 mL/kg of normal saline. The rats in the LDD group were gavaged with LDD 6.75 g crude drug/kg. The rats in the positive control group were gavaged with alendronate sodium 0.1 mg/mL.

#### 2.7.2. Specimen Collection and Processing

Under anesthesia with 3% pentobarbital sodium 30 mg/kg, blood was collected from the abdominal aorta. After the blood was placed at room temperature for 1 hour, the blood was centrifuged at 3 500 r/min for 15 minutes. The serum was separated and stored in a refrigerator at −80°C. The right femur of the rat was stored at −20°C for bone density detection and Quantitative Real-time PCR (qRT-PCR). The left femoral head of the rat was fixed with 4% paraformaldehyde for HE staining.

#### 2.7.3. Serum E2, ALP Level, and Bone Mineral Density (BMD) Detection

Serum E2 and ALP were detected by ELISA. BMD was measured by a dual-energy X-ray bone densitometer.

#### 2.7.4. Histopathological Observation

The left femoral head was fixed with a volume fraction of 4% paraformaldehyde for 72 h and then decalcified with 10% EDTA decalcification solution, and the decalcification solution was changed every 3 days. After decalcification was completed, it was rinsed with PBS, dehydrated with gradient ethanol, cleared with xylene, embedded in paraffin, and sliced for HE staining. The histopathological changes in the femoral head were observed under a light microscope.

#### 2.7.5. Genetic Testing Methods

Blood RNA was extracted and reverse-transcribed into cDNA for qRT-PCR detection. Reaction system was as follows: PCR forward primer (2 *μ*M) 2 *μ*L; PCR reverse primer (2 *μ*M) 2 *μ*L; 2X All-in-One qPCR Mix 10 *μ*L; Template 2 *μ*L; 50X Rox Reference Dye 0.4 *μ*L; ddH20 3.6 *μ*L; total 20 *μ*L. The reaction conditions were set as follows: predenaturation: 95°C 10 min, 1 cycle; amplification reaction: 95°C 10 S, 62°C 20 s, 72°C 15 S, 45 cycles; draw melting curve: 95°C 10 S, 25°C 30 s. Each sample was repeated 3 times. For data analysis, the 2^−ΔCt^ method was used to calculate the difference in Wnt3a and *β*-catenin gene mRNA transcription levels. The gene sequence was searched in the NCBI database, and primers were designed, as shown in Table 2.

### 2.8. Statistical Analysis

SPSS22.0 software was used for statistical analysis. The measurement data are expressed by mean ± standard deviation (*x* ± *s*), and the comparison between groups adopts multisample mean comparison and analysis of variance. The test level adopts *α* = 0.05; *P* < 0.05 indicates that the difference is statistically significant.

### 2.9. Molecular Docking Validation

The top 6 components in the compound-compound target network of LDD were selected as ligand, and ESR1, *β*-catenin (CTNNB1), and JAK2 were selected as receptors. The “sdf” format file of the top 6 components was input into ChemBioDraw for energy minimization and saved as “mol2” format. The PDB database (https://www.rcsb.org/) was used to retrieve the 3D structure of ESR1 (PDB ID: 1QKT), CTNNB1 (PDB ID: 1JDH), and JAK2 (PDB ID: 3E62) [36]. Discovery Studio Client Ver. 4.5 software was used to hydrogenate proteins, remove water, and remove ligand molecules. Auto Dock ver. 4.2 software was used for molecular docking. The binding energy ≤−5.0 kJ/mol was used as a standard to screen ligand-receptor molecules that can bind stably [37–39].

## 3. Results and Discussion

### 3.1. Osteoporosis Genes and the Potential Targets of LDD

**Figure 2:**
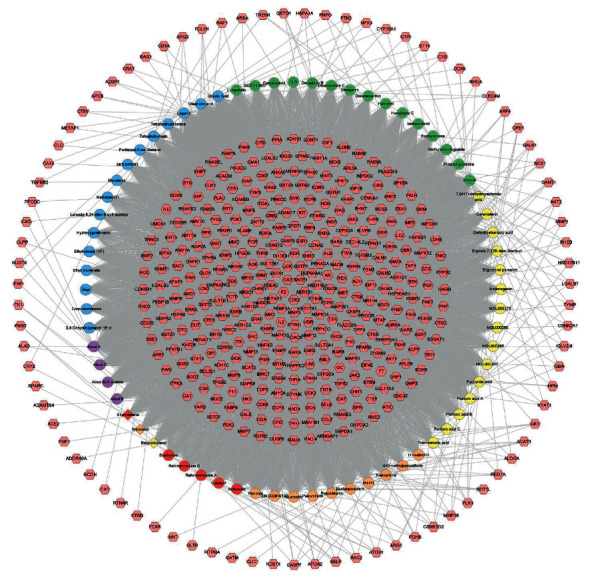
Compound-compound target network of LDD. Pink hexagons represent compound targets; red, orange, yellow, green, blue, and purple circles represent Rehmanniae Radix Praeparata, Cortex Moutan, *Poria cocos* (Schw.) Wolf., Rhizoma Dioscoreae*, Cornus officinalis* Sieb. et Zucc., *Alisma orientale* (Sam.) Juz., resp. The red diamond represents a common compound of *Rehmanniae Radix Praeparata, Rhizoma Dioscoreae*, and *Cornus officinalis Sieb. et Zucc.* The orange diamond represents a common compound of *Cornus officinalis* Sieb. et Zucc. and *Cortex Moutan.* The yellow diamond represents the common compound of *Cornus officinalis* Sieb. et Zucc. and *Rehmanniae Radix Praeparata*.

Two thousand and nine hundred and eighty-three osteoporosis genes were obtained from GeneCards and OMIM database. The osteoporosis genes with a relevance score ≥5.0 were selected to construct the LDD-osteoporosis PPI network (see Table S2). A total of 423 LDD potential targets were predicted from PharmMapper. The relationship between LDD compounds and LDD potential targets is shown in Figure 1, which consists of 423 compound targets, 68 compounds, and 10009 edges. In Figure 2, targets near the center are regulated by more compounds, whereas targets near the periphery are regulated by fewer compounds. For example, HSP90AA1, CDK2, GSTP1, AKR1B1, BACE1, CA2, F2, LCK, and GSTA1 are regulated by all compounds; ACE2 can be only regulated by chlorogenic acid.

### 3.2. LDD-Osteoporosis PPI Network Analysis

#### 3.2.1. LDD-Osteoporosis PPI Network

**Figure 3:**
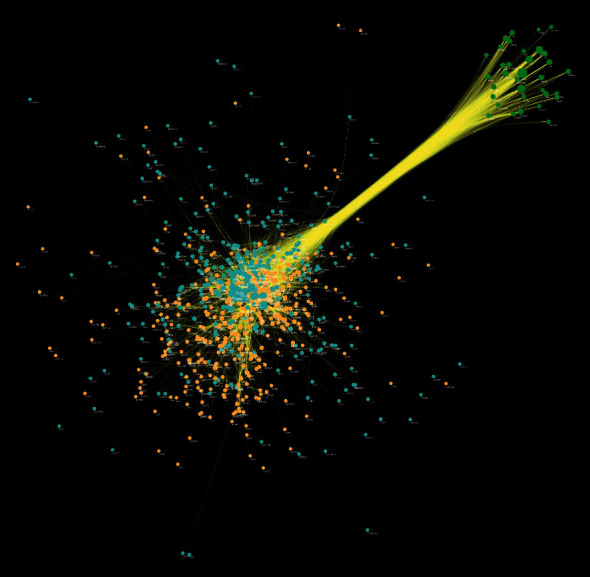
LDD-osteoporosis PPI network; blue circle stands for osteoporosis genes; orange circle stands for LDD potential targets; green circle stands for LDD-osteoporosis targets. The size of each node is related to its degree; the bigger nodes have a larger value of degree. The width of the line is associated with its edge betweenness; the wider lines have a larger value of edge betweenness.

The target shared by LDD potential target and osteoporosis genes is LDD-osteoporosis targets. The LDD-osteoporosis PPI network contains 743 nodes (379 LDD target nodes, 35 LDD-osteoporosis nodes, and 329 osteoporosis genes nodes) and 8081 edges (Figure 3). After analysis of the LDD-osteoporosis PPI network, it was found that LDD can directly and indirectly regulate the core target of osteoporosis. The top 20 targets are selected and divided into three categories: (1) osteoporosis genes: INS (300 edges), TP53 (248 edges), IL6 (245 edges), TNF (207 edges), MAPK3 (202 edges), EGF (199 edges), JUN (166 edges), CTNNB1 (161 edges), and CCND1 (149 edges); (2) LDD targets: AKT1 (253 edges), EGFR (205 edges), MAPK1 (181 edges), MMP9 (165 edges), MAPK8 (161 edges), HRAS (151 edges), and CASP3 (150 edges); (3) LDD-osteoporosis targets: ALB (275 edges), IGF1 (187 edges), SRC (173 edges), and ESR1 (168 edges).

#### 3.2.2. Clusters of LDD-Osteoporosis PPI Network

**Figure 4:**
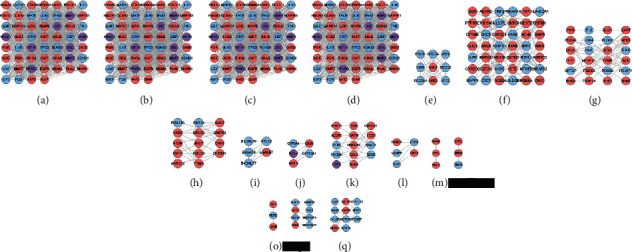
Clusters of LDD-osteoporosis PPI network A, B, C, D, E F, G H, I J, K L, M N, O P, Q stand for clusters 1, 2, 3, 4, 5, 6, 7, 8, 9, 10, 11, 12, 13; blue circle stands for osteoporosis gene; pink circle stands for LDD potential targets; purple circle stands for LDD-osteoporosis targets.

The Cytoscape's plug-in MCODE was utilized to analyze the LDD-osteoporosis PPI network, and 13 clusters were obtained (Table 3 and Figure 4). The targets and genes in clusters were input into the DAVID database to undergo GO enrichment analysis.

Cluster 1 is mainly involved in several signaling pathways in osteoporosis (such as MAPK, PI3K, and JAK-STAT signaling pathway) and positive regulation of calmodulin 1-monooxygenase activity, estrogen metabolism, endothelial cell proliferation, and neovascularization during bone remodeling. Cluster 2 is mainly involved in endothelial cell proliferation in bone remodeling, differentiation of osteoblasts and chondrocytes, bone resorption, and some signaling pathways (such as PI3K, MAPK, NF-*κ*B, TGF-beta/SMAD, BMP, and Wnt signaling pathway). Cluster 3 is associated with bone resorption and reconstruction, mesenchymal cell differentiation, osteoblast differentiation, osteogenic matrix, and osteoporosis-related signaling pathways (BMP, Wnt, and TGF-*β* signaling pathway). Cluster 4 is mainly involved in endothelial cell proliferation in bone remodeling and differentiation of osteoblasts and chondrocytes. Cluster 6 is mainly related to steroid metabolism. Cluster 10 is related to steroid metabolism. Cluster 15 failed to return any human biological processes. Clusters 5, 7, 8, 9, 11, 13, 14, 16 and 17 do not return any osteoporosis-related biological processes. The details of each cluster and biological processes are described in Table S3.

**Figure 5:**
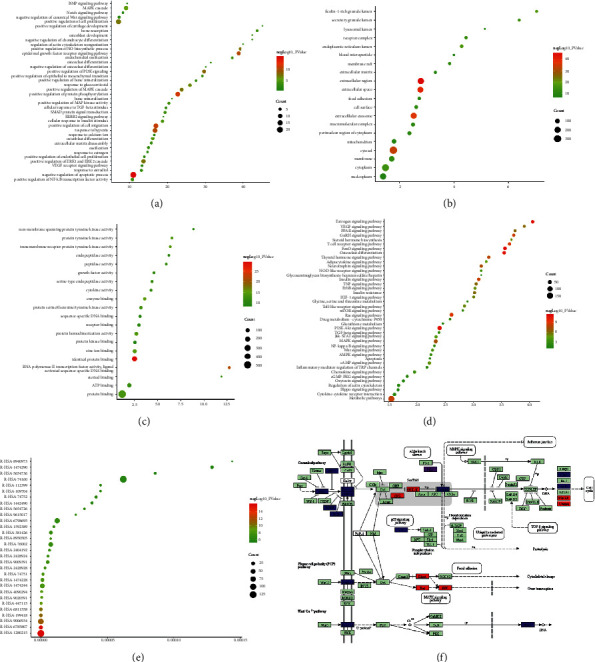
Bubble chart. (a) Biological processes of cluster 1. (b) Cell components. (c) Molecular function. (d) Signaling pathways; *X*-axis stands for fold enrichment. (e) Reactome pathways; *X*-axis stands for FDR. (f) The role of LDD in the Wnt signaling pathway modified from hsa04310.

Since cluster 1 is the most important one, it is used as an example to show its main biological processes on the bubble chart (Figure 5(a)).

#### 3.2.3. Cell Components, Molecular Functions, and Signaling Pathway of LDD-Osteoporosis PPI Network

The LDD targets and osteoporosis in the LDD-osteoporosis PPI network were input into DAVID to collect cell component, molecular functions, and signaling pathways. The top 10 cell components were as follows: extracellular region, extracellular space, extracellular exosome, cytosol, ficolin-1-rich granule lumen, endoplasmic reticulum lumen, cytoplasm, secretory granule lumen, receptor complex, and membrane (Figure 5(b)). The top 10 molecular functions were as follows: identical protein binding, RNA polymerase II transcription factor activity, ligand-activated sequence-specific DNA binding, enzyme binding, zinc ion binding, transmembrane receptor protein tyrosine kinase activity, protein tyrosine kinase activity, protein homodimerization activity, protein binding, protein serine/threonine/tyrosine kinase activity, and ATP binding (Figure 5(c)). The top 10 pathways were as follows: FoxO signaling pathway, estrogen signaling pathway, osteoclast differentiation, PI3K-Akt signaling pathway, Ras signaling pathway, T-cell receptor signaling pathway, GnRH signaling pathway, metabolic pathways, and insulin signaling pathway (Figure 5(d)). The role of LDD in the Wnt signaling pathway is shown in Figure 5(e). The LDD targets are marked in red, the osteoporosis gene is marked in blue, and the LDD-osteoporosis is marked in purple (see Table S4).

**Figure 6:**
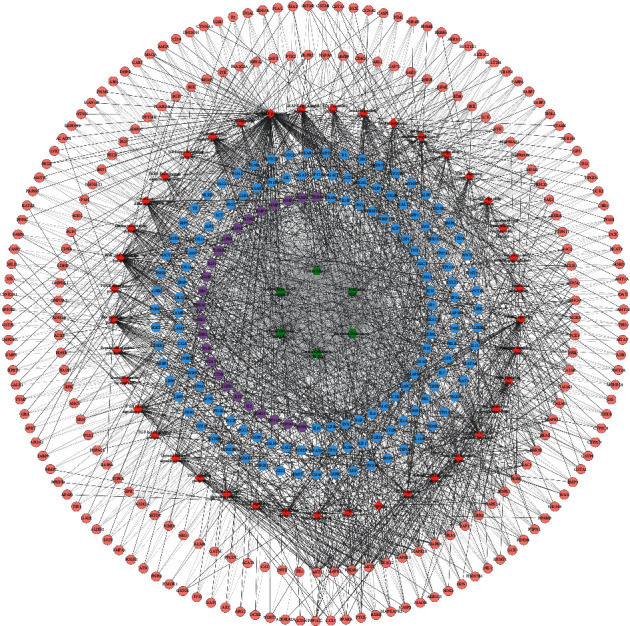
Pathway of LDD-osteoporosis PPI network. Blue circle stands for osteoporosis gene; pink circle stands for LDD target. Purple circle stands for LDD-osteoporosis targets; green hexagon stands for herb; red diamond stands for signaling pathway.

Meanwhile, the compounds of *Cornus officinalis* Sieb. et Zucc. totally regulate 228 targets (which is the most), while those of Rhizoma Dioscoreae regulate 216 targets. The compounds of Rhizoma Dioscoreae and *Rhizoma Dioscoreae* regulate 206 targets, respectively. This suggests that *Cornus officinalis* Sieb. et Zucc. and *Rhizoma Dioscoreae* play a major role in LDD (Figure 6).

#### 3.2.4. Reactome Pathway of LDD-Osteoporosis PPI Network

The LDD targets and osteoporosis in the LDD-osteoporosis PPI network were input into Reactome Pathway Database to collect the Reactome pathways. These osteoporosis-related Reactome pathways were arranged according to the *P*-value from small to large, and it is found that interleukin-4 and interleukin-13 signaling is ranked first. According to the sorting, the other Reactome pathways (top 10) are as follows: Cytokine Signaling in Immune system, Signaling by Receptor Tyrosine Kinases, Negative regulation of the PI3K/AKT network, PI5P, PP2A and IER3 Regulate PI3K/AKT Signaling, Interleukin-12 family signaling, SUMOylation of intracellular receptors, Interleukin-12 signaling, Extracellular matrix organization, and Degradation of the extracellular matrix. The top 30 Reactome pathways are shown in Figure 5(e). The details of each Reactome pathways are shown in Table S5

At present, it was found that LDD has obvious antiosteoporosis effect in ovariectomized rats, which is mainly achieved by activating the Wnt/*β*-catenin signaling pathway [40]. Ge et al. found that in patients with postmenopausal osteoporosis (PMOP) with Shen (Kidney) yin deficiency, the therapeutic effect of LDD may be mediated through upregulation of CLCF1 gene and IRF1 gene expression and activation of JAK/STAT signaling pathway [12, 41]. Xu et al. found that LDD can significantly inhibit the methylation of ER*α* gene promoter and increase the expression of ER*α* mRNA and protein and promote the proliferation and differentiation of osteoblasts by AMPK and *β*-catenin signaling mediated by this pathway [42]. In addition, in exploring the plant estrogen activity of LDD, new studies have shown that in the ovariectomized rat model, enhanced estrogen activity occurs when combined with the soy diet with LDD [43]. Moreover, a large number of studies confirmed that the active compounds in LDD's herbs play an antiosteoporosis role.

For example, Luo et al. found that Rehmanniae Radix Praeparata extract has osteoprotective effects, the mechanism of which is related to the IGF-1/PI3K/mTOR pathway [44]. Lai et al. showed that Th1/Th2 skew is associated with bone loss caused by estrogen deficiency, and catalpol can effectively reduce bone loss by regulating Th1/Th2 balance [45]. Zhu et al. found that catalpol promotes osteogenic differentiation of bone marrow mesenchymal stem cells via the Wnt/*β*-catenin pathway [46]. Ferulic acid can alter specific aspects of the bone epigenome to improve osteoblast differentiation, reduce osteoblast apoptosis, improve bone mineralization, and reduce osteoclast differentiation and function [47, 48]. Ferulic acid can also inhibit osteoclast fusion by inhibiting the expression of RANKL/DC-STAMP and induce apoptosis of mature osteoclasts via the caspase-3 pathway [49]. Paeonol can inhibit RANKL-induced osteoclastogenesis by inhibiting ERK, p38, and NF-*κ*B pathways [50]. Further studies showed that its derivatives, YPH-PA3, promote the differentiation of precursor cells of the monocyte/macrophage lineage into osteoblasts and enhance their autophagy [51]. Kaempferol in LDD has a strong antiosteoporosis effect [52–56]. Evidence from different teams shows the mechanism of acteoside against osteoporosis [57, 58]. Quercetin in LDD was also found to have antiosteoporosis effects [59, 60]. In addition, the metabolites of quercetin upregulate the antioxidant capacity of osteoblasts isolated from the fetal rat skull [61]. Paeoniflorin can regulate osteoclastogenesis and osteoblastogenesis both in vitro and in vivo experiments [62, 63]. (+)-Catechin can significantly inhibit RANKL-induced osteoclastogenesis by inhibiting NF-*κ*B transcriptional activity and nuclear transport [64, 65]. Furthermore, (+)-catechin inhibits IGF-I-induced osteoblast migration via p44/p42 MAP kinase [66]. Dioscin can reduce bone loss by enhancing osteoblast production and inhibiting osteoclastogenesis [67, 68]. Diosgenin also plays a role in antiosteoporosis. It can inhibit osteoclastogenesis, stimulates the osteogenic activity of osteoblasts in vitro, and exerts some antiosteoporosis effects on rats in vivo [69–72]. Ursolic acid primarily regulates the homeostasis of osteoclasts and osteoblasts to regulate osteoporosis [73–75]. Oleanolic acid combined with ursolic acid can increase BMD and improve microstructure in aged female rats, which may be potential candidates for the prevention and treatment of osteoporosis [76]. Loganin, alisol A 24-acetate, and alisol B also have antiosteoporosis effects [77–79].

Through the study of small molecule compounds, we determined the synergy between the main active compounds of LDD in the treatment of osteoporosis. Meanwhile, through the construction and analysis of the network, we obtained important targets for LDD and the potential biological processes and signaling pathways for the treatment of osteoporosis (such as positive regulation of calmodulin 1-monooxygenase activity, estrogen metabolism, endothelial cell proliferation, JAK-STAT signaling pathway, osteoclast differentiation, and degradation of the extracellular matrix). However, since the above processes are all completed by computer simulation, experiments are still needed for further verification. Therefore, we used the human transcriptomics data in the GEO database (GSE57273) and protein arrays data in reference [30] for further verification.

### 3.3. Human Transcriptomics Data Analysis

#### 3.3.1. Human Transcriptomics Data

**Figure 7:**
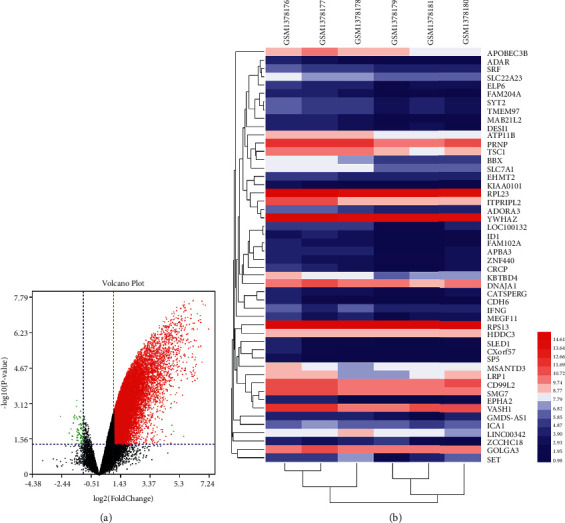
Human transcriptomics data. (a) Volcano plot; red point stands for upregulated gene; green point stands for downregulated gene. Black point stands for gene that is not differentially expressed. (b) Gene expression matrix of osteoporosis-related genes.

The transcriptomics data from GSE57273 were collected from GEO. A total of 45220 genes with their data were obtained (Table S9). The genes with log2FC ≥1 or ≤−1 and *P*-value <0.05 were thought to be differentially expressed genes (Figure 7(a)). The gene expression matrix of osteoporosis-related genes is shown in Figure 7(b); take 50 genes as an example (Table S6).

To further analyze the transcriptomics data, the identified genes with log2FC ≥2 or ≤−2 and *P*-value <0.01 (significantly differentially expressed genes) were selected. After the selection, a total of 4031 genes were obtained. Finally, the top 3000 genes were selected for enrichment analysis.

#### 3.3.2. Enrichment Analysis for Human Transcriptomics Data

**Figure 8:**
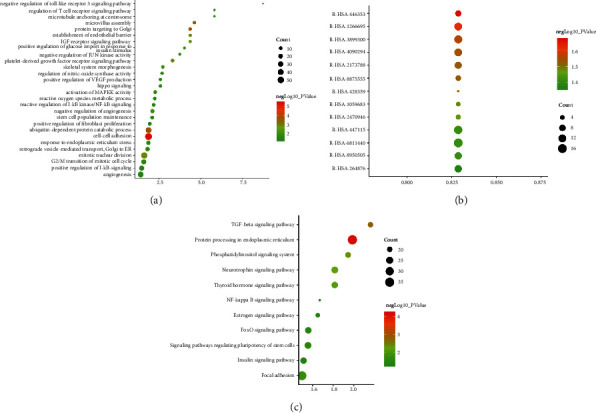
Bubble chart of biological processes, Reactome, and signaling pathways. (a) Bubble chart of biological processes; (b) bubble chart of Reactome pathways; (c) bubble chart of signaling pathways. *X*-axis in A and C stands for fold enrichment and *X*-axis in B stands for FDR.

After the enrichment analysis, several biological processes, Reactome pathways, and signaling pathways are obtained. The results of GO enrichment are mainly related to insulin-like growth factor and its receptor signaling pathway (GO:0048009), extracellular matrix (GO: 0098609), endothelial cell formation (GO:0061028), skeletal system morphogenesis (GO: 0048705), several osteoporosis-related signaling (such as hippo signaling, MAPK signaling, regulation of T-cell receptor signaling pathway), and so on (Figure 8(a)).

The results returned by Reactome pathways enrichment show that LDD is able to regulate Cell-extracellular matrix interactions, Interleukin-7 signaling, SUMOylation of transcription cofactors, SUMOylation of intracellular receptors, Downregulation of TGF-beta receptor signaling, MET activates RAP1 and RAC1, Insulin-like Growth Factor-2 mRNA Binding Proteins (IGF2BPs/IMPs/VICKZs) bind RNA, Interleukin-6 signaling, Cohesin Loading onto Chromatin, Interleukin-12 family signaling, and so on (Figure 8(b)).

The results of signaling pathway enrichment are protein processing in the endoplasmic reticulum, TGF-beta signaling pathway, Phosphatidylinositol signaling system, Neurotrophin signaling pathway, Thyroid hormone signaling pathway, Focal adhesion, Signaling pathways regulating pluripotency of stem cells, FoxO signaling pathway, Estrogen signaling pathway, Insulin signaling pathway, and NF-kappa B signaling pathway (Figure 8(c)). The details of biological processes, Reactome pathways, and signaling pathways are described in Tables S7–S9.

### 3.4. Protein Arrays Data Analysis

#### 3.4.1. Protein Arrays Data

**Figure 9:**
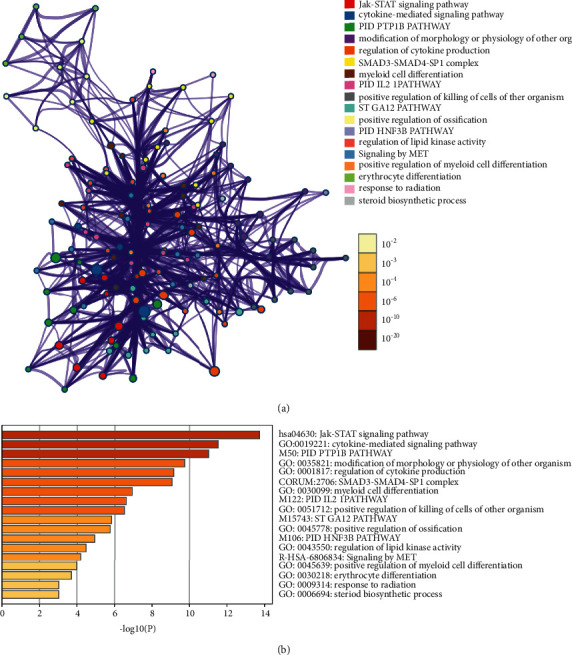
Osteoporosis-related protein arrays data: (a) proteomics data network; (b) the top signaling pathway, signaling processes, and Reactome pathway.

The osteoporosis-related protein arrays data comes from [30]. The network was constructed by Metascape (Figure 9).

#### 3.4.2. Enrichment Analysis for Protein Arrays Data

**Figure 10:**
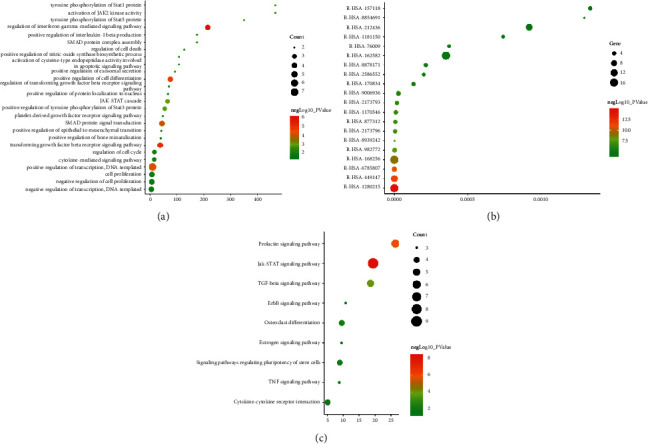
Bubble chart of biological processes, Reactome, and signaling pathways. (a) Bubble chart of biological processes; (b) bubble chart of Reactome pathways; (c) bubble chart of signaling pathways. *X*-axis in A and C stands for fold enrichment and *X*-axis in B stands for FDR.

Protein arrays data were input into DAVID and Reactome for enrichment analysis and returned several biological processes, Reactome pathways, and signaling pathways. The results of GO enrichment are shown in Figures 10(a)–10(c).

The results of GO enrichment are mainly related to immune response (GO:0060334), cell proliferation (GO:0045597, GO:0040008, GO:0042127, GO:0007179, GO:0060395, GO:0060397), and other aspects (GO:1903543, GO:0010718, GO:0030501) (Figure 10(a)).

The results of signaling pathway enrichment are related to bone homeostasis (such as hsa04630: Jak-STAT signaling pathway, hsa04380:Osteoclast differentiation, hsa04550:Signaling pathways regulating pluripotency of stem cells, and hsa04012:ErbB signaling pathway) and other pathways (such as hsa04060:Cytokine-cytokine receptor interaction) (Figure 10(b)).

The results returned by Reactome pathways enrichment are associated with immunomodulatory (such as R-HSA-1280215, R-HSA-449147, R-HSA-6785807, R-HSA-168256, and R-HSA-8877330), cell growth, proliferation, and cell cycle (such as R-HSA-9617828 and R-HSA-452723), transcription factor and coactivated genes that interact with STAT proteins (R-HSA-2173796 and R-HSA-9006936), and other transcription factors and their coactivated genes (R-HSA-2173789 and R-HSA-76002) (Figure 10(c)). The details of biological processes, Reactome pathways, and signaling pathways are described in Tables S10–S12.

In summary, we found that the mechanism of LDD in the treatment of osteoporosis is related to immunity, cell growth and proliferation, endocrine hormones and cytokines, and differentiation of osteoclasts and osteoblasts. These pathways work together to regulate the activity of osteoclasts and balance the process of bone turnover through the interaction of crucial proteins. Next, animal experiments were conducted to verify the mechanism of LDD on osteoporosis.

### 3.5. Effect of LDD on BMD of Proximal Femur

**Figure 11:**
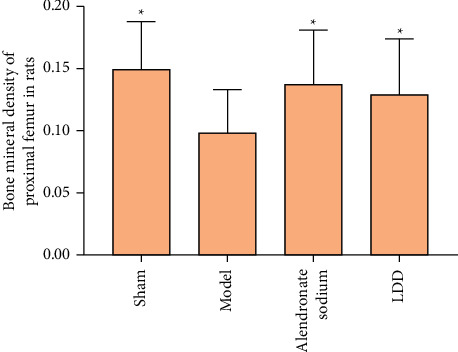
Effect of LDD on BMD of proximal femur (^*∗*^compared with the model group, *P* < 0.01).

The BMD of the proximal femur in the model group was lower than that in the sham operation group (*P* < 0.01). The BMD of the proximal femur in the LDD group and the positive control group was higher than that in the model group (*P* < 0.01). Compared with the positive control group, the BMD of the proximal femur in the LDD group was not statistically significant (*P* > 0.05) (Figure 11).

### 3.6. Effect of LDD on Serum E2 and ALP

**Figure 12:**
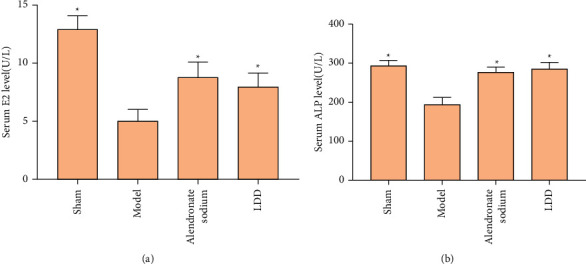
Effect of LDD on serum E2 and ALP. (a) Serum E2 level; (b) serum APL level; ^*∗*^compared with the model group, *P* < 0.05.

Compared with the model group, the serum E2 and ALP of the LDD group were significantly improved (*P* < 0.05), suggesting that LDD can increase serum E2 and ALP levels in osteoporotic rats (Figure 12).

### 3.7. Effect of LDD on Bone Histopathology

**Figure 13:**
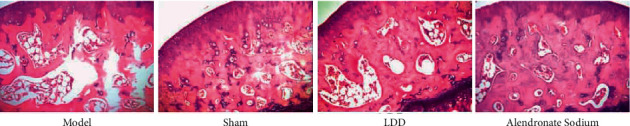
Pathological morphological changes (400X, HE staining).

In the sham operation group, dense and regular bone trabeculae were arranged in the bone tissue of the rats, and the morphological structure was complete, and the bone marrow cavity size was normal. Compared with the model group, the trabecular bones of the LDD group were regularly arranged, with few fractures, morphological structure close to normal, and the size of the bone marrow cavity was normal, similar to the sham operation group. It is suggested that LDD can improve the destruction of bone tissue structure in PMOP rats (Figure 13).

### 3.8. Effect of LDD on Wnt3a and *β*-Catenin mRNA Expression

**Figure 14:**
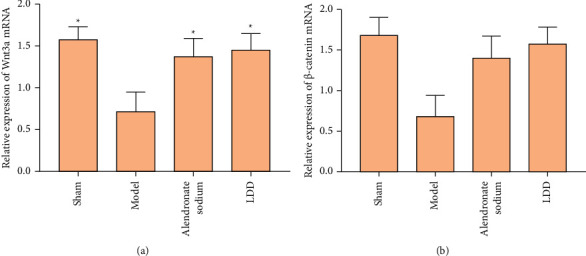
Effect of LDD on Wnt3a and *β*-catenin mRNA expression. (a) Wnt3a expression; (b) *β*-catenin expression. ^*∗*^Compared with model group, *P* < 0.05.

Compared with the sham operation group, the Wnt3a and *β*-catenin mRNA expression of the model group decreased (*P* < 0.05). Compared with the model group, Wnt3a and *β*-catenin were significantly increased in the LDD group and the positive control group (*P* < 0.05), similar to the sham operation group. It is suggested that LDD can improve osteoporosis in rats by regulating the expression of Wnt3a and *β*-catenin (Figure 14).

### 3.9. Molecular Docking Results of LDD Components and Osteoporosis-Related Gene

**Figure 15:**
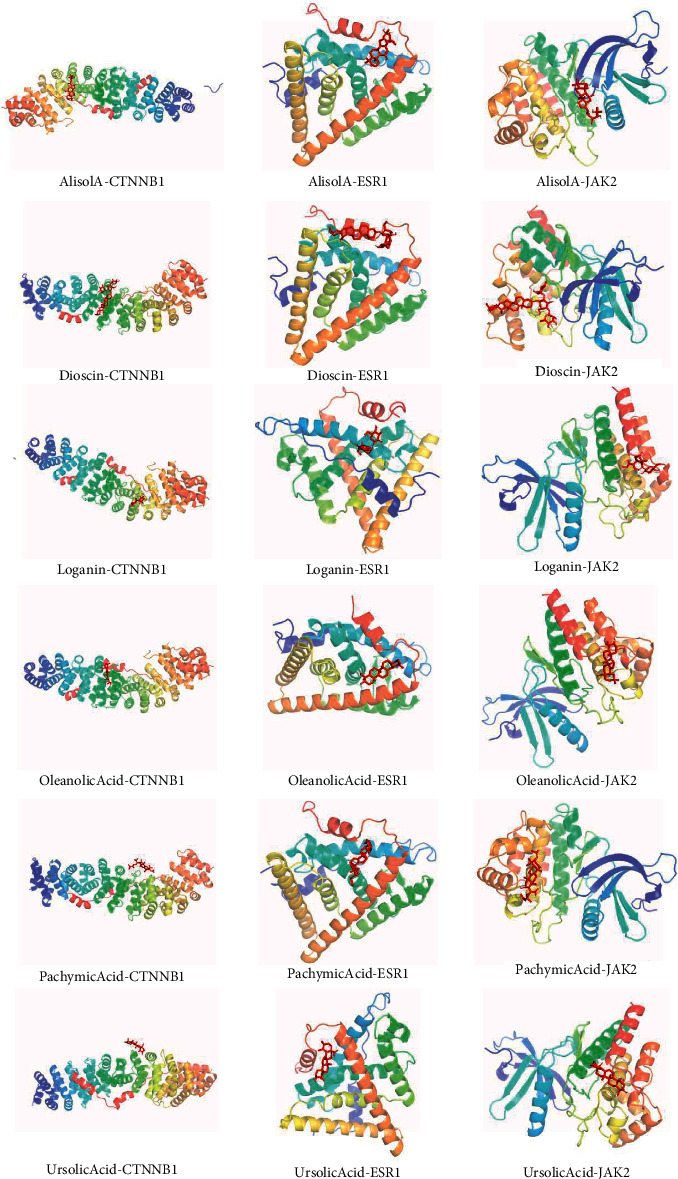
Docking mode of LDD components with JAK2, ESR1, and CTNNB1.

Due to the limitations of the prediction database, this study used molecular docking technology to further explore whether the LDD components can directly interact with osteoporosis-related genes. The lowest binding energy between the LDD component and the target protein is less than −5 kJ/mol, indicating that the ligand and the receptor can bind spontaneously and stably. The results are shown in Table 4. The docking mode of LDD components with JAK2, ESR1, and CTNNB1 is shown in Figure 15.

Comparing the pathways of the LDD-osteoporosis PPI network and human transcriptomics data, it was found that the osteoporosis-related pathways they share are TGF-beta signaling pathway, Neurotrophin signaling pathway, Thyroid hormone signaling pathway, FoxO signaling pathway, Estrogen signaling pathway, Insulin signaling pathway, and NF-kappa B signaling pathway. The unique osteoporosis-related pathways in enrichment results of human transcriptomics data were as follows: Protein processing in endoplasmic reticulum, Phosphatidylinositol signaling system, Focal adhesion, and Signaling pathways regulating pluripotency of stem cells. Comparing the pathways of protein arrays data network and LDD-osteoporosis PPI network, it is found that the osteoporosis-related pathways they share are Jak-STAT signaling pathway, TGF-beta signaling pathway, Osteoclast differentiation, ErbB signaling pathway, Estrogen signaling pathway, Cytokine-cytokine receptor interaction, and TNF signaling pathway. The unique osteoporosis-related pathways in enrichment results of protein arrays data were as follows: Prolactin signaling pathway and Signaling pathways regulating pluripotency of stem cells [80, 81].

The skeletal structure undergoes subtle changes throughout life, requiring the involvement of osteoblasts and osteoclasts [82]. The two cells interact at the same site on the bone surface, in which osteoclasts are involved in bone resorption, which weakens bone strength and causes osteoporosis when bone resorption is more than bone formation [82]. The current intervention in osteoporosis is to inhibit bone resorption—osteoclast activity regulation [83]. Osteoclasts are matured from mononuclear-macrophage cell lines, mainly regulated by macrophage colony-stimulating factor (MCSF), nuclear stimulating factor-*κ*B receptor ligand (RANKL), and osteoprotegerin (OPG) [84, 85], of which MCSF is a key factor in the regulation of osteoclast differentiation [86]. Bone biopsy data and in vitro studies have shown that osteoclasts that complete the bone resorption mission will automatically undergo apoptosis, which is one of the mechanisms for the body to maintain bone metabolism balance [86]. The regulation of these cytokines on osteoclast activity is mainly reflected in [87–90]: (1) the auto/paracrine effect of local cells directly regulates the proliferation and differentiation of immature osteoblasts and bone resorption activities; (2) they can indirectly increase the sensitivity of bone resorption associated with parathyroid hormone (PTH); (3) inhibition of osteoclast activity by the interaction of signaling pathways. Among them, the RANKL/RANK system is the most irreplaceable regulation of bone cell unit activity, and various external factors play a role in changing the signaling pathway in this system [86]. For example, L-1a, IL-6, IL-11, IL-16, and PTH can activate the RANKL/RANK system to enhance RANKL expression, thus completing bone remodeling [91]. The signaling pathways associated with the RANKL/RANK system are mainly the NF-*κ*B pathway, MAPK pathway, PI3K/AKt pathway, and CN/NFAT pathway [92]. Hence, the RANKL/RANK system plays a role similar to the common terminal pathway during osteoclast activation; and the drugs designed by the system, such as denosumab, have many advantages in the clinical practice of treating osteoporosis, such as fewer side effects [93].

MCSF is produced by various cells in the bone microenvironment and acts on the osteoclasts in a paracrine manner, resulting in increased expression of genes involved in osteoclast differentiation and increased osteoclast activity [94]. In addition, MCSF can also promote the formation of mature osteoclasts indirectly through the RANKL pathway [94]. Osteoprotegerin (OPG) is mainly expressed on the surface of osteoblasts and inhibits the activation of osteoclasts and its bone resorption activity by competitively inhibiting the binding of RANKL to RANK [95]. Meanwhile, prostaglandins (PGs), metastatic growth factors (TGF-*β*), interferon (INF), and insulin-like growth factor (IGF) are also associated with the activation of osteoclasts [96].

Hormones also play an important role in the process of bone regulation, especially the regulation of osteoclast activity [97]. For example, estrogen plays an important role in inhibiting bone resorption, and various forms of hypogonadism that lack estrogen can lead to osteoporosis [97]. The mechanism is that estrogen can act on ER*α* of osteoclasts, stimulate OPG expression, inhibit the activation of osteoclast-associated cytokine expression, and ultimately reduce the rate of osteoclastogenesis through the RANKL signaling pathway [98]. For this target, the main drugs are selective estrogen receptor modulator (raloxifene) and artificial estrogen to inhibit bone resorption, treat osteoporosis, and at the same time relieve menopausal symptoms in menopausal women [99]. Other hormones, such as calcitonin, are also associated with inhibition of bone resorption, and their short-term efficacy in the treatment of osteoporosis is obvious, but long-term efficacy is poor [100]. The hormones that act indirectly on bone resorption are PTH and glucocorticoids [101]. The synthetic PTH analog, teriparatide, enhances osteoclast function by promoting the lysosomal enzyme production and acid synthesis in osteoclasts and through the AC/cAMP pathway [101].

Through the construction and enrichment analysis of the predicted target network of small molecule compounds in LDD, we can find that the small molecule compounds of LDD can regulate some biological processes. For example, they can regulate endothelial cell proliferation and neovascularization, bone formation, bone resorption and reconstruction, differentiation of mesenchymal stem cells, formation of extracellular matrix and collagen of bone formation, differentiation of osteoblasts, and osteoclasts, and so on. These biological processes play an important role in the development of osteoporosis. Meanwhile, this study suggests that LDD can regulate some cytokines related to osteoporosis, such as TGF-*β*, INF, IGF, and calmodulin 1-monooxygenase, which are closely associated with the activation of osteoclasts. [94, 102]. The GO enrichment analysis of LDD has also shown that it can regulate the production and metabolism of hormones such as estrogen and glucocorticoids; these hormones can regulate osteoclast activity directly (estrogen) or indirectly (glucocorticoids), thereby regulating bone resorption [103]. The results of the pathway enrichment analysis show that LDD can regulate the FoxO signaling pathway, Estrogen signaling pathway, Osteoclast differentiation, PI3K-Akt signaling pathway, Ras signaling pathway, T-cell receptor signaling pathway, and so on. These pathways are closely related and synergistic, acting together on the bone resorption process, regulating osteoclast activity [104], and ultimately regulating the core mechanism of osteoporosis—bone turnover. The current drug development of osteoporosis has also developed targeted drugs for the above multiple pathways, such as denosumab (for OPG/RANKL/RANK pathway) [105], romosozumab (for Wnt signaling pathway) [106], and rapamycin (for mTOR signaling pathway) [107].

The most important signaling pathways associated with osteoporosis are the OPG/RANKL/RANK signaling pathway, which includes NF-*κ*B signaling, MAPK signaling, PI3K/AKt signaling, and CN/NFAT signaling [92, 96]. The MAPK family pathway mainly includes the extracellular-regulated protein kinase (ERK) signaling pathway, Jun N-terminal kinase (JNK) signaling pathway, the ERK5/macroton activating protein kinase signaling pathway, and the p38 signaling pathway [108]. In addition, the signaling pathway with osteoporosis includes PPAR-*γ* [109], Wnt/*β*-catenin [110], Hedgehog [111], BMP [112], Notch [113], JAK/STAT [114], and TGF-*β*/SMAD signaling pathway [115].

In conclusion, although this study explored the molecular mechanism of LDD in the treatment of osteoporosis through bioinformatics and systems pharmacology and transcriptomics and verified some related molecular mechanisms, its molecular mechanism still needs to be further elucidated in order to promote the thorough disclosure and elucidation of the molecular network mechanism of LDD in the treatment of osteoporosis.

## 4. Conclusion

LDD may have a therapeutic effect on osteoporosis through regulating the targets (such as ALB, IGF1, SRC, and ESR1), biological processes (such as positive regulation of calmodulin 1-monooxygenase activity, estrogen metabolism, and endothelial cell proliferation), and pathways (such as JAK-STAT signaling pathway, osteoclast differentiation, and degradation of the extracellular matrix) found in this research.

## Figures and Tables

**Table 1:** The pharmacokinetic parameters of components.

Molecule	OB (%)	Caco-2	DL
Sitosterol	36.91	1.32	0.75
Stigmasterol	43.83	1.44	0.76
Paeoniflorin	68.18	−0.34	0.4
Mairin	55.38	0.73	0.78
Kaempferol	41.88	0.26	0.24
(+)-Catechin	54.83	−0.03	0.24
4-O-Methylpaeoniflorin	67.24	0.15	0.43
5-[[5-(4-Methoxyphenyl)-2-furyl]methylene]barbituric acid (ZINC02816192)	43.44	0.09	0.3
Mudanpioside H	42.36	−0.39	0.37
Paeonidanin	65.31	−0.09	0.35
Quercetin	46.43	0.05	0.28
(2R)-2-[(3S,5R,10S,13R,14R,16R,17R)-3,16-Dihydroxy-4,4,10,13,14-pentamethyl-2,3,5,6,12,15,16,17-octahydro-1H-cyclopenta[a]phenanthren-17-yl]-6-methylhept-5-enoic acid (MOL000273)	30.93	0.01	0.81
Trametenolic acid	38.71	0.52	0.8
7,9 (11)-Dehydropachymic acid	35.11	0.03	0.81
Cerevisterol	37.96	0.28	0.77
(2R)-2-[(3S,5R,10S,13R,14R,16R,17R)-3,16-Dihydroxy-4,4,10,13,14-pentamethyl-2,3,5,6,12,15,16,17-octahydro-1H-cyclopenta [a]phenanthren-17-yl]-5-isopropyl-hex-5-enoic acid (MOL000280)	31.07	0.05	0.82
Ergosta-7,22E-dien-3beta-ol	43.51	1.32	0.72
Ergosterol peroxide	40.36	0.84	0.81
(2R)-2-[(5R,10S,13R,14R,16R,17R)-16-Hydroxy-3-keto-4,4,10,13,14-pentamethyl-1,2,5,6,12,15,16,17-octahydrocyclopenta[a]phenanthren-17-yl]-5-isopropyl-hex-5-enoic acid (MOL000285)	38.26	0.12	0.82
3beta-Hydroxy-24-methylene-8-lanostene-21-oic acid (MOL000287)	38.7	0.61	0.81
Pachymic acid	33.63	0.1	0.81
Poricoic acid A	30.61	−0.14	0.76
Poricoic acid B	30.52	−0.08	0.75
Poricoic acid C	38.15	0.32	0.75
Hederagenin	36.91	1.32	0.75
Dehydroeburicoic acid	44.17	0.38	0.83
Piperlonguminine	30.71	0.95	0.18
(-)-Taxifolin	60.51	−0.24	0.27
Denudatin B	61.47	0.9	0.38
Kadsurenone	54.72	0.82	0.38
Hancinol	64.01	0.53	0.37
Hancinone C	59.05	0.74	0.39
Campesterol	37.58	1.34	0.71
Isofucosterol	43.78	1.36	0.76
Dioscoreside C	36.38	0.39	0.87
Diosgenin	80.88	0.82	0.81
Doradexanthin	38.16	0.52	0.54
Methylcimicifugoside	31.69	0.21	0.24
AIDS180907	45.33	0.73	0.77
CLR	37.87	1.43	0.68
Mandenol	42	1.46	0.19
Ethyl linolenate	46.1	1.54	0.2
Poriferast-5-en-3beta-ol	36.91	1.45	0.75
Diop	43.59	0.79	0.39
Ethyl oleate (NF)	32.4	1.4	0.19
Malkangunin	57.71	0.22	0.63
2,6,10,14,18-Pentamethylicosa-2,6,10,14,18-pentaene (MOL005481)	33.4	1.94	0.24
3,4-Dehydrolycopen-16-al	46.64	2	0.49
Cornudentanone	39.66	0.47	0.33
Hydroxygenkwanin	36.47	0.52	0.27
Telocinobufagin	69.99	−0.12	0.79
Tetrahydroalstonine	32.42	0.9	0.81
Lanosta-8,24-dien-3-ol,3-acetate	44.3	1.45	0.82
Alisol B	34.47	0.04	0.82
Alisol B23 acetate	32.52	−0.06	0.82
Alisol C	32.7	−0.34	0.82
Acteoside^*∗*^	2.94	−1.89	0.62
Catalpol^*∗*^	5.07	−1.72	0.44
Rehmannioside A^*∗*^	25.95	−3	0.87
Rehmannioside D^*∗*^	—	—	—
Stachyose^*∗*^	3.25	−5.54	0.59

^*∗*^Components supplemented according to literature.

**Table 2:** Primers.

Gene	Orientation	Primer	Product length (bp)
Wnt3a	Sense	CGGGTTTCTACCTGATGGTG	123
	Antisense	CTTACCTGTCTCCGTTTGAGC	

*β*-Catenin	Sense	GTGCAATTCCTGAGCTGACC	184
	Antisense	CGGGCTGTTTCTACGTCATT	

**Table 3:** Cluster of LDD-osteoporosis PPI network.

Cluster	Score	Nodes	Edges	Targets and genes
1	32.962	53	857	JAK2, MET, MDM2, PPARG, GRB2, MAPK1, MMP2, TNF, EGFR, IL1B, APOE, BCL2L1, CCND1, AKT2, MMP1, LEP, IGF2, IFNG, EGF, NOS3, TP53, PLAU, FOS, CDC42, REN, CCL5, MMP3, MMP7, JUN, HRAS, NR3C1, CRP, LOX, HSP90AA1, SELE, MMP9, CCNA2, RHOA, MAPK3, SRC, CAT, ANXA5, XIAP, ACE, IL10, RUNX2, IGF1R, MAPK14, PGR, AR, MAP2K1, HMOX1, LCK

2	23.884	70	824	PTPN1, PRL, GSK3B, SMAD3, ELANE, IL2, IL6, TGFB1, CASP3, LMNA, PTPN11, CASP1, PARP1, CSK, TERT, FBN1, SERPINA1, MEN1, ERBB4, SPP1, MMP13, ADAM17, PIK3R1, CP, PTK2, FGG, SOD2, NQO1, CTNNB1, MAPK8, ALPL, APOA2, STAT1, INS, MAPK10, NOS2, ADIPOQ, SP1, PTK2B, ABL1, IGF1, AKT1, BMP15, IGFBP4, LGALS3, IGFBP1, IGFBP5, MEPE, BMP4, CDK2, JAK3, TRAF6, ESR1, SPARC, CTSB, ESR2, KIT, EIF4E, CD40LG, TF, HPGDS, KDR, BMP2, BGLAP, F2, RAF1, MMP14, ALB, TNFSF11, SOX9

3	9.901	82	401	SP7, COL2A1, EPHA2, DPP4, HSPA8, TEK, HSPA1A, AURKA, BMP7, WNT3A, PTH, CTSG, GNRH1, LDLR, POMC, TGFBR1, SOST, PRKACA, FABP5, GM2A, BTK, IL1A, ACP5, CALR, RAC2, SELP, CHEK1, GGT1, HEXB, JAG1, RHEB, CRH, GALNS, VCP, CYP19A1, PTHLH, RNASE2, CASP7, CSF1, DUSP6, IMPDH1, FGFR2, IBSP, ELN, GSR, LEPR, IL1RN, RNASE3, ATIC, EIF2AK3, LYZ, IL11, HCK, ZAP70, FGFR1, CDK6, VDR, PGF, IL7, WT1, FGF1, BACE1, NOG, TGM2, LRP5, COL1A1, CTSK, TNFRSF11B, PIK3CG, NFATC1, GATA4, CALCA, LCN2, WNT1, TTR, MAP3K1, APAF1, GLB1, COL1A2, DKK1, RAC1, ARSA

4	6.909	56	190	CDA, PROK2, FABP4, HBB, WAS, GHRL, PPARA, SLPI, PTH1R, HPRT1, SYK, PRKACB, SHBG, PRKCQ, IGFBP3, GNAI2, CANT1, HS6ST1, NSMF, CASR, LTA4H, CTSS, HP, CHIT1, KL, GLI2, OTX2, FGF23, DUT, GNRHR, ALDOA, TACR3, CHD7, BMP6, HSPG2, MAPK12, TGFBR2, AHSG, PRLR, FGF8, PDPK1, TAC3, BRAF, MGP, KISS1R, MMP8, CTSD, PROKR2, SULT2A1, HSP90AB1, CNR1, CALM1, TGFB2, ASAH1, RBP4, RXRA

5	6.75	9	27	PMS2, ERCC6, ERCC2, CDK7, NBN, RECQL4, RFC2, POLD1, WRN

6	4.293	42	88	PRKAR1A, NOP10, DTYMK, CYP2C8, TINF2, DKC1, MLXIPL, STS, HLA-DQB1, IMPDH2, GSTM1, MTHFR, ARG1, UMPS, HSD17B1, AKR1C3, NR1H4, CLIC1, RTEL1, IAPP, ENPP1, MME, ADH1C, ACE2, CTC1, HLA-DQA1, CYP2C9, PPP1CC, GSTA1, PDE5A, GCK, TK1, GMPR, BCHE, NHP2, IRF5, PARN, WRAP53, CMA1, GSTM2, ADH1B, RXRB

7	4.174	24	48	GLO1, CRTAP, APRT, GART, PDE8B, MTHFD1, PNP, FKBP1A, PPIB, BHMT, POU1F1, GH1, PLOD2, LHX4, F11, UCK2, NME2, PDE4B, PDE4D, ADK, PAH, DCK, F12, TYMP

8	4	14	26	NT5M, PDE3B, YARS, PDE11A, GMPR2, AKR1C2, AHCY, EPHX2, DHODH, PEX12, DHFR, AGXT, CRAT, TYMS

9	4	5	8	B4GALT7, LGALS7, XYLT2, B3GALT6, B3GAT3

10	3.5	5	7	NR1I2, CYP27A1, CYP3A4, NR1I3, CES1

11	3.077	14	20	FECH, GALE, ALDH2, FDPS, POR, SC5D, GNPDA2, SHMT1, PHGDH, GNPDA1, PSAT1, SDS, ALAD, GATM

12	3	5	6	RAB5A, FZD4, KIF11, VAMP7, NDP

13	3	3	3	TPI1, PDHB, PKLR

14	3	3	3	SMS, SRM, OTC

15	3	3	3	LDHB, AK1, PEPD

16	2.857	8	10	CD79A, GBA, FKBP10, PLS3, SERPINH1, SERPINF1, SORD, MALT1

17	2.4	11	12	GSTP1, FBXW7, GNAS, KAT2B, CALCR, NOTCH3, WNT16, CYP17A1, SFRP1, CGA, NR3C2

**Table 4:** The binding energy of molecules (kJ/mol).

Components	JAK2	ESR1	CTNNB1
Alisol A	−20.7207	−23.4835	−15.3626
Dioscin	−15.1115	−15.9068	−19.8416
Loganin	−15.4463	−16.1161	−12.1394
Oleanolic acid	−27.2509	−27.209	−22.437
Pachymic acid	−20.637	−15.1952	−13.5626
Ursolic acid	−27.9625	−29.3857	−22.8974

## Data Availability

The data that support the findings of this study are available in supplementary materials.

## Supplementary Materials

Supplementary Materials Table S1-1: components meeting the screening criteria. Table S1-2: compound targets for each compound of LDD. Table S2: osteoporosis genes. Table S3: enrichment analysis of clusters based on Gene Ontology (GO) annotation of LDD-osteoporosis PPI network. Table S4: pathway enrichment analysis of LDD-osteoporosis PPI network. Table S5: Reactome pathways of LDD-osteoporosis PPI network. Table S6: Human Transcriptomics Data. Table S7: the biological processes of Human Transcriptomics Data Network. Table S8: the Reactome pathways of Human Transcriptomics Data Network. Table S9: the signaling pathways of Human Transcriptomics Data Network. Table S10: the biological processes of protein arrays data network. Table S11: the Reactome pathways of protein arrays data network. Table S12: the signaling pathways of protein arrays data network.
