# Supplementary material for: Research on the Mechanism of Liuwei Dihuang Decoction for Osteoporosis Based on Systematic Biological Strategies
Source: Evid Based Complement Alternat Med. 2022 Sep 22;2022:7017610. doi: 10.1155/2022/7017610 (PMC9522519; doi:10.1155/2022/7017610)
Supplement: Supplementary Materials — Table S1-1: components meeting the screening criteria. Table S1-2: compound targets for each compound of LDD. Table S2: osteoporosis genes. Table S3: enrichment analysis of clusters based on Gene Ontology (GO) annotation of LDD-osteoporosis PPI network. Table S4: pathway enrichment analysis of LDD-osteoporosis PPI network. Table S5: Reactome pathways of LDD-osteoporosis PPI network. Table S6: Human Transcriptomics Data. Table S7: the biological processes of Human Transcriptomics Data Network. Table S8: the Reactome pathways of Human Transcriptomics Data Network. Table S9: the signaling pathways of Human Transcriptomics Data Network. Table S10: the biological processes of protein arrays data network. Table S11: the Reactome pathways of protein arrays data network. Table S12: the signaling pathways of protein arrays data network. [file 7017610.f1.zip › 7017610.f1/Table S10.pdf]

Table S10 The Biological Processes of Protein Arrays Data Network

Term	Biological processes	Count	%
GO:0060334	regulation of interferon-gamma-mediated signaling pathway	4	0.104466
GO:0007179	transforming growth factor beta receptor signaling pathway	5	0.130582
GO:0045597	positive regulation of cell differentiation	4	0.104466
GO:0045944	positive regulation of transcription from RNA polymerase II pr	9	0.235048
GO:0040008	regulation of growth	4	0.104466
GO:0045893	positive regulation of transcription, DNA-templated	7	0.182815
GO:0060395	SMAD protein signal transduction	4	0.104466
GO:0042127	regulation of cell proliferation	5	0.130582
GO:0060397	JAK-STAT cascade involved in growth hormone signaling path	3	0.078349
GO:0007259	JAK-STAT cascade	3	0.078349
GO:0010634	positive regulation of epithelial cell migration	3	0.078349
GO:0042517	positive regulation of tyrosine phosphorylation of Stat3 protein	3	0.078349
GO:0097191	extrinsic apoptotic signaling pathway	3	0.078349
GO:0045429	positive regulation of nitric oxide biosynthetic process	3	0.078349
GO:0035556	intracellular signal transduction	5	0.130582
GO:0000122	negative regulation of transcription from RNA polymerase II pr	6	0.156699
GO:0009612	response to mechanical stimulus	3	0.078349
GO:0009967	positive regulation of signal transduction	3	0.078349
GO:0043066	negative regulation of apoptotic process	5	0.130582
GO:0051712	positive regulation of killing of cells of other organism	2	0.052233
GO:0042508	tyrosine phosphorylation of Stat1 protein	2	0.052233
GO:0042977	activation of JAK2 kinase activity	2	0.052233
GO:0042506	tyrosine phosphorylation of Stat5 protein	2	0.052233
GO:0051098	regulation of binding	2	0.052233
GO:0032909	regulation of transforming growth factor beta2 production	2	0.052233
GO:0050731	positive regulation of peptidyl-tyrosine phosphorylation	3	0.078349
GO:0007166	cell surface receptor signaling pathway	4	0.104466
GO:0042493	response to drug	4	0.104466
GO:0042503	tyrosine phosphorylation of Stat3 protein	2	0.052233
GO:0032731	positive regulation of interleukin-1 beta production	2	0.052233
GO:0007183	SMAD protein complex assembly	2	0.052233
GO:1901522	positive regulation of transcription from RNA polymerase II pr	2	0.052233
GO:0051726	regulation of cell cycle	3	0.078349
GO:0008283	cell proliferation	4	0.104466
GO:0019221	cytokine-mediated signaling pathway	3	0.078349
GO:0010941	regulation of cell death	2	0.052233
GO:0008285	negative regulation of cell proliferation	4	0.104466
GO:0051770	positive regulation of nitric-oxide synthase biosynthetic proces	2	0.052233
GO:0097296	activation of cysteine-type endopeptidase activity involved in a	2	0.052233
GO:1903543	positive regulation of exosomal secretion	2	0.052233
GO:0007596	blood coagulation	3	0.078349
GO:1902895	positive regulation of pri-miRNA transcription from RNA poly	2	0.052233
GO:0017015	regulation of transforming growth factor beta receptor signalin	2	0.052233
GO:0007492	endoderm development	2	0.052233
GO:1900182	positive regulation of protein localization to nucleus	2	0.052233
GO:0045892	negative regulation of transcription, DNA-templated	4	0.104466

GO:0048008	platelet-derived growth factor receptor signaling pathway	2	0.052233
GO:0010718	positive regulation of epithelial to mesenchymal transition	2	0.052233
GO:0030501	positive regulation of bone mineralization	2	0.052233

PValue	Genes	Fold Enrichment	Bonferroni
6.36E-07	SOCS3, IF	3.33E-04	3.33E-04
6.90E-06	JUN, SMA	0.003601471	0.001802
1.69E-05	SOCS3, JU	0.008809488	0.002945
2.94E-05	OSM, SP1	0.015256142	0.003836
5.03E-05	OSM, SOC	0.025956664	0.005246
5.23E-05	SP1, JUN,	0.026976857	0.004548
8.05E-05	JUN, SMA	0.041245041	0.005999
1.07E-04	JUN, JAK	0.054522786	0.006984
1.86E-04	PRLR, JAI	0.092895714	0.010775
8.68E-04	SOCS3, JA	0.365069139	0.044408
9.23E-04	JUN, IFNC	0.383166291	0.042973
0.00122433	OSM, SOC	0.473085489	0.051993
0.001494516	IFNG, SM	0.542608901	0.058396
0.001566115	IFNG, SM	0.559445157	0.05687
0.002015518	SMAD4, S	0.65187019	0.067928
0.00252569	YY1, IFNC	0.733560182	0.079339
0.002928239	JUN, SRC,	0.784267425	0.086269
0.003126696	STAM, SH	0.805597318	0.086973
0.003129098	PRLR, SO	0.805842183	0.082651
0.004103718	FCER2, IF	0.88359274	0.101953
0.004103718	OSM, JAK	0.88359274	0.101953
0.004103718	PRLR, JAI	0.88359274	0.101953
0.005468041	OSM, JAK	0.943166687	0.127641
0.005468041	SMAD4, S	0.943166687	0.127641
0.005468041	SMAD4, S	0.943166687	0.127641
0.005576061	OSM, JAK	0.946305292	0.124473
0.005976809	PRLR, F2,	0.956511111	0.127432
0.00795637	JUN, IFNC	0.984668163	0.159767
0.008191325	OSM, JAK	0.98645456	0.158078
0.010907475	SMAD3, J	0.996772182	0.197973
0.010907475	SMAD4, S	0.996772182	0.197973
0.012262881	SMAD5, S	0.998424425	0.212589
0.012366128	JUN, SRC,	0.998508252	0.207388
0.013162071	OSM, SMA	0.999021478	0.212543
0.013727937	SOCS3, SO	0.999275079	0.214144
0.014968364	JUN, JUN	0.999624644	0.224649
0.016242872	OSM, JUN	0.999809293	0.234824
0.017666758	FCER2, JA	0.999910593	0.246099
0.017666758	SMAD3, J	0.999910593	0.246099
0.020358081	IFNG, STA	0.999978707	0.264605
0.02598033	F2, JAK2,	0.999998951	0.303925
0.027055566	JUN, SMA	0.999999411	0.307759
0.027055566	SMAD4, S	0.999999411	0.307759
0.028389797	SMAD4, S	0.999999713	0.313787
0.028389797	F2, SRC	0.999999713	0.313787
0.029708337	CEBPD, JU	0.999999859	0.319349

0.03900082	JAK2, SR[illegible]	0.999999999	0.363837
0.044264676	SMAD4, S[illegible]	1	0.389392
0.04688625	ISG15, SM[illegible]	1	0.401034
