# Supplementary material for: Research on the Mechanism of Liuwei Dihuang Decoction for Osteoporosis Based on Systematic Biological Strategies
Source: Evid Based Complement Alternat Med. 2022 Sep 22;2022:7017610. doi: 10.1155/2022/7017610 (PMC9522519; doi:10.1155/2022/7017610)
Supplement: Supplementary Materials — Table S1-1: components meeting the screening criteria. Table S1-2: compound targets for each compound of LDD. Table S2: osteoporosis genes. Table S3: enrichment analysis of clusters based on Gene Ontology (GO) annotation of LDD-osteoporosis PPI network. Table S4: pathway enrichment analysis of LDD-osteoporosis PPI network. Table S5: Reactome pathways of LDD-osteoporosis PPI network. Table S6: Human Transcriptomics Data. Table S7: the biological processes of Human Transcriptomics Data Network. Table S8: the Reactome pathways of Human Transcriptomics Data Network. Table S9: the signaling pathways of Human Transcriptomics Data Network. Table S10: the biological processes of protein arrays data network. Table S11: the Reactome pathways of protein arrays data network. Table S12: the signaling pathways of protein arrays data network. [file 7017610.f1.zip › 7017610.f1/Table S11.pdf]

Table S11 The Reactome Pathways of Protein Arrays Data Network

Pathway identifier	Pathway name	Entities ratio	PValue	FDR
R-HSA-1280215	Cytokine Signaling in Imm	0.08785548	1.55E-15	5.95E-13
R-HSA-449147	Signaling by Interleukins	0.05899372	4.04E-12	7.72E-10
R-HSA-6785807	Interleukin-4 and Interleuk	0.014889563	1.67E-11	2.12E-09
R-HSA-168256	Immune System	0.197798321	1.35E-09	1.29E-07
R-HSA-982772	Growth hormone receptor s	0.002046433	2.53E-08	1.92E-06
R-HSA-8939242	RUNX1 regulates transcrip	7.76E-04	3.59E-08	2.26E-06
R-HSA-2173796	SMAD2/SMAD3:SMAD4	0.002752099	1.09E-07	5.12E-06
R-HSA-877312	Regulation of IFNG signali	0.001129066	1.59E-07	6.68E-06
R-HSA-1170546	Prolactin receptor signaling	0.0012702	2.54E-07	9.65E-06
R-HSA-2173793	Transcriptional activity of	0.003881166	5.87E-07	1.82E-05
R-HSA-9006936	Signaling by TGF-beta fam	0.008044598	9.57E-07	2.78E-05
R-HSA-170834	Signaling by TGF-beta Rec	0.005998165	4.86E-06	1.31E-04
R-HSA-2586552	Signaling by Leptin	9.17E-04	8.02E-06	2.00E-04
R-HSA-8878171	Transcriptional regulation l	0.018417896	9.33E-06	2.14E-04
R-HSA-162582	Signal Transduction	0.22666008	1.59E-05	3.51E-04
R-HSA-76009	Platelet Aggregation (Plug	0.003740032	1.77E-05	3.73E-04
R-HSA-1181150	Signaling by NODAL	0.001623033	4.35E-05	7.40E-04
R-HSA-212436	Generic Transcription Path	0.107543575	5.73E-05	9.17E-04
R-HSA-8854691	Interleukin-20 family signa	0.002046433	8.62E-05	0.001293
R-HSA-157118	Signaling by NOTCH	0.018135629	9.27E-05	0.001333
R-HSA-6783589	Interleukin-6 family signali	0.002117	9.52E-05	0.001333
R-HSA-8952158	RUNX3 regulates BCL2L1	4.23E-04	1.45E-04	0.001928
R-HSA-73857	RNA Polymerase II Transc	0.117352339	1.48E-04	0.001928
R-HSA-8849474	PTK6 Activates STAT3	4.94E-04	1.98E-04	0.00226
R-HSA-354192	Integrin alphaIIb beta3 sigr	0.002752099	2.05E-04	0.00226
R-HSA-9006921	Integrin signaling	0.002752099	2.05E-04	0.00226
R-HSA-8941855	RUNX3 regulates CDKN1	5.65E-04	2.58E-04	0.002833
R-HSA-74160	Gene expression (Transcrip	0.12850187	3.87E-04	0.003873
R-HSA-1433557	Signaling by SCF-KIT	0.003528333	4.24E-04	0.004235
R-HSA-177929	Signaling by EGFR	0.004163432	6.83E-04	0.006834
R-HSA-913531	Interferon Signaling	0.027662127	8.77E-04	0.007889
R-HSA-1502540	Signaling by Activin	0.0010585	8.94E-04	0.008045
R-HSA-2197563	NOTCH2 intracellular dom	0.001129066	0.00101513	0.008932
R-HSA-8848021	Signaling by PTK6	0.004939666	0.001116556	0.008932
R-HSA-9006927	Signaling by Non-Receptor	0.004939666	0.001116556	0.008932
R-HSA-1059683	Interleukin-6 signaling	0.001199633	0.001143893	0.009151
R-HSA-6788467	IL-6-type cytokine receptor	0.001199633	0.001143893	0.009151
R-HSA-8877330	RUNX1 and FOXP3 contro	0.001199633	0.001143893	0.009151
R-HSA-1433559	Regulation of KIT signalin	0.0012702	0.001280083	0.010241
R-HSA-9020591	Interleukin-12 signaling	0.005927599	0.001876171	0.013133
R-HSA-1266738	Developmental Biology	0.08510338	0.001897962	0.013286
R-HSA-372708	p130Cas linkage to MAPK	0.001552466	0.00189829	0.013288
R-HSA-6806834	Signaling by MET	0.006139299	0.002072003	0.014504
R-HSA-447115	Interleukin-12 family signa	0.006774398	0.002734288	0.016999
R-HSA-2173795	Downregulation of SMAD2	0.0019053	0.002833205	0.016999
R-HSA-9617828	FOXO-mediated transcript	0.0019053	0.002833205	0.016999

R-HSA-201451	Signaling by BMP	0.002117	0.003478682	0.020872
R-HSA-9006934	Signaling by Receptor Tyro	0.038176558	0.004410918	0.0243
R-HSA-166520	Signaling by NTRKs	0.008115165	0.004525571	0.0243
R-HSA-5619507	Activation of HOX genes d	0.008185731	0.004635417	0.0243
R-HSA-456926	Thrombin signalling throug	0.002469833	0.004691885	0.0243
R-HSA-8878159	Transcriptional regulation	0.008326865	0.00485994	0.0243
R-HSA-2173789	TGF-beta receptor signalin	0.002540399	0.004954781	0.024774
R-HSA-186763	Downstream signal transdu	0.002540399	0.004954781	0.024774
R-HSA-5673000	RAF activation	0.002540399	0.004954781	0.024774
R-HSA-9013508	NOTCH3 Intracellular Dor	0.002540399	0.004954781	0.024774
R-HSA-1980145	Signaling by NOTCH2	0.002681533	0.005500518	0.027503
R-HSA-187687	Signalling to ERKs	0.002752099	0.005783281	0.028916
R-HSA-877300	Interferon gamma signaling	0.017641663	0.005831716	0.029159
R-HSA-8939211	ESR-mediated signaling	0.017994496	0.006247446	0.031237
R-HSA-8941326	RUNX2 regulates bone dev	0.003034366	0.006979401	0.034472
R-HSA-8875878	MET promotes cell motility	0.003175499	0.00761597	0.034472
R-HSA-452723	Transcriptional regulation	0.003175499	0.00761597	0.034472
R-HSA-9615017	FOXO-mediated transcript	0.003457766	0.008964622	0.035858
R-HSA-512988	Interleukin-3, Interleukin-5	0.003528333	0.009317301	0.037269
R-HSA-9018519	Estrogen-dependent gene e	0.010867264	0.010056866	0.040227
R-HSA-76002	Platelet activation, signalin	0.020676029	0.010066704	0.040267
R-HSA-6802955	Paradoxical activation of R	0.003740032	0.010411986	0.041648

Genes

JUN;SMAD3;CEBPD;SRC;OSM;ISG15;PRL;PRLR;PIAS1;SOCS3;FCER2;IFNG;JAK2;JUNB;CRK;[illegible]
SOCS3;FCER2;JUN;SMAD3;IFNG;CEBPD;SRC;OSM;JAK2;JUNB;CRK
FCER2;SOCS3;CEBPD;OSM;JAK2;JUNB
JUN;SMAD3;CEBPD;SRC;OSM;ISG15;PRL;F2;PRLR;PIAS1;SOCS3;FCER2;IFNG;JAK2;JUNB;C[illegible]
SOCS3;PRL;JAK2;PRLR;SH2B1
SOCS3;SOCS4
SMAD4;SMAD3;SP1;JUNB
SOCS3;IFNG;JAK2;PIAS1
PRL;JAK2;PRLR;SH2B1
SMAD4;SMAD3;SP1;JUNB
SMAD4;SMAD3;SP1;JUNB;SMAD5
SMAD4;SMAD3;SP1;JUNB
SOCS3;JAK2;SH2B1
SOCS3;IFNG;SRC;SOCS4
JUN;SMAD4;SMAD3;SRC;STAM;PRL;F2;SMAD5;YY1;SOCS3;FCER2;SP1;JAK2;JUNB;CRK;SO[illegible]
SRC;F2;CRK
SMAD4;SMAD3;SP1
YY1;SOCS3;JUN;SMAD4;SMAD3;IFNG;SP1;SRC;JUNB;SOCS4
SOCS3;JAK2
FCER2;JUN;SMAD3;PRL
SOCS3;OSM;JAK2
SMAD4;SMAD3
YY1;SOCS3;JUN;SMAD4;SMAD3;IFNG;SP1;SRC;JUNB;SOCS4
SOCS3
SRC;CRK
SRC;CRK
SMAD4;SMAD3
YY1;SOCS3;JUN;SMAD4;SMAD3;IFNG;SP1;SRC;JUNB;SOCS4
SRC;JAK2;SOCS4
SRC;STAM
SOCS3;IFNG;ISG15;JAK2;PIAS1
SMAD4;SMAD3
FCER2
SOCS3;CRK
SOCS3;CRK
SOCS3;JAK2
OSM;JAK2
IFNG
SRC;SOCS4
IFNG;JAK2
YY1;SMAD4;JUN;SMAD3;CEBPD;SP1;SRC;PRL
SRC;CRK
SRC;STAM;CRK
IFNG;JAK2
SMAD4;SMAD3
SMAD4;SMAD3

SMAD4;SMAD5
SRC;STAM;JAK2;CRK;SOCS4
SRC;CRK
YY1;JUN;PRL
SRC;F2
SMAD4;SMAD3;SRC
SMAD4;SMAD3
SRC;CRK
SRC;JAK2
PRL
FCER2
SRC;CRK
SOCS3;IFNG;JAK2;PIAS1
YY1;JUN;SP1;SRC
SMAD4;SRC
SRC;CRK
SMAD4;PRL
SMAD4;SMAD3
JAK2;CRK
YY1;JUN;SP1
SRC;F2;CRK
SRC;JAK2

CS4;SH2B1
