# Supplementary material for: Research on the Mechanism of Liuwei Dihuang Decoction for Osteoporosis Based on Systematic Biological Strategies
Source: Evid Based Complement Alternat Med. 2022 Sep 22;2022:7017610. doi: 10.1155/2022/7017610 (PMC9522519; doi:10.1155/2022/7017610)
Supplement: Supplementary Materials — Table S1-1: components meeting the screening criteria. Table S1-2: compound targets for each compound of LDD. Table S2: osteoporosis genes. Table S3: enrichment analysis of clusters based on Gene Ontology (GO) annotation of LDD-osteoporosis PPI network. Table S4: pathway enrichment analysis of LDD-osteoporosis PPI network. Table S5: Reactome pathways of LDD-osteoporosis PPI network. Table S6: Human Transcriptomics Data. Table S7: the biological processes of Human Transcriptomics Data Network. Table S8: the Reactome pathways of Human Transcriptomics Data Network. Table S9: the signaling pathways of Human Transcriptomics Data Network. Table S10: the biological processes of protein arrays data network. Table S11: the Reactome pathways of protein arrays data network. Table S12: the signaling pathways of protein arrays data network. [file 7017610.f1.zip › 7017610.f1/Table S12.pdf]

Table S12 The Signaling Pathways of Protein Arrays Data Network

Term	Signaling pathways	Count	%	PValue	Genes	Fold Enri
hsa04630	Jak-STAT signaling pathway	9	0.235048	5.19E-09	OSM, PRI	19.40784
hsa04917	Prolactin signaling pathway	6	0.156699	1.82E-06	PRLR, SO	26.42382
hsa04350	TGF-beta signaling pathway	5	0.130582	1.06E-04	SP1, SMA	18.61201
hsa04380	Osteoclast differentiation	4	0.104466	0.006985	SOCS3, JU	9.547536
hsa04550	Signaling pathways regulating pluripoten	4	0.104466	0.008389	SMAD5, S	8.933766
hsa04012	ErbB signaling pathway	3	0.078349	0.028416	JUN, CRK	10.78213
hsa04915	Estrogen signaling pathway	3	0.078349	0.03605	SP1, JUN,	9.475207
hsa04060	Cytokine-cytokine receptor interaction	4	0.104466	0.036194	OSM, PRI	5.147026
hsa04668	TNF signaling pathway	3	0.078349	0.041535	SOCS3, JU	8.76678

Bonferroni
4.98E-07
1.74E-04
0.010097
0.489771
0.554584
0.937175
0.97054
0.970961
0.982965
