# Supplementary material for: Research on the Mechanism of Liuwei Dihuang Decoction for Osteoporosis Based on Systematic Biological Strategies
Source: Evid Based Complement Alternat Med. 2022 Sep 22;2022:7017610. doi: 10.1155/2022/7017610 (PMC9522519; doi:10.1155/2022/7017610)
Supplement: Supplementary Materials — Table S1-1: components meeting the screening criteria. Table S1-2: compound targets for each compound of LDD. Table S2: osteoporosis genes. Table S3: enrichment analysis of clusters based on Gene Ontology (GO) annotation of LDD-osteoporosis PPI network. Table S4: pathway enrichment analysis of LDD-osteoporosis PPI network. Table S5: Reactome pathways of LDD-osteoporosis PPI network. Table S6: Human Transcriptomics Data. Table S7: the biological processes of Human Transcriptomics Data Network. Table S8: the Reactome pathways of Human Transcriptomics Data Network. Table S9: the signaling pathways of Human Transcriptomics Data Network. Table S10: the biological processes of protein arrays data network. Table S11: the Reactome pathways of protein arrays data network. Table S12: the signaling pathways of protein arrays data network. [file 7017610.f1.zip › 7017610.f1/Table S2.pdf]

Table S2 Osteoporosis Genes

Gene Symbol	Description	Relevance score
LRP5	LDL Receptor Related Protein 5	73.38
SLC34A1	Solute Carrier Family 34 Membe	54.37
COL1A1	Collagen Type I Alpha 1 Chain	53.6
ESR1	Estrogen Receptor 1	47.71
IGF1	Insulin Like Growth Factor 1	45.59
TNFRSF11B	TNF Receptor Superfamily Men	45.41
CALCA	Calcitonin Related Polypeptide A	45.4
WNT1	Wnt Family Member 1	41.82
VDR	Vitamin D Receptor	41.55
RUNX2	Runt Related Transcription Fact	39.32
BGLAP	Bone Gamma-Carboxyglutamate	39.1
SLC9A3R1	SLC9A3 Regulator 1	39.03
COL1A2	Collagen Type I Alpha 2 Chain	38.15
PLS3	Plastin 3	37.03
CALCR	Calcitonin Receptor	37
CYP19A1	Cytochrome P450 Family 19 Sul	36.89
DKK1	Dickkopf WNT Signaling Pathw	35.59
TNFRSF11A	TNF Receptor Superfamily Men	32.56
MMP14	Matrix Metallopeptidase 14	30.65
WRN	Werner Syndrome RecQ Like H	30.05
PRKACA	Protein Kinase CAMP-Activated	29.66
AR	Androgen Receptor	29.43
TNFSF11	TNF Superfamily Member 11	29.28
PRKAR1A	Protein Kinase CAMP-Depender	29.25
ALB	Albumin	29.18
NOTCH2	Notch 2	28.86
SP7	Sp7 Transcription Factor	28.41
FSHR	Follicle Stimulating Hormone R	28.36
SMS	Spermine Synthase	28.02
BMP2	Bone Morphogenetic Protein 2	27.81
COL2A1	Collagen Type II Alpha 1 Chain	27.64
SPARC	Secreted Protein Acidic And Cy	26.91
GORAB	Golgin, RAB6 Interacting	26.42
XYLT2	Xylosyltransferase 2	26.3
SMAD3	SMAD Family Member 3	26.14
SLC7A7	Solute Carrier Family 7 Member	25.9
PLOD1	Procollagen-Lysine,2-Oxoglutar	25.52
PDLIM4	PDZ And LIM Domain 4	25.14
ADCY10	Adenylate Cyclase 10	25.14
CD36	CD36 Molecule	24.9
MIR2861	MicroRNA 2861	24.42
IL6	Interleukin 6	24.4
UGT2B17	UDP Glucuronosyltransferase Fa	24.4
SATB2	SATB Homeobox 2	24.31
LRP6	LDL Receptor Related Protein 6	23.82
CYP24A1	Cytochrome P450 Family 24 Sul	23.31

TGFB1	Transforming Growth Factor Be	21.78
WNT3A	Wnt Family Member 3A	21.39
PTH	Parathyroid Hormone	21.26
LMNA	Lamin A/C	21.19
BMND2	Bone Mineral Density Variation	21.06
GNRH1	Gonadotropin Releasing Hormoı	20.42
BMND8	Bone Mineral Density Quantiati	20.39
BMND3	Bone Mineral Density Variabilit	19.9
BMND4	Bone Mineral Density Variation	19.78
BMND5	Bone Mineral Density Variation	19.78
GNAS	GNAS Complex Locus	19.72
BMND10	Bone Mineral Density QTL 10	19.62
BMND11	Bone Mineral Density QTL 11	19.62
BMND13	Bone Mineral Density QTL 13	19.62
BMND14	Bone Mineral Density QTL 14	19.62
BMND6	Bone Mineral Density QTL 6	19.62
BMND7	Bone Mineral Density Quantiati	19.62
BMND9	Bone Mineral Density QTL 9	19.62
TNF	Tumor Necrosis Factor	19.51
SOST	Sclerostin	19.32
SHBG	Sex Hormone Binding Globulin	18.55
SLPI	Secretory Leukocyte Peptidase I	18.54
GC	GC, Vitamin D Binding Protein	18.46
TERT	Telomerase Reverse Transcripta	18.37
PTH1R	Parathyroid Hormone 1 Recepto	18.11
CYP27B1	Cytochrome P450 Family 27 Sul	17.71
CTSK	Cathepsin K	17.7
MEN1	Menin 1	17.57
IL1B	Interleukin 1 Beta	17.45
SPP1	Secreted Phosphoprotein 1	17.44
APOE	Apolipoprotein E	17.36
IGFBP3	Insulin Like Growth Factor Binc	17.26
IL1A	Interleukin 1 Alpha	16.69
ACP5	Acid Phosphatase 5, Tartrate Re	16.6
ALPP	Alkaline Phosphatase, Placental	16.51
PAX5	Paired Box 5	16.25
BMP1	Bone Morphogenetic Protein 1	15.66
CASR	Calcium Sensing Receptor	15.61
NR3C1	Nuclear Receptor Subfamily 3 C	15.58
LEP	Leptin	15.58
IFNG	Interferon Gamma	15.49
CA2	Carbonic Anhydrase 2	15.39
CDX2	Caudal Type Homeobox 2	15.39
ESR2	Estrogen Receptor 2	15.38
PDE11A	Phosphodiesterase 11A	15.32
GH1	Growth Hormone 1	15.27
MMP2	Matrix Metallopeptidase 2	15.25
CTNNB1	Catenin Beta 1	15.03

CDC73	Cell Division Cycle 73	14.99
ALPL	Alkaline Phosphatase, Liver/Bor	14.92
HMGCR	3-Hydroxy-3-Methylglutaryl-Co	14.75
NR5A1	Nuclear Receptor Subfamily 5 G	14.73
CYP3A4	Cytochrome P450 Family 3 Subf	14.71
ARHGEF3	Rho Guanine Nucleotide Exchar	14.58
NHP2	NHP2 Ribonucleoprotein	14.55
FOS	Fos Proto-Oncogene, AP-1 Tran	14.5
FKBP10	FK506 Binding Protein 10	14.49
TGFBR1	Transforming Growth Factor Be	14.49
KL	Klotho	14.48
INS	Insulin	14.4
CYP11A1	Cytochrome P450 Family 11 Sul	14.25
CBS	Cystathionine-Beta-Synthase	14.12
PTHLH	Parathyroid Hormone Like Horn	14.08
CSF1	Colony Stimulating Factor 1	13.92
CA8	Carbonic Anhydrase 8	13.8
SP1	Sp1 Transcription Factor	13.74
PTK2B	Protein Tyrosine Kinase 2 Beta	13.73
NOP10	NOP10 Ribonucleoprotein	13.71
FGF23	Fibroblast Growth Factor 23	13.67
THSD7A	Thrombospondin Type 1 Domai	13.63
IBSP	Integrin Binding Sialoprotein	13.6
ELN	Elastin	13.59
TINF2	TERF1 Interacting Nuclear Fact	13.56
ARMC5	Armadillo Repeat Containing 5	13.56
FDPS	Farnesyl Diphosphate Synthase	13.54
LEPR	Leptin Receptor	13.53
BMP15	Bone Morphogenetic Protein 15	13.4
IL1RN	Interleukin 1 Receptor Antagoni	13.37
IGFBP4	Insulin Like Growth Factor Binc	13.35
CNR2	Cannabinoid Receptor 2	13.3
JUN	Jun Proto-Oncogene, AP-1 Tran	13.26
CYP17A1	Cytochrome P450 Family 17 Sul	13.19
EIF2AK3	Eukaryotic Translation Initiation	13.18
PPARG	Peroxisome Proliferator Activate	13.17
SFRP1	Secreted Frizzled Related Protei	13.11
IL11	Interleukin 11	13.09
AHSG	Alpha 2-HS Glycoprotein	13.09
CRP	C-Reactive Protein	13.07
LOX	Lysyl Oxidase	12.96
GHR	Growth Hormone Receptor	12.77
HFE	Homeostatic Iron Regulator	12.7
IGFBP5	Insulin Like Growth Factor Binc	12.64
HSD11B1	Hydroxysteroid 11-Beta Dehydr	12.62
RTEL1	Regulator Of Telomere Elongati	12.53
P3H1	Prolyl 3-Hydroxylase 1	12.53
SRC	SRC Proto-Oncogene, Non-Rece	12.51

IAPP	Islet Amyloid Polypeptide	12.44
MEPE	Matrix Extracellular Phosphogly	12.4
BMP4	Bone Morphogenetic Protein 4	12.36
MGP	Matrix Gla Protein	12.23
WNT16	Wnt Family Member 16	12.22
SLC26A2	Solute Carrier Family 26 Membe	12.21
MAPK3	Mitogen-Activated Protein Kina	12.17
IL7	Interleukin 7	12.1
WT1	Wilms Tumor 1	12.09
GALT	Galactose-1-Phosphate Uridylylt	12.09
SRY	Sex Determining Region Y	12.09
POF1B	POF1B, Actin Binding Protein	12.09
CRTAP	Cartilage Associated Protein	12
ANTXR2	ANTXR Cell Adhesion Molecul	11.97
TRAF6	TNF Receptor Associated Facto	11.91
TGFB2	Transforming Growth Factor Be	11.91
PDE8B	Phosphodiesterase 8B	11.8
NBN	Nibrin	11.77
MMP3	Matrix Metallopeptidase 3	11.69
IL1RAPL2	Interleukin 1 Receptor Accessor	11.69
CNR1	Cannabinoid Receptor 1	11.64
NOG	Noggin	11.64
TGM2	Transglutaminase 2	11.64
BANF1	Barrier To Autointegration Facto	11.58
DANCR	Differentiation Antagonizing No	11.54
ATP7A	ATPase Copper Transporting Al	11.49
PLOD2	Procollagen-Lysine,2-Oxoglutar	11.45
B3GAT3	Beta-1,3-Glucuronyltransferase 3	11.4
CHST3	Carbohydrate Sulfotransferase 3	11.4
TRPV1	Transient Receptor Potential Ca	11.25
CDH23	Cadherin Related 23	11.21
TGFBR2	Transforming Growth Factor Be	11.19
NFATC1	Nuclear Factor Of Activated T C	10.82
PROP1	PROP Paired-Like Homeobox 1	10.62
FLNA	Filamin A	10.48
CYP21A2	Cytochrome P450 Family 21 Sul	10.4
STAR	Steroidogenic Acute Regulatory	10.38
USP8	Ubiquitin Specific Peptidase 8	10.33
PARN	Poly(A)-Specific Ribonuclease	10.33
WRAP53	WD Repeat Containing Antisens	10.33
ATP7B	ATPase Copper Transporting Be	10.33
NR0B1	Nuclear Receptor Subfamily 0 G	10.33
IFIH1	Interferon Induced With Helicas	10.18
RECQL4	RecQ Like Helicase 4	10.16
GCM2	Glial Cells Missing Homolog 2	10.16
FGFR1	Fibroblast Growth Factor Recep	10.12
SOX9	SRY-Box 9	10.1
STAT1	Signal Transducer And Activato	10.1

ZBTB20	Zinc Finger And BTB Domain C	9.92
PRL	Prolactin	9.89
TERC	Telomerase RNA Component	9.89
LIFR	LIF Receptor Alpha	9.74
SOX3	SRY-Box 3	9.67
DUSP6	Dual Specificity Phosphatase 6	9.67
ZMPSTE24	Zinc Metallopeptidase STE24	9.67
COL7A1	Collagen Type VII Alpha 1 Chai	9.51
MMP1	Matrix Metallopeptidase 1	9.51
FGF17	Fibroblast Growth Factor 17	9.51
PROK2	Prokineticin 2	9.51
DKC1	Dyskerin Pseudouridine Synthas	9.5
HAMP	Hepcidin Antimicrobial Peptide	9.5
FMR1	Fragile X Mental Retardation 1	9.33
HBB	Hemoglobin Subunit Beta	9.28
HSD3B2	Hydroxy-Delta-5-Steroid Dehyd	9.28
CYP11B1	Cytochrome P450 Family 11 Sul	9.28
POU1F1	POU Class 1 Homeobox 1	9.18
HESX1	HESX Homeobox 1	9.18
LHX4	LIM Homeobox 4	9.18
MTHFR	Methylenetetrahydrofolate Redu	8.9
ACD	ACD, Shelterin Complex Subun	8.8
EIF2B2	Eukaryotic Translation Initiation	8.78
MCM9	Minichromosome Maintenance 9	8.78
SGO2	Shugoshin 2	8.78
HPGD	15-Hydroxyprostaglandin Dehyd	8.76
NDP	NDP, Norrin Cystine Knot Grow	8.74
LDLR	Low Density Lipoprotein Recep	8.7
POMC	Proopiomelanocortin	8.63
KIT	KIT Proto-Oncogene Receptor T	8.6
CYP27A1	Cytochrome P450 Family 27 Sul	8.45
FZD4	Frizzled Class Receptor 4	8.38
AAAS	Aladin WD Repeat Nucleoporin	8.34
MIR3615	MicroRNA 3615	8.33
LOC105371893	Uncharacterized LOC10537189	8.33
TENT5A	Terminal Nucleotidyltransferase	8.3
LRP8	LDL Receptor Related Protein 8	8.29
FBN1	Fibrillin 1	8.27
TCF4	Transcription Factor 4	8.26
PYCR1	Pyrroline-5-Carboxylate Reducta	8.26
FLRT3	Fibronectin Leucine Rich Transm	8.26
MST1	Macrophage Stimulating 1	8.26
GPR35	G Protein-Coupled Receptor 35	8.26
IL17RD	Interleukin 17 Receptor D	8.26
SPRY4	Sprouty RTK Signaling Antagon	8.26
HS6ST1	Heparan Sulfate 6-O-Sulfotransf	8.26
NSMF	NMDA Receptor Synaptonuclea	8.26
CTC1	CST Telomere Replication Com	8.26

WISP3	WNT1 Inducible Signaling Path	8.26
HLA-DQA1	Major Histocompatibility Comp	8.17
IGF2	Insulin Like Growth Factor 2	8.09
PPIB	Peptidylprolyl Isomerase B	8.07
POR	Cytochrome P450 Oxidoreducta	8.03
SMPD1	Sphingomyelin Phosphodiestera	8.03
MALT1	MALT1 Paracaspase	8.03
GK	Glycerol Kinase	8.03
FBLN5	Fibulin 5	8.03
GALNS	Galactosamine (N-Acetyl)-6-Sul	8.03
IL12RB1	Interleukin 12 Receptor Subunit	8.03
ERCC6	ERCC Excision Repair 6, Chron	8.03
G6PC	Glucose-6-Phosphatase Catalytic	8.03
SLC37A4	Solute Carrier Family 37 Membe	8.03
PEX12	Peroxisomal Biogenesis Factor 1	8.03
ASXL2	ASXL Transcriptional Regulator	8.03
TRMT10A	TRNA Methyltransferase 10A	8.03
MMEL1	Membrane Metalloendopeptidas	8.03
USB1	U6 SnRNA Biogenesis Phospho	8.03
HJV	Hemojuvelin BMP Co-Receptor	8.03
SERPINH1	Serpin Family H Member 1	8.03
B4GALT7	Beta-1,4-Galactosyltransferase 7	8.03
ADIPOQ	Adiponectin, C1Q And Collagen	8
CYB5A	Cytochrome B5 Type A	7.85
F12	Coagulation Factor XII	7.69
IGF1R	Insulin Like Growth Factor 1 Re	7.69
GLI2	GLI Family Zinc Finger 2	7.56
OTX2	Orthodenticle Homeobox 2	7.56
NUP107	Nucleoporin 107	7.56
PSMC3IP	PSMC3 Interacting Protein	7.56
SPIDR	Scaffold Protein Involved In DN	7.56
GNRHR	Gonadotropin Releasing Hormo	7.29
TACR3	Tachykinin Receptor 3	7.29
CHD7	Chromodomain Helicase DNA B	7.29
FAT4	FAT Atypical Cadherin 4	7.29
BMP6	Bone Morphogenetic Protein 6	7.17
MLXIPL	MLX Interacting Protein Like	7.13
ZFPM2	Zinc Finger Protein, FOG Famil	7.06
LEPQTL1	Leptin, Serum Levels Of	7.02
HSPG2	Heparan Sulfate Proteoglycan 2	6.96
SERPINF1	Serpin Family F Member 1	6.96
HLA-DQB1	Major Histocompatibility Comp	6.93
FLNB	Filamin B	6.86
TFR2	Transferrin Receptor 2	6.8
SEPSECS	Sep (O-Phosphoserine) TRNA:S	6.8
CAVIN1	Caveolae Associated Protein 1	6.8
IGFBP1	Insulin Like Growth Factor Binc	6.69
PRLR	Prolactin Receptor	6.64

FGF8	Fibroblast Growth Factor 8	6.64
RPL11	Ribosomal Protein L11	6.64
LIMK1	LIM Domain Kinase 1	6.64
PHGDH	Phosphoglycerate Dehydrogenas	6.64
HSD17B4	Hydroxysteroid 17-Beta Dehydr	6.64
PSAT1	Phosphoserine Aminotransferase	6.64
TAC3	Tachykinin 3	6.64
AIP	Aryl Hydrocarbon Receptor Inte	6.64
KISS1R	KISS1 Receptor	6.64
HERC2	HECT And RLD Domain Conta	6.64
RFC2	Replication Factor C Subunit 2	6.64
SNRPN	Small Nuclear Ribonucleoprotei	6.64
PROKR2	Prokineticin Receptor 2	6.64
GTF2I	General Transcription Factor IIi	6.64
KISS1	KiSS-1 Metastasis Suppressor	6.64
BAZ1B	Bromodomain Adjacent To Zinc	6.64
ADAMTS2	ADAM Metallopeptidase With T	6.64
GTF2IRD1	GTF2I Repeat Domain Containi	6.64
NDN	Necdin, MAGE Family Member	6.64
PIGT	Phosphatidylinositol Glycan An	6.64
B3GALT6	Beta-1,3-Galactosyltransferase 6	6.64
MKRN3	Makorin Ring Finger Protein 3	6.64
TBL2	Transducin Beta Like 2	6.64
CLIP2	CAP-Gly Domain Containing Li	6.64
SH3PXD2B	SH3 And PX Domains 2B	6.64
MAGEL2	MAGE Family Member L2	6.64
WDR11	WD Repeat Domain 11	6.64
ANOS1	Anosmin 1	6.64
NPAP1	Nuclear Pore Associated Protein	6.64
RNU4ATAC	RNA, U4atac Small Nuclear (U1	6.64
PWRN1	Prader-Willi Region Non-Protein	6.64
SNORD115-1	Small Nucleolar RNA, C/D Box	6.64
PWAR1	Prader Willi/Angelman Region I	6.64
IPW	Imprinted In Prader-Willi Syndro	6.64
SNORD116-1	Small Nucleolar RNA, C/D Box	6.64
MKRN3-AS1	MKRN3 Antisense RNA 1	6.64
TNFSF15	TNF Superfamily Member 15	6.63
CD40LG	CD40 Ligand	6.62
TF	Transferrin	6.59
GATA4	GATA Binding Protein 4	6.56
TMEM165	Transmembrane Protein 165	6.56
IL10	Interleukin 10	6.46
MAP3K1	Mitogen-Activated Protein Kina	6.4
IRF5	Interferon Regulatory Factor 5	6.4
GLB1	Galactosidase Beta 1	6.4
PCCA	Propionyl-CoA Carboxylase Sub	6.4
AKR1D1	Aldo-Keto Reductase Family 1 M	6.4
ASAH1	N-Acylsphingosine Amidohydro	6.4

LAMB3	Laminin Subunit Beta 3	6.4
WWOX	WW Domain Containing Oxidor	6.4
POLD1	DNA Polymerase Delta 1, Cataly	6.4
LAMC2	Laminin Subunit Gamma 2	6.4
IL12A	Interleukin 12A	6.4
PCCB	Propionyl-CoA Carboxylase Sub	6.4
ABCC9	ATP Binding Cassette Subfamily	6.4
LAMA3	Laminin Subunit Alpha 3	6.4
CANT1	Calcium Activated Nucleotidase	6.4
KCNJ8	Potassium Voltage-Gated Chann	6.4
SLC25A19	Solute Carrier Family 25 Membe	6.4
VAMP7	Vesicle Associated Membrane P	6.4
NGLY1	N-Glycanase 1	6.4
EFEMP2	EGF Containing Fibulin Extrace	6.4
RIN2	Ras And Rab Interactor 2	6.4
HSD3B7	Hydroxy-Delta-5-Steroid Dehyd	6.4
CTDP1	CTD Phosphatase Subunit 1	6.4
VPS53	VPS53, GARP Complex Subuni	6.4
TNPO3	Transportin 3	6.4
IFT122	Intraflagellar Transport 122	6.4
SC5D	Sterol-C5-Desaturase	6.4
PRDM5	PR/SET Domain 5	6.4
RAB3GAP1	RAB3 GTPase Activating Protei	6.4
WDR35	WD Repeat Domain 35	6.4
SPIB	Spi-B Transcription Factor	6.4
POU2AF1	POU Class 2 Associating Factor	6.4
WDR19	WD Repeat Domain 19	6.4
IFT43	Intraflagellar Transport 43	6.4
IFT52	Intraflagellar Transport 52	6.4
ZNF469	Zinc Finger Protein 469	6.4
NSD2	Nuclear Receptor Binding SET I	6.4
DMRT3	Doublesex And Mab-3 Related T	6.4
TCIRG1	T Cell Immune Regulator 1, ATI	6.4
PEPD	Peptidase D	6.34
GHRL	Ghrelin And Obestatin Prepropе	6.28
HLA-A	Major Histocompatibility Compl	6.25
ENPP1	Ectonucleotide Pyrophosphatase	6.15
LCT	Lactase	5.94
PRKACB	Protein Kinase CAMP-Activatec	5.81
AEBP1	AE Binding Protein 1	5.78
FBXW7	F-Box And WD Repeat Domain	5.76
CD79A	CD79a Molecule	5.65
ERCC2	ERCC Excision Repair 2, TFIIH	5.55
GNAI2	G Protein Subunit Alpha I2	5.55
CREB3L1	CAMP Responsive Element Bin	5.55
CELIAC2	Celiac Disease 2	5.55
PSC	Cholangitis, Primary Sclerosing	5.55
CELIAC5	Celiac Disease, Susceptibility To	5.55

HRPT3	Hyperparathyroidism 3	5.55
CELIAC10	Celiac Disease, Susceptibility To	5.55
CELIAC11	Celiac Disease, Susceptibility To	5.55
CELIAC12	Celiac Disease, Susceptibility To	5.55
CELIAC13	Celiac Disease, Susceptibility To	5.55
CELIAC6	Celiac Disease, Susceptibility To	5.55
CELIAC7	Celiac Disease, Susceptibility To	5.55
CELIAC8	Celiac Disease, Susceptibility To	5.55
CELIAC9	Celiac Disease, Susceptibility To	5.55
CALR	Calreticulin	5.48
IL2	Interleukin 2	5.47
CCND1	Cyclin D1	5.43
CP	Ceruloplasmin	5.42
GGT1	Gamma-Glutamyltransferase 1	5.39
THRB	Thyroid Hormone Receptor Beta	5.37
JAG1	Jagged 1	5.33
NOTCH3	Notch 3	5.26
EGF	Epidermal Growth Factor	5.25
GNPTAB	N-Acetylglucosamine-1-Phospha	5.15
IER3IP1	Immediate Early Response 3 Inte	5.15
CGA	Glycoprotein Hormones, Alpha [illegible]	5.11
CRH	Corticotropin Releasing Hormon	5.11
IGFBP2	Insulin Like Growth Factor Binc	5.09
CCDC170	Coiled-Coil Domain Containing	5.09
HP	Haptoglobin	5.08
HELLS	Helicase, Lymphoid Specific	5.08
NR1I2	Nuclear Receptor Subfamily 1 G	5.04
TP53	Tumor Protein P53	5.03
VCP	Valosin Containing Protein	5.02
CLCN5	Chloride Voltage-Gated Channe	5.01
