# Supplementary material for: Research on the Mechanism of Liuwei Dihuang Decoction for Osteoporosis Based on Systematic Biological Strategies
Source: Evid Based Complement Alternat Med. 2022 Sep 22;2022:7017610. doi: 10.1155/2022/7017610 (PMC9522519; doi:10.1155/2022/7017610)
Supplement: Supplementary Materials — Table S1-1: components meeting the screening criteria. Table S1-2: compound targets for each compound of LDD. Table S2: osteoporosis genes. Table S3: enrichment analysis of clusters based on Gene Ontology (GO) annotation of LDD-osteoporosis PPI network. Table S4: pathway enrichment analysis of LDD-osteoporosis PPI network. Table S5: Reactome pathways of LDD-osteoporosis PPI network. Table S6: Human Transcriptomics Data. Table S7: the biological processes of Human Transcriptomics Data Network. Table S8: the Reactome pathways of Human Transcriptomics Data Network. Table S9: the signaling pathways of Human Transcriptomics Data Network. Table S10: the biological processes of protein arrays data network. Table S11: the Reactome pathways of protein arrays data network. Table S12: the signaling pathways of protein arrays data network. [file 7017610.f1.zip › 7017610.f1/Table S3.pdf]

Table S3 Enrichment analysis of clusters based on Gene Ontology (GO) annotation

Cluster	Term	Pathway	Count	%	Pvalue
	GO:0000165	MAPK cascade	11	0.127315	6.40E-09
	GO:0038128	ERBB2 signaling pathway	6	0.069444	1.09E-07
	GO:1904707	positive regulation of vascular smooth	5	0.05787	1.09E-07
	GO:0043066	negative regulation of apoptotic proce	12	0.138889	1.14E-07
	GO:0008284	positive regulation of cell proliferatio	12	0.138889	1.45E-07
	GO:0045429	positive regulation of nitric oxide bios	6	0.069444	2.06E-07
	GO:0000187	activation of MAPK activity	7	0.081019	9.33E-07
	GO:0070374	positive regulation of ERK1 and ERK	8	0.092593	1.07E-06
	GO:0050900	leukocyte migration	7	0.081019	2.01E-06
	GO:0007265	Ras protein signal transduction	6	0.069444	2.43E-06
	GO:0045080	positive regulation of chemokine bios	4	0.046296	3.31E-06
	GO:0014066	regulation of phosphatidylinositol 3-k	6	0.069444	4.16E-06
	GO:0007173	epidermal growth factor receptor sign	5	0.05787	2.67E-05
	GO:0060559	positive regulation of calcidiol 1-mon	3	0.034722	2.82E-05
	GO:0043406	positive regulation of MAP kinase act	5	0.05787	3.28E-05
	GO:0038096	Fc-gamma receptor signaling pathway	6	0.069444	4.47E-05
	GO:0030574	collagen catabolic process	5	0.05787	4.53E-05
	GO:0043627	response to estrogen	5	0.05787	4.81E-05
	GO:0006915	apoptotic process	10	0.115741	5.39E-05
	GO:0070371	ERK1 and ERK2 cascade	4	0.046296	5.41E-05
	GO:0001525	angiogenesis	7	0.081019	6.27E-05
	GO:0048010	vascular endothelial growth factor rec	5	0.05787	7.20E-05
	GO:2000379	positive regulation of reactive oxygen	4	0.046296	1.07E-04
	GO:0007568	aging	6	0.069444	1.54E-04
	GO:0042059	negative regulation of epidermal grow	4	0.046296	1.86E-04
**1**	GO:0033197	response to vitamin E	3	0.034722	5.08E-04
	GO:0034116	positive regulation of heterotypic cell-	3	0.034722	5.08E-04
	GO:0030278	regulation of ossification	3	0.034722	5.08E-04
	GO:0060397	JAK-STAT cascade involved in grow	3	0.034722	9.62E-04
	GO:0014068	positive regulation of phosphatidylino	4	0.046296	0.001068
	GO:0046330	positive regulation of JNK cascade	4	0.046296	0.001068
	GO:0006954	inflammatory response	7	0.081019	0.001077
	GO:0045672	positive regulation of osteoclast differ	3	0.034722	0.001555
	GO:0031295	T cell costimulation	4	0.046296	0.001809
	GO:0042346	positive regulation of NF-kappaB imp	3	0.034722	0.001902
	GO:0001503	ossification	4	0.046296	0.001945
	GO:0043410	positive regulation of MAPK cascade	4	0.046296	0.002016
	GO:0046427	positive regulation of JAK-STAT cas	3	0.034722	0.002088
	GO:0007179	transforming growth factor beta recep	4	0.046296	0.002897
	GO:0071392	cellular response to estradiol stimulus	3	0.034722	0.004129
	GO:0060557	positive regulation of vitamin D biosy	2	0.023148	0.006184
	GO:0051092	positive regulation of NF-kappaB tran	4	0.046296	0.00809
	GO:0000186	activation of MAPKK activity	3	0.034722	0.008923
	GO:0051591	response to cAMP	3	0.034722	0.008923

GO:0000189	MAPK import into nucleus	2	0.023148	0.009262
GO:0007260	tyrosine phosphorylation of STAT pro	2	0.023148	0.012331
GO:0007262	STAT protein import into nucleus	2	0.023148	0.01539
GO:0050727	regulation of inflammatory response	3	0.034722	0.016285
GO:0060070	canonical Wnt signaling pathway	3	0.034722	0.027285
GO:0032355	response to estradiol	3	0.034722	0.032325
GO:0043122	regulation of I-kappaB kinase/NF-kap	2	0.023148	0.045476

GO:0043066	negative regulation of apoptotic proce	21	0.182927	1.07E-15
GO:0001934	positive regulation of protein phospho	12	0.10453	3.75E-12
GO:0030335	positive regulation of cell migration	13	0.11324	1.07E-11
GO:0007173	epidermal growth factor receptor signa	9	0.078397	6.50E-11
GO:0001666	response to hypoxia	12	0.10453	1.03E-10
GO:0014068	positive regulation of phosphatidylino	8	0.069686	8.34E-09
GO:0045429	positive regulation of nitric oxide bios	7	0.060976	2.09E-08
GO:0070374	positive regulation of ERK1 and ERK	10	0.087108	3.91E-08
GO:0043410	positive regulation of MAPK cascade	8	0.069686	3.96E-08
GO:0008284	positive regulation of cell proliferation	14	0.121951	4.60E-08
GO:0051384	response to glucocorticoid	7	0.060976	2.63E-07
GO:0000165	MAPK cascade	10	0.087108	1.20E-06
GO:0051000	positive regulation of nitric-oxide syn	5	0.043554	1.81E-06
GO:0001649	osteoblast differentiation	7	0.060976	4.26E-06
GO:0008285	negative regulation of cell proliferatio	11	0.095819	4.67E-06
GO:0043552	positive regulation of phosphatidylino	5	0.043554	7.55E-06
GO:0032869	cellular response to insulin stimulus	6	0.052265	1.59E-05
GO:0042523	positive regulation of tyrosine phosph	4	0.034843	4.33E-05
GO:0000187	activation of MAPK activity	6	0.052265	7.77E-05
GO:0045453	bone resorption	4	0.034843	9.67E-05
GO:1901203	positive regulation of extracellular ma	3	0.026132	9.93E-05
GO:0043406	positive regulation of MAP kinase act	5	0.043554	1.00E-04
GO:0045669	positive regulation of osteoblast differ	5	0.043554	1.07E-04
GO:0060395	SMAD protein signal transduction	5	0.043554	1.22E-04
GO:0001958	endochondral ossification	4	0.034843	1.61E-04
GO:0051092	positive regulation of NF-kappaB tran	6	0.052265	2.16E-04
GO:0022617	extracellular matrix disassembly	5	0.043554	2.68E-04
GO:0014066	regulation of phosphatidylinositol 3-k	5	0.043554	2.96E-04
GO:0001503	ossification	5	0.043554	3.26E-04
GO:0010718	positive regulation of epithelial to mes	4	0.034843	3.32E-04
GO:0030501	positive regulation of bone mineraliza	4	0.034843	3.96E-04
GO:0043123	positive regulation of I-kappaB kinase	6	0.052265	5.22E-04
GO:0032355	response to estradiol	5	0.043554	5.33E-04
GO:0043124	negative regulation of I-kappaB kinase	4	0.034843	5.88E-04
GO:0010862	positive regulation of pathway-restrict	4	0.034843	0.001006
GO:0071560	cellular response to transforming grov	4	0.034843	0.001069
GO:0060391	positive regulation of SMAD protein i	3	0.026132	0.001261
GO:1902176	negative regulation of oxidative stress	3	0.026132	0.001467

GO:0022408	negative regulation of cell-cell adhesi	3	0.026132	0.001688
GO:0030502	negative regulation of bone mineraliza	3	0.026132	0.001688
GO:0060397	JAK-STAT cascade involved in grow	3	0.026132	0.001688
GO:0070373	negative regulation of ERK1 and ERK	4	0.034843	0.001743
GO:0051592	response to calcium ion	4	0.034843	0.001743
GO:0061036	positive regulation of cartilage develo	3	0.026132	0.001924
GO:0032331	negative regulation of chondrocyte di	3	0.026132	0.002175
GO:0002076	osteoblast development	3	0.026132	0.002175
GO:0043627	response to estrogen	4	0.034843	0.002417
GO:2000249	regulation of actin cytoskeleton reorga	3	0.026132	0.00244
GO:0001938	positive regulation of endothelial cell	4	0.034843	0.002866
GO:0048010	vascular endothelial growth factor rec	4	0.034843	0.003234
GO:0032967	positive regulation of collagen biosyn	3	0.026132	0.003982
GO:0030316	osteoclast differentiation	3	0.026132	0.003982
GO:0070371	ERK1 and ERK2 cascade	3	0.026132	0.004332
GO:0045671	negative regulation of osteoclast diffe	3	0.026132	0.004332
GO:0090090	negative regulation of canonical Wnt s	5	0.043554	0.004533
GO:0002053	positive regulation of mesenchymal ce	3	0.026132	0.005074
GO:0007179	transforming growth factor beta recep	4	0.034843	0.006424
GO:0030282	bone mineralization	3	0.026132	0.008576
GO:0043407	negative regulation of MAP kinase ac	3	0.026132	0.00958
GO:0038128	ERBB2 signaling pathway	3	0.026132	0.010634
GO:0007219	Notch signaling pathway	4	0.034843	0.011813
GO:2000366	positive regulation of STAT protein in	2	0.017422	0.012277
GO:0002548	monocyte chemotaxis	3	0.026132	0.012888
GO:0030155	regulation of cell adhesion	3	0.026132	0.013481
GO:0051591	response to cAMP	3	0.026132	0.01533
GO:0007204	positive regulation of cytosolic calciu	4	0.034843	0.017755
GO:0035630	bone mineralization involved in bone	2	0.017422	0.02038
GO:0007182	common-partner SMAD protein phosp	2	0.017422	0.024406
GO:0007183	SMAD protein complex assembly	2	0.017422	0.032411
GO:0030509	BMP signaling pathway	3	0.026132	0.039024
GO:0033690	positive regulation of osteoblast proli	2	0.017422	0.044296
GO:0033628	regulation of cell adhesion mediated b	2	0.017422	0.044296
GO:0060070	canonical Wnt signaling pathway	3	0.026132	0.045755
GO:0045779	negative regulation of bone resorption	2	0.017422	0.048225
GO:0097067	cellular response to thyroid hormone s	2	0.017422	0.048225
GO:0019430	removal of superoxide radicals	2	0.017422	0.048225
GO:0001501	skeletal system development	14	0.111492	9.30E-14
GO:0008284	positive regulation of cell proliferation	17	0.135383	6.14E-10
GO:0010628	positive regulation of gene expression	13	0.103528	4.41E-09
GO:0007267	cell-cell signaling	12	0.095564	3.64E-08
GO:0045453	bone resorption	6	0.047782	5.69E-08
GO:0006468	protein phosphorylation	13	0.103528	1.80E-06
GO:0006954	inflammatory response	12	0.095564	1.97E-06

	GO:0048762	mesenchymal cell differentiation	4	0.031855	5.95E-06
	GO:0010468	regulation of gene expression	7	0.055746	8.67E-06
	GO:0018108	peptidyl-tyrosine phosphorylation	8	0.063709	8.99E-06
	GO:0001525	angiogenesis	9	0.071673	1.19E-05
	GO:0030198	extracellular matrix organization	8	0.063709	4.45E-05
	GO:0001649	osteoblast differentiation	6	0.047782	1.46E-04
	GO:0001837	epithelial to mesenchymal transition	4	0.031855	5.81E-04
	GO:0030199	collagen fibril organization	4	0.031855	8.72E-04
	GO:0006687	glycosphingolipid metabolic process	4	0.031855	0.001326
	GO:0030514	negative regulation of BMP signaling	4	0.031855	0.001326
	GO:0042340	keratan sulfate catabolic process	3	0.023891	0.00147
	GO:0036092	phosphatidylinositol-3-phosphate bios	4	0.031855	0.001698
	GO:0030574	collagen catabolic process	4	0.031855	0.003646
	GO:0043627	response to estrogen	4	0.031855	0.003809
	GO:1904886	beta-catenin destruction complex disa	3	0.023891	0.004987
	GO:0001503	ossification	4	0.031855	0.006804
	GO:0045840	positive regulation of mitotic nuclear	3	0.023891	0.006929
	GO:0001958	endochondral ossification	3	0.023891	0.006929
	GO:0002053	positive regulation of mesenchymal ce	3	0.023891	0.006929
**3**	GO:0043410	positive regulation of MAPK cascade	4	0.031855	0.007041
	GO:0060349	bone morphogenesis	3	0.023891	0.00746
	GO:0010575	positive regulation of vascular endoth	3	0.023891	0.00746
	GO:0000165	MAPK cascade	6	0.047782	0.008708
	GO:0006874	cellular calcium ion homeostasis	4	0.031855	0.010277
	GO:0046034	ATP metabolic process	3	0.023891	0.010379
	GO:0048705	skeletal system morphogenesis	3	0.023891	0.010379
	GO:0046854	phosphatidylinositol phosphorylation	4	0.031855	0.01058
	GO:0030282	bone mineralization	3	0.023891	0.011666
	GO:0043065	positive regulation of apoptotic proces	6	0.047782	0.014963
	GO:0045668	negative regulation of osteoblast diffe	3	0.023891	0.015172
	GO:0090263	positive regulation of canonical Wnt s	4	0.031855	0.02028
	GO:0050900	leukocyte migration	4	0.031855	0.021175
	GO:0043066	negative regulation of apoptotic proce	7	0.055746	0.022557
	GO:0071560	cellular response to transforming grov	3	0.023891	0.023345
	GO:0010310	regulation of hydrogen peroxide metal	2	0.015927	0.02389
	GO:0060351	cartilage development involved in end	2	0.015927	0.02389
	GO:0007189	adenylate cyclase-activating G-proteir	3	0.023891	0.024243
	GO:0001957	intramembranous ossification	2	0.015927	0.0286
	GO:0031960	response to corticosteroid	2	0.015927	0.0286
	GO:0007050	cell cycle arrest	4	0.031855	0.030747
	GO:0043406	positive regulation of MAP kinase act	3	0.023891	0.032934
	GO:0051216	cartilage development	3	0.023891	0.032934
	GO:0046850	regulation of bone remodeling	2	0.015927	0.033287
	GO:0071374	cellular response to parathyroid horm	2	0.015927	0.033287
	GO:0030512	negative regulation of transforming gr	3	0.023891	0.038213
	GO:0014068	positive regulation of phosphatidylino	3	0.023891	0.039305

	GO:0001501	skeletal system development	6	0.068855	7.71E-05
	GO:0007188	adenylate cyclase-modulating G-prote	4	0.045903	2.26E-04
	GO:0018105	peptidyl-serine phosphorylation	5	0.057379	6.94E-04
	GO:0051289	protein homotetramerization	4	0.045903	9.45E-04
	GO:0000165	MAPK cascade	6	0.068855	0.001509
	GO:0007568	aging	5	0.057379	0.00194
	GO:0001974	blood vessel remodeling	3	0.034427	0.004733
	GO:0048015	phosphatidylinositol-mediated signali	4	0.045903	0.004801
	GO:0001525	angiogenesis	5	0.057379	0.005698
	GO:0042157	lipoprotein metabolic process	3	0.034427	0.006626
	GO:0007186	G-protein coupled receptor signaling p	9	0.103282	0.007627
	GO:0032526	response to retinoic acid	3	0.034427	0.007681
	GO:0036092	phosphatidylinositol-3-phosphate bios	3	0.034427	0.010837
	GO:0090080	positive regulation of MAPKKK casc	2	0.022952	0.015978
	GO:0008284	positive regulation of cell proliferatio	6	0.068855	0.016701
	GO:0030574	collagen catabolic process	3	0.034427	0.018018
**4**	GO:0007584	response to nutrient	3	0.034427	0.023653
	GO:0022617	extracellular matrix disassembly	3	0.034427	0.024856
	GO:0014066	regulation of phosphatidylinositol 3-k	3	0.034427	0.026085
	GO:0001503	ossification	3	0.034427	0.027337
	GO:0042060	wound healing	3	0.034427	0.027337
	GO:0008543	fibroblast growth factor receptor signa	3	0.034427	0.028613
	GO:0008283	cell proliferation	5	0.057379	0.029889
	GO:0008154	actin polymerization or depolymerizat	2	0.022952	0.037927
	GO:0060389	pathway-restricted SMAD protein pho	2	0.022952	0.041023
	GO:0033630	positive regulation of cell adhesion m	2	0.022952	0.041023
	GO:0032516	positive regulation of phosphoprotein	2	0.022952	0.041023
	GO:0032026	response to magnesium ion	2	0.022952	0.041023
	GO:0005513	detection of calcium ion	2	0.022952	0.044109
	GO:0001649	osteoblast differentiation	3	0.034427	0.044136
	GO:0002223	stimulatory C-type lectin receptor sig	3	0.034427	0.044902
	GO:0006955	immune response	5	0.057379	0.046144
	GO:0030502	negative regulation of bone mineraliza	2	0.022952	0.047186
	GO:0002003	angiotensin maturation	3	0.056201	3.15E-04
	GO:0006749	glutathione metabolic process	4	0.074934	3.42E-04
	GO:1904874	positive regulation of telomerase RNA	3	0.056201	5.99E-04
**6**	GO:1901687	glutathione derivative biosynthetic pro	3	0.056201	0.001303
	GO:0055114	oxidation-reduction process	7	0.131135	0.002943
	GO:0008202	steroid metabolic process	3	0.056201	0.00493
	GO:0070458	cellular detoxification of nitrogen com	2	0.037467	0.007307
	GO:0006805	xenobiotic metabolic process	3	0.940439	1.27E-04
**10**	GO:0042738	exogenous drug catabolic process	2	0.626959	0.002856
	GO:0008202	steroid metabolic process	2	0.626959	0.010205

	GO:0043401	steroid hormone mediated signaling pa	2	0.626959	0.01351
	GO:0035426	extracellular matrix-cell signaling	2	0.21164	9.53E-04
**12**	GO:0060070	canonical Wnt signaling pathway	2	0.21164	0.019627
	GO:0016055	Wnt signaling pathway	2	0.21164	0.04381

l

Genes	Fold Enrichment	Bonferroni
EGFR, MAPK1, HRAS, TNF, MAP2K1, GRB2, MAPK3, IL1	13.30203082	8.39E-06
EGFR, HRAS, HSP90AA1, GRB2, EGF, SRC	50.02581927	1.42E-04
MMP9, MDM2, JAK2, MMP2, IL10	105.6100629	1.43E-04
LEP, EGFR, IGF1R, XIAP, MMP9, TP53, MDM2, CAT, BCl	8.35596102	1.49E-04
LEP, EGFR, MAPK1, IGF1R, AR, HRAS, IFNG, MDM2, IG	8.158717305	1.89E-04
EGFR, TNF, HSP90AA1, IFNG, IL1B, JAK2	44.20886354	2.70E-04
MAPK1, TNF, MAP2K1, MAPK14, MAPK3, IL1B, EGF	20.72720861	0.001220991
EGFR, HRAS, TNF, MAP2K1, JUN, MAPK3, CCL5, SRC	14.48366577	0.001396784
HRAS, GRB2, MMP9, LCK, SELE, SRC, MMP1	18.17878132	0.002634357
HRAS, GRB2, MAPK14, JUN, TP53, CCNA2	27.15687332	0.003179047
TNF, HMOX1, IFNG, IL1B	126.7320755	0.004326792
EGFR, MAPK1, GRB2, MAPK3, LCK, EGF	24.37155298	0.005436462
EGFR, HRAS, GRB2, EGF, SRC	28.2884097	0.034320754
TNF, IFNG, IL1B	316.8301887	0.036219613
EGFR, HRAS, TNF, EGF, SRC	26.85001599	0.042069427
CDC42, MAPK1, HSP90AA1, GRB2, MAPK3, SRC	14.96835537	0.056878948
MMP9, MMP7, MMP3, MMP2, MMP1	24.75235849	0.057595858
MAPK1, CCND1, HSP90AA1, HMOX1, PPARG	24.37155298	0.061119337
MAPK1, XIAP, MAPK14, MAPK3, IFNG, TP53, IL1B, JAK	5.587834016	0.068135784
MAPK1, MAP2K1, MAPK3, EGF	52.80503145	0.068474721
LEP, MAPK14, JUN, HMOX1, NOS3, EGF, MMP2	9.945342246	0.078853166
CDC42, HSP90AA1, MAPK14, RHOA, SRC	22.00209644	0.090003549
LEP, GRB2, MAPK14, TP53	42.24402516	0.131018982
FOS, GRB2, JUN, MMP7, CAT, IL10	11.52109777	0.183010273
EGFR, CDC42, GRB2, EGF	35.2033543	0.2163269
LEP, CCND1, CAT	86.40823328	0.486164401
TNF, IL1B, IL10	86.40823328	0.486164401
MAPK1, MAPK14, MAPK3	86.40823328	0.486164401
MAPK1, MAPK3, JAK2	63.36603774	0.71674718
LEP, JAK2, CAT, CCL5	19.49724238	0.753521344
CDC42, HRAS, TNF, IL1B	19.49724238	0.753521344
FOS, TNF, CRP, IL1B, CCL5, SELE, IL10	5.85174491	0.756237328
FOS, TNF, IFNG	50.02581927	0.869801195
CDC42, GRB2, LCK, SRC	16.24770198	0.906707223
TNF, RHOA, IL1B	45.26145553	0.917437165
EGFR, MMP9, IGF2, RUNX2	15.84150943	0.921978178
LEP, AR, HRAS, IGF2	15.64593524	0.928867712
LEP, CCL5, IL10	43.20411664	0.935329151
FOS, JUN, RHOA, SRC	13.77522559	0.977635295
EGFR, CCNA2, IL10	30.660986	0.995576215
TNF, IFNG	316.8301887	0.999704316
AR, TNF, IL1B, CAT	9.528727479	0.999976094
EGFR, JAK2, EGF	20.66283839	0.999992048
FOS, REN, JUN	20.66283839	0.999992048

MAPK1, MAPK3	211.2201258	0.999994917
LEP, JAK2	158.4150943	0.999999913
JAK2, EGF	126.7320755	0.999999998
XIAP, JAK2, SELE	15.08715184	1
CDC42, CCND1, EGF	11.45169357	1
LEP, CCND1, CAT	10.44495128	1
TNF, IL1B	42.24402516	1
BMP4, IL6, ERBB4, RAF1, SMAD3, IGF1, SOX9, KDR, SO	11.07164835	1.85E-12
AKT1, BMP4, PTK2, BMP2, ERBB4, F2, ADAM17, ABL1,	22.6663667	6.24E-09
BMP4, PTK2, BMP2, INS, PTK2B, SMAD3, IGF1, ADAM1	16.9484472	1.78E-08
PTK2, PTK2B, ADAM17, ABL1, CSK, SOX9, TGFB1, PIK3	38.55306122	1.08E-07
BMP2, CASP3, PTK2B, ADAM17, RAF1, SMAD3, NOS2, N	16.73621262	1.71E-07
PTK2, ERBB4, INS, F2, IGF1, KIT, SOX9, KDR	29.5243956	1.39E-05
AKT1, IL6, PTK2B, INS, ESR1, SMAD3, SOD2	39.05116279	3.47E-05
BMP4, BMP2, FGG, IL6, ERBB4, PTK2B, ABL1, TGFB1, K	13.7077551	6.50E-05
BMP2, IL6, INS, IGF1, KIT, IGFBP4, KDR, CTNNB1	23.69241623	6.58E-05
IL6, ERBB4, IGF1, KIT, SOX9, CDK2, TGFB1, KDR, PTK2	7.206866953	7.64E-05
ALPL, BGLAP, APOA2, CASP3, IL6, SPARC, ADIPOQ	25.83384615	4.38E-04
MEN1, PTK2, ERBB4, PTK2B, INS, RAF1, KIT, JAK3, TGI	9.155943293	0.00198716
AKT1, PTK2B, INS, ESR1, TERT	54.51948052	0.002998766
ALPL, AKT1, BMP4, BGLAP, BMP2, SPP1, IGFBP5	16.14615385	0.007067522
MEN1, BMP4, BMP2, IL6, ERBB4, PTK2B, RAF1, CSK, TC	6.663492063	0.007740123
PTK2, ERBB4, PTK2B, KIT, TGFB1	38.69124424	0.012484753
AKT1, SP1, PARP1, STAT1, ADIPOQ, PIK3R1	18.69239332	0.026073108
ERBB4, IGF1, KIT, IL2	56.44369748	0.069522484
BMP2, IGF1, MAPK10, KIT, TRAF6, PTPN11	13.45153538	0.12125254
TNFSF11, PTK2B, TRAF6, CTNNB1	43.61558442	0.1485438
SMAD3, SOX9, TGFB1	179.9142857	0.152250248
TNFSF11, ELANE, KIT, CSK, TGFB1	20.32929782	0.153512461
BMP4, BMP2, IL6, IGF1, CTNNB1	19.99047619	0.163069384
BMP4, BMP2, SMAD3, BMP15, TGFB1	19.34562212	0.183225698
ALPL, BMP4, MMP14, MMP13	36.90549451	0.235339641
IL6, TNFSF11, CD40LG, INS, TRAF6, TGFB1	10.82191192	0.302275566
ELANE, FBN1, MMP14, MMP13, SPP1	15.78195489	0.359708179
AKT1, ERBB4, KIT, PIK3R1, PTPN11	15.37728938	0.388988481
TNFSF11, SPARC, TRAF6, SOX9, SPP1	14.99285714	0.418925084
BMP2, SMAD3, TGFB1, CTNNB1	29.07705628	0.423995629
BMP4, BMP2, SMAD3, TGFB1	27.4155102	0.48205607
TNFSF11, TRAF6, ABL1, CASP1, ADIPOQ, CTNNB1	8.939840284	0.580202063
CASP3, ESR1, NQO1, TGFB1, CTNNB1	13.18053375	0.588074401
ESR1, ABL1, STAT1, ADIPOQ	23.98857143	0.624211926
BMP4, BMP2, BMP15, TGFB1	19.99047619	0.812498476
FBN1, ABL1, SOX9, TGFB1	19.58250729	0.83101996
BMP4, PARP1, TGFB1	55.35824176	0.87725503
AKT1, INS, SOD2	51.40408163	0.912927701

PTK2, ABL1, TGFB1	47.97714286	0.939758976
PTK2B, MEPE, SOX9	47.97714286	0.939758976
PTPN1, JAK3, PRL	47.97714286	0.939758976
PTPN1, ABL1, CSK, ADIPOQ	16.54384236	0.945042024
FGG, IL6, PTK2B, SPARC	16.54384236	0.945042024
BMP4, BMP2, SOX9	44.97857143	0.959344207
BMP4, SOX9, CTNNB1	42.33277311	0.973229831
MEN1, BGLAP, SMAD3	42.33277311	0.973229831
BGLAP, APOA2, ESR1, MMP14	14.7621978	0.982137166
PTK2B, ABL1, TGFB1	39.98095238	0.982798819
AKT1, BMP4, BMP2, KDR	13.90641822	0.991544624
PTK2, PTK2B, PIK3R1, KDR	13.32698413	0.995422581
BMP4, F2, TGFB1	31.28944099	0.998686274
TNFSF11, TRAF6, CTNNB1	31.28944099	0.998686274
TNFSF11, IGF1, SOX9	29.98571429	0.999268338
FBN1, PIK3R1, CTNNB1	29.98571429	0.999268338
BMP2, GSK3B, IGFBP1, SOX9, IGFBP4	7.358457493	0.99947689
STAT1, SOX9, CTNNB1	27.67912088	0.999788293
PTK2, SMAD3, PARP1, TGFB1	10.42981366	0.999977829
BGLAP, BMP2, MMP13	21.16638655	0.999999398
BMP4, PTPN1, ADIPOQ	19.99047619	0.999999888
AKT1, ERBB4, PIK3R1	18.93834586	0.999999981
BMP2, ADAM17, SOX9, TGFB1	8.343850932	0.999999997
IL6, ERBB4	159.9238095	0.999999999
IL6, TNFSF11, LGALS3	17.13469388	1
PTK2B, ABL1, SOX9	16.73621262	1
PTK2B, SPARC, STAT1	15.6447205	1
PTK2B, ESR1, ABL1, IL2	7.160767591	1
BMP2, IGF1	95.95428571	1
BMP4, TGFB1	79.96190476	1
SMAD3, TGFB1	59.97142857	1
BMP4, BMP2, BMP15	9.469172932	1
BMP2, ABL1	43.61558442	1
PTK2, PTPN11	43.61558442	1
GSK3B, SMAD3, CTNNB1	8.670567986	1
IL6, CSK	39.98095238	1
KIT, CTSB	39.98095238	1
NQO1, SOD2	39.98095238	1
PTHLH, VDR, FGFR1, NOG, TNFRSF11B, TGFBR1, HEXI	20.92647321	1.22E-10
FGFR2, FGFR1, PGF, IL7, HCK, TGFBR1, CSF1, WNT3A,	7.470532817	8.06E-07
LCN2, VDR, NOG, LDLR, CSF1, TGFBR1, WNT3A, CRH,	10.1608639	5.79E-06
CALCA, FGFR2, PTHLH, WNT1, GNRH1, PGF, IL7, PTH,	9.674668715	4.78E-05
CTSK, RAC2, IL7, PTH, RAC1, ACP5	55.84922395	7.48E-05
PIK3CG, FGFR1, HCK, TGFBR1, CDK6, AURKA, BTK, CA	5.838040223	0.002365939
PIK3CG, CALCA, SELP, TNFRSF11B, CSF1, HCK, RAC1,	6.483814917	0.002589276

FGFR2, FGFR1, TGFBR1, BMP7	102.3902439	0.007789032
PTHLH, GNRH1, IL7, TGFBR1, PTH, COL2A1, CDK6	14.33463415	0.011333707
FGFR2, FGFR1, HCK, TEK, ZAP70, FGF1, EPHA2, BTK	10.70747649	0.011737852
PIK3CG, FGFR2, FGFR1, PGF, LEPR, TEK, JAG1, FGF1, E	8.264683364	0.015559128
IBSP, TTR, TNFRSF11B, WNT3A, ELN, COL1A2, COL2A1	8.358387257	0.056739458
IBSP, NOG, WNT3A, SP7, COL1A1, EPHA2	11.81425891	0.174487997
FGFR2, NOG, TGFBR1, BMP7	24.09182209	0.533980571
TGFBR1, COL1A2, COL2A1, COL1A1	21.00312695	0.682160015
GM2A, HEXB, ARSA, GLB1	18.20271003	0.825089156
WNT1, NOG, SOST, DKK1	18.20271003	0.825089156
HEXB, GALNS, GLB1	51.19512195	0.855286632
PIK3CG, FGFR2, FGFR1, FGF1	16.71677451	0.892792054
CTSK, COL1A2, COL2A1, COL1A1	12.79878049	0.99176463
TNFRSF11B, TEK, CRH, ARSA	12.60187617	0.993359888
WNT1, WNT3A, LRP5	27.92461197	0.998596623
IBSP, SOST, BMP7, EIF2AK3	10.23902439	0.999872944
AURKA, IL1A, LRP5	23.62851782	0.999892314
TEK, COL2A1, COL1A1	23.62851782	0.999892314
FGFR2, FGFR1, LRP5	23.62851782	0.999892314
FGFR2, FGFR1, TNFRSF11B, IL11	10.11261668	0.999907199
FGFR2, ACP5, LRP5	22.75338753	0.999946676
GATA4, EIF2AK3, IL1A	22.75338753	0.999946676
FGFR2, FGFR1, MAP3K1, TEK, FGF1, DUSP6	4.689629492	0.99998979
VDR, HEXB, PTH, CALR	8.807762916	0.999998727
VCP, HSPA1A, HSPA8	19.19817073	0.999998888
FGFR2, FGFR1, TGFBR1	19.19817073	0.999998888
PIK3CG, FGFR2, FGFR1, FGF1	8.714063311	0.999999148
IBSP, FGFR2, EIF2AK3	18.06886657	0.999999799
RAC1, TGM2, APAF1, BMP7, WT1, DUSP6	4.095609756	0.999999998
NOG, CDK6, LRP5	15.75234522	0.999999998
FGFR2, WNT1, WNT3A, COL1A1	6.82601626	1
SELP, TEK, COL1A2, COL1A1	6.714114354	1
GNRH1, IL7, HCK, TEK, TGM2, AURKA, WT1	3.150469043	1
TGFBR1, APAF1, COL1A1	12.53758089	1
RAC2, RAC1	81.91219512	1
COL2A1, COL1A1	81.91219512	1
CALCA, PTHLH, PTH	12.28682927	1
CTSK, COL1A1	68.2601626	1
GNRH1, COL1A1	68.2601626	1
TGFBR1, RHEB, CDK6, CALR	5.809375541	1
PIK3CG, FGFR1, FGF1	10.41256718	1
NOG, COL2A1, BMP7	10.41256718	1
LEPR, LRP5	58.5087108	1
SOST, PRKACA	58.5087108	1
WNT1, TGFBR1, HSPA1A	9.599085366	1
FGFR1, SELP, TEK	9.451407129	1

CHD7, PTH1R, GLI2, TGFB2, AHSG, BMP6	13.37120106	0.063198257
GNAI2, CNR1, PTH1R, PRKACB	33.00638821	0.174283081
PRKCQ, PDPK1, MAPK12, TGFBR2, SYK	12.21236364	0.444616594
ALDOA, RXRA, CDA, HPRT1	20.35393939	0.55102544
FGF8, BRAF, MAPK12, KL, FGF23, CALM1	6.991811242	0.721623304
TACR3, KL, CNR1, PTH1R, TGFBR2	9.251790634	0.806890825
FGF8, CHD7, TGFB2	28.62272727	0.982016263
PDPK1, FGF8, KL, FGF23	11.52109777	0.983032693
PROK2, HSPG2, HS6ST1, TGFB2, SYK	6.845495312	0.992093836
PPARA, HSPG2, PRKACB	24.10334928	0.996414749
PROK2, CASR, KISS1R, GNAI2, PTH1R, PROKR2, GHRL,	3.056487006	0.998474038
RBP4, RXRA, BMP6	22.33968958	0.998542847
FGF8, KL, FGF23	18.69239332	0.999901875
KL, FGF23	122.1236364	0.999998811
PROK2, FGF8, GNAI2, PTH1R, TGFBR2, TGFB2	3.931018338	0.999999362
MMP8, CTSD, CTSS	14.31136364	0.999999795
GNAI2, CNR1, TGFBR2	12.37739558	0.999999998
MMP8, HSPG2, CTSS	12.05167464	0.999999999
FGF8, KL, FGF23	11.74265734	1
CASR, MGP, AHSG	11.44909091	1
PPARA, TGFBR2, TGFB2	11.44909091	1
FGF8, KL, FGF23	11.16984479	1
PROK2, GNAI2, GLI2, TGFB2, SYK	4.17088922	1
GHRL, WAS	50.88484848	1
TGFBR2, TGFB2	46.97062937	1
TGFB2, SYK	46.97062937	1
HSP90AB1, CALM1	46.97062937	1
FGF23, BMP6	46.97062937	1
CASR, CALM1	43.61558442	1
GLI2, IGFBP3, BMP6	8.806993007	1
PDPK1, PRKACB, SYK	8.723116883	1
SLPI, CTSS, WAS, CHIT1, BMP6	3.625998704	1
FGF23, AHSG	40.70787879	1
ACE2, MME, CMA1	109.038961	0.125222074
GSTM1, GSTA1, GSTM2, CLIC1	28.55782313	0.135123833
DKC1, NHP2, NOP10	79.96190476	0.224205959
GSTM1, GSTA1, GSTM2	54.51948052	0.424601863
AKR1C3, MTHFR, CYP2C9, HSD17B1, CYP2C8, GMPR, I	4.727477477	0.713414333
AKR1C3, CYP2C9, CYP2C8	27.89368771	0.876986437
GSTM1, GSTM2	266.5396825	0.955387797
CYP3A4, NR1I2, CES1	129.1692308	0.004563208
CYP3A4, NR1I2	559.7333333	0.097829844
CYP3A4, NR1I2	156.2046512	0.308749492

NR1I3, NR1I2	117.8385965	0.387177234
NDP, FZD4	1679.2	0.069869643
NDP, FZD4	80.9253012	0.77831129
NDP, FZD4	35.91871658	0.96678385
