# Supplementary material for: Research on the Mechanism of Liuwei Dihuang Decoction for Osteoporosis Based on Systematic Biological Strategies
Source: Evid Based Complement Alternat Med. 2022 Sep 22;2022:7017610. doi: 10.1155/2022/7017610 (PMC9522519; doi:10.1155/2022/7017610)
Supplement: Supplementary Materials — Table S1-1: components meeting the screening criteria. Table S1-2: compound targets for each compound of LDD. Table S2: osteoporosis genes. Table S3: enrichment analysis of clusters based on Gene Ontology (GO) annotation of LDD-osteoporosis PPI network. Table S4: pathway enrichment analysis of LDD-osteoporosis PPI network. Table S5: Reactome pathways of LDD-osteoporosis PPI network. Table S6: Human Transcriptomics Data. Table S7: the biological processes of Human Transcriptomics Data Network. Table S8: the Reactome pathways of Human Transcriptomics Data Network. Table S9: the signaling pathways of Human Transcriptomics Data Network. Table S10: the biological processes of protein arrays data network. Table S11: the Reactome pathways of protein arrays data network. Table S12: the signaling pathways of protein arrays data network. [file 7017610.f1.zip › 7017610.f1/Table S4 (1).pdf]

Table S4 Cell components, molecular functions, signaling pathway of LDD-osteoporosis PPI Ne

Category	Term	Pathway	Count	%	Pvalue
	hsa04068	FoxO signaling pathway	38	0.0328	6.44E-12
	hsa04915	Estrogen signaling pathway	32	0.027621	9.67E-12
	hsa04380	Osteoclast differentiation	37	0.031937	1.46E-11
	hsa04151	PI3K-Akt signaling pathway	66	0.056969	1.76E-11
	hsa04014	Ras signaling pathway	47	0.040569	1.50E-09
	hsa04660	T cell receptor signaling pathway	29	0.025032	1.79E-09
	hsa04912	GnRH signaling pathway	27	0.023305	4.29E-09
	hsa01100	Metabolic pathways	150	0.129474	5.30E-09
	hsa04910	Insulin signaling pathway	34	0.029348	5.33E-09
	hsa04919	Thyroid hormone signaling pathway	30	0.025895	1.28E-08
	hsa04722	Neurotrophin signaling pathway	30	0.025895	3.58E-08
	hsa04010	MAPK signaling pathway	47	0.040569	6.10E-08
	hsa03320	PPAR signaling pathway	20	0.017263	6.10E-07
	hsa04370	VEGF signaling pathway	19	0.0164	6.31E-07
	hsa04668	TNF signaling pathway	26	0.022442	6.33E-07
	hsa04931	Insulin resistance	26	0.022442	7.63E-07
	hsa04066	HIF-1 signaling pathway	23	0.019853	4.21E-06
	hsa04620	Toll-like receptor signaling pathway	24	0.020716	6.84E-06
	hsa00140	Steroid hormone biosynthesis	17	0.014674	7.04E-06
	hsa04012	ErbB signaling pathway	21	0.018126	1.09E-05
Signaling Pathway	hsa04024	cAMP signaling pathway	35	0.030211	1.25E-05
	hsa04920	Adipocytokine signaling pathway	18	0.015537	2.33E-05
	hsa04630	Jak-STAT signaling pathway	27	0.023305	6.24E-05
	hsa04310	Wnt signaling pathway	25	0.021579	1.94E-04
	hsa04621	NOD-like receptor signaling pathway	14	0.012084	3.43E-04
	hsa04152	AMPK signaling pathway	22	0.01899	6.20E-04
	hsa04062	Chemokine signaling pathway	29	0.025032	7.05E-04
	hsa04150	mTOR signaling pathway	13	0.011221	0.0017
	hsa04350	TGF-beta signaling pathway	16	0.013811	0.002285
	hsa00982	Drug metabolism - cytochrome P450	14	0.012084	0.002354
	hsa04064	NF-kappa B signaling pathway	16	0.013811	0.003255
	hsa04750	Inflammatory mediator regulation of T	17	0.014674	0.004237
	hsa04022	cGMP-PKG signaling pathway	23	0.019853	0.006659
	hsa04810	Regulation of actin cytoskeleton	28	0.024169	0.008369
	hsa04060	Cytokine-cytokine receptor interactio	31	0.026758	0.009918
	hsa00260	Glycine, serine and threonine metabol	9	0.007768	0.010307
	hsa04921	Oxytocin signaling pathway	21	0.018126	0.014866
	hsa00480	Glutathione metabolism	10	0.008632	0.017768
	hsa04210	Apoptosis	11	0.009495	0.023256
	hsa04390	Hippo signaling pathway	20	0.017263	0.029933
	hsa00534	Glycosaminoglycan biosynthesis - hep	6	0.005179	0.03744
	GO:0005576	extracellular region	215	29.17232	1.69E-46
	GO:0005615	extracellular space	193	26.18725	8.51E-41
	GO:0070062	extracellular exosome	204	27.67978	2.33E-37
	GO:0005829	cytosol	350	47.48982	1.35E-34

Cell Components	GO:1904813	ficolin-1-rich granule lumen	32	4.341927	8.00E-18
	GO:0005788	endoplasmic reticulum lumen	48	6.51289	4.85E-17
	GO:0005737	cytoplasm	290	39.34871	1.28E-14
	GO:0034774	secretory granule lumen	27	3.663501	5.34E-14
	GO:0043235	receptor complex	35	4.748982	5.74E-13
	GO:0016020	membrane	157	21.30258	7.42E-12
	GO:0045121	membrane raft	35	4.748982	3.78E-11
	GO:0009986	cell surface	59	8.005427	4.70E-11
	GO:0032991	macromolecular complex	63	8.548168	6.39E-11
	GO:0048471	perinuclear region of cytoplasm	65	8.819539	2.94E-10
	GO:0005925	focal adhesion	42	5.698779	1.27E-08
	GO:0031012	extracellular matrix	31	4.206242	1.86E-08
	GO:0005739	mitochondrion	93	12.61872	4.02E-08
	GO:0043202	lysosomal lumen	18	2.442334	6.21E-08
	GO:0072562	blood microparticle	21	2.849389	2.87E-07
	GO:0005654	nucleoplasm	196	26.5943	2.96E-07
Molecular Function	GO:0042802	identical protein binding	168	22.79512	4.36E-30
	GO:0004879	RNA polymerase II transcription facto	27	3.663501	1.07E-22
	GO:0019899	enzyme binding	54	7.327001	9.15E-16
	GO:0008270	zinc ion binding	86	11.66893	3.27E-15
	GO:0004714	transmembrane receptor protein tyrosi	29	3.934871	5.53E-15
	GO:0004713	protein tyrosine kinase activity	28	3.799186	8.51E-15
	GO:0042803	protein homodimerization activity	75	10.17639	8.58E-15
	GO:0005515	protein binding	582	78.96879	3.52E-14
	GO:0004712	protein serine/threonine/tyrosine kina	54	7.327001	1.27E-13
	GO:0005524	ATP binding	118	16.01085	1.72E-12
	GO:0004252	serine-type endopeptidase activity	33	4.477612	3.16E-12
	GO:0008233	peptidase activity	24	3.256445	8.53E-12
	GO:0008083	growth factor activity	30	4.070556	1.14E-11
	GO:0005125	cytokine activity	32	4.341927	1.41E-11
	GO:0005496	steroid binding	14	1.899593	2.97E-11
	GO:0005102	receptor binding	47	6.377205	6.60E-11
	GO:0004175	endopeptidase activity	21	2.849389	8.93E-11
	GO:0004715	non-membrane spanning protein tyros	16	2.170963	1.12E-10
	GO:0043565	sequence-specific DNA binding	40	5.427408	7.44E-10
	GO:0019901	protein kinase binding	50	6.784261	3.19E-09

etwork

Genes	Fold Enrichment	Bonferroni
HRAS, GRB2, IL10, TGFB1, TGFB2, AKT1, IGF1R, PDPK1	3.559783201	1.81E-09
HSP90AB1, HRAS, GNAI2, GRB2, MMP9, HSPA1A, MMP.	4.057509401	2.72E-09
CALCR, TNF, GRB2, CSF1, PPARG, ACP5, TGFB1, TGFB2	3.545481139	4.11E-09
HSP90AB1, HRAS, FGF8, PGF, FGF17, AKT1, PDPK1, CR	2.401428118	4.94E-09
FGFR2, FGFR1, FGF8, HRAS, GRB2, PGF, FGF17, CSF1, E	2.610562948	4.22E-07
HRAS, TNF, GRB2, IL10, AKT1, FOS, CDC42, PDPK1, IFN	3.640346715	5.02E-07
CGA, HRAS, GRB2, GNRHR, MMP2, SRC, CDC42, PTK2E	3.724492661	1.20E-06
CYP3A4, CYP24A1, LDHB, ALAD, SC5D, GNPDA1, GNPI	1.54465788	1.49E-06
HRAS, GRB2, PDE3B, HK1, AKT1, PDPK1, INS, PRKACA	3.092748334	1.50E-06
HRAS, THRA, THRB, SRC, CTNNB1, AKT1, PDPK1, GAT	3.274674706	3.59E-06
HRAS, GRB2, MAPKAPK2, AKT1, CDC42, PDPK1, MAP3	3.138229927	1.01E-05
FGFR2, FGFR1, FGF8, HRAS, TNF, GRB2, FGF17, HSPA1.	2.331965321	1.71E-05
PPARA, PPARD, ACADM, RXRB, RXRA, PPARG, ADIPO	3.747140211	1.72E-04
PIK3CG, HRAS, MAP2K1, RAF1, MAPKAPK2, SRC, KDR	3.909925811	1.77E-04
TNF, CSF1, MMP9, JAG1, CCL5, MMP3, AKT1, FOS, CAS	3.050242172	1.78E-04
PPARA, TNF, AKT1, NR1H2, PDPK1, INS, CREB3L1, NOS	3.021999189	2.14E-04
EGFR, PIK3CG, TF, IL6, MAP2K1, IGF1, HK1, PDHB, AK1	3.007470347	0.001182297
PIK3CG, IL6, TNF, MAP2K1, MAPK10, STAT1, CCL5, AK	2.8421705	0.001919099
HSD3B2, CYP3A4, STS, CYP11A1, CYP11B1, CYP21A2, F	3.679304052	0.001976109
EGFR, PIK3CG, HRAS, ERBB4, BRAF, MAP2K1, GRB2, R	3.030015102	0.003067464
PPARA, GNAI2, ADORA2A, PDE3B, SOX9, AKT1, FOS, R	2.218950453	0.003504749
PPARA, TNF, RXRB, LEPR, RXRA, MAPK10, POMC, ADI	3.227893639	0.006522776
GRB2, LEPR, BCL2L1, IL10, IL11, AKT1, IL12RB1, IFNG,	2.337440221	0.017375239
PPARD, WNT16, WNT3A, MMP7, CTNNB1, WNT1, CSNK	2.274079657	0.053032107
HSP90AB1, IL6, TNF, HSP90AA1, MAPK10, CCL5, MAPK	3.138229927	0.091837868
PIK3CG, HMGCR, LEPR, PFKFB1, PPARG, IGF1, ADIPOC	2.245237671	0.159840207
HRAS, GNAI2, GRB2, CCL5, SRC, AKT1, CDC42, PTK2, F	1.957175653	0.179700808
PIK3CG, TNF, BRAF, IGF1, AKT1, MAPK1, PDPK1, EIF4I	2.813585452	0.379966408
BMP4, MAPK1, NOG, BMP2, TNF, SP1, TGFBR1, MAPK3	2.391032325	0.474234419
GSTA1, CYP3A4, GSTA3, CYP2C9, MAOA, CYP2C8, MA(	2.584424646	0.484287993
TNF, XIAP, MALT1, BCL2L1, BTK, PRKCQ, TNFRSF11A,	2.308582935	0.59989405
PIK3CG, IGF1, MAPK10, PPP1CC, SRC, PRKCQ, MAPK12	2.177547296	0.69669725
GNAI2, MAP2K1, PDE3B, RAF1, PPP1CC, AKT1, MAPK1,	1.827323755	0.847007755
FGFR2, FGFR1, ITGAL, HRAS, FGF8, FGF17, SRC, CDC4:	1.673722628	0.905731883
TNF, LEPR, CSF1, TNFSF15, CCL5, IL10, TGFB1, IL11, T(	1.60140128	0.939237524
SHMT1, GATM, SDS, MAOA, BHMT, MAOB, PHGDH, PS	2.896827625	0.945602442
EGFR, HRAS, GNAI2, MAP2K1, RAF1, PPP1CC, SRC, MA	1.757408759	0.985132874
GSTM1, GSTA1, GSTM2, GSR, GSTA3, SRM, GGT1, GST(	2.461356805	0.99351219
PIK3CG, AKT1, CASP3, TNF, XIAP, CASP7, TP53, APAF1	2.227130916	0.998656041
BMP4, WNT16, BMP2, WNT3A, TGFBR1, TGFBR2, SMAI	1.662638372	0.999804431
B3GAT3, B3GALT6, NDST1, XYLT2, HS6ST1, B4GALT7	3.138229927	0.999977958
CDA, SPARC, ANTXR2, LGALS3, PNP, KDR, PROK2, TN	2.782380214	1.07E-43
IL1RN, SPARC, XYLT2, LGALS3, CHEK1, TNFSF11, IL12	2.766912952	5.38E-38
IL1RN, PITPNA, LGALS3, PNP, PRKACA, PRKACB, EPHI	2.520944873	1.47E-34
GLTP, PNMT, CDA, NUP107, GORAB, PITPNA, HERC2, F	1.774400203	8.53E-32

CFD, GPI, CDA, ASAH1, VCP, SERPINA1, CANT1, HSP90	7.070126227	5.06E-15
FKBP10, SERPINA1, CSF1, INS, BMP15, STS, BGLAP, SE	4.283529245	3.07E-14
GLTP, IL1RN, SPARC, GORAB, PITPNA, SPIB, LGALS3,	1.482009765	8.07E-12
CFD, GPI, CDA, VCP, HSP90AB1, GSTP1, PYGL, INS, AL	6.432277883	3.37E-11
NOTCH2, NOTCH3, LRP5, TNFRSF11B, PTH1R, LRP8, EC	4.439286433	3.63E-10
GLTP, SPARC, NUP107, CLEC4M, CSF1, FMR1, TNF, IGF	1.712978114	4.69E-09
RTN4R, SRC, TNF, CD1A, EGFR, LRP6, GNAI2, HK1, DPI	3.835543478	2.39E-08
SPARC, HSP90AB1, ANTXR2, ITGAL, TNF, GHR, LGALS	2.611320854	2.97E-08
HSP90AB1, REG1A, KIF11, NR3C1, TNF, PPP1CC, AKT2,	2.494211799	4.04E-08
HSP90AB1, CSF1, HFE, FMR1, REG1A, ARHGAP1, AKR1	2.361787856	1.86E-07
SRC, RND3, EGFR, DPP4, CDC42, PPP1CC, PLAU, FLRT3	2.726689676	8.02E-06
ELN, TNFRSF11B, FGF1, FBLN5, ADAMTS4, ADAMTS2,	3.317573879	1.18E-05
GSK3B, HSP90AB1, NR3C1, HK1, GHR, IFIH1, PPP1CC, A	1.799362104	2.54E-05
HSPA8, ARSA, ASAH1, HSP90AA1, HEXB, GBA, CTSV, I	5.136888587	3.93E-05
APCS, BCHE, HSPA8, TGFB1, C1S, AHSG, C1R, FGG, HP,	3.99535779	1.82E-04
GORAB, SOX3, HERC2, AKT2, CHEK1, AKT1, SOX9, PRI	1.396193674	1.87E-04
GLTP, CDA, CSF1, FMR1, REG1A, TNF, IGF1R, PNP, LEP	2.514352419	6.06E-27
RARG, THRB, THRA, NR1I3, NR1I2, HNF4G, RORA, PIK3	12.7513587	1.49E-19
THRB, MCM9, CCND1, AKT1, BANF1, RAC1, PARP1, RF	3.605096176	1.23E-12
CDA, RARG, THRB, THRA, HNF4G, RORA, NR3C1, GLI2	2.538464926	4.47E-12
ITK, SRC, EGFR, IGF1R, ERBB4, KDR, ABL1, PTK2B, CS	6.375679348	7.71E-12
ITK, SRC, EGFR, IGF1R, ERBB4, KDR, ABL1, PTK2B, CS	6.551156761	1.19E-11
CDA, HSP90AB1, CSF1, FMR1, PTH1R, GHR, EEA1, HPG	2.713055042	1.19E-11
PNMT, CDA, NUP107, SPIB, NDST1, AKT2, CREB3L1, AI	1.185699115	4.89E-11
GSK3B, ITK, PIK3CG, IGF1R, AKT2, CHEK1, PIM1, KDR,	3.202666835	1.77E-10
IGF1R, AKT2, CHEK1, PIM1, KDR, ENPP1, AKT1, PRKAC	1.95410432	2.40E-09
CFD, C1S, C1R, HPN, HP, CTSV, CTSS, DPP4, PLAU, CTS	4.406228659	4.40E-09
MMP1, HPN, MMP3, PEPD, MMP8, MMP9, MALT1, ZMPS	5.942380751	1.19E-08
GPI, CSF1, REG1A, FGF1, TYMP, BMP15, FGF8, IL12A, F	4.60892483	1.59E-08
GPI, IL1RN, CSF1, TNFRSF11B, TNF, BMP15, SPP1, TNFS	4.295194508	1.96E-08
AKR1D1, ESRRG, CYP3A4, NR3C1, ESR1, ESR2, NR3C2,	11.90126812	4.12E-08
CALCA, SRC, HFE, GBA, LAMA3, LAMC2, LRP6, DPP4, S	2.989096552	9.17E-08
ACE, MMP7, MME, MMP1, CMA1, MMP2, MMP3, MMEL	6.227407735	1.24E-07
ITK, SYK, SRC, TNK2, BAZ1B, PTK2, CLK1, ZAP70, HCK	8.870510397	1.55E-07
GCM2, RARG, THRB, THRA, NR1I3, NR1I2, PRDM5, HNI	3.100634333	1.03E-06
GSK3B, HSP90AB1, CCNT1, KIF11, NR3C1, RND3, GHR,	2.560513794	4.43E-06
