# Supplementary material for: Research on the Mechanism of Liuwei Dihuang Decoction for Osteoporosis Based on Systematic Biological Strategies
Source: Evid Based Complement Alternat Med. 2022 Sep 22;2022:7017610. doi: 10.1155/2022/7017610 (PMC9522519; doi:10.1155/2022/7017610)
Supplement: Supplementary Materials — Table S1-1: components meeting the screening criteria. Table S1-2: compound targets for each compound of LDD. Table S2: osteoporosis genes. Table S3: enrichment analysis of clusters based on Gene Ontology (GO) annotation of LDD-osteoporosis PPI network. Table S4: pathway enrichment analysis of LDD-osteoporosis PPI network. Table S5: Reactome pathways of LDD-osteoporosis PPI network. Table S6: Human Transcriptomics Data. Table S7: the biological processes of Human Transcriptomics Data Network. Table S8: the Reactome pathways of Human Transcriptomics Data Network. Table S9: the signaling pathways of Human Transcriptomics Data Network. Table S10: the biological processes of protein arrays data network. Table S11: the Reactome pathways of protein arrays data network. Table S12: the signaling pathways of protein arrays data network. [file 7017610.f1.zip › 7017610.f1/Table S5.pdf]

Table S5 Reactome pathways

Pathway i	Pathway name	Entities ratio	PValue	FDR	Genes
R-HSA-67	Interleukin-4 and Int	0.014889563	1.11E-16	4.94E-14	MAOB;MAOA;RORA
R-HSA-12	Cytokine Signaling i	0.08785548	1.11E-16	4.94E-14	IL1RN;NUP107;CSF1;
R-HSA-90	Signaling by Recept	0.038176558	6.13E-12	1.81E-09	SPARC;PDE3B;LAM
R-HSA-19	Negative regulation	0.009455931	1.69E-11	4.28E-09	SRC;PIK3R1;FGF1;E
R-HSA-68	PI5P, PP2A and IER	0.008891398	4.68E-11	1.04E-08	SRC;PIK3R1;FGF1;E
R-HSA-44	Interleukin-12 family	0.006774398	1.03E-09	1.83E-07	IL10;GSTO1;STAT1;F
R-HSA-40	SUMOylation of intr	0.002822666	4.43E-09	6.09E-07	THRB;THRA;VDR;N
R-HSA-90	Interleukin-12 signal	0.005927599	4.56E-09	6.09E-07	IL10;GSTO1;PITPNA;
R-HSA-14	Extracellular matrix	0.023216428	9.49E-09	1.12E-06	SPARC;ELN;LAMC2;
R-HSA-14	Degradation of the e	0.010443864	1.04E-08	1.15E-06	ELN;LAMA3;LAMC2
R-HSA-74	Insulin receptor sign	0.005010232	1.54E-08	1.61E-06	KL;PDPK1;INSR;PDE
R-HSA-24	IRS-related events tr	0.004798532	3.22E-08	3.15E-06	KL;PDPK1;PDE3B;IG
R-HSA-90	Extra-nuclear estrog	0.007691765	5.77E-08	5.14E-06	SRC;PIK3R1;EGFR;G
R-HSA-24	IGF1R signaling cas	0.005010232	6.72E-08	5.37E-06	KL;PDPK1;PDE3B;IG
R-HSA-24	Signaling by Type 1	0.005080799	8.51E-08	6.55E-06	KL;PDPK1;PDE3B;IG
R-HSA-89	Gene and protein ex	0.005151365	1.07E-07	7.40E-06	IL10;CDC42;MTAP;C
R-HSA-76	Platelet activation, si	0.020676029	1.09E-07	7.40E-06	SPARC;SERPINA1;PI
R-HSA-38	Regulation of Insulin	0.008961965	1.17E-07	7.60E-06	SPARC;SERPINA1;C
R-HSA-15	Activation of Matrix	0.002469833	1.55E-07	9.79E-06	MMP7;CMA1;MMP1;
R-HSA-67	Neutrophil degranula	0.033871992	2.01E-07	1.22E-05	CDA;GPI;HSP90AB1;
R-HSA-96	FOXO-mediated tran	0.003457766	4.45E-07	2.62E-05	IGFBP1;POMC;G6PC;
R-HSA-56	Negative regulation	0.002752099	5.95E-07	3.27E-05	KL;SRC;BRAF;PTPN
R-HSA-14	Collagen degradation	0.004869099	7.23E-07	3.83E-05	MMP7;MME;MMP1;
R-HSA-74	Signaling by Insulin	0.006774398	7.96E-07	4.14E-05	KL;PDPK1;INSR;PDE
R-HSA-11	IRS-mediated signal	0.004516266	9.61E-07	4.48E-05	KL;PDPK1;PDE3B;PT
R-HSA-10	PI3K Cascade	0.004092866	9.95E-07	4.48E-05	KL;PDPK1;PDE3B;PT
R-HSA-74	Gene expression (Tr	0.12850187	1.41E-06	6.19E-05	NUP107;CCNT1;ROR
R-HSA-56	Signaling by FGFR1	0.004304566	2.00E-06	8.58E-05	KL;SRC;BRAF;PTPN
R-HSA-14	Collagen formation	0.007338932	3.07E-06	1.29E-04	CRTAP;MMP7;LAMB
R-HSA-89	RUNX2 regulates os	0.002399266	3.50E-06	1.44E-04	COL1A1;AR;BGLAP;
R-HSA-56	Negative regulation	0.002893233	5.37E-06	2.15E-04	FGF17;FGF8;SRC;MA
R-HSA-88	Transcriptional regul	0.010373298	6.19E-06	2.41E-04	GSK3B;STAT1;SRC;F
R-HSA-56	PI-3K cascade:FGFR	0.001975866	1.52E-05	5.46E-04	FGF17;KL;FGF8;GRB
R-HSA-90	Intracellular signalin	0.025474561	1.73E-05	6.06E-04	GSK3B;SRC;XIAP;PI
R-HSA-12	PIP3 activates AKT	0.022228495	1.87E-05	6.34E-04	GSK3B;SRC;XIAP;PI
R-HSA-96	Estrogen-dependent	0.002046433	2.08E-05	6.87E-04	CCND1;EGF;AKT2;A
R-HSA-21	Regulation of gene e	0.002469833	2.37E-05	7.59E-04	PKLR;GK;AKT2;HNF
R-HSA-12	Nuclear signaling by	0.003316633	2.39E-05	7.63E-04	WWOX;ADAM17;SP
R-HSA-56	Downstream signalin	0.002893233	2.46E-05	7.63E-04	FGF17;KL;FGF8;FLR
R-HSA-90	Interferon alpha/beta	0.012984264	2.49E-05	7.63E-04	ISG20;PTPN1;RNASE
R-HSA-12	Signaling by ERBB4	0.005715899	2.54E-05	7.63E-04	WWOX;SPARC;EGF;
R-HSA-89	ESR-mediated signal	0.017994496	2.63E-05	7.63E-04	HSP90AB1;CCNT1;S
R-HSA-56	FRS-mediated FGFR	0.002117	2.82E-05	8.19E-04	FGF17;KL;FGF8;GRB
R-HSA-56	PI-3K cascade:FGFR	0.002187566	3.78E-05	0.001021	FGF17;FGF8;GRB2;P
R-HSA-19	FGFR1 ligand bindin	0.0014819	4.61E-05	0.001118	FGF17;KL;FGF8;AN
R-HSA-44	Other interleukin sig	0.022440195	4.72E-05	0.001118	CSF1;SRC;PDE3B;PI
R-HSA-44	VEGFA-VEGFR2 P	0.008820831	5.71E-05	0.001255	HSP90AA1;PDPK1;N
R-HSA-19	Signaling by VEGF	0.009455931	6.32E-05	0.001378	SRC;PIK3R1;CDC42;
R-HSA-56	FRS-mediated FGFR	0.002328699	6.56E-05	0.001378	FGF17;FGF8;GRB2;P
R-HSA-56	Downstream signalin	0.002893233	1.05E-04	0.002119	FGF17;FGF8;GRB2;P
R-HSA-56	SHC-mediated casca	0.002046433	1.06E-04	0.002119	FGF17;KL;FGF8;GRB
R-HSA-56	MAPK family signal	0.023428128	1.25E-04	0.002502	SRC;FGF1;EGFR;CD
R-HSA-11	GPVI-mediated activ	0.003034366	1.62E-04	0.003239	COL1A1;CDC42;COL
R-HSA-96	FOXO-mediated tran	0.007762332	1.74E-04	0.003477	IGFBP1;G6PC;SMAD

R-HSA-56(	MAPK1/MAPK3 sig	0.019829229	1.98E-04	0.003759	SRC;FGF1;EGFR;FGF
R-HSA-12:	Signaling by ERBB2	0.004727966	2.83E-04	0.005092	USP8;HSP90AA1;EGF
R-HSA-56:	Negative regulation (	0.002399266	3.71E-04	0.006302	FGF17;FGF8;SRC;MA
R-HSA-56:	Negative feedback re	5.65E-04	4.30E-04	0.007308	MAP2K1;MAPK1;BR.
R-HSA-76(	Response to elevated	0.010161598	4.43E-04	0.007408	CFD;SPARC;CANT1;S
R-HSA-56:	RAF/MAP kinase ca	0.019335262	4.59E-04	0.007408	SRC;FGF1;EGFR;FGF
R-HSA-56:	Negative regulation (	0.002469833	4.63E-04	0.007408	FGF17;FGF8;SRC;MA
R-HSA-67(	Interleukin-10 signal	0.006068732	4.73E-04	0.007563	IL10;IL1A;IL1RN;IL6;
R-HSA-11:	Platelet degranulatio	0.009667631	5.24E-04	0.008376	CFD;TGFB2;CANT1;T
R-HSA-33(	Signaling by TGF-be	6.35E-04	7.28E-04	0.010916	FKBP1A;SMAD3;TGF
R-HSA-89:	Metabolism of steroi	0.022651895	7.44E-04	0.011158	AKR1D1;AKR1B1;HS
R-HSA-12:	Antigen processing-C	0.013195964	8.10E-04	0.011986	BTK;TAP1;HLA-A;CA
R-HSA-56:	Phospholipase C-me	0.001764166	8.19E-04	0.011986	FGF17;FGF8;FGF1;FC
R-HSA-89:	RUNX2 regulates ge	9.88E-04	8.41E-04	0.011986	MMP13;AKT2;AKT1;
R-HSA-56:	Signaling by FGFR3	0.003669466	8.56E-04	0.011986	FGF17;FGF8;SRC;MA
R-HSA-21:	Molecules associated	0.002681533	8.61E-04	0.012053	BMP4;EFEMP2;BMP2
R-HSA-88:	MET promotes cell r	0.003175499	8.79E-04	0.012304	COL1A1;COL2A1;CO
R-HSA-88:	MET activates PTK2	0.002258133	9.76E-04	0.013597	COL1A1;COL2A1;CO
R-HSA-19(	GRB2 events in ERE	0.001411333	0.00103	0.013597	ERBB4;EGF;GRB2;HI
R-HSA-38:	CD28 co-stimulation	0.002752099	0.001044	0.013597	CDC42;PDPK1;LCK;S
R-HSA-19(	Metabolism of steroi	0.004869099	0.001129	0.014316	HSD3B2;AKR1B1;HS
R-HSA-37:	Negative regulation (	0.0010585	0.001193	0.014316	SFRP1;WNT3A;LRP5
R-HSA-18:	Signaling by activate	0.0010585	0.001193	0.014316	FGF17;FGF8;FGF1;FC
R-HSA-11:	Cytochrome c-media	0.0010585	0.001193	0.014316	CASP7;APAF1;CASP3
R-HSA-12:	ER-Phagosome path	0.011643497	0.001279	0.015354	BTK;TAP1;HLA-A;CA
R-HSA-91:	Interferon Signaling	0.027662127	0.001284	0.015402	PTPN1;RNASEL;NUP
R-HSA-56:	Signaling by FGFR2	0.006139299	0.001426	0.017107	SRC;BRAF;PTPN11;P
R-HSA-56:	Signaling by FGFR2	0.004445699	0.001441	0.017294	FGF17;FGF8;GRB2;PI
R-HSA-19:	Synthesis of bile acid	0.002399266	0.001474	0.017692	CYP27A1;HSD3B7;RX
R-HSA-15:	Gamma-carboxylatic	0.001129066	0.001649	0.019275	F7;F10;BGLAP;GC;F2
R-HSA-41:	Calcitonin-like ligan	7.76E-04	0.001752	0.019275	CALCA;CALCR;IAPP
R-HSA-45(	MAPK targets/ Nucl	0.002469833	0.001791	0.019697	MAPK10;MAPK8;JUN
R-HSA-88(	Downregulation of E	0.002540399	0.002159	0.022389	USP8;HSP90AA1;ERE
R-HSA-19(	FGFR2c ligand bindi	0.001199633	0.002225	0.022389	FGF17;FGF8;FGF1;FC
R-HSA-16:	PKA-mediated phosp	4.94E-04	0.002239	0.022389	MLXIPL;PFKFB1;PRI
R-HSA-11:	SMAC (DIABLO) b	4.94E-04	0.002239	0.022389	CASP7;APAF1;CASP3
R-HSA-11:	SMAC(DIABLO)-m	4.94E-04	0.002239	0.022389	CASP7;APAF1;CASP3
R-HSA-19(	FGFR1c and Klotho	4.94E-04	0.002239	0.022389	KL;FGF23;FGFR1
R-HSA-88:	Gastrin-CREB signal	0.001623033	0.002254	0.02254	MMP1;MMP3;MAPK1
R-HSA-18(	Regulation of beta-ce	0.004727966	0.002441	0.02441	KAT2B;PKLR;GK;AK
R-HSA-52:	VEGFR2 mediated v	0.003104933	0.002493	0.024931	HSP90AA1;PDPK1;NC
R-HSA-67(	Interleukin-6 family	0.002117	0.002535	0.025348	IL11;IL6;STAT1;PTPN
R-HSA-21:	EGFR Transactivatic	8.47E-04	0.002544	0.025442	MMP1;MMP3;GRB2;I
R-HSA-16(	Toll-like Receptor C	0.012984264	0.002647	0.026472	JUN;MAP2K1;MAP3K
R-HSA-68(	Signaling by modera	0.003669466	0.002686	0.026483	MAP2K1;SRC;FGG;B
R-HSA-88(	Transcriptional regul	0.003669466	0.002686	0.026483	WWOX;KIT;APOE;CC
R-HSA-56:	PI-3K cascade:FGFF	0.0016936	0.002846	0.026483	FGF17;FGF8;GRB2;PT
R-HSA-11:	Apoptotic factor-med	0.0012702	0.002943	0.026483	CASP7;APAF1;CASP3
R-HSA-68(	Paradoxical activatic	0.003740032	0.003096	0.027866	MAP2K1;SRC;FGG;B
R-HSA-90(	Signaling by Non-Re	0.004939666	0.003516	0.030558	PTPN1;CCND1;ERBB
R-HSA-56:	PI-3K cascade:FGFF	0.001764166	0.00355	0.030558	FGF17;FGF8;GRB2;PT
R-HSA-44:	Signal transduction b	0.001764166	0.00355	0.030558	MAP2K1;CSNK2A1;M
R-HSA-11(	MAPK3 (ERK1) act	9.17E-04	0.003568	0.030558	IL6;MAP2K1;MAPK1
R-HSA-37:	Hormone ligand-binc	9.17E-04	0.003568	0.030558	FSHR;GNRHR;GNRH
R-HSA-74:	Signal attenuation	9.17E-04	0.003568	0.030558	INSR;MAPK1;GRB2;I
R-HSA-11:	SMAC, XIAP-regula	5.65E-04	0.003598	0.030558	CASP7;APAF1;CASP3

R-HSA-448	Interleukin-1 process	5.65E-04	0.003598	0.030558	IL1A;CMA1;IL1B;CA
R-HSA-566	CLEC7A/inflammas	5.65E-04	0.003598	0.030558	IL1B;MALT1
R-HSA-111	Activation of caspase	5.65E-04	0.003598	0.030558	CASP7;APAF1;CASP3
R-HSA-330	Loss of Function of S	5.65E-04	0.003598	0.030558	SMAD3;TGFB1;TGFB
R-HSA-187	Signalling to ERKs	0.002752099	0.003637	0.030558	SRC;MAPKAPK2;MA
R-HSA-364	Loss of Function of T	2.82E-04	0.003804	0.030558	TGFB1;TGFBR1;TGF
R-HSA-678	ERBB2 Regulates Ce	0.001340766	0.00382	0.030558	ERBB4;EGF;RHOA;E
R-HSA-129	Spry regulation of FG	0.001340766	0.00382	0.030558	SRC;MAPK1;GRB2;B
R-HSA-567	MAP2K and MAPK	0.003316633	0.003945	0.03156	MAP2K1;SRC;FGG;M
R-HSA-451	Interleukin-2 family	0.003316633	0.003945	0.03156	SYK;STAT1;LCK;PTK
R-HSA-337	Attenuation phase	0.003316633	0.003945	0.03156	HSPA8;HSP90AA1;HS
R-HSA-565	FRS-mediated FGFR	0.001834733	0.004379	0.035036	FGF17;FGF8;GRB2;PT
R-HSA-680	Signaling by BRAF a	0.005080799	0.004426	0.035404	MAP2K1;SRC;FGG;B
R-HSA-183	FGFR1 mutant recep	0.003387199	0.004555	0.036441	FGF17;FGF8;STAT1;G
R-HSA-902	Interleukin-2 signalin	9.88E-04	0.004858	0.038868	SYK;LCK;PTK2B;JAK
R-HSA-381	Transcriptional regul	0.007621198	0.004935	0.039483	FABP4;RXRA;TGFB1
R-HSA-566	RHO GTPases Activ	0.002893233	0.004997	0.039976	CDC42;BTK;ABL1;W
R-HSA-183	FGFR2 mutant recep	0.003457766	0.005237	0.041896	FGF17;FGF8;FGF1;FG
R-HSA-565	Signaling by FGFR1	0.004022299	0.005267	0.042136	FGF17;FGF8;STAT1;G
R-HSA-565	Downstream signalin	0.002399266	0.005309	0.042473	FGF17;FGF8;GRB2;PT
R-HSA-565	FRS-mediated FGFR	0.0019053	0.005347	0.04278	FGF17;FGF8;GRB2;PT
R-HSA-159	Transport of gamma-	6.35E-04	0.005429	0.043432	F7;F10;BGLAP;F2
R-HSA-163	Triglyceride cataboli	0.002963799	0.00581	0.046484	PPP1CC;FABP3;FABP
R-HSA-565	Downstream signalin	0.002469833	0.006272	0.047057	FGF17;FGF8;GRB2;PT
R-HSA-125	SHC1 events in ERB	0.002469833	0.006272	0.047057	ERBB4;EGF;GRB2;PR
R-HSA-242	SHC-related events t	0.0010585	0.006449	0.047057	IGF2;GRB2;IGF1;HRA
R-HSA-112	RAF-independent M	0.001975866	0.006468	0.047057	IL6;MAP2K1;MAPK1
R-HSA-337	HSF1 activation	0.003034366	0.006722	0.047057	VCP;HSP90AA1;HSP9
R-HSA-177	Signaling by EGFR	0.004163432	0.00673	0.04711	CDC42;ADAM17;EGF
R-HSA-373	Netrin-1 signaling	0.004163432	0.00673	0.04711	CDC42;MAPK8;HJV;S
R-HSA-880	MET activates PTPN	3.53E-04	0.007022	0.049157	GRB2;PTPN11;MET
R-HSA-196	PI3K events in ERBB	0.001552466	0.007614	0.053298	ERBB4;EGF;GRB2;PI
R-HSA-376	Deactivation of the b	0.003104933	0.00774	0.054182	SOX3;AKT2;AKT1;CT
R-HSA-203	Activated point muta	0.002046433	0.007753	0.054274	FGF17;FGF8;FGF1;FG
R-HSA-895	RUNX3 regulates W	7.06E-04	0.007797	0.054577	CCND1;CTNNB1;TCF
R-HSA-894	RUNX3 Regulates Im	7.06E-04	0.007797	0.054577	SPP1;ITGAL
R-HSA-181	Toll Like Receptor 2	0.008044598	0.008181	0.057264	JUN;MAP2K1;MAP3K
R-HSA-168	Toll Like Receptor T	0.008044598	0.008181	0.057264	JUN;MAP2K1;MAP3K
R-HSA-898	Interleukin-35 Signal	0.001129066	0.008373	0.058614	STAT1;IL12A;JAK2;IL
R-HSA-877	Regulation of IFNG	0.001129066	0.008373	0.058614	PTPN1;IFNG;STAT1;I
R-HSA-167	Trafficking and proc	0.001129066	0.008373	0.058614	CTSK;CTSV;CTSS;CT
R-HSA-166	Signaling by NTRKs	0.008115165	0.008859	0.06201	SRC;BRAF;PTPN11;P
R-HSA-388	Costimulation by the	0.006844965	0.009221	0.064548	PDPK1;SRC;PTPN11;
R-HSA-680	Signaling by high-kin	0.003246066	0.010124	0.070866	MAP2K1;SRC;FGG;M
R-HSA-680	Regulation of TP53 E	0.003246066	0.010124	0.070866	CCNA2;PDPK1;AKT2
R-HSA-364	TGFBR2 MSI Frame	1.41E-04	0.010586	0.074099	TGFB1;TGFBR2
R-HSA-105	Interleukin-6 signalin	0.001199633	0.010662	0.074637	IL6;STAT1;PTPN11;J
R-HSA-125	Downregulation of E	7.76E-04	0.010758	0.075305	ERBB4;SRC
R-HSA-373	Class B/2 (Secretin f	0.006986098	0.010925	0.076475	CALCA;CALCR;WNT
R-HSA-885	Signaling by FGFR2	0.0016936	0.011314	0.079199	FGF1;FGFR2
R-HSA-143	Regulation of KIT si	0.0012702	0.013346	0.080075	LCK;SRC;KIT;GRB2;
R-HSA-171	p38MAPK events	0.0012702	0.013346	0.080075	SRC;MAPKAPK2;MA
R-HSA-884	ERBB2 Activates PT	0.0012702	0.013346	0.080075	ERBB4;EGF;EGFR
R-HSA-565	SHC-mediated casca	0.001764166	0.013574	0.081444	FGF17;FGF8;GRB2;FG
R-HSA-167	Signalling to RAS	0.001764166	0.013574	0.081444	SRC;MAPKAPK2;GR
R-HSA-389	CTLA4 inhibitory si	0.001764166	0.013574	0.081444	LCK;SRC;AKT2;AKT

R-HSA-17	Telomere Extension	8.47E-04	0.014361	0.086168	TERT;DKC1;NHP2;W
R-HSA-11	MAPK1 (ERK2) act	8.47E-04	0.014361	0.086168	IL6;MAPK1;PTPN11;
R-HSA-89	Triglyceride metabol	0.004657399	0.014467	0.086805	PPP1CC;FABP3;FAB
R-HSA-46	Disassembly of the d	0.002328699	0.014826	0.088956	GSK3B;WNT3A;LRP
R-HSA-16	Collagen biosynthesi	0.005363065	0.016044	0.096265	COL1A1;ADAMTS2;
R-HSA-56	SHC-mediated casca	0.001834733	0.016132	0.096789	FGF17;FGF8;GRB2;F
R-HSA-38	CD28 dependent PI3	0.001834733	0.016132	0.096789	PDPK1;LCK;AKT2;A
R-HSA-18	Digestion of dietary	0.001834733	0.016132	0.096789	CHIT1;AMY2A;AMY
R-HSA-21	TGF-beta receptor si	0.001340766	0.016451	0.098704	FKBP1A;TGFB1;RHC
R-HSA-51	Interleukin-3, Interle	0.003528333	0.016493	0.098961	HCK;SYK;GRB2;PTP
R-HSA-89	RUNX2 regulates ch	4.94E-04	0.017231	0.103384	RUNX2;GLI2
R-HSA-33	HSF1-dependent tra	0.004163432	0.017278	0.103667	HSPA8;HSP90AA1;H
R-HSA-89	Nucleotide salvage	0.004798532	0.017586	0.105517	CDA;UCK2;PNP;GM
R-HSA-21	Biological oxidation	0.038458824	0.018041	0.105899	SLC26A2;HSP90AB1;
R-HSA-33	Cellular response to	0.009526498	0.018594	0.105899	HSPA8;GSK3B;VCP;
R-HSA-11	Formation of apopto	9.17E-04	0.018648	0.105899	APAF1;XIAP;MAPK1
R-HSA-96	Regulation of the ap	9.17E-04	0.018648	0.105899	APAF1;XIAP;MAPK1
R-HSA-25	Signaling by Leptin	9.17E-04	0.018648	0.105899	LEP;LEPR;PTPN11;J
R-HSA-45	MAP kinase activati	0.004869099	0.019321	0.105899	MAPK10;MAPK8;JU
R-HSA-68	Regulation of TP53	0.003034366	0.019364	0.105899	CCNA2;PDPK1;AKT
R-HSA-24	DAP12 signaling	0.002469833	0.019702	0.105899	SYK;LCK;BTK;GRB
R-HSA-18	DARPP-32 events	0.002469833	0.019702	0.105899	PRKAR1A;PDE4D;PD
R-HSA-12	SHC1 events in ERE	0.001411333	0.020001	0.105899	ERBB4;GRB2;HRAS
R-HSA-68	TP53 Regulates Tra	0.001411333	0.020001	0.105899	APAF1;CASP1;TP53
R-HSA-30	Non-integrin membr	0.004304566	0.021134	0.105899	COL1A1;TGFB1;COL
R-HSA-24	Activation of gene e	0.004939666	0.02118	0.105899	FDPS;RXRA;SP1;SC5
R-HSA-88	Transcriptional regul	0.008326865	0.022223	0.111117	KAT2B;JAG1;TGFB1
R-HSA-90	NOTCH3 Intracellul	0.002540399	0.022518	0.11259	NOTCH3;KAT2B;ST
R-HSA-56	RAF activation	0.002540399	0.022518	0.11259	MAP2K1;SRC;BRAF;
R-HSA-20	Calcineurin activates	9.88E-04	0.023651	0.118253	FKBP1A;NFATC1;CA
R-HSA-18	Signaling by NTRK	0.006350999	0.023839	0.119196	SRC;BRAF;PIK3R1;M
R-HSA-89	RUNX3 regulates C	5.65E-04	0.02433	0.121651	SMAD3;TGFB1;TP53
R-HSA-56	Negative regulation	0.003175499	0.024486	0.12243	MAP2K1;PPP5C;MA
R-HSA-90	Signaling by NOTC	0.004445699	0.025581	0.127907	NOTCH3;KAT2B;JA
R-HSA-39	G beta:gamma signa	0.002046433	0.025753	0.128766	PDPK1;AKT2;AKT1;
R-HSA-16	Toll Like Receptor T	0.007832898	0.026878	0.134389	JUN;MAP2K1;MAP3
R-HSA-16	MyD88:MAL(TIRA	0.007832898	0.026878	0.134389	JUN;MAP2K1;MAP3
R-HSA-21	Tie2 Signaling	0.001552466	0.028523	0.142616	GRB2;PTPN11;PIK3R
R-HSA-46	Regulation of FZD b	0.001552466	0.028523	0.142616	USP8;WNT3A;FZD4;
R-HSA-15	Phase II - Conjugati	0.018206196	0.02885	0.144249	SLC26A2;GSTM2;AH
R-HSA-56	RHO GTPases Activ	0.002681533	0.028959	0.144794	RAC2;MAPK1;RAC1;
R-HSA-11	Apoptotic cleavage	0.002681533	0.028959	0.144794	CASP7;FNTA;CASP3
R-HSA-17	GRB2 events in EG	0.0010585	0.029393	0.146966	EGF;GRB2;HRAS;EG
R-HSA-37	DSCAM interaction	0.0010585	0.029393	0.146966	MAPK8;RAC1;MAPK
R-HSA-52	VEGFR2 mediated c	0.002117	0.029658	0.148292	PDPK1;SRC;KDR;CA
R-HSA-33	Regulation of HSF1-	0.007974031	0.030686	0.153429	HSPA8;GSK3B;NUP1
R-HSA-16	Toll Like Receptor 4	0.010161598	0.032065	0.160327	JUN;MAP2K1;MAP3
R-HSA-97	MyD88 cascade initi	0.006633265	0.032086	0.160432	JUN;MAP2K1;MAP3
R-HSA-16	Toll Like Receptor 1	0.006633265	0.032086	0.160432	JUN;MAP2K1;MAP3
R-HSA-16	Toll Like Receptor 5	0.006633265	0.032086	0.160432	JUN;MAP2K1;MAP3
R-HSA-16	Nef and signal trans	6.35E-04	0.032777	0.163886	HCK;LCK;RAC1
R-HSA-16	PKA activation in g	0.001623033	0.033528	0.16764	PRKAR1A;GNAS;PR
R-HSA-19	A tetrasaccharide lin	0.002187566	0.033933	0.169663	B3GAT3;B3GAT1;XY
R-HSA-43	Post NMDA recepto	0.006703832	0.034435	0.172176	PDPK1;SRC;ERBB4;
R-HSA-19	AKT phosphorylates	0.001129066	0.035891	0.179457	GSK3B;AKT2;MDM2
R-HSA-87	Advanced glycosylat	0.001129066	0.035891	0.179457	LGALS3;MAPK1;S10

R-HSA-165	Regulation of choles	0.006068732	0.036281	0.181406	FDPS;RXRA;SP1;SC5
R-HSA-448	Interleukin-17 signal	0.005433632	0.038024	0.190121	MAPK10;MAPK8;JUN
R-HSA-680	Regulation of TP53 I	2.82E-04	0.038315	0.191577	TP53
R-HSA-168	Toll Like Receptor 9	0.007550632	0.039624	0.191631	JUN;MAP2K1;MAP3K
R-HSA-885	Signaling by FGFR3	0.002893233	0.04078	0.191631	FGF17;FGF8;GRB2;PI
R-HSA-565	Signaling by FGFR3	0.002893233	0.04078	0.191631	FGF17;FGF8;GRB2;PI
R-HSA-557	Metabolic disorders	0.006209865	0.041863	0.191631	AHCY;MAOB;MAOA
R-HSA-885	MET activates PI3K	7.06E-04	0.042552	0.191631	GRB2;PIK3R1;MET
R-HSA-190	FGFR3b ligand bind	7.06E-04	0.042552	0.191631	FGF17;FGF8;FGF1
R-HSA-389	CD28 dependent Va	0.001199633	0.043154	0.191631	CDC42;LCK;GRB2;R
R-HSA-442	CREB1 phosphoryla	0.001199633	0.043154	0.191631	PRKAR1A;CALM3;C
R-HSA-678	IL-6-type cytokine re	0.001199633	0.043154	0.191631	IL11;LIFR;CLC;JAK2
R-HSA-567	TNF receptor superf	0.001199633	0.043154	0.191631	CD40LG;TNFSF11;TN
R-HSA-190	FGFR4 ligand bindir	0.001199633	0.043154	0.191631	FGF17;FGF8;FGF1;FG
R-HSA-183	Signaling by activate	0.001199633	0.043154	0.191631	FGF17;FGF8;FGF1;FG
R-HSA-887	RUNX1 and FOXP3	0.001199633	0.043154	0.191631	IFNG;IL2
R-HSA-975	Retinoid metabolism	0.005574765	0.044191	0.191631	RBP4;TTR;AKR1C1;A
R-HSA-679	TP53 Regulates Tran	0.006280432	0.044867	0.191631	JUN;CDK7;CTDP1;CC
R-HSA-392	EPHB-mediated forw	0.003598899	0.045358	0.191631	CDC42;SRC;LIMK1;R
R-HSA-701	Glycolysis	0.007762332	0.047723	0.191631	PFKFB1;GPI;NUP107
R-HSA-703	Glucose metabolism	0.009949898	0.047908	0.191631	PFKFB1;GPI;NUP107
R-HSA-201	TCF dependent signa	0.015242396	0.048372	0.193488	GSK3B;USP8;CSNK2
R-HSA-217	DAP12 interactions	0.003669466	0.049707	0.198826	SYK;LCK;BTK;GRB2

;PIK3R1;TNF;CCND1;PIM1;AKT1;NDN;HMOX1;IL12A;CD36;JAK2;JAK3;IL10;HSPA8;HSP90AA1
;PITPNA;RORA;FGF1;TNF;FGF8;CCND1;AKT2;PIM1;AKT1;TNFSF11;IL12A;PRKACA;MAP2K1;A
2;TCIRG1;FGF1;IGF1R;FGF8;AKT2;CHEK1;KDR;AKT1;RAC1;PRKACA;JAK2;PRKACB;HRAS;U
GFR;INS;FGF8;ERBB4;AKT2;RAC2;MAPK1;AKT1;RAC1;FGF23;MAPK3;KL;EGF;INSR;PTPN11;E
GFR;INS;FGF8;ERBB4;RAC2;MAPK1;AKT1;RAC1;FGF23;MAPK3;KL;EGF;INSR;PTPN11;ESR1;ES
PITPNA;AIP;MIF;SOD2;CDC42;MTAP;CA1;VAMP7;IFNG;CA2;IL12A;JAK2;IL12RB1;PPIA
R1H2;NR1I2;NR1H4;NR1H3;RORA;NR3C1;ESR1;NR3C2;NR5A1;AR;RXRA;RARA;PGR;PPARG;PF

PLOD2;PLOD1;CTSV;ITGAL;CTSS;ADAMTS4;EFEMP2;ADAMTS2;IBSP;CASP3;CTSK;KDR;CTS
;CTSV;CTSS;ADAMTS4;CTSK;CASP3;SPP1;CTSG;CTSD;ELANE;CTSB;MMP7;LAMB3;MME;CM
3B;PTPN11;PIK3R1;FGF1;INS;FGF17;FGF8;AKT2;MAPK1;GRB2;HRAS;FGF23;FGFR2;MAPK3;FC
F2;PTPN11;PIK3R1;IGF1;FGF1;IGF1R;FGF17;FGF8;AKT2;GRB2;HRAS;FGF23;FGFR2;FGFR1
NAI2;IGF1R;CCND1;AKT2;MAPK1;AKT1;HRAS;MAPK3;HSP90AA1;MMP7;PDPK1;NOS3;MMP1;
F2;PTPN11;PIK3R1;IGF1;FGF1;IGF1R;FGF17;FGF8;AKT2;GRB2;HRAS;FGF23;FGFR2;FGFR1
F2;PTPN11;PIK3R1;IGF1;FGF1;IGF1R;FGF17;FGF8;AKT2;GRB2;HRAS;FGF23;FGFR2;FGFR1

K3CG;RAC2;AKT1;RAC1;CD36;CTSF;PRKACA;SYK;PDPK1;AHSG;FGG;ANXA5;GP1BA;F2;RHO
F1;HSD17B11;BMP15;SPP1;CTSG;APOE;FGF23;MEN1;IGFBP1;IGFBP5;CMA1;AHSG;MMP1;IGF

SERPINA1;HEXB;HP;HBB;TCIRG1;PYGL;ITGAL;CTSS;FCAR;ALAD;LGALS3;PNP;GM2A;PLAU;

MMP2;MMP3;CTSV;MMP8;MMP9;COL1A1;MMP12;ADAM17;MMP14;COL2A1;COL1A2;MMP13;
3B;PTPN11;TCIRG1;PIK3R1;FGF1;INS;FGF17;FGF8;AKT2;MAPK1;GRB2;HRAS;FGF23;FGFR2;M

A;NR3C1;NR3C2;LGALS3;CCND1;AKT2;CHEK1;AKT1;SOX9;NBN;PRKACA;GGT1;MEN1;IGFBI
11;PIK3R1;FGF1;FGF17;FGF8;FLRT3;ANOS1;MAPK1;GRB2;HRAS;FGF23;MAPK3;FGFR1
3;MMP1;LAMA3;MMP3;P3H1;LAMC2;PLOD2;PLOD1;CTSV;MMP9;CTSS;COL1A1;ADAMTS2;B

FBXW7;SATB2;NR3C1;ESR1;RUNX2;GLI2;COL1A1;AR;LGALS3;BMP2;MMP13;CCND1;BGLAP;A

K3R1;FGF1;EGFR;INS;FGF8;ERBB4;AKT2;RAC2;MAPK1;AKT1;RAC1;PRKACA;PRKACB;FGF23
K3R1;FGF1;EGFR;INS;FGF8;ERBB4;AKT2;RAC2;MAPK1;AKT1;RAC1;FGF23;MAPK3;KL;JUN;CS

RC;PIK3R1;EGFR;GNAI2;IGF1R;CCND1;ERBB4;AKT2;MAPK1;AKT1;CTSD;HRAS;MAPK3;JUN;H

K3R1;FGF1;EGFR;FGF8;ERBB4;AKT2;CASP3;MAPK1;CSK;JAK2;JAK3;HRAS;FGF23;MAPK3;KL;
OS3;SRC;PIK3R1;MAPK14;PTK2;RHOA;MAPK12;CDC42;AKT2;MAPKAPK2;KDR;CTNNA1;PTK2
AKT2;KDR;CTNNA1;PTK2B;AKT1;RAC1;PRKACA;PRKACB;HRAS;HSP90AA1;PDPK1;NOS3;MA

42;FGF8;ERBB4;MAPK1;CSK;RAC1;PRKACA;JAK2;PRKACB;JAK3;HRAS;FGF23;MAPK3;KL;JU

8;ERBB4;MAPK1;CSK;JAK2;JAK3;HRAS;FGF23;MAPK3;KL;MAP2K1;EGF;FGG;BRAF;PTPN11;I

SERPINA1;PSAP;FLNA;CD36;CTSF;PRKACA;TGFB2;TGFB1;AHSG;EGF;FGG;ANXA5;IGF2;IGF1

8;ERBB4;MAPK1;CSK;JAK2;JAK3;HRAS;FGF23;MAPK3;KL;MAP2K1;EGF;FGG;BRAF;IL17RD;

TGFB1;SPARC;SERPINA1;AHSG;EGF;ANXA5;FGG;IGF2;IGF1;SELP;TF;ALB;PSAP;FLNA;CALM

D17B4;HMGCR;HSD17B11;CYP19A1;CYP17A1;HSD11B1;CYP27B1;RXRA;CYP11A1;HSD17B1;S

D17B11;CYP19A1;CYP17A1;HSD11B1;POMC;STAR;CYP21A2;CYP11A1;HSD17B1;CYP11B1;CG

107;STAT1;PTPN11;HLA-A;AAAS;ISG20;IFNG;FLNA;MAPK1;FLNB;IRF5;JAK2;EIF4E;HLA-DQA

1;FGG;PTPN11;CTSV;FOS;MAPK14;CTSS;DUSP6;EEA1;MAPK10;MAPK8;CTSK;TRAF6;MAPK

RAF;PAPSS1;LMNA;MAPK1;CSK;CALM3;RAF1;CALM1;JAK2;CALM2;HRAS;MAPK3

K1;FOS;MAPK14;DUSP6;MAPK10;MAPK8;TRAF6;MAPKAPK2;PSAP;BTK;MAPK1;CD36;S100A9

K1;FOS;MAPK14;DUSP6;MAPK10;MAPK8;TRAF6;MAPKAPK2;PSAP;BTK;MAPK1;CD36;S100A9

IK3R1;MAPK14;DUSP6;RHOA;MAPK12;ADORA2A;MAPKAPK2;MAPK1;GRB2;RAC1;HRAS;CD

AHCY;ADH1C;MAOB;ADH1B;MAOA;GSTP1;GSTT2;CYP3A4;CYP19A1;PAPSS1;SULT1A1;CYP2
HSP90AA1;NUP107;HSP90AB1;AAAS;MAPKAPK2;BAG1;SERPINH1;MAPK1;HSPA1A;MAPK3

IAPK14;DUSP6;RHOA;MAPK12;ADORA2A;MAPKAPK2;MAPK1;GRB2;HRAS;MAPK3

K1;FOS;MAPK14;DUSP6;MAPK10;MAPK8;TRAF6;MAPKAPK2;BTK;MAPK1;CD36;S100A9;MAPI
K1;FOS;MAPK14;DUSP6;MAPK10;MAPK8;TRAF6;MAPKAPK2;BTK;MAPK1;CD36;S100A9;MAPI

CY;GSTM1;GSTO1;GSTP1;GSTT2;UGT2B17;PAPSS1;SULT1A1;SULT2B1;GSTZ1;HPGDS;SULT1

K1;PTPN11;FOS;MAPK14;DUSP6;MAPK10;MAPK8;TRAF6;MAPKAPK2;PSAP;BTK;MAPK1;CD36
K1;FOS;MAPK14;DUSP6;MAPK10;MAPK8;TRAF6;MAPKAPK2;MAPK1;S100A9;MAPK3
K1;FOS;MAPK14;DUSP6;MAPK10;MAPK8;TRAF6;MAPKAPK2;MAPK1;S100A9;MAPK3
K1;FOS;MAPK14;DUSP6;MAPK10;MAPK8;TRAF6;MAPKAPK2;MAPK1;S100A9;MAPK3

K1;FOS;MAPK14;DUSP6;EEA1;MAPK10;MAPK8;TRAF6;MAPKAPK2;MAPK1;S100A9;MAPK3

1;CYP19A1;CYP17A1;POMC;CYP27A1;CYP27B1;CYP24A1;CYP21A2;CYP11A1;CYP11B1;GGT1

;G6PC;TPI1;PKLR;GK;AAAS;GCK;HK1;GNPDA1;GNPDA2;ALDOA;PRKACA;SLC37A4;PCK1;PR
A1;WNT3A;FZD4;LRP5;XIAP;DKK1;CDC73;LRP6;SOX3;SFRP1;TERT;AKT2;AKT1;CTNNB1;TCF

.;TGFB1;NOS2;MMP1;STAT1;MMP2;MMP3;FOS;MMP9;POMC;IL1A;IL6;COL1A2;IL1B;LCN2;TP5
AIP;LIFR;HLA-A;MIF;PRLR;MTAP;ADAM17;MAPKAPK2;RAF1;PPIA;TP53;S100A9;CES1;MAOB;I
JSP8;HSP90AA1;PDPK1;MST1;MMP9;PGF;DUSP6;RHOA;ADAM17;COL2A1;ADORA2A;LCK;MA

G;PRKACA;CTSD;CTSB;MMP7;MME;MMP1;MMP2;FGG;MMP3;P3H1;MMP8;MMP9;HSPG2;MM
IA1;MMP1;MMP2;MMP3;MMP8;MMP9;HSPG2;COL1A1;MMP12;MMP14;ADAM17;COL2A1;COL

)A;LCK;PRKCQ;ALDOA;RAF1;PPIA;CFD;CANT1;SRC;PIK3R1;GNAI2;CDC42;PSAP;MAPK1;FLN
BP4;MMP2;IGFBP3;MMP3;FGG;IGFBP2;APOA2;IGF2;MEPE;IGF1;F2;CP;BMP4;TF;IL6;ALB;FBN1

CTSG;RAC1;CD36;CTSD;CTSB;ARSA;HSP90AA1;MME;APAF1;AHSG;ARG1;NME2;HLA-A;MIF;

P1;G6PC;CSNK2A1;FBXW7;IGFBP3;LIFR;RUNX2;AR;DDX39B;PGR;PRKCQ;TP53;NOTCH2;NOT(

;MAPK3;KL;JUN;CSNK2A1;PDPK1;EGF;INSR;PTPN11;ESR1;ESR2;FGF17;RHEB;LCK;PRKAR1A;
SNK2A1;PDPK1;EGF;INSR;PTPN11;ESR1;ESR2;FGF17;RHEB;LCK;TRAF6;KIT;MDM2;GRB2;PPA

ISP90AA1;MMP7;PDPK1;NOS3;MMP1;EGF;MMP2;MMP3;FOS;ESR1;MMP9;PTK2;ESR2;KAT2B;I

MAP2K1;PDPK1;EGF;FGG;BRAF;PTPN11;IL17RD;PTK2;DUSP6;IL2;FGF17;HCK;PPP5C;KIT;GRE

JN;MAP2K1;EGF;MMP2;FGG;BRAF;PTPN11;IL17RD;PTK2;DUSP6;IL2;FGF17;IL6;PPP5C;KIT;GR

L17RD;PTK2;DUSP6;IL2;FGF17;IL6;PPP5C;KIT;GRB2;CALM3;TEK;CALM1;RAF1;CALM2;MET;[illegible]

[illegible]TK2;DUSP6;IL2;FGF17;PPP5C;KIT;GRB2;CALM3;TEK;CALM1;RAF1;CALM2;MET;FGFR2;FGFR[illegible]

[illegible]C5D;CGA;GC;FDPS;HSD3B7;HSD3B2;VDR;AKR1C1;NR1H4;AKR1C3;AKR1C2;LSS;POMC;CYP2

27B1;HPGDS;RXRA;ALDH2;SULT1E1;CYP11A1;GGT1;SULT2A1;CBR1;GSTM2;NQO2;GSTM1;GS

MAOA;TNFRSF11B;PIK3R1;TNFRSF11A;AAAS;NDN;HMOX1;FGF23;HLA-DQA1;KL;HSPA8;JUN
PKAPK2;KIT;ANOS1;PGR;MET;SRC;LAMA3;HPN;PIK3R1;EGFR;INS;CDC42;FLRT3;ERBB4;SPP1

P12;MMP14;ADAM17;COL2A1;MMP13;LOX;MMP16;PPIB;LAMA3;FBLN5;TTR;SERPINH1;SPP1;

A;CSK;MAPK3;PTPN1;TGFB2;TGFB1;EGF;IGF2;PTPN11;IGF1;MAPK14;PTK2;SELP;COL1A1;TF;

RNASE3;MMP8;RNASE2;MMP9;RHOA;APRT;CHIT1;SLPI;CAT;ALDOA;ACPP;PPIA;S100A9;CFD

CH3;GATA4;AAAS;ABL1;APOE;CGA;PCK1;JUN;TGFB1;JAG1;SMAD3;VDR;NR0B1;ESRRG;ESR1

STO1;AIP;NR1H4;UGT2B17;SULT2B1;POMC;CYP27A1;GSTZ1;CYP2C9;POR;CYP2C8;CYP24A1;C

;TGFB1;SMAD3;TNFSF15;BRAF;IL2;PTK2;FGF17;ISG20;GH1;PPP5C;IL6;COL1A2;IL7;LCN2;GRE
1;CTNNA1;MAPK1;PTK2B;CSK;APOE;FGF23;MAPK3;WWOX;PTPN1;KL;LAMB3;NOS3;STAT1;C

ELANE;CRTAP;TGFB2;TGFB1;LAMB3;CMA1;BMP7;BMP4;COL1A1;BMP2;BMP1;COL1A2;COL7

;ASAH1;VCP;CANT1;GSTP1;TTR;TTPA;PSAP;MAPK1;LTA4H;ELANE;HSPA8;CMA1;MAPK14;L

;IL2;ESR2;COL1A1;BMP2;CDK7;CDK6;SP1;RHEB;CDK2;MDM2;MKRN3;TCF4;CALM3;SP7;CAL

32;CALM3;CALM1;CALM2;FGFR2;BCL2L1;FGFR1;PDE3B;PRL;GHR;CA1;CA2;CASP3;CASP1;CT
CMA1;EGF;INSR;IGF2;BRAF;PTPN11;IGF1;MAPK14;ESR1;PTK2;MAPK12;FGF17;COL1A1;COL1A

M1;CALM2;GPI;GSK3B;RARG;THRB;THRA;HNF4G;CTSV;ITGAL;CDC73;GLI2;CTSK;CASP1;RN

SG;CD36;JAK2;JAK3;HRAS;IL10;IL11;HSP90AA1;SYK;GSTO1;PDPK1;MMP1;MMP2;FGG;MMP3

NU4ATAC;HSP90AA1;PARP1;APAF1;PDPK1;RFC2;GP1BA;FOS;PAX5;BAZ1B;NR5A1;CCNA2;KA

;FOS;IL17RD;MMP9;DUSP6;HCK;IL1A;VAMP7;IFNG;LCK;TRAF6;IL1B;KIT;IRF5;MET;HLA-DQ[illegible]

T2B;MMP13;IFNG;CAT;KIT;CRH;RARA;RARB;PPARG;PPARA;MET;PPARD;CTDP1;SRC;NR1I3;[illegible]

[illegible]1;RNASEL;SRC;EGFR;CDC42;MAPK8;ERBB4;CCL5;FLNA;MAPK1;PTK2B;FLNB;CSK;IL12RB1;

SATB2;NR1I2;HDAC8;EGFR;AURKA;INS;RXRB;WRN;RXRA;BGLAP;SPP1;PMS2;MAPK1;MAPK

EIF4E;MAPK3;PTPN1;NOS2;STAT1;CMA1;EGF;PTPN11;SOD2;MAPK14;POMC;MAPK10;CD40L

3;PTPN1;WWOX;CAVIN1;GK;STAT1;NR1H2;GSR;NR1H4;NR1H3;PTPN11;SOD2;MAPK14;GCK;

POMC;ERCC2;CTNNB1;ERCC6;RAN;CDK5R1
