# Supplementary material for: Research on the Mechanism of Liuwei Dihuang Decoction for Osteoporosis Based on Systematic Biological Strategies
Source: Evid Based Complement Alternat Med. 2022 Sep 22;2022:7017610. doi: 10.1155/2022/7017610 (PMC9522519; doi:10.1155/2022/7017610)
Supplement: Supplementary Materials — Table S1-1: components meeting the screening criteria. Table S1-2: compound targets for each compound of LDD. Table S2: osteoporosis genes. Table S3: enrichment analysis of clusters based on Gene Ontology (GO) annotation of LDD-osteoporosis PPI network. Table S4: pathway enrichment analysis of LDD-osteoporosis PPI network. Table S5: Reactome pathways of LDD-osteoporosis PPI network. Table S6: Human Transcriptomics Data. Table S7: the biological processes of Human Transcriptomics Data Network. Table S8: the Reactome pathways of Human Transcriptomics Data Network. Table S9: the signaling pathways of Human Transcriptomics Data Network. Table S10: the biological processes of protein arrays data network. Table S11: the Reactome pathways of protein arrays data network. Table S12: the signaling pathways of protein arrays data network. [file 7017610.f1.zip › 7017610.f1/Table S6.pdf]

TableS6 Human Transcriptomics Data

GeneSymbol	PValue	t	B	log2FC
NKTR	1.31E-07	22.54093	8.18569	7.243047
CEP290	4.06E-07	19.02915	7.262111	7.170359
GCC2	3.66E-08	27.2844	9.118515	6.969286
C7orf73	3.27E-08	27.75672	9.196134	6.814075
TRERF1	2.37E-08	29.11117	9.405991	6.762752
KIAA1033	1.12E-05	11.47489	4.127947	6.747274
SRP9	2.02E-07	21.12609	7.842051	6.684027
KRAS	2.23E-06	14.70915	5.72044	6.659171
HSP90AA1	9.87E-08	23.52863	8.405804	6.638143
DCK	1.73E-07	21.62868	7.968168	6.524954
SYNE2	6.05E-05	8.812674	2.384682	6.444967
SETD2	3.96E-07	19.10251	7.283987	6.432161
KIAA1551	1.30E-06	15.96888	6.227478	6.363694
USP37	1.76E-05	10.69984	3.668557	6.343967
CCDC88A	7.06E-05	8.596325	2.220175	6.303104
CCDC88A	3.35E-05	9.677258	3.004654	6.28571
STRN3	1.23E-06	16.09714	6.276159	6.254585
CCDC88A	4.28E-05	9.308093	2.746988	6.239854
PTBP1	2.22E-07	20.83041	7.76566	6.235063
CCDC88A	2.39E-05	10.20615	3.356763	6.214463
DDX17	4.13E-07	18.98162	7.247873	6.196647
CCDC88A	1.99E-05	10.49779	3.542841	6.191309
USP47	2.27E-08	29.29611	9.433258	6.179731
CCDC88A	4.64E-05	9.192059	2.663873	6.177031
CCDC88A	2.84E-05	9.929297	3.174873	6.175878
HSP90AA4P	2.27E-06	14.6648	5.701576	6.171207
TPM1	6.60E-06	12.45658	4.662348	6.151335
XAF1	4.82E-06	13.07191	4.973039	6.138985
TNRC6B	8.27E-05	8.381899	2.053168	6.128786
HSP90AA1	4.64E-05	9.192249	2.664009	6.108545
CCDC88A	3.91E-05	9.443274	2.84252	6.102961
PTGES3	6.93E-06	12.36357	4.613824	6.089804
ATP8B5P	9.48E-08	23.66994	8.436066	6.072678
CCDC88A	3.31E-05	9.693201	3.015556	6.05753
BOD1L1	1.39E-07	22.35506	8.142518	6.003275
UBR3	5.29E-08	25.82436	8.862702	6.002347
IKZF2	2.52E-06	14.43352	5.602037	5.99019
CCDC88A	2.90E-05	9.896146	3.15274	5.985485
ITM2B	2.44E-07	20.54126	7.689329	5.923956
NUTM2B-AS1	1.60E-06	15.46335	6.030459	5.901967
SMG1	3.11E-08	27.95592	9.22816	5.900477
RHOQ	2.94E-06	14.0981	5.454068	5.866456
ZFC3H1	6.29E-07	17.81514	6.881482	5.843224
BDP1	1.12E-06	16.32899	6.362862	5.840869
JAK1	1.21E-07	22.80938	8.247044	5.811572
HIST1H4B	6.17E-05	8.785205	2.364013	5.774644

VMP1	1.11E-05	11.50179	4.143272	5.765917
LUC7L3	2.54E-07	20.41885	7.656518	5.759512
THAP9-AS1	5.71E-08	25.53503	8.808982	5.726246
ANKRD36B	3.85E-05	9.466648	2.858899	5.699225
TRIM14	2.61E-05	10.06079	3.261912	5.685968
FAM53C	3.36E-07	19.57969	7.423329	5.681742
CEP97	1.31E-07	22.55367	8.188629	5.673357
CEP104	4.57E-08	26.39719	8.966035	5.65534
MAP3K1	2.00E-05	10.49464	3.54086	5.652148
ATP13A3	6.08E-06	12.61631	4.744705	5.641332
C3orf58	6.07E-08	25.30098	8.76475	5.639151
TRAF7	4.02E-07	19.05452	7.269692	5.618657
FAM8A1	4.69E-08	26.29682	8.948213	5.611546
ZMYM2	2.69E-05	10.0166	3.232794	5.603066
ZBED5	3.99E-08	26.93749	9.059954	5.572413
COX11	5.56E-06	12.79097	4.833374	5.558004
ANKRD36BP2	1.10E-05	11.5135	4.149932	5.530498
RNF166	1.20E-06	16.15476	6.297862	5.523291
XAF1	2.93E-07	19.98055	7.536554	5.51221
SF1	3.21E-04	6.713601	0.602333	5.472161
RPN1	1.60E-06	15.4739	6.034657	5.471822
CENPK	3.88E-05	9.453749	2.849865	5.470126
ZNF652	8.22E-07	17.10757	6.642285	5.454297
RRM2	3.64E-07	19.34116	7.354309	5.448482
LOC105370109	1.33E-07	22.50071	8.176398	5.447736
ERC1	1.34E-06	15.8795	6.193246	5.444865
SRRM1	2.04E-05	10.46127	3.519841	5.443964
SPEN	2.13E-05	10.38771	3.473259	5.423788
HOTAIRM1	1.68E-07	21.72862	7.992702	5.421445
GOLGA4	1.09E-07	23.19356	8.332844	5.416861
HIST1H4L	8.16E-07	17.12552	6.648524	5.413758
SLX4IP	2.29E-07	20.74133	7.742318	5.406445
SCAF11	3.09E-07	19.82216	7.492225	5.393338
AGGF1	7.46E-06	12.22396	4.540186	5.388297
C19orf43	4.98E-08	26.05953	8.905603	5.375484
CD300A	7.69E-07	17.28238	6.702643	5.37167
SCAMP1	2.84E-05	9.930508	3.175681	5.362632
CTAGE7P	2.60E-06	14.367	5.573039	5.360585
REEP3	1.32E-06	15.93009	6.212655	5.337706
PPIAP30	2.87E-07	20.04246	7.553738	5.332224
LRP5L	2.11E-07	20.99177	7.807554	5.320463
RBMX	3.15E-06	13.95463	5.389434	5.317292
BOD1L1	3.83E-07	19.19284	7.310757	5.299448
SUMO2	1.82E-07	21.46633	7.927934	5.265287
SAMD9	1.77E-05	10.69431	3.665152	5.25295
HADHA	1.45E-04	7.657184	1.457612	5.240698
CRKL	1.32E-06	15.92394	6.2103	5.240102
PPIAL4A	5.43E-07	18.21182	7.009833	5.227576

SMCHD1	2.24E-06	14.69467	5.714289	5.212271
SF3A1	2.00E-07	21.17081	7.853461	5.208763
MBNL2	1.00E-07	23.47348	8.393914	5.20679
RBM27	3.51E-06	13.72151	5.282647	5.205521
RPL21	1.34E-07	22.46913	8.169082	5.188601
CPED1	3.71E-05	9.523077	2.898272	5.181575
SMC5	1.37E-07	22.40017	8.153049	5.178403
KIAA0319L	1.59E-06	15.48352	6.038482	5.173324
HCG18	2.92E-06	14.11244	5.460483	5.171494
N4BP2	1.98E-05	10.50486	3.547287	5.149923
ZNF319	6.16E-07	17.8697	6.899372	5.149586
RAB11FIP2	2.35E-07	20.65662	7.719979	5.143256
KRIT1	8.04E-06	12.0848	4.465813	5.139304
RPS7	3.41E-06	13.78578	5.31231	5.133353
SCAF11	5.09E-06	12.96233	4.918992	5.131278
RPL7	5.05E-07	18.41187	7.073068	5.102733
SNTB1	4.36E-08	26.58509	8.99908	5.100422
ATP5E	4.95E-07	18.47024	7.091332	5.089612
MDM1	4.34E-06	13.28247	5.075392	5.082444
GALNT3	6.65E-07	17.66363	6.831388	5.080888
ANKRD12	3.03E-06	14.03545	5.425946	5.075596
APOH	6.96E-04	5.884685	-0.2344	5.074375
PEX13	5.25E-06	12.9026	4.8893	5.060363
ZNF516	1.65E-05	10.81174	3.737091	5.059592
NIPBL	4.62E-07	18.66095	7.150436	5.053087
SEC63	1.29E-06	15.9718	6.228593	5.048372
CD44	1.28E-07	22.62781	8.205675	5.040941
CEP170	3.00E-05	9.848476	3.120779	5.038758
REST	9.06E-06	11.86399	4.345771	5.038302
BCL11A	6.07E-06	12.61874	4.745951	5.029473
SYNE2	2.99E-04	6.790802	0.676009	5.025078
EIF1AY	2.29E-07	20.73532	7.740737	5.016286
CTAGE5	5.54E-07	18.15948	6.993126	5.013095
RBM43	4.63E-07	18.65252	7.147843	5.010325
PLEKHA2	3.24E-06	13.88945	5.359802	5.008016
ZNF800	2.15E-06	14.78508	5.752563	5.007079
SEC63	3.39E-06	13.7968	5.317379	5.004584
TCP1	9.98E-07	16.61319	6.466915	4.980324
RPL9	8.95E-07	16.88917	6.565676	4.961519
AR	6.73E-05	8.662411	2.270845	4.950997
SKIL	1.41E-06	15.77127	6.151453	4.945017
SNX2	8.52E-08	24.05233	8.516472	4.944236
TMEM109	1.44E-07	22.2288	8.112863	4.934574
ALB	6.12E-04	6.016108	-0.09594	4.931473
PDP1	2.78E-06	14.22127	5.50891	4.915343
NIN	1.07E-07	23.22996	8.340854	4.910657
PHACTR2	6.90E-08	24.82354	8.672315	4.908901
CASC4	9.85E-06	11.71226	4.261799	4.900678

CAPN10	1.62E-05	10.84535	3.757523	4.893134
ZNF654	2.34E-05	10.23896	3.377978	4.885643
ANKRD12	1.01E-05	11.67023	4.238327	4.880554
PCM1	2.38E-07	20.61306	7.708436	4.879961
FUBP1	9.20E-07	16.81975	6.541042	4.876067
GMCL1	1.31E-05	11.21118	3.97557	4.86955
MYSM1	3.54E-07	19.42258	7.378009	4.853215
IBTK	2.35E-06	14.59209	5.6705	4.848177
MIS18BP1	3.98E-06	13.4596	5.159996	4.840848
BAG4	8.33E-07	17.0725	6.630074	4.832373
BBX	9.42E-07	16.75906	6.519391	4.832179
CCDC180	1.64E-06	15.41378	6.010685	4.811595
LCORL	5.37E-05	8.981161	2.510102	4.804658
RIC3	2.13E-05	10.38648	3.472478	4.802563
LIMS1	1.10E-06	16.36803	6.377299	4.796069
NOL8	1.21E-06	16.13049	6.288733	4.780583
ZNF814	4.64E-07	18.6469	7.146111	4.779576
AR	1.56E-05	10.90393	3.792974	4.77885
AR	5.72E-05	8.890388	2.44282	4.76831
EP300	1.14E-06	16.27798	6.343928	4.764012
FRG1BP	7.58E-04	5.797111	-0.32793	4.758642
SUMO1P3	2.39E-05	10.20225	3.354241	4.758306
C8orf59	3.78E-06	13.5672	5.210731	4.7574
THAP6	6.56E-06	12.47011	4.669372	4.754081
HLTF	1.29E-04	7.79715	1.576541	4.75384
CHURC1	1.54E-07	22.02018	8.06327	4.752126
EP300	5.69E-07	18.08386	6.968866	4.74576
SVIP	1.87E-06	15.10597	5.886038	4.744293
CABIN1	7.28E-07	17.42598	6.751595	4.734684
GPCPD1	6.92E-07	17.55786	6.796062	4.721767
LINC00662	4.89E-06	13.0423	4.95849	4.718675
ELF1	3.63E-05	9.556217	2.921285	4.71342
PLAA	8.96E-07	16.88534	6.564321	4.713208
BTN2A1	7.66E-07	17.29046	6.705412	4.708921
ANKRD11	2.65E-07	20.29411	7.622777	4.703334
DZIP3	2.65E-06	14.3213	5.553015	4.700274
CNTRL	2.25E-07	20.79679	7.756868	4.699675
UBE2V2	1.61E-05	10.85579	3.763855	4.696467
TPM1	2.27E-05	10.28636	3.408494	4.687479
EP300	4.73E-07	18.59475	7.130018	4.686206
RPRD1A	7.39E-06	12.24278	4.550166	4.681547
PWWP2A	1.10E-06	16.3652	6.376255	4.68118
P2RY13	5.44E-05	8.961693	2.495729	4.679882
GAPVD1	1.76E-05	10.70662	3.67273	4.679485
DYRK2	1.25E-04	7.840944	1.613356	4.676616
CHD9	1.55E-06	15.54813	6.064083	4.674857
LARP1B	4.17E-06	13.36572	5.115322	4.666201
DPY19L1	5.96E-06	12.65357	4.76374	4.634766

IFIT2	2.66E-07	20.27289	7.617005	4.633359
AR	5.95E-06	12.65604	4.764999	4.625257
AR	5.03E-05	9.075342	2.579208	4.624137
SELL	1.37E-05	11.13326	3.929788	4.620057
UFM1	7.92E-05	8.440785	2.099433	4.616883
HIGD1A	7.61E-07	17.30729	6.711174	4.614136
ZNF644	2.72E-06	14.2643	5.527929	4.613828
TMEM129	3.90E-05	9.449063	2.846581	4.608886
MAP3K1	1.11E-04	7.992804	1.739586	4.608698
EP300	6.77E-07	17.61868	6.816412	4.608245
PPIAL4A	3.35E-07	19.58575	7.425066	4.604071
TPM3	2.96E-06	14.08797	5.449535	4.601984
EP300	1.02E-06	16.56025	6.447714	4.59976
CCDC186	3.35E-06	13.8208	5.328403	4.589507
EP300	2.42E-06	14.52186	5.640293	4.588252
RSF1	3.93E-06	13.4891	5.173952	4.58793
ITSN2	1.30E-05	11.21909	3.980199	4.586588
SRGAP2C	7.34E-05	8.542894	2.178935	4.583002
COX5A	7.64E-05	8.489782	2.137695	4.581913
CTSS	3.25E-06	13.88408	5.357351	4.575903
EP300	8.83E-07	16.92276	6.577546	4.570705
EP300	1.14E-06	16.29284	6.349452	4.570248
DNAJB14	4.47E-06	13.22381	5.047076	4.5672
AR	3.11E-05	9.792375	3.082959	4.566575
TRMT13	2.77E-05	9.969744	3.201774	4.56546
HNRNPCL1	4.21E-06	13.34647	5.106118	4.555939
MRI1	3.98E-06	13.46111	5.16071	4.545612
GALNT4	3.33E-06	13.83568	5.335222	4.54296
NCOA3	4.10E-06	13.40004	5.131696	4.542761
CARF	1.37E-06	15.83441	6.175882	4.539707
DDX24	2.49E-06	14.4581	5.612711	4.539364
YTHDC2	8.01E-07	17.17622	6.666091	4.536734
AMD1P3	1.72E-05	10.73742	3.691658	4.532467
FAM133CP	5.65E-06	12.75916	4.817334	4.532412
EP300	5.53E-07	18.16072	6.993522	4.530783
ARRDC3	1.16E-06	16.2493	6.333247	4.527797
CHCHD2	4.28E-07	18.87746	7.216488	4.521294
C18orf32	8.08E-07	17.15103	6.657369	4.51337
EIF3F	9.58E-06	11.76244	4.289708	4.511192
PNPLA8	3.07E-07	19.84847	7.499625	4.504479
CBWD5	2.87E-06	14.15004	5.477267	4.504382
SEPT7P2	1.45E-05	11.02849	3.86767	4.476801
HM13	1.90E-04	7.321396	1.164212	4.475016
KLF13	7.81E-05	8.458828	2.113548	4.467036
BCL7B	5.17E-07	18.34624	7.052431	4.462749
GNAQ	3.92E-06	13.49145	5.175062	4.461334
ZC3H6	4.12E-06	13.39222	5.127973	4.452693
CYLD	3.04E-05	9.826877	3.106245	4.451653

MIER1	5.67E-06	12.75145	4.813439	4.450971
MBNL1	4.54E-06	13.1924	5.03185	4.44883
COMMD6	6.32E-06	12.53986	4.705442	4.439559
TAGLN3	1.00E-06	16.60902	6.465404	4.436455
LINC01000	9.18E-07	16.82572	6.543167	4.435629
CCDC91	1.42E-05	11.06285	3.888114	4.435479
SEC62	8.48E-07	17.02659	6.614039	4.431828
RIF1	1.04E-05	11.60826	4.203532	4.430129
MAP4K3	4.30E-05	9.301513	2.742303	4.42379
RPL21	3.04E-07	19.87422	7.506854	4.42135
ATRX	5.90E-06	12.67198	4.773121	4.419643
PACS1	1.43E-05	11.05341	3.882505	4.412749
MYO5A	2.04E-06	14.90178	5.801529	4.412105
CCDC112	8.03E-07	17.16907	6.663616	4.408878
AR	1.80E-05	10.6673	3.648488	4.405419
GTF2IP20	1.22E-07	22.79779	8.24442	4.39985
FAM133B	1.02E-06	16.54722	6.442978	4.398669
SUMO2	1.73E-04	7.434665	1.264484	4.395599
DNAJB6	3.48E-06	13.74144	5.291864	4.395372
TRUB1	3.34E-07	19.5967	7.428205	4.393746
VPS13A	6.41E-07	17.76286	6.864265	4.384148
RBM25	2.80E-06	14.2035	5.501037	4.3827
KIF1B	6.99E-07	17.53215	6.787431	4.380885
AR	2.90E-05	9.900302	3.155519	4.38038
EXOC5	8.07E-05	8.414298	2.078661	4.357962
CCR6	6.95E-05	8.61801	2.236843	4.350463
LOC100507131	5.34E-06	12.86766	4.871859	4.350105
NCOA3	2.40E-05	10.19763	3.351246	4.349751
PRRC2C	2.03E-05	10.46363	3.52133	4.349628
NBPF9	8.88E-06	11.90119	4.366171	4.348037
SP3P	2.29E-05	10.26893	3.397289	4.347215
TRIM23	2.49E-06	14.46456	5.615516	4.344891
TPT1	1.76E-06	15.24701	5.943555	4.343999
SPPL3	3.16E-07	19.76369	7.475726	4.342174
BDP1	1.47E-05	11.01119	3.857353	4.340068
SECISBP2L	2.89E-05	9.905851	3.159228	4.334392
RPL17	1.62E-06	15.44153	6.021765	4.334124
ZNF675	3.01E-07	19.89962	7.51397	4.332954
EXOC5	1.95E-07	21.24707	7.872834	4.332011
NPHP4	5.61E-05	8.917162	2.462735	4.331991
PPIA	1.33E-06	15.9069	6.203769	4.328154
FAM84B	4.09E-05	9.375402	2.794729	4.326888
ZNF518A	2.78E-06	14.21882	5.507826	4.326825
RPS3A	7.27E-07	17.42808	6.752305	4.320645
INTS2	3.39E-07	19.55548	7.416382	4.320498
COL4A4	8.89E-07	16.90769	6.572226	4.317635
LLPH-AS1///LLPH	4.17E-06	13.36438	5.114684	4.31395
ADO	6.92E-05	8.625278	2.242421	4.313447

BCL10	1.08E-04	8.028997	1.769345	4.31074
SF3B1	6.28E-06	12.55236	4.711879	4.305126
VPS13D	1.32E-05	11.19742	3.967512	4.302145
ARL13B	6.71E-07	17.63872	6.823096	4.300723
RPL24	1.37E-06	15.83087	6.174516	4.294978
DBT	1.49E-06	15.64115	6.100703	4.28913
CDC42-IT1	9.61E-06	11.75639	4.286348	4.288275
AR	5.69E-05	8.897442	2.448072	4.278798
C2orf57	1.10E-04	8.009967	1.753713	4.278711
QKI	1.03E-03	5.495097	-0.65847	4.278392
UBA6	1.04E-04	8.078135	1.809552	4.276947
CMTR2	1.95E-05	10.53117	3.563791	4.267893
STRIP2	5.70E-06	12.74167	4.808492	4.239141
HSPD1	5.24E-04	6.177815	0.07135	4.237633
ATP11B	4.25E-06	13.32507	5.095861	4.236465
ITPRIPL2	3.98E-06	13.46047	5.160408	4.235428
SRGN	2.75E-05	9.981368	3.209484	4.235254
NCOA2	1.65E-04	7.49138	1.314189	4.234704
CEP170	5.07E-05	9.061593	2.569164	4.232365
HSP90B1	6.24E-05	8.768786	2.351629	4.230828
HSPA13	1.53E-05	10.93817	3.813602	4.230378
CLIP1	2.16E-06	14.77819	5.749655	4.222084
ZNF25	2.96E-07	19.95854	7.530426	4.219578
DUT	2.71E-07	20.21968	7.602491	4.217167
LILRB1	2.04E-06	14.90414	5.802513	4.213107
HIST1H4C	5.67E-06	12.74999	4.812698	4.211995
SIKE1	4.66E-07	18.63721	7.143126	4.211076
SENP7	1.86E-07	21.38878	7.908544	4.208923
OXR1	8.21E-06	12.04583	4.444811	4.204414
CEP170	9.02E-05	8.265458	1.960775	4.202529
ZNF492	2.71E-06	14.27746	5.533733	4.201208
SNHG10	4.40E-06	13.25799	5.063595	4.199739
SLC31A1	7.28E-05	8.555777	2.188902	4.198901
CRY1	2.15E-05	10.37133	3.462837	4.195479
MYBL1	7.40E-05	8.532696	2.171036	4.195178
TDG	4.66E-06	13.14116	5.00692	4.191137
BRD7P3	6.25E-05	8.766009	2.349532	4.182949
BOLA2-SMG1P6	2.84E-06	14.17126	5.486716	4.180384
SURF4	3.99E-04	6.470279	0.365525	4.174639
ZNF254	1.06E-06	16.47371	6.41615	4.172355
ZNF709	1.52E-06	15.58789	6.079771	4.169549
AP5M1	1.05E-05	11.60282	4.200473	4.169072
MICALCL	2.93E-04	6.81445	0.698438	4.168707
MTERF1	1.74E-03	5.001697	-1.22606	4.165176
ASF1A	9.39E-06	11.79808	4.309445	4.163491
ERICH1	6.16E-06	12.59019	4.731323	4.159476
LOC100507663	4.86E-06	13.05666	4.965549	4.158285
TXLNG	9.89E-06	11.70462	4.257539	4.155189

RAB5A	2.64E-06	14.33135	5.557425	4.149791
USP15	1.70E-05	10.75715	3.703752	4.1493
ZC3H7B	2.53E-04	6.984527	0.857878	4.148602
LRRC58	1.19E-04	7.906674	1.668264	4.14498
CPEB3	6.94E-04	5.88689	-0.23206	4.14427
MT1M	6.78E-05	8.652536	2.263298	4.143324
SLC10A7	6.72E-06	12.42186	4.644284	4.141155
ZNF644	9.52E-04	5.570946	-0.57428	4.138359
TAF2	5.22E-06	12.91247	4.894217	4.137457
ANKRD36BP2	4.11E-05	9.370513	2.791272	4.135997
CTSO	1.35E-04	7.74061	1.528732	4.130683
FYB	9.31E-06	11.81414	4.31832	4.128718
CR1	3.85E-05	9.467215	2.859296	4.126755
PBX1	1.15E-06	16.25546	6.335544	4.12456
SOCS4	9.86E-07	16.6435	6.47787	4.121857
RNF138	1.73E-04	7.433087	1.263096	4.118812
SLC38A2	1.12E-05	11.47786	4.129641	4.114805
IL6ST	2.68E-05	10.0205	3.23537	4.109856
ZNF467	1.10E-04	8.000143	1.74563	4.109277
IPPK	6.91E-06	12.36959	4.616974	4.104873
SPTY2D1	3.70E-07	19.29359	7.340393	4.104658
TRG-AS1	8.17E-06	12.05387	4.449148	4.102356
HACD3	6.76E-07	17.62048	6.817012	4.100641
PANK3	1.69E-05	10.77196	3.712814	4.095372
ZNF680	5.32E-07	18.26701	7.027378	4.095155
CDX4	3.41E-06	13.7845	5.311723	4.081683
PLEKHB2	5.53E-05	8.938775	2.478768	4.079833
PNISR	5.56E-05	8.932038	2.473775	4.078698
DESI1	7.24E-06	12.2805	4.570123	4.07492
STX7	4.23E-06	13.33694	5.101552	4.071305
CEP85L	1.21E-05	11.34777	4.054989	4.068061
KAT6A	7.67E-05	8.482966	2.132386	4.066353
TOLLIP	5.43E-05	8.96353	2.497086	4.063781
FAM63A	1.67E-05	10.78908	3.723274	4.063695
PDGFC	3.13E-05	9.778916	3.073853	4.06189
LCORL	6.63E-06	12.44808	4.657928	4.060909
PYROXD1	1.64E-06	15.4033	6.006496	4.058904
PTP4A1	6.68E-04	5.926191	-0.19043	4.058857
LPGAT1	7.93E-06	12.11066	4.479706	4.058037
PLXNC1	1.13E-04	7.972439	1.722785	4.057187
CPEB2	1.98E-05	10.50987	3.550431	4.057017
SLC26A2	1.58E-05	10.88242	3.779984	4.055414
PRR13	7.32E-05	8.546892	2.18203	4.054731
RFC1	4.63E-06	13.15334	5.012854	4.053445
TAS2R45	1.38E-05	11.11622	3.91973	4.048693
DNAJC3	4.08E-05	9.381233	2.798848	4.04868
SF3B1	1.22E-05	11.3337	4.046859	4.048472
EIF3E	2.04E-05	10.45693	3.517107	4.0471

TBL1XR1	4.25E-04	6.402417	0.298209	4.044439
HNRNPH1	1.45E-04	7.653528	1.45448	4.041544
PDE7A	1.31E-05	11.19941	3.968682	4.038608
RNMT	3.79E-05	9.490817	2.875791	4.038588
TAOK1	4.50E-05	9.236066	2.695518	4.035615
DISC1	1.59E-04	7.541543	1.357876	4.030833
TACC1	2.25E-05	10.2971	3.415394	4.028271
RGPD2	1.02E-03	5.499154	-0.65395	4.026516
TAF3	2.37E-06	14.56887	5.660532	4.022383
FCN2	2.61E-04	6.95019	0.825952	4.021119
TERF1	6.65E-07	17.66437	6.831634	4.020052
CASD1	1.48E-03	5.149629	-1.05221	4.019958
FCRL1	1.53E-05	10.93431	3.811277	4.018723
LMNB2	2.22E-06	14.71781	5.724114	4.017171
PPP6R1	2.52E-06	14.43708	5.603586	4.014129
DHFR	1.05E-04	8.068497	1.801684	4.010255
BCCIP	3.58E-05	9.574092	2.933664	4.008642
GSTA5	3.70E-04	6.553214	0.447034	4.005247
MIB1	5.08E-05	9.059112	2.567349	4.004632
MGA	5.17E-06	12.93449	4.905172	4.001647
SLC35E3	3.65E-05	9.545115	2.913585	3.999883
RAB21	5.76E-04	6.079955	-0.02949	3.99669
STK10	2.75E-06	14.245	5.519409	3.994384
SRP72	1.15E-04	7.943528	1.698868	3.992766
NOTCH2NL	3.56E-05	9.582735	2.939642	3.989246
BRWD1	3.38E-03	4.416781	-1.94523	3.979071
ZSWIM8	2.28E-06	14.6551	5.697441	3.97851
MYBL1	4.43E-05	9.259154	2.71206	3.977989
LOC100132217	2.77E-07	20.15555	7.584924	3.976691
ZFP1	9.14E-06	11.8479	4.336923	3.970661
PARP11	4.79E-05	9.144906	2.629797	3.96727
NAA16	3.29E-06	13.8601	5.346399	3.966555
MIB1	4.02E-05	9.40102	2.812808	3.965701
ZNF738	3.07E-06	14.00628	5.412798	3.963731
SP140	4.58E-06	13.17506	5.023423	3.963023
LGALS8	1.58E-05	10.88306	3.78037	3.962887
RP2	7.34E-05	8.543972	2.17977	3.960172
PDCD4	7.60E-06	12.18868	4.521423	3.958934
B2M	3.63E-07	19.35503	7.358356	3.954916
FAM35A	5.11E-05	9.05062	2.561137	3.952247
EXOC5	1.40E-05	11.09372	3.906422	3.952008
LOC389705	2.34E-05	10.236	3.376066	3.951955
IKZF1	4.35E-06	13.28121	5.074787	3.949024
ETNK1	2.19E-06	14.74972	5.73763	3.947707
CD46	6.73E-05	8.662558	2.270958	3.946193
ABCC4	4.19E-05	9.34101	2.770379	3.9455
TWF1	4.02E-05	9.402339	2.813738	3.941875
TSNAX	1.10E-06	16.37119	6.378468	3.941565

EHBP1	3.37E-05	9.666668	2.997403	3.934374
GTF2A1	7.90E-06	12.11592	4.482528	3.930801
ZC3H13	1.16E-05	11.42169	4.097523	3.924242
CARNMT1	8.01E-06	12.09226	4.469825	3.923825
PKP4	6.30E-06	12.54634	4.708781	3.921369
CDK11B	9.16E-07	16.82914	6.544381	3.919369
ZNF562	1.23E-06	16.09099	6.273838	3.918813
DMXL2	9.68E-05	8.172374	1.886033	3.913948
LYRM5	2.43E-04	7.030538	0.900451	3.911713
ZNF22	3.10E-06	13.98671	5.403958	3.903739
ANKRD29	1.72E-05	10.73721	3.69153	3.896505
PTPRE	1.40E-04	7.701509	1.495485	3.891625
HNRNPKP3	2.92E-06	14.1172	5.462611	3.8901
ANP32E	3.16E-04	6.73146	0.619438	3.888838
EP300	5.45E-05	8.959598	2.49418	3.888297
COCH	1.63E-06	15.42905	6.016788	3.886819
ZNF292	4.58E-05	9.208974	2.676054	3.886791
USP10	2.09E-04	7.209915	1.064199	3.886336
PTPRE	2.27E-04	7.112681	0.975874	3.885577
PBRM1	5.72E-05	8.890532	2.442927	3.882391
FAM120A	2.93E-05	9.880605	3.142338	3.873137
CAND1	3.71E-05	9.523791	2.898769	3.872731
ZNF789	2.83E-06	14.17776	5.489607	3.871632
PCDHA11	9.41E-06	11.79485	4.307657	3.871607
MPHOSPH8	4.14E-05	9.357101	2.781782	3.871602
PHIP	4.36E-06	13.27466	5.071632	3.870249
HOOK3	4.35E-04	6.377725	0.273576	3.869823
CISH	5.86E-06	12.68788	4.781213	3.867265
ALCAM	2.31E-04	7.09032	0.955415	3.866492
APPL1	1.07E-05	11.55493	4.173431	3.863968
KCNA3	1.64E-03	5.052222	-1.16633	3.863755
ZXDC	3.42E-06	13.77756	5.308528	3.86268
SULT1B1	4.92E-06	13.03143	4.953139	3.861153
C4orf46	9.69E-07	16.68879	6.494188	3.859341
ANKRD26	8.44E-05	8.355104	2.032015	3.857224
CCR1	4.35E-04	6.377465	0.273316	3.857206
CLIC2	2.84E-03	4.564212	-1.75914	3.855735
HPSE	1.29E-05	11.23368	3.988727	3.854702
AASDH	3.40E-05	9.653547	2.988407	3.854399
LAMA4	9.76E-04	5.545996	-0.60189	3.854034
SNX16	4.19E-04	6.418351	0.314066	3.852873
NPLOC4	2.35E-04	7.068525	0.935423	3.852154
BZW1	1.57E-05	10.89107	3.78521	3.852076
RBM22	6.13E-05	8.794315	2.370875	3.850823
TBL1XR1	4.90E-07	18.49575	7.099288	3.848873
RBBP4	3.86E-06	13.52619	5.191454	3.846521
EFHB	8.04E-06	12.08319	4.464946	3.843987
SCLT1	3.53E-04	6.607814	0.500244	3.843282

TMED2	1.29E-04	7.803499	1.58189	3.839673
AR	5.86E-06	12.68643	4.780475	3.838924
ANKRD36B	1.13E-04	7.976121	1.725826	3.838708
LIN7C	2.01E-05	10.48018	3.531764	3.836453
SMG1	3.60E-05	9.566079	2.928118	3.834908
GSKIP	9.97E-06	11.68904	4.248845	3.834728
NDC1	9.60E-05	8.182595	1.894278	3.833369
ARL4A	9.50E-06	11.77769	4.298163	3.831775
MACF1	1.47E-04	7.633512	1.437306	3.831376
LINC00691	3.19E-06	13.92622	5.376539	3.831284
LOC283701	1.70E-05	10.7614	3.706358	3.831238
FAR1	4.05E-05	9.390943	2.805702	3.830764
NCBP2	7.91E-03	3.724685	-2.86187	3.830469
LOC100190986	1.43E-04	7.67296	1.471115	3.826232
DCAF13	1.41E-06	15.77011	6.151002	3.82265
RUFY2	6.13E-05	8.792917	2.369822	3.822238
FAM199X	1.10E-04	8.005477	1.75002	3.821017
PAPOLA	4.02E-05	9.40261	2.813929	3.819909
NEK1	1.94E-05	10.54154	3.570284	3.816482
MOSPD2	2.78E-05	9.966522	3.199635	3.815755
TFEC	2.49E-05	10.13861	3.312873	3.815367
SREK1	1.78E-06	15.21194	5.929317	3.813904
SRSF6	3.20E-04	6.714758	0.603442	3.813464
TMED10P1	7.59E-06	12.19116	4.522743	3.8122
DOCK4	1.36E-03	5.22562	-0.96414	3.807564
LIG4	6.30E-04	5.9865	-0.12694	3.806126
AP3D1	3.61E-06	13.66353	5.255742	3.805955
NBEAL1	1.59E-05	10.86852	3.771572	3.798028
YPEL2	3.98E-04	6.475363	0.370545	3.797053
LATS2	5.79E-04	6.074024	-0.03564	3.79391
CSNK1A1P1	5.47E-05	8.954017	2.490053	3.792612
MFSD6	1.16E-04	7.937752	1.69408	3.786304
SYNCRIP	1.29E-05	11.23403	3.988931	3.782775
PDXDC2P	1.79E-05	10.67631	3.654052	3.782734
CHORDC1	7.46E-06	12.22499	4.540733	3.7778
APPL1	3.25E-06	13.88589	5.358175	3.7757
SLC8A1	2.44E-04	7.026316	0.896554	3.773807
DBT	3.82E-06	13.54417	5.199915	3.771596
GLIPR1	1.78E-05	10.68565	3.659816	3.771149
ZNF492	8.99E-07	16.877	6.561367	3.770335
BMI1	1.95E-04	7.293677	1.139469	3.765234
MSN	1.11E-05	11.50258	4.143725	3.762848
CHMP2B	6.57E-05	8.695745	2.296263	3.759539
BMT2	1.11E-04	7.998709	1.744449	3.757863
FNIP1	2.18E-05	10.35363	3.451557	3.756499
DNAJB4	3.37E-04	6.659458	0.550247	3.756158
GCH1	7.50E-05	8.513868	2.156428	3.754425
OTUD6B	9.55E-07	16.72336	6.506605	3.754346

PPM1A	2.89E-05	9.901929	3.156607	3.752558
DNAJB4	6.51E-04	5.953054	-0.16209	3.752145
ZNF420	2.03E-05	10.46946	3.525008	3.752049
NR4A2	1.13E-04	7.970925	1.721535	3.750835
SLC25A36	4.24E-06	13.33313	5.099728	3.750517
PTEN	4.93E-05	9.103653	2.599843	3.750219
EBLN3P	3.10E-04	6.751796	0.63887	3.749135
PPP1R12A	2.22E-05	10.32198	3.431331	3.747571
PAPD4	7.04E-06	12.33313	4.597848	3.745708
SOWAHA	5.24E-05	9.014819	2.534881	3.742924
CACUL1	6.17E-04	6.00733	-0.10512	3.740431
ZNF146	3.93E-04	6.489282	0.384274	3.73898
ZNF714	1.10E-06	16.35943	6.374124	3.738198
LRRCC1	2.78E-05	9.966926	3.199904	3.735339
WHSC1L1	1.93E-05	10.54677	3.573555	3.730665
SLC43A2	2.04E-05	10.45885	3.518317	3.728946
ZCCHC6	2.12E-06	14.82387	5.768891	3.725997
TSPAN9	6.92E-05	8.623726	2.24123	3.725666
ARL14EP	2.13E-06	14.80362	5.760376	3.724516
SNX18	1.48E-04	7.626057	1.430899	3.719051
MALAT1	1.57E-05	10.90012	3.790675	3.718426
TCAF1	2.06E-05	10.44487	3.509486	3.717578
TMEM261	2.23E-05	10.31179	3.424811	3.717515
MEX3C	6.10E-04	6.019411	-0.09249	3.715112
EIF2AK3	8.09E-07	17.14815	6.656374	3.712311
CSNK1G1	2.63E-05	10.05267	3.256577	3.7121
RHOT1	5.82E-05	8.866962	2.425347	3.711479
ERC1	2.83E-05	9.937433	3.180294	3.710819
ZNF680	7.03E-07	17.5157	6.781896	3.70912
INO80D	3.60E-03	4.362441	-2.01465	3.708488
KIAA1586	5.49E-06	12.81361	4.844766	3.705552
GMFB	2.20E-04	7.15049	1.010341	3.705027
AVL9	4.02E-06	13.44048	5.150927	3.70469
ZNF765	3.20E-05	9.747484	3.052536	3.698414
ZNF578	5.04E-06	12.98444	4.929943	3.69646
POM121	4.69E-06	13.12759	5.000298	3.695613
TCEA1	9.33E-05	8.220992	1.92517	3.694302
ZDHHC21	1.21E-06	16.13339	6.289826	3.693194
DIS3L2	8.11E-04	5.729528	-0.40082	3.692642
ASAP1-IT1	1.52E-05	10.95433	3.823308	3.692435
FAM126A	1.17E-05	11.39897	4.084482	3.688848
LMBR1	3.52E-06	13.71668	5.280411	3.685881
RASA2	1.04E-05	11.61684	4.208366	3.684915
LOC100506282	1.85E-06	15.12923	5.89557	3.680455
ZNF283	1.26E-05	11.27513	4.012886	3.680026
ZDHHC17	6.51E-07	17.72101	6.850431	3.677823
MAPK6	2.58E-05	10.0827	3.276302	3.675118
POLB	6.83E-05	8.642929	2.255947	3.673467

ZNF441	1.10E-05	11.51088	4.148445	3.672365
ACTR2	5.34E-04	6.158685	0.051734	3.66957
ITPKB	9.17E-05	8.243693	1.94337	3.668849
LONP2	1.38E-06	15.82318	6.171546	3.668569
ITSN1	1.46E-06	15.68418	6.117546	3.664591
CHD6	1.48E-05	10.99622	3.848409	3.662641
TBL1XR1	7.87E-07	17.22137	6.681673	3.661437
HLA-F-AS1	1.28E-06	15.99575	6.23772	3.659861
WDR47	1.39E-06	15.80055	6.162799	3.658041
ZNF292	9.77E-07	16.66743	6.486501	3.657217
EIF4E	1.58E-05	10.88396	3.780915	3.655403
TBC1D24	7.20E-05	8.571018	2.200673	3.655224
LOC100128164	4.57E-05	9.213543	2.67934	3.651917
IFT74	2.54E-05	10.10827	3.293051	3.651644
ZNF493	1.73E-05	10.73395	3.68953	3.650899
CPEB3	5.29E-06	12.88706	4.881551	3.650399
MTMR6	1.70E-04	7.454688	1.28207	3.64741
CFAP44	1.47E-05	11.00569	3.854064	3.645126
SPIN4	9.44E-05	8.20565	1.912843	3.644202
SEPT7	1.08E-05	11.55312	4.172405	3.644054
OXR1	2.98E-05	9.855925	3.125784	3.64311
FAF2	3.71E-06	13.60681	5.229287	3.642505
ABCE1	1.78E-05	10.68684	3.660549	3.641134
SCAMP1	1.34E-05	11.1602	3.94566	3.640417
SMN2	3.05E-05	9.822429	3.103248	3.638842
HK2	2.41E-04	7.041587	0.910639	3.638198
CREB1	2.31E-04	7.092748	0.957639	3.637574
TBC1D23	4.98E-06	13.00725	4.941215	3.637018
ARHGAP5	2.20E-06	14.73224	5.730229	3.636687
EPS15	3.66E-06	13.63764	5.243685	3.634223
ZBTB44	2.00E-05	10.49002	3.537957	3.63388
ZNF577	4.53E-06	13.19551	5.033357	3.63357
CHM	2.80E-06	14.2051	5.501743	3.631337
NBPF3	4.83E-05	9.132374	2.620712	3.630671
MAGEA8	3.42E-04	6.641711	0.5331	3.63064
RNF13	4.52E-04	6.337001	0.232784	3.630409
ERAP2	6.91E-05	8.626596	2.243432	3.629277
NIPSNAP3B	6.82E-05	8.644288	2.256987	3.626552
RAB11FIP2	2.38E-06	14.56077	5.657052	3.625647
VAV2	1.27E-06	16.02407	6.248489	3.624019
MYEF2	2.12E-04	7.193055	1.048957	3.618715
GTF3C3	3.03E-05	9.828441	3.107298	3.617826
ARID5B	1.28E-04	7.811786	1.588866	3.617203
SSB	9.59E-06	11.75926	4.287943	3.615467
CCNG2	3.88E-04	6.502783	0.397569	3.614808
ZNF714	1.52E-06	15.59048	6.080793	3.608216
PRKAB2	7.32E-07	17.41181	6.74679	3.607351
IL1R2	7.16E-07	17.46894	6.766131	3.606864

FANCM	6.83E-06	12.39121	4.628289	3.606679
EXOC5	2.68E-05	10.02036	3.235276	3.60614
NSF	1.72E-04	7.440246	1.26939	3.603153
TMTC1	6.98E-03	3.822509	-2.72812	3.602772
CEP85L	3.14E-04	6.738987	0.626636	3.602112
ROCK1	6.55E-05	8.701193	2.300408	3.601329
SAMHD1	3.34E-05	9.682714	3.008387	3.597694
SLC20A2	2.74E-04	6.890742	0.770364	3.597382
BMPR2	8.48E-07	17.02914	6.614931	3.591774
RAB9BP1	1.27E-05	11.2642	4.006524	3.588707
FAT2	5.87E-04	6.058785	-0.05146	3.586634
LOC283788	9.83E-05	8.151305	1.869006	3.585828
USP51	2.74E-05	9.985866	3.212465	3.585697
NSA2	6.32E-05	8.749841	2.337311	3.584039
NR5A1	1.46E-05	11.02348	3.864682	3.579848
AIDA	1.18E-05	11.38772	4.078015	3.579071
ESAM	5.99E-06	12.64318	4.758438	3.578654
SMARCC1	1.10E-05	11.51688	4.151853	3.57714
ABHD2	4.92E-04	6.245191	0.140066	3.575429
KIAA1109	7.70E-05	8.478006	2.128518	3.574805
ARID2	1.60E-05	10.8661	3.770102	3.573623
PRPF4B	2.66E-05	10.03628	3.24578	3.572711
TMEM123	3.48E-06	13.74004	5.291219	3.57151
ZNF766	9.94E-07	16.62402	6.470833	3.571494
ZNF37A	1.47E-05	11.01089	3.857174	3.568951
RYBP	1.13E-03	5.405855	-0.75856	3.568879
NUP58	6.10E-05	8.798997	2.374398	3.568375
TMTC2	9.88E-05	8.14513	1.864007	3.56832
TPR	2.43E-05	10.17479	3.336422	3.567908
MED1	4.97E-04	6.235075	0.129786	3.567673
NDUFS4	2.23E-06	14.70403	5.718266	3.566979
MAP4K3	1.15E-04	7.945847	1.70079	3.561778
SNHG20	1.84E-06	15.14076	5.900286	3.55821
TMOD3	2.91E-06	14.12376	5.465543	3.5574
AGAP9	3.92E-06	13.49476	5.176628	3.555509
ERP44	4.15E-05	9.353602	2.779305	3.553575
LOC148709	5.07E-05	9.062278	2.569664	3.551157
ZNF169	1.10E-05	11.50596	4.145647	3.55095
CCDC88B	4.19E-05	9.341636	2.770822	3.550434
SYNJ1	2.31E-05	10.25896	3.390872	3.550245
ITGA2	1.40E-05	11.09287	3.905917	3.55008
HDAC9	1.67E-04	7.476552	1.301226	3.54925
ZNF644	1.22E-06	16.11868	6.284284	3.544918
ATF7IP	1.39E-05	11.10039	3.910368	3.544562
LYRM7	1.71E-06	15.31471	5.970923	3.543541
PPP4R2	3.32E-05	9.691508	3.014399	3.541309
RASA2	1.22E-05	11.33286	4.046373	3.536741
CYP4F3	1.39E-06	15.7997	6.162468	3.536135

CD46	9.15E-05	8.247293	1.946252	3.535177
KMT5B	4.21E-05	9.332518	2.764352	3.533019
ANTXR2	1.95E-05	10.53365	3.565342	3.53265
TMEM33	6.77E-06	12.40853	4.63733	3.532601
C2CD2	1.59E-06	15.48196	6.037862	3.530637
DLGAP4	4.95E-05	9.096579	2.594692	3.530187
YBX1	4.07E-06	13.41581	5.139206	3.528698
UFM1	3.69E-06	13.62076	5.235806	3.528524
TMED2	2.80E-04	6.868483	0.749448	3.527507
FBXW7	2.65E-05	10.03972	3.248046	3.526565
MAP3K2	2.06E-03	4.849096	-1.40878	3.525059
USP32P1	2.48E-03	4.681003	-1.61404	3.5209
SLF1	8.54E-06	11.97144	4.404499	3.520369
KMT2E	4.24E-05	9.322291	2.757087	3.520011
RASSF4	6.46E-05	8.720091	2.314767	3.519724
ABCB10	8.22E-06	12.04287	4.443209	3.519003
ATMIN	1.29E-05	11.22763	3.985191	3.51705
VPS4B	9.43E-06	11.79003	4.304995	3.515262
CALU	3.05E-04	6.7718	0.657938	3.513536
LMTK2	1.66E-06	15.37382	5.994686	3.512716
IMPACT	3.37E-05	9.66938	2.99926	3.507776
MON1B	4.96E-05	9.095092	2.593609	3.507077
LOC255308	2.94E-03	4.533915	-1.79711	3.505899
CDHR3	3.85E-05	9.4653	2.857955	3.502586
TMEM259	9.46E-06	11.78436	4.301856	3.50201
ZNF808	9.48E-05	8.199307	1.90774	3.497587
RBMXL1	1.37E-04	7.722834	1.513636	3.497089
GCOM1	2.07E-06	14.86779	5.787319	3.497016
UCHL5	2.65E-05	10.04144	3.24918	3.495219
CCDC57	1.01E-05	11.67382	4.240331	3.492574
DCUN1D1	3.06E-05	9.814137	3.097657	3.490579
CXCL5	3.52E-05	9.603005	2.953638	3.490171
PPP4R2	3.63E-04	6.576753	0.470017	3.489657
ZCRB1	2.15E-04	7.176587	1.03404	3.489451
KDM6B	1.81E-04	7.383752	1.21958	3.487984
PAPD5	1.72E-05	10.74264	3.694861	3.487348
MBNL3	1.34E-05	11.16091	3.946078	3.484758
NPAT	9.07E-05	8.257738	1.954607	3.483939
ZNF141	7.35E-05	8.541528	2.177878	3.482248
PLEKHA1	1.14E-05	11.45019	4.113845	3.482133
PLEKHB2	1.06E-04	8.053457	1.789387	3.481766
NBPF10	6.32E-05	8.75009	2.3375	3.481718
BCL10	3.34E-04	6.669443	0.559879	3.481561
GNAS	3.25E-05	9.724843	3.037138	3.4812
SLC25A40	4.26E-06	13.32021	5.093529	3.481099
PPP1R12A	1.66E-05	10.79971	3.72976	3.479697
CAPRIN1	3.28E-05	9.709209	3.026483	3.478383
TCEAL5	1.18E-06	16.19999	6.314825	3.47826

GNA13	2.19E-05	10.34076	3.443341	3.477295
CAPZA2	1.13E-05	11.46354	4.121474	3.476538
IPO8	1.63E-05	10.8364	3.752088	3.476458
ARSK	1.27E-03	5.289755	-0.89045	3.474492
COQ9	7.01E-07	17.52257	6.784207	3.473618
RMDN1	1.27E-03	5.296624	-0.8826	3.472975
CCDC191	3.88E-06	13.51596	5.186632	3.472934
CD84	5.26E-06	12.89921	4.887613	3.47252
BLOC1S5	1.15E-05	11.43947	4.107713	3.472357
CCSAP	5.19E-05	9.02869	2.545066	3.471144
RBM47	1.34E-04	7.75296	1.539202	3.469633
DKFZP586B0319	1.36E-05	11.13973	3.933602	3.469045
YWHAEP1	2.15E-03	4.809759	-1.45643	3.468789
UBA6	8.59E-06	11.96095	4.398796	3.468399
ZBTB18	5.96E-05	8.832592	2.39963	3.467913
WWC2	2.67E-03	4.61738	-1.69283	3.466102
LRP8	2.11E-06	14.82962	5.771309	3.466094
SERP1	1.15E-04	7.95145	1.70543	3.465731
NEDD1	2.52E-05	10.11934	3.300292	3.463318
ELL2	5.78E-05	8.874891	2.431266	3.46115
SAMD9	1.33E-05	11.18198	3.958458	3.459126
ANKRD36B	1.79E-05	10.67263	3.65178	3.458391
TMEM192	2.95E-04	6.809661	0.693901	3.457178
BMS1P5	2.28E-05	10.28075	3.404895	3.456322
AMPD3	8.81E-05	8.297219	1.986098	3.455726
LYPLA1	8.44E-05	8.354481	2.031522	3.454255
DNAJC21	2.01E-04	7.25518	1.104968	3.4525
FAM126B	4.23E-06	13.33779	5.101958	3.451679
LTN1	1.49E-05	10.98613	3.842374	3.45123
NBPF11	5.57E-05	8.927449	2.470371	3.450162
FAR1	5.72E-05	8.891204	2.443427	3.449699
VPS13B	2.29E-05	10.27137	3.398862	3.448574
XIAP	1.85E-06	15.12948	5.895673	3.448107
PLPP5	2.09E-05	10.4161	3.491277	3.447082
ZDHHC2	7.69E-06	12.16643	4.509555	3.445305
CCNT2	1.50E-05	10.96707	3.830953	3.44446
RBM41	4.93E-05	9.102767	2.599198	3.443449
C9orf72	2.50E-05	10.13331	3.309416	3.442006
WSB1	3.07E-05	9.809755	3.0947	3.441075
LACC1	6.78E-06	12.40486	4.635418	3.440947
POM121	2.06E-05	10.44682	3.51072	3.440276
MTAP	9.41E-06	11.7939	4.307136	3.440153
FIP1L1	1.47E-05	11.00676	3.854703	3.437649
ZNF681	9.99E-07	16.61143	6.466276	3.43602
NFE2L1	2.97E-03	4.527465	-1.80522	3.431933
ACTR2	6.36E-06	12.52883	4.69975	3.430527
DSTYK	2.68E-06	14.29992	5.543623	3.429583
THAP9	3.27E-05	9.711671	3.028163	3.428521

ASH1L	3.86E-04	6.507482	0.402191	3.428347
HIST1H3E	2.69E-05	10.01419	3.231205	3.427491
OSTM1	1.39E-05	11.09824	3.909095	3.425458
NKTR	1.58E-06	15.49217	6.041918	3.42362
HOOK3	3.14E-05	9.776708	3.072358	3.423259
PDE12	6.99E-06	12.34646	4.604848	3.421783
NEDD1	1.30E-05	11.21571	3.978223	3.421055
SLC31A1	3.33E-05	9.686157	3.010741	3.420888
CASP8	6.55E-05	8.70162	2.300733	3.420165
ZMYND8	1.36E-05	11.13713	3.932069	3.4197
IRX5	5.52E-03	4.010021	-2.4755	3.417781
IPO8	2.24E-05	10.30546	3.420752	3.41714
LYPLA1	1.02E-04	8.102551	1.829446	3.415309
SYCP2	3.14E-03	4.478871	-1.86646	3.414405
SLC9A6	1.60E-05	10.86297	3.768207	3.414203
PPTC7	1.71E-04	7.453272	1.280828	3.414155
ABCA1	3.44E-06	13.76265	5.301654	3.413863
PNISR	6.07E-05	8.807435	2.380745	3.411135
SETX	1.47E-04	7.639185	1.442177	3.410765
RPL13P5	9.35E-06	11.80547	4.313528	3.410625
DHFR2	6.83E-06	12.39194	4.628667	3.407978
FRYL	1.59E-05	10.87142	3.773326	3.407652
TSNAX	6.34E-05	8.747237	2.335341	3.406466
ZNF430	4.12E-06	13.39192	5.127829	3.40535
OR2V2	4.55E-04	6.329442	0.22519	3.405286
ANKRD17	1.49E-05	10.98077	3.839164	3.404478
NCOA3	3.96E-06	13.47286	5.166272	3.401808
HELQ	4.16E-03	4.241573	-2.17063	3.401144
GJC3	3.17E-05	9.760853	3.061612	3.401013
PRIM2	1.78E-03	4.977547	-1.25475	3.399344
RIF1	5.47E-06	12.82037	4.848159	3.397423
LYRM7	1.63E-04	7.506741	1.327594	3.395579
SPAG9	1.25E-05	11.28266	4.017266	3.394627
SEC62	4.99E-05	9.085849	2.586874	3.39122
RUFY3	1.86E-04	7.348007	1.187891	3.390483
TYW3	8.61E-06	11.95663	4.396444	3.38862
MAP3K2	2.16E-04	7.170839	1.028826	3.387004
NOTCH2NL	5.93E-04	6.049533	-0.06108	3.386249
ORAOV1	1.68E-05	10.77889	3.717051	3.385216
QKI	1.12E-05	11.48016	4.130953	3.384096
FUNDC2	5.60E-06	12.77706	4.826364	3.383827
CR1	4.43E-04	6.357423	0.253266	3.383681
CCDC126	1.80E-05	10.66517	3.647167	3.383537
TRIM52	6.64E-06	12.44582	4.656754	3.382782
SLC4A7	5.81E-04	6.069878	-0.03994	3.38216
CELF6	1.75E-04	7.418997	1.250694	3.380826
SH3KBP1	3.29E-06	13.85671	5.344852	3.379976
FZD3	3.03E-05	9.832032	3.109717	3.379228

HIST1H2AC	8.88E-04	5.639694	-0.49866	3.377962
CCDC91	8.16E-05	8.39937	2.066926	3.376942
PRKG1	1.63E-05	10.82989	3.748131	3.376681
SLFN5	2.73E-05	9.99367	3.217634	3.375773
CEP295	9.53E-07	16.73101	6.509348	3.375142
SON	9.12E-06	11.85168	4.339004	3.375027
RIOK2	9.44E-06	11.78806	4.303905	3.374531
ARMCX3	2.65E-06	14.32219	5.553405	3.37403
LOC114224	5.60E-05	8.921912	2.466262	3.373318
RANBP2	2.23E-05	10.31527	3.427038	3.372235
MPP7	4.65E-05	9.18762	2.660672	3.372134
G2E3	3.32E-05	9.691109	3.014126	3.371164
HIST1H3A	2.09E-05	10.41921	3.493251	3.370308
GSPT1	1.51E-06	15.59651	6.083168	3.367916
KNL1	6.03E-06	12.63023	4.751827	3.36617
EPHA4	3.60E-06	13.66982	5.258668	3.36591
PRKAR1A	1.52E-05	10.95252	3.822224	3.361343
ETNK1	4.64E-05	9.190423	2.662693	3.36071
ANKRD13A	3.17E-05	9.759462	3.060669	3.3605
RUFY2	6.19E-06	12.57947	4.725817	3.356727
TMEM30A	7.13E-05	8.584185	2.210827	3.355921
POLD3	5.15E-06	12.94108	4.908449	3.355371
GNB4	1.28E-04	7.813537	1.590338	3.354534
RACGAP1	4.11E-06	13.39754	5.130505	3.354503
USP48	5.10E-04	6.207631	0.10183	3.352361
ENC1	5.55E-04	6.118428	0.010303	3.352019
CCR1	3.15E-05	9.771528	3.068849	3.351897
SMG1	1.72E-06	15.29541	5.963135	3.35067
HIPK3	3.06E-04	6.765406	0.651849	3.349495
HCG4	7.64E-06	12.17888	4.5162	3.348853
EXOC6	1.47E-04	7.640733	1.443507	3.348343
NGLY1	3.55E-06	13.69783	5.271675	3.347155
USP3	2.42E-04	7.037221	0.906615	3.345794
CLN8	1.55E-04	7.572723	1.384901	3.34379
ANP32A	2.72E-04	6.899005	0.778114	3.343532
CCDC122	3.20E-06	13.91849	5.373026	3.341275
MSL3	8.60E-06	11.95951	4.398008	3.339088
FAR1	2.83E-04	6.855146	0.736888	3.336971
CYP1B1	5.49E-05	8.949196	2.486486	3.336793
AKIRIN1	1.12E-05	11.47926	4.13044	3.336164
PABPC3	9.15E-05	8.247317	1.946271	3.333952
DCAF16	1.44E-05	11.04663	3.878471	3.331439
GOLGA8A	4.14E-03	4.245411	-2.16564	3.32489
IQGAP2	3.67E-05	9.539395	2.909613	3.324811
FTHL17	7.87E-05	8.448513	2.105482	3.324645
NPM1	2.09E-06	14.85295	5.781099	3.323139
ARL8B	1.55E-04	7.573005	1.385145	3.320062
RPS11P6	2.49E-05	10.13817	3.312583	3.319444

STRBP	1.11E-03	5.424615	-0.73743	3.317623
ZCCHC4	1.36E-06	15.84913	6.181557	3.317469
NOL8	1.24E-05	11.3054	4.030466	3.317429
ZNF738	1.33E-04	7.762168	1.546998	3.316488
EXD2	3.01E-06	14.04644	5.430891	3.314311
PIK3AP1	4.61E-05	9.200598	2.670024	3.314002
TRAF3	3.29E-06	13.85638	5.344697	3.313427
ZNF160	6.55E-06	12.47118	4.669927	3.309491
SACS	1.35E-05	11.14935	3.939271	3.308988
QKI	4.47E-04	6.348926	0.24475	3.305154
MAF	5.07E-03	4.07847	-2.38456	3.303262
ZRANB2	2.10E-05	10.41321	3.489447	3.302611
UBE2W	6.86E-05	8.636305	2.250874	3.300195
CDC14A	8.73E-04	5.655834	-0.481	3.298941
RBAK	8.77E-05	8.302527	1.990321	3.298611
FKBP14	2.32E-05	10.25204	3.386413	3.296927
TSPAN5	2.69E-04	6.91499	0.793086	3.295498
KHDRBS1	8.53E-06	11.97506	4.406467	3.294897
BTN2A2	7.80E-06	12.14179	4.496388	3.293901
TACC1	2.46E-05	10.1561	3.324265	3.292294
FABP5	6.89E-06	12.3739	4.619234	3.290801
SYCP2	3.08E-05	9.803684	3.090601	3.28879
TAF15	6.05E-06	12.62495	4.749126	3.285271
IPO8	7.71E-06	12.16343	4.507957	3.284859
PARP4	2.58E-03	4.648298	-1.65446	3.283895
ZC3H11A	5.52E-05	8.941268	2.480615	3.283152
AAK1	6.18E-05	8.780757	2.360661	3.282655
DPY19L1	3.01E-06	14.04881	5.431956	3.280668
CASP10	6.05E-04	6.028317	-0.08319	3.278799
CEP63	3.99E-04	6.47142	0.366652	3.278578
ASH1L	3.89E-05	9.452826	2.849218	3.278284
SZRD1	2.21E-04	7.144024	1.004457	3.277826
KIAA1804	6.80E-04	5.907666	-0.21002	3.277157
REEP3	1.14E-04	7.958359	1.711148	3.276042
GNPTAB	7.82E-04	5.766048	-0.36136	3.275734
RNF19A	1.28E-04	7.815871	1.592301	3.275508
CENPL///GAS5	2.96E-06	14.08711	5.449149	3.275457
ATP2B1	2.52E-05	10.11961	3.300466	3.274128
VPS13A	5.03E-05	9.075167	2.57908	3.273333
YOD1	3.51E-05	9.607797	2.956942	3.273312
EIF2A	1.19E-03	5.358594	-0.81202	3.266907
GUSBP4	2.22E-04	7.134908	0.996155	3.264993
IPO8	4.87E-05	9.119688	2.611502	3.263826
MSL3P1	1.89E-05	10.58595	3.598012	3.263478
PRPF4B	2.21E-05	10.33294	3.438344	3.262877
PTEN	5.07E-05	9.061933	2.569412	3.261299
TNFRSF1A	2.55E-05	10.10313	3.289692	3.260075
TMEM120B	1.80E-04	7.386654	1.222146	3.259004

BICD2	2.80E-04	6.867774	0.748781	3.257311
CNEP1R1	4.98E-04	6.231431	0.126079	3.256218
ATG3	5.58E-05	8.924717	2.468344	3.256006
CACUL1	1.63E-04	7.510822	1.331152	3.254341
ACTBL2	3.18E-05	9.755178	3.057761	3.253492
PARP11	1.25E-03	5.309275	-0.86814	3.25306
AEBP2	1.02E-03	5.500972	-0.65192	3.252512
PRKAR2A	4.38E-03	4.198784	-2.22637	3.252054
KIAA1652	1.32E-04	7.775445	1.558225	3.251959
TTC3	9.18E-04	5.606668	-0.53491	3.2513
SSFA2	3.37E-06	13.80903	5.322997	3.250753
VPS50	3.42E-05	9.643647	2.981611	3.250361
TMX3	1.29E-05	11.23332	3.988517	3.250105
CLEC7A	2.51E-03	4.670995	-1.62639	3.248556
AP1AR	4.54E-04	6.331409	0.227167	3.248179
RIOK1	5.56E-05	8.931831	2.473621	3.247451
PNMAL1	7.60E-03	3.755285	-2.81989	3.246966
COPA	1.08E-03	5.447553	-0.71166	3.246499
DENND4A	6.04E-06	12.62813	4.750752	3.244977
IPO8	1.88E-05	10.59104	3.601185	3.243033
FAM135A	4.96E-05	9.093879	2.592726	3.242213
CCDC66	1.99E-04	7.268215	1.116667	3.241958
ANO6	7.41E-05	8.530164	2.169073	3.241288
GDF15	5.06E-05	9.0652	2.5718	3.240282
CHIC1	1.43E-04	7.671012	1.469448	3.239475
IPO8	6.25E-05	8.765381	2.349058	3.239323
ANKRD50	3.13E-06	13.9667	5.394906	3.239091
DIS3L2	1.52E-04	7.599239	1.407805	3.239001
TBL1XR1	8.91E-03	3.63205	-2.98973	3.238492
NSL1	2.98E-04	6.797734	0.682591	3.238472
KCNH4	4.74E-05	9.161537	2.641835	3.237923
CDK17	1.53E-05	10.93354	3.810816	3.237087
HSPA14	2.71E-06	14.27611	5.533137	3.234628
CDK13	1.28E-04	7.806479	1.584399	3.233755
BRWD3	3.14E-05	9.777474	3.072877	3.231763
MIGA1	7.34E-05	8.544208	2.179952	3.22952
ZNF37BP	8.49E-06	11.98263	4.410582	3.2289
SNX29	1.43E-04	7.669025	1.467749	3.228292
AGT	8.22E-03	3.694857	-2.90292	3.227419
HIP1	4.93E-04	6.243163	0.138006	3.221238
MIB1	3.31E-05	9.696429	3.017761	3.219278
CCNC	3.11E-05	9.789894	3.081282	3.219093
HNRNPA1L2	1.98E-06	14.9726	5.831005	3.217822
KIAA0368	1.18E-05	11.38413	4.07595	3.217638
CEP120	8.70E-06	11.93882	4.386734	3.215702
CEP152	2.01E-04	7.255556	1.105305	3.215244
STT3B	1.14E-05	11.45354	4.115758	3.214751
CXCR2	3.26E-05	9.720285	3.034033	3.213048

GNAQ	4.36E-05	9.280898	2.727602	3.212219
SEC22B	2.08E-04	7.217122	1.070704	3.210892
IPO7	3.56E-03	4.372044	-2.00235	3.210542
ARF1	3.86E-06	13.52551	5.191132	3.210364
RPL23AP7	1.70E-06	15.32394	5.974639	3.209713
PRPS2	2.98E-05	9.858226	3.127329	3.20876
SRP9	4.02E-05	9.400734	2.812606	3.208443
TTC33	9.36E-06	11.80477	4.313141	3.208109
MARS	1.63E-06	15.41621	6.011656	3.207448
ITGAV	4.47E-06	13.22481	5.047556	3.206861
MMRN2	2.36E-03	4.727427	-1.55693	3.206729
OSBPL11	6.65E-05	8.680266	2.284472	3.205511
UBE2Q2	1.14E-05	11.44868	4.112981	3.205233
UPRT	5.81E-06	12.7046	4.789706	3.204337
RAPGEF1	6.24E-05	8.767369	2.350559	3.204086
TCAF1	1.60E-04	7.533143	1.350578	3.200929
TXLNG	2.24E-06	14.69163	5.712996	3.20054
PPP6R3	3.81E-06	13.55348	5.204291	3.199177
FAM19A2	1.52E-03	5.125097	-1.08082	3.19912
PIK3R1	5.25E-04	6.175609	0.06909	3.197249
CYSLTR1	8.93E-05	8.27867	1.97132	3.196019
HESX1	2.62E-04	6.945753	0.821817	3.194624
ZNF770	2.60E-05	10.07282	3.269817	3.19347
GTF2I	3.69E-05	9.5295	2.902738	3.191677
TMPRSS2	3.46E-04	6.626965	0.518824	3.189789
P2RX7	9.17E-03	3.609981	-3.02037	3.189145
NBPF11	2.55E-06	14.40643	5.590246	3.187368
PITPNB	1.34E-05	11.16552	3.948786	3.187017
ZNF234	2.90E-05	9.897693	3.153775	3.187001
SMAD4	5.58E-05	8.926763	2.469862	3.185071
SMAD4	2.06E-04	7.225307	1.078086	3.184429
UPF1	5.78E-06	12.7137	4.794324	3.18434
ZNF638	1.02E-05	11.65608	4.2304	3.181885
BDP1	3.08E-04	6.758317	0.645091	3.179689
FGD4	6.29E-05	8.75654	2.342377	3.178702
ZNF124	1.82E-04	7.373219	1.210255	3.176953
PCMTD1	8.15E-05	8.401761	2.068808	3.176818
SSFA2	2.79E-05	9.96068	3.195756	3.173606
STK4	2.05E-05	10.45003	3.512749	3.173511
APC	1.61E-04	7.525065	1.343554	3.173411
GCOM1	9.14E-06	11.84833	4.337161	3.17338
TMEM260	1.85E-05	10.621	3.619811	3.172898
ASS1	4.62E-05	9.198458	2.668483	3.1724
UACA	7.24E-04	5.843971	-0.27776	3.172284
ALDH5A1	2.81E-05	9.948613	3.187735	3.172177
VPS29	9.56E-06	11.76567	4.291498	3.171864
H6PD	1.10E-04	8.001884	1.747063	3.169583
KIAA1109	1.76E-05	10.70314	3.670588	3.169183

GON4L	4.45E-04	6.352047	0.24788	3.166544
MINOS1P1	7.51E-04	5.807044	-0.31727	3.165429
DDB1	8.91E-06	11.89436	4.362432	3.162403
ANKRD12	4.29E-05	9.306665	2.745972	3.161259
PPP1R3D	7.55E-05	8.505718	2.150095	3.159902
TNFRSF1A	2.30E-05	10.26258	3.393207	3.159396
TCF25	1.58E-05	10.88531	3.781726	3.159167
WASF3	1.07E-04	8.046441	1.783645	3.157895
PAICS	1.70E-05	10.75916	3.704983	3.157675
NEURL1	2.40E-06	14.5436	5.649662	3.157079
CASC4	1.08E-05	11.54456	4.167558	3.156987
SLC40A1	4.29E-04	6.393653	0.289475	3.156689
TET2	2.62E-04	6.944044	0.820224	3.156427
APC	8.49E-05	8.346929	2.025548	3.155375
NBPF10	3.23E-05	9.732656	3.042456	3.154946
KMO	1.64E-05	10.81726	3.740448	3.154202
ZNF609	2.85E-05	9.92818	3.174129	3.153233
SLC39A10	2.87E-05	9.914519	3.165016	3.151257
SMAD4	5.82E-05	8.865353	2.424146	3.150847
SMARCA1	1.92E-04	7.307424	1.151751	3.149457
EIF4H	2.48E-04	7.009511	0.881024	3.148483
SOGA1	2.55E-04	6.975417	0.849421	3.148212
ZBED3-AS1	2.63E-05	10.04929	3.254353	3.148157
AGT	8.34E-03	3.683021	-2.91924	3.147781
MTMR4	8.95E-04	5.631828	-0.50728	3.146965
TNFRSF1A	9.59E-05	8.18466	1.895943	3.146207
TNFRSF1A	3.50E-05	9.61091	2.959088	3.145823
KPNA3	1.24E-04	7.849063	1.620161	3.145697
AK3	9.59E-05	8.185174	1.896358	3.145525
MAN1A1	4.31E-06	13.29949	5.083582	3.145371
ANKRD44	1.03E-04	8.096577	1.824583	3.144812
TGFBR2	4.79E-05	9.145394	2.630151	3.143648
MDFIC	2.56E-06	14.39787	5.586518	3.14258
C2CD5	7.99E-05	8.429126	2.090298	3.142026
USP6	3.35E-04	6.664094	0.554721	3.14193
SYNJ2	3.35E-03	4.42398	-1.93607	3.141921
PHF10	2.36E-05	10.2232	3.3678	3.141895
PPP2R5C	1.91E-05	10.56728	3.586368	3.141317
ZNF254	2.38E-06	14.56408	5.658474	3.140882
ACBD3	7.80E-06	12.13992	4.49539	3.139678
TANC2	5.60E-05	8.922039	2.466356	3.139534
MIA3	4.89E-06	13.04417	4.95941	3.13947
LYRM7	3.38E-06	13.80215	5.319838	3.135986
FAM91A1	2.11E-05	10.40481	3.484116	3.135511
NME2P1	3.30E-06	13.85565	5.344364	3.134832
PIK3C2A	9.97E-06	11.68881	4.248713	3.133212
PARP14	2.50E-05	10.13204	3.308585	3.132506
POLR3E	1.15E-03	5.387847	-0.77889	3.132299

SPOPL	1.35E-05	11.15674	3.943619	3.131147
MTHFR	2.92E-06	14.11296	5.46072	3.129547
SMAD4	6.53E-05	8.704542	2.302955	3.127111
SCLT1	1.62E-04	7.514543	1.334394	3.125953
CNOT6L	5.85E-06	12.68952	4.782046	3.125544
USF2	2.55E-05	10.10294	3.289564	3.125289
PREPL	2.10E-05	10.40851	3.486464	3.124658
PROS1	3.81E-04	6.520525	0.415006	3.12092
TMPO	2.18E-04	7.158394	1.017526	3.116573
FAM160B1	3.13E-04	6.741786	0.629311	3.116338
APC	8.68E-04	5.661339	-0.47499	3.115212
SLC39A6	8.46E-05	8.350637	2.028482	3.114919
KIAA1841	7.76E-05	8.467494	2.120317	3.11413
RND3	7.47E-05	8.518897	2.160332	3.112948
BLOC1S6	4.34E-04	6.379785	0.275634	3.112916
F2RL1	7.17E-03	3.801255	-2.75706	3.112295
KLHL8	3.84E-04	6.514055	0.408651	3.110249
SPRYD7	1.14E-04	7.963473	1.715376	3.109016
SIPA1L3	2.56E-05	10.09469	3.284165	3.107383
ANKRD36B	8.74E-06	11.93051	4.382198	3.106964
DNAJC27	2.37E-05	10.21448	3.362157	3.106937
ZNF569	8.18E-04	5.720503	-0.4106	3.105625
SMAD4	1.31E-04	7.786176	1.567286	3.1056
BLOC1S6	6.86E-05	8.636746	2.251211	3.104445
NAA16	1.43E-05	11.05235	3.881874	3.10147
MTHFR	1.58E-05	10.88613	3.782221	3.100864
TNFRSF1A	5.02E-05	9.077927	2.581094	3.099022
DIXDC1	1.18E-04	7.919469	1.678904	3.097673
LINC00909	1.34E-05	11.16169	3.946532	3.096354
DIO3OS	8.82E-05	8.294877	1.984233	3.096198
SMAD4	7.98E-05	8.430276	2.091199	3.095921
CRYBG3	3.65E-04	6.568387	0.461856	3.095317
OR8D2	7.67E-06	12.17219	4.512632	3.095201
SMARCA2	3.65E-05	9.547472	2.91522	3.094051
LY86-AS1	9.09E-05	8.255025	1.952437	3.093704
ZEB2	3.07E-06	14.00636	5.412833	3.092216
DTWD2	3.59E-06	13.67555	5.261333	3.091295
SLC31A1	2.18E-05	10.34786	3.447875	3.090952
SMAD4	1.67E-04	7.47576	1.300533	3.090661
ATP6V1C1	2.62E-04	6.942822	0.819084	3.090535
ZNF664-FAM101/	4.11E-06	13.39599	5.129769	3.090231
PARP14	2.65E-05	10.03777	3.246765	3.088555
CSGALNACT2	3.32E-05	9.689802	3.013233	3.088393
E2F4	8.48E-06	11.98586	4.412339	3.08787
MAP4K5	6.04E-05	8.813552	2.385341	3.087448
FBXL17	6.24E-03	3.912006	-2.60692	3.08684
ZNF813	1.26E-06	16.03088	6.251075	3.086683
ZNF365	4.89E-03	4.10814	-2.34535	3.08501

FAM175A	5.05E-05	9.068152	2.573957	3.084994
SEPT2	1.45E-05	11.03473	3.871388	3.084984
KIAA0232	6.19E-06	12.58037	4.726279	3.084775
FAM193A	1.59E-05	10.87401	3.774893	3.084405
MTHFR	2.53E-05	10.11031	3.294386	3.083465
KBTBD8	5.63E-04	6.102786	-0.00585	3.082709
MAP7D3	1.15E-04	7.951545	1.705509	3.082007
MTHFR	1.79E-05	10.67405	3.652657	3.08143
CLK3	1.79E-04	7.393814	1.228475	3.078884
ZNF137P	1.12E-05	11.47574	4.128434	3.076414
USP38	2.66E-04	6.925484	0.802899	3.074476
ZCCHC11	2.94E-05	9.878137	3.140684	3.073822
TOP1P2	3.95E-05	9.428262	2.83198	3.071983
LOC100190986	4.75E-04	6.283372	0.178753	3.071876
SEPT7P2	1.87E-05	10.59928	3.606311	3.0715
RAB1A	1.78E-04	7.400799	1.234645	3.071214
ZNF451	3.53E-04	6.606201	0.498677	3.070973
KRAS	1.52E-04	7.598299	1.406995	3.070896
RBM34	5.10E-06	12.95821	4.916947	3.070769
SMAD4	1.46E-04	7.646679	1.448608	3.070464
ELN	1.41E-04	7.685616	1.481928	3.069062
KIAA0825	1.26E-04	7.833714	1.607292	3.06795
FAM120A	2.02E-04	7.247701	1.098247	3.066392
SASH1	4.06E-03	4.26064	-2.14588	3.065677
DDX6	4.73E-06	13.11042	4.991907	3.065417
KL	3.17E-05	9.759123	3.060438	3.064969
TWSG1	6.95E-05	8.618083	2.236899	3.064314
STK17B	2.78E-06	14.21644	5.506773	3.064232
TBC1D8B	1.16E-03	5.378037	-0.78999	3.063466
CLN5	6.09E-05	8.801708	2.376438	3.06314
POLK	2.74E-04	6.892011	0.771555	3.062826
TNFRSF1A	3.63E-05	9.555532	2.92081	3.061799
FAM135A	5.79E-04	6.07419	-0.03546	3.060714
MPP7	4.79E-03	4.12478	-2.32342	3.060123
PDE12	2.28E-05	10.28084	3.40495	3.05837
STX16	6.34E-04	5.980303	-0.13344	3.056696
UEVLD	2.41E-06	14.53214	5.644728	3.056292
SUCNR1	5.90E-04	6.053391	-0.05707	3.054549
RPL36A	2.11E-06	14.82825	5.770732	3.054109
GMCL1	2.12E-05	10.39641	3.478786	3.051126
EIF2AK2	9.96E-06	11.69199	4.250489	3.051044
LRRC37A2	6.07E-06	12.6199	4.746542	3.050548
NFE2L3	7.76E-04	5.773782	-0.35302	3.05019
NETO2	1.06E-03	5.466561	-0.69036	3.050129
KLF12	3.40E-04	6.649655	0.54078	3.04976
CALM1	3.55E-04	6.600348	0.492989	3.048771
UBE2E1	3.94E-03	4.285881	-2.11319	3.048256
APC	1.41E-04	7.692871	1.48812	3.047618

GALNT5	7.14E-04	5.85745	-0.26338	3.047341
RAPGEF6	5.06E-05	9.06722	2.573276	3.047051
VPS50	2.93E-05	9.884389	3.144873	3.046733
COL4A3	1.05E-03	5.479147	-0.67628	3.045801
ZNF727	3.71E-06	13.60945	5.230523	3.0456
SEC23IP	1.81E-05	10.65591	3.641441	3.044806
ZMAT3	3.29E-05	9.703191	3.022377	3.044506
RGPD1	6.14E-03	3.924561	-2.59001	3.044371
HMGCS1	8.28E-04	5.708496	-0.42363	3.042645
ABHD10	1.30E-05	11.21356	3.976963	3.042478
AGT	3.86E-03	4.303425	-2.09053	3.041034
ZNF451	5.20E-06	12.92192	4.898924	3.038542
IL17A	2.69E-03	4.610929	-1.70085	3.037038
PTPRC	9.36E-04	5.587483	-0.55603	3.036953
MANEA	1.15E-04	7.944155	1.699388	3.036416
SCOC	2.17E-05	10.36121	3.456392	3.034469
ZC3H11A	1.70E-05	10.75936	3.705105	3.03332
C9orf72	1.32E-04	7.767824	1.551783	3.033193
GTF2IRD2	7.73E-06	12.1587	4.505431	3.032825
APC	6.80E-05	8.64983	2.261228	3.032115
GPR89B	1.43E-05	11.05258	3.882008	3.031812
REEP3	1.94E-05	10.54019	3.569437	3.030277
CHRNA5	9.22E-04	5.601931	-0.54012	3.0296
EMCN	1.28E-03	5.285845	-0.89493	3.02956
UBE3C	3.63E-04	6.575202	0.468505	3.028738
RSRC1	5.89E-05	8.850327	2.412913	3.028587
MIOS	4.05E-05	9.391918	2.806391	3.027409
MAP9	4.75E-05	9.155779	2.63767	3.026488
C2orf69	8.31E-06	12.02202	4.431937	3.025883
YIPF5	9.72E-05	8.166498	1.881288	3.025718
NUPR1	1.14E-03	5.396005	-0.76968	3.025017
MALAT1	2.26E-05	10.29171	3.411933	3.024983
DYRK1A	2.70E-03	4.608463	-1.70392	3.023984
GABPA	1.17E-05	11.39957	4.084826	3.022801
TCP1	1.51E-04	7.601781	1.409997	3.021051
LPAR4	2.60E-04	6.952164	0.82779	3.019655
RPS2P32	9.68E-05	8.172565	1.886187	3.018322
AKAP13	5.04E-04	6.219668	0.114103	3.018247
GPATCH11	4.70E-04	6.293326	0.188808	3.018144
RAB33B	1.01E-05	11.67043	4.238435	3.01783
BRCC3	2.01E-04	7.254392	1.10426	3.016581
RSF1	8.63E-05	8.325017	2.008185	3.016339
ITGB1	1.01E-04	8.120301	1.843874	3.016115
TGFBR1	5.17E-05	9.035174	2.549821	3.015886
SRSF4	7.61E-06	12.18623	4.520115	3.015171
ASXL2	4.56E-05	9.216916	2.681766	3.015122
CLEC7A	3.77E-04	6.532474	0.426728	3.013874
SNX13	8.88E-06	11.90062	4.365857	3.013224

TCF4	2.72E-05	9.997845	3.220397	3.01279
SP3	2.10E-04	7.201582	1.05667	3.012617
IPO8	1.02E-03	5.501074	-0.65181	3.012489
ZNF451	5.60E-04	6.10936	0.000943	3.011267
PLCB1	4.81E-04	6.270497	0.165727	3.010061
ZNF525	1.40E-05	11.08752	3.902749	3.009855
GXYLT1	5.20E-05	9.025356	2.542619	3.009623
TNPO1	3.19E-05	9.752518	3.055955	3.009333
GAB1	9.33E-05	8.220845	1.925052	3.008772
ANKIB1	3.14E-04	6.738328	0.626006	3.005079
IGF2R	8.98E-05	8.270943	1.965154	3.004858
NEMF	2.14E-04	7.178674	1.035932	3.004589
HERC4	7.98E-06	12.09711	4.472433	3.003291
CRCP	9.68E-06	11.74236	4.278554	3.001023
KANK2	2.91E-05	9.893495	3.150967	2.999696
PLGLB1	2.56E-04	6.972281	0.846507	2.999349
STXBP3	2.90E-03	4.547715	-1.7798	2.999247
GLIPR1	2.60E-04	6.952722	0.828311	2.999142
TRIM44	1.79E-04	7.397907	1.232091	2.998474
ANP32D	5.09E-05	9.05648	2.565424	2.997267
LNPEP	1.92E-05	10.5557	3.579138	2.996372
F11R	5.38E-04	6.150314	0.043136	2.99622
SPDYE1	4.79E-06	13.08528	4.979599	2.995393
NFATC2IP	1.90E-05	10.57313	3.590021	2.995318
TNFRSF1A	4.17E-05	9.347405	2.774913	2.995049
WTAP	3.01E-05	9.842688	3.116887	2.992949
KIF3B	3.90E-06	13.5023	5.180186	2.991001
FOPNL	3.21E-05	9.742614	3.049228	2.990943
FBXO45	8.93E-05	8.279441	1.971935	2.990846
ADIPOR2	4.96E-03	4.095953	-2.36144	2.989568
RABL3	8.93E-06	11.89075	4.360454	2.98817
TTC37	2.64E-03	4.626761	-1.68117	2.988022
MAN1A1	2.31E-05	10.25553	3.38866	2.987918
RANBP9	6.47E-05	8.717087	2.312486	2.987783
CPN2	1.18E-05	11.39188	4.080406	2.987472
HPS3	3.56E-06	13.69144	5.268711	2.98621
CLN8	8.09E-05	8.412021	2.076873	2.985148
RPL21	2.24E-06	14.69579	5.714764	2.984786
DKFZP434C153	2.00E-06	14.95163	5.822295	2.983776
POLI	6.03E-05	8.817133	2.388031	2.983066
TCF4	5.58E-06	12.78289	4.829306	2.982529
MTHFR	1.34E-05	11.16925	3.950978	2.981816
OGN	1.83E-04	7.365657	1.203555	2.98111
LINC00167	1.31E-04	7.785181	1.566446	2.979504
KDM1B	1.18E-05	11.38358	4.075634	2.978386
GTF2H3	2.62E-05	10.0585	3.260406	2.97802
ATF6	9.86E-05	8.147532	1.865952	2.97679
MANEA	4.23E-05	9.327127	2.760524	2.976259

TM4SF4	1.78E-04	7.400865	1.234703	2.975189
MTHFR	2.12E-05	10.39695	3.479131	2.973277
HIF1A	3.03E-05	9.832445	3.109995	2.97301
CXorf21	4.54E-04	6.331978	0.227738	2.970678
TBL1Y	4.72E-05	9.167682	2.646278	2.968868
UBE3C	2.40E-05	10.19527	3.349711	2.968774
HERC4	3.51E-05	9.606198	2.95584	2.968276
ZFYVE16	1.63E-04	7.512697	1.332785	2.967822
ITPR2	1.05E-04	8.071646	1.804255	2.967417
PARP14	1.04E-05	11.61984	4.210051	2.966798
CD2AP	3.16E-04	6.731391	0.619372	2.966323
ITGB1	1.62E-04	7.514941	1.33474	2.965629
HSD17B13	2.88E-04	6.83605	0.71887	2.965478
FAM210A	4.40E-06	13.25825	5.063717	2.965365
CNOT6L	9.19E-06	11.83808	4.331519	2.964668
MTHFR	1.86E-05	10.60786	3.611648	2.96282
METTL2B	2.56E-05	10.09257	3.282773	2.962503
LOC100190986	4.43E-04	6.357389	0.253232	2.962455
ARFIP1	1.42E-03	5.1847	-1.01146	2.962057
SRGAP2B	7.87E-04	5.759369	-0.36856	2.962041
TNFRSF1A	5.42E-05	8.966777	2.499485	2.960281
ERRFI1	4.11E-05	9.370394	2.791188	2.959987
LRRC2	2.92E-04	6.817692	0.701509	2.958837
BBS5	3.02E-05	9.83454	3.111405	2.958517
RPL21	3.85E-05	9.467515	2.859506	2.958375
DMXL1	1.15E-04	7.944961	1.700055	2.958011
PDK4	1.04E-03	5.481926	-0.67318	2.957382
SLMAP	8.62E-05	8.326448	2.009321	2.957221
YWHAZ	7.05E-05	8.599152	2.222351	2.957217
HIF1A	2.38E-05	10.21087	3.359818	2.956436
TANK	1.44E-04	7.663984	1.463435	2.95593
TMED7	2.93E-06	14.10725	5.458163	2.955685
RALA	3.59E-05	9.573369	2.933164	2.955657
RUFY3	5.24E-05	9.01695	2.536446	2.954598
MTDH	8.77E-05	8.303319	1.99095	2.95437
ALG1	1.96E-03	4.893048	-1.3558	2.953623
ZNF248	6.37E-05	8.73865	2.328839	2.953593
CSNK1G1	1.49E-04	7.623506	1.428706	2.953205
UBXN4	8.46E-05	8.351373	2.029064	2.953154
GUSBP3///SMA4	3.41E-05	9.651458	2.986974	2.95267
ABCD4	1.76E-06	15.24535	5.942881	2.952396
ZNF615	8.62E-06	11.95567	4.395918	2.952108
UBTD2	9.58E-04	5.565104	-0.58074	2.95208
ESF1	8.22E-06	12.04214	4.442815	2.950636
IMPA1	5.69E-05	8.899322	2.449472	2.949581
RAPH1	5.70E-04	6.089394	-0.01971	2.949443
LSG1	4.91E-05	9.110325	2.604697	2.948173
APC	1.91E-04	7.315088	1.158589	2.947645

LOC286382	3.44E-04	6.633882	0.525523	2.947445
CPEB4	2.75E-04	6.888627	0.76838	2.946711
ZXDC	6.19E-05	8.780155	2.360206	2.945173
UPF3B	1.88E-05	10.59381	3.60291	2.944919
KIAA1109	2.52E-05	10.11728	3.298947	2.944917
TCF19	9.10E-05	8.254566	1.95207	2.944152
RIN3	1.28E-05	11.24699	3.996495	2.943716
SCOC	1.73E-04	7.43437	1.264224	2.943654
GRK3	3.91E-05	9.442739	2.842145	2.943544
MTDH	1.10E-04	7.999495	1.745096	2.942759
MTDH	1.50E-04	7.614495	1.420952	2.942576
ITGB1	1.74E-04	7.432417	1.262507	2.94161
CEACAM6	2.81E-04	6.86469	0.745878	2.940055
CLCC1	4.04E-05	9.393689	2.807639	2.938921
FEM1C	3.33E-04	6.672574	0.562897	2.938914
LRRC15	8.33E-06	12.01805	4.429793	2.938604
DDX17	3.54E-04	6.601724	0.494326	2.938151
APC	8.57E-05	8.333814	2.015161	2.937652
SLC28A3	1.00E-03	5.522997	-0.62741	2.936932
SMAD4	3.48E-04	6.622694	0.514684	2.936895
SMIM13	1.12E-04	7.979761	1.72883	2.93529
EOGT	4.32E-05	9.295367	2.737923	2.935047
TLK2	2.48E-04	7.005507	0.877319	2.934582
MTDH	1.45E-04	7.652287	1.453416	2.933303
UBE2Q2	3.26E-05	9.719242	3.033323	2.93227
MTDH	1.39E-04	7.709913	1.502644	2.93223
APC	3.77E-04	6.534031	0.428255	2.931713
UBE2G2	2.13E-04	7.185791	1.042381	2.931466
HIF1A	4.58E-05	9.20975	2.676612	2.931453
MARCH1	3.34E-05	9.679427	3.006139	2.930471
LINC01420	9.59E-05	8.184438	1.895764	2.930143
ITGB1	2.31E-04	7.088717	0.953947	2.929641
ITGB1	1.89E-04	7.329482	1.171416	2.929311
TMEM30A	5.78E-05	8.876299	2.432317	2.928797
FNTA	8.88E-04	5.63964	-0.49872	2.928276
FAM200A	1.24E-04	7.851871	1.622514	2.927563
COMMD2	7.15E-05	8.580389	2.207901	2.927534
BCL2L11	4.98E-04	6.231582	0.126232	2.927231
ADSS	4.16E-05	9.350638	2.777205	2.926069
EYA1	3.28E-05	9.707855	3.02556	2.924931
GNG2	5.18E-06	12.9298	4.90284	2.923498
RND3	2.84E-05	9.933591	3.177735	2.92326
MTDH	1.11E-04	7.995683	1.741957	2.922006
ABCC13	1.93E-05	10.55087	3.576118	2.921946
PRKCB	5.57E-04	6.114156	0.005894	2.92154
CEP57	5.78E-04	6.075116	-0.0345	2.921363
ABCD3	1.59E-04	7.539783	1.356347	2.920976
FAM120A	9.10E-05	8.25448	1.952001	2.920955

ATRX	1.34E-04	7.751353	1.53784	2.919977
TNFRSF1A	6.59E-05	8.691528	2.293052	2.919845
PCGF2	7.26E-06	12.27682	4.568177	2.919241
CHD9	1.91E-03	4.914157	-1.33046	2.91867
DDX6	6.27E-04	5.991249	-0.12196	2.918568
SKAP2	1.90E-05	10.57362	3.590325	2.915989
PICALM	3.95E-04	6.482717	0.377802	2.915348
EEA1	3.83E-04	6.517529	0.412064	2.914962
MFSD14A	4.79E-05	9.144629	2.629596	2.912297
CUL2	9.79E-06	11.72292	4.267741	2.911318
RBM26	2.58E-03	4.648222	-1.65456	2.911234
BTAF1	1.07E-05	11.56569	4.179513	2.90903
DNAH3	1.84E-03	4.951334	-1.28598	2.907913
ARL4A	1.19E-05	11.38009	4.073623	2.906793
PCMTD1	1.50E-05	10.97026	3.832868	2.90664
CERS6	6.26E-05	8.762861	2.347154	2.906468
NFIC	5.27E-04	6.173334	0.06676	2.906024
MIER1	6.64E-05	8.68144	2.285366	2.905878
CAPRIN1	3.39E-03	4.41195	-1.95139	2.904415
ATP2A2	1.82E-05	10.65126	3.638569	2.904394
C8orf88	2.66E-04	6.92422	0.801718	2.904131
CNST	3.54E-05	9.593569	2.947126	2.903955
SMCHD1	5.75E-06	12.72225	4.798661	2.903605
AIDA	3.18E-05	9.755224	3.057793	2.90302
FAM21A	3.44E-04	6.635501	0.527091	2.902729
MTDH	2.35E-04	7.071319	0.937988	2.902052
TRPM7	5.83E-05	8.863916	2.423072	2.901638
FGD4	6.70E-04	5.922416	-0.19442	2.901415
MTHFR	1.77E-05	10.69588	3.666119	2.900948
C2orf61	1.15E-04	7.943824	1.699114	2.900311
UBE3A	1.42E-05	11.06154	3.887335	2.899087
APC	3.25E-05	9.723017	3.035894	2.898768
HIF1A	2.43E-05	10.17405	3.335943	2.898353
UBE2W	5.36E-05	8.982606	2.511168	2.898237
CHRAC1	1.31E-05	11.20039	3.969252	2.897912
CEP97	4.34E-04	6.379976	0.275824	2.897819
ZBTB10	1.89E-05	10.58066	3.594713	2.897603
HES2	3.59E-04	6.587764	0.480745	2.895925
HIF1A	3.69E-05	9.529371	2.902649	2.895868
ZNF24	1.01E-04	8.111759	1.836934	2.893904
HSD17B12	1.03E-05	11.6313	4.216495	2.89329
PRKD3	5.37E-05	8.979833	2.509123	2.893237
PTER	2.11E-04	7.194758	1.050497	2.893235
PLEKHA8	4.71E-04	6.292269	0.187742	2.892571
ITGB1	2.80E-04	6.86778	0.748786	2.892011
PPA2	1.89E-05	10.58828	3.599463	2.891197
LMAN1	1.24E-05	11.30758	4.031734	2.890261
PIAS2	4.60E-05	9.205256	2.673378	2.889933

VN1R3	3.33E-05	9.687334	3.011546	2.889245
AGAP9	1.25E-05	11.28773	4.020208	2.888171
FAM209B	6.64E-04	5.931986	-0.1843	2.887757
SCAMP1	6.81E-05	8.646663	2.258805	2.887314
RPL9	1.17E-04	7.928501	1.686406	2.886055
MIATNB	1.50E-03	5.139678	-1.0638	2.88397
FLJ11710	5.55E-05	8.932851	2.474377	2.883623
RNF213	4.45E-04	6.353015	0.24885	2.883118
EXTL3	1.57E-04	7.559123	1.373125	2.882585
KIAA1107	8.73E-05	8.309796	1.9961	2.882333
PPP4R2	8.54E-04	5.678462	-0.4563	2.882078
CACUL1	4.50E-05	9.237533	2.69657	2.881025
NEK7	2.12E-05	10.39838	3.480036	2.880837
HIF1A	2.75E-05	9.980456	3.20888	2.880722
MME	7.76E-05	8.467578	2.120382	2.880201
ELMOD3	1.06E-05	11.57405	4.184237	2.878405
NAA30	3.70E-05	9.527927	2.901645	2.878347
ARV1	2.22E-04	7.137971	0.998945	2.877579
ZBTB10	9.72E-05	8.167204	1.881859	2.877486
ZNF562	1.67E-05	10.78983	3.72373	2.877314
ZNF85	3.26E-05	9.720417	3.034123	2.876579
AP5M1	9.54E-05	8.19099	1.901044	2.875985
AK4	2.49E-03	4.679125	-1.61635	2.875936
TMBIM1	1.99E-03	4.878667	-1.3731	2.875466
TALDO1	2.64E-05	10.04792	3.253451	2.875163
ALMS1	1.06E-05	11.58404	4.189882	2.874785
MTHFR	1.53E-05	10.94104	3.815327	2.873986
UTP15	2.48E-04	7.008807	0.880373	2.873779
MPZL2	2.29E-03	4.75165	-1.52725	2.873642
APAF1	4.31E-03	4.211016	-2.21041	2.873618
ITGB1	2.16E-04	7.168785	1.026962	2.873012
KDM4A	3.15E-05	9.770703	3.06829	2.872421
SERPINB9	7.58E-05	8.499224	2.145045	2.872331
HIST1H3C	6.39E-05	8.735888	2.326747	2.872072
IMPAD1	2.84E-03	4.563354	-1.76021	2.871695
PDE4C	1.11E-04	7.998077	1.743929	2.871544
ATP11A	1.05E-03	5.478716	-0.67676	2.871158
ITGB1	2.48E-04	7.005923	0.877704	2.870229
PHC3	3.01E-05	9.84149	3.116082	2.869671
TNFRSF1A	1.36E-04	7.73379	1.522944	2.869588
USP34	1.09E-04	8.014668	1.757578	2.869077
CCL13	2.89E-06	14.13619	5.471091	2.868904
APOA1	6.04E-03	3.937658	-2.57239	2.86735
RBM5	1.22E-04	7.870911	1.638442	2.867161
SIGLEC11	4.24E-03	4.225391	-2.19168	2.865752
SPAG1	1.22E-03	5.334699	-0.83917	2.865087
KIAA1429	4.37E-05	9.277647	2.72528	2.864894
TTC6	4.28E-04	6.395788	0.291603	2.864451

PPM1A	6.93E-05	8.623053	2.240713	2.864023
ZFAND5	7.24E-05	8.562817	2.194341	2.862345
HCAR1	6.10E-04	6.020066	-0.0918	2.86196
PMAIP1	1.18E-04	7.909785	1.670852	2.861723
UBQLN1	2.88E-03	4.552301	-1.77405	2.860377
ZNF253	4.98E-05	9.087346	2.587965	2.860303
HIF1A	3.97E-05	9.420958	2.826845	2.860226
CAPN7	1.64E-05	10.82466	3.744954	2.859947
MRPS10	3.37E-05	9.666682	2.997412	2.859135
PMAIP1	4.86E-04	6.257978	0.153043	2.858302
PSMD5	2.23E-05	10.31671	3.427961	2.856921
ZNF320	6.52E-04	5.95038	-0.1649	2.85687
UPB1	3.39E-05	9.658364	2.991711	2.8568
OIP5-AS1	1.17E-05	11.40817	4.089768	2.856278
MCM3AP-AS1	1.59E-04	7.541212	1.357589	2.855893
MTPN	7.64E-06	12.17848	4.515987	2.854862
CCDC88A	8.55E-05	8.336701	2.017449	2.854661
CANX	1.16E-04	7.93326	1.690354	2.8541
TMEM68	3.56E-05	9.586143	2.941997	2.853427
TMEM135	2.55E-05	10.10333	3.289821	2.853327
TMX4	1.47E-05	11.00214	3.851946	2.851821
UTP23	8.48E-06	11.98475	4.411735	2.851796
ESPN	1.82E-04	7.373713	1.210694	2.851614
RNFT1	6.61E-06	12.45268	4.660321	2.851217
EEF1D	1.11E-04	7.993777	1.740387	2.850307
USP22	3.34E-05	9.681456	3.007526	2.849688
NEXN	8.86E-04	5.64136	-0.49684	2.849653
CHD2	1.05E-04	8.070686	1.803472	2.849277
CCP110	3.24E-06	13.89052	5.360288	2.848659
KIAA1109	2.35E-05	10.23084	3.372733	2.848593
ZNF251	8.61E-05	8.328097	2.010629	2.847998
JOSD1	2.90E-05	9.89995	3.155284	2.847734
NFE2L2	1.13E-04	7.974505	1.724492	2.847614
IPO7	1.35E-04	7.742771	1.530565	2.846468
MTHFR	1.59E-05	10.87563	3.775875	2.843939
PTGS2	1.70E-04	7.454864	1.282225	2.842565
CNTLN	5.62E-04	6.105359	-0.00319	2.842563
EP400	7.99E-05	8.429014	2.090209	2.841657
TRIM29	5.97E-04	6.041337	-0.06962	2.841194
MTHFR	8.92E-06	11.89124	4.360722	2.840895
HIF1A	5.02E-05	9.075938	2.579643	2.840657
SC5D	1.09E-05	11.52784	4.158075	2.839591
MDM1	3.02E-04	6.780347	0.666071	2.839305
PMAIP1	1.74E-04	7.428993	1.259495	2.839147
ITGB1	2.44E-04	7.025467	0.89577	2.839071
SLC25A43	2.70E-04	6.910801	0.789166	2.837967
GLIDR	9.53E-05	8.192272	1.902077	2.837938
STAU2	7.01E-05	8.607626	2.228867	2.837746

CRYZ	2.93E-03	4.536799	-1.79349	2.836927
UBL3	1.85E-04	7.35536	1.194421	2.834683
ZNF585A	2.21E-05	10.33109	3.437161	2.834363
PDLIM5	1.70E-04	7.458539	1.285448	2.83405
RBM12B	8.31E-05	8.375189	2.047878	2.833848
FCHO2	2.97E-05	9.862777	3.130384	2.833841
MTDH	1.96E-04	7.283041	1.129953	2.833784
SPATA18	2.49E-03	4.67932	-1.61611	2.83341
PIKFYVE	7.26E-05	8.558854	2.191279	2.831391
MSMO1	2.36E-05	10.22619	3.369729	2.831341
ZNF641	4.11E-04	6.439007	0.334574	2.830413
SERPINB2	1.63E-03	5.057945	-1.15958	2.829193
SSR1	1.64E-04	7.505028	1.3261	2.829188
LINC01572	2.45E-03	4.692873	-1.5994	2.828678
AKIRIN1	2.78E-05	9.962438	3.196923	2.827777
ZDHHC13	2.91E-05	9.895248	3.15214	2.827601
NAV1	1.59E-03	5.082508	-1.1307	2.826659
N4BP2L2	1.49E-05	10.98323	3.840638	2.826598
KCTD9	2.12E-05	10.39653	3.478862	2.826554
EFL1	1.03E-05	11.63163	4.216679	2.825213
RAB6A	6.69E-05	8.671407	2.277714	2.824981
UBR1	9.24E-06	11.82863	4.326309	2.824908
APOL4	3.06E-05	9.813785	3.097419	2.824501
MTDH	1.52E-04	7.597569	1.406365	2.824116
NOL10	8.08E-05	8.412768	2.077459	2.824085
ZBTB38	1.61E-03	5.072076	-1.14295	2.823389
SRGAP2C	1.24E-03	5.314286	-0.86243	2.82294
LNX1	3.96E-04	6.479646	0.374772	2.822249
PAPOLG	9.64E-06	11.75104	4.283377	2.821986
RIF1	9.40E-05	8.210592	1.916816	2.8218
BEND2	1.78E-05	10.68557	3.659762	2.821664
CEP192	2.24E-04	7.125597	0.987666	2.821378
ZNF277	2.01E-04	7.254255	1.104137	2.820809
TRMT5	4.93E-06	13.02961	4.952239	2.820535
DIMT1	1.55E-04	7.568723	1.381439	2.82034
VNN1	1.65E-04	7.496793	1.318916	2.818838
ODF2L	6.99E-04	5.879439	-0.23997	2.818355
SIPA1L2	3.12E-05	9.785337	3.0782	2.817974
TPMT	1.06E-04	8.056649	1.791999	2.817362
CPD	2.96E-05	9.868151	3.13399	2.81717
IPO8	5.93E-04	6.048472	-0.06219	2.81702
ITGB1	4.97E-04	6.233502	0.128185	2.815791
SLC8A1	2.30E-04	7.098017	0.962464	2.815662
PA2G4	6.39E-06	12.51848	4.694411	2.81469
QKI	7.35E-04	5.828447	-0.29435	2.814467
LOC401320	6.92E-04	5.889555	-0.22923	2.814114
IPO8	7.47E-04	5.811814	-0.31216	2.814063
FAR1	1.32E-04	7.771042	1.554503	2.811895

HPS3	6.07E-06	12.61871	4.745932	2.811855
TMEM241	1.06E-04	8.053742	1.789621	2.811588
LRP12	3.47E-03	4.392493	-1.9762	2.81135
RND3	1.34E-03	5.242632	-0.94454	2.811183
CLCN5	1.94E-04	7.295407	1.141015	2.811042
PIGA	3.27E-05	9.715008	3.030438	2.810172
PTPN11	3.14E-03	4.479564	-1.86559	2.809738
MPV17L	2.24E-05	10.30475	3.420297	2.809493
PKN2	9.29E-05	8.226228	1.929372	2.80945
BMPR2	5.15E-05	9.041692	2.554598	2.808682
RAB2A	4.92E-05	9.105539	2.601215	2.808208
RBAK	4.91E-05	9.110017	2.604472	2.808142
EIF5B	1.06E-05	11.57821	4.186588	2.807461
B4GALT6	1.09E-05	11.53286	4.160921	2.807441
RNFT1	7.31E-04	5.834395	-0.28799	2.807382
XAF1	5.67E-06	12.74991	4.812659	2.807095
CLN8	4.30E-05	9.302789	2.743212	2.806087
CGGBP1	7.00E-06	12.34439	4.603763	2.805217
P4HA1	4.20E-04	6.415629	0.311358	2.804877
ANKRD13D	5.27E-04	6.172713	0.066123	2.804797
TIA1	1.34E-05	11.16199	3.946711	2.803186
SLC25A52	5.64E-05	8.910742	2.457965	2.802999
PLD1	1.75E-03	4.99333	-1.23599	2.802698
FAM179A	5.24E-05	9.016261	2.53594	2.800538
SUGCT	2.75E-03	4.592219	-1.72416	2.800015
FAM133B	4.47E-05	9.244758	2.70175	2.798541
SESN3	1.55E-05	10.92072	3.803099	2.798164
YWHAZ	8.53E-05	8.340638	2.020568	2.797299
SYPL1	1.15E-05	11.42897	4.101699	2.796904
CTBS	8.58E-05	8.331976	2.013705	2.796004
BCKDHB	5.28E-05	9.004857	2.527556	2.7957
ITGB1	6.61E-05	8.687111	2.289688	2.794976
PYROXD1	1.47E-05	11.01222	3.857967	2.794206
YPEL2	2.48E-04	7.0056	0.877406	2.793418
NAIP	6.10E-04	6.019509	-0.09239	2.793365
KCMF1	6.16E-06	12.58893	4.730673	2.793322
KALRN	7.61E-05	8.494137	2.141086	2.79316
ZC3H7A	3.32E-05	9.68942	3.012972	2.79247
ANKHD1	2.32E-05	10.24904	3.384481	2.792297
SREK1	4.15E-06	13.37786	5.121121	2.792197
OSBPL8	1.60E-04	7.533808	1.351156	2.792157
CDC42BPA	9.98E-03	3.545429	-3.11033	2.791972
IGKV1D-8	8.92E-05	8.281031	1.973202	2.789548
ALCAM	2.41E-04	7.040552	0.909685	2.789141
NUDT4	3.13E-05	9.779274	3.074096	2.78842
MAPRE2	1.24E-03	5.315141	-0.86145	2.787842
NFKBIZ	2.12E-05	10.39455	3.477607	2.787188
DENND5B	1.51E-05	10.96053	3.827032	2.787115

RNF216	9.19E-06	11.83658	4.33069	2.786253
SLC36A4	2.34E-05	10.23984	3.378547	2.785444
PAQR3	8.17E-06	12.05506	4.44979	2.784915
MTDH	2.49E-04	7.003906	0.875837	2.784702
WASF2	2.43E-03	4.700802	-1.58964	2.784396
MNS1	4.10E-05	9.373978	2.793722	2.784229
STAP1	4.23E-04	6.40734	0.303112	2.783626
ARHGAP24	3.61E-04	6.58061	0.473776	2.783597
DPH6	4.98E-04	6.233367	0.128048	2.782845
HAUS6	1.16E-04	7.941809	1.697444	2.782014
BLOC1S6	6.90E-05	8.628685	2.245033	2.780676
DENND4A	3.01E-05	9.842355	3.116664	2.780659
H2AFV	2.85E-05	9.92819	3.174135	2.778446
UBXN4	4.73E-05	9.162649	2.64264	2.778361
DZIP1L	2.81E-04	6.864734	0.74592	2.778246
PPP1R9A	5.93E-04	6.04849	-0.06217	2.778157
IPCEF1	1.11E-04	7.988753	1.736246	2.778062
GOT2	3.47E-06	13.74697	5.294421	2.778022
RAB28	1.73E-04	7.434102	1.263989	2.777943
ZFX	1.89E-04	7.328021	1.170114	2.777731
SERPINC1	5.07E-03	4.078213	-2.3849	2.777117
RMND1	1.11E-04	7.998029	1.743889	2.776604
ACADM	7.83E-06	12.13242	4.491371	2.775837
FCAR	4.39E-04	6.366756	0.262609	2.775703
KLHDC1	2.29E-05	10.27161	3.399012	2.775441
EPHA4	1.03E-04	8.094497	1.82289	2.774985
CCDC15	6.14E-05	8.791861	2.369027	2.77287
LOC100130078	1.70E-04	7.458434	1.285355	2.772779
EIF3A	5.36E-05	8.982457	2.511058	2.772612
PAPOLA	1.63E-04	7.506013	1.326959	2.772272
IL1RAP	8.86E-05	8.289014	1.979565	2.770215
ACTG1	9.39E-05	8.212417	1.918283	2.769827
YWHAE	2.45E-03	4.694642	-1.59722	2.769659
PMAIP1	1.88E-04	7.334156	1.175576	2.769511
TET1	2.05E-04	7.232285	1.084374	2.768076
CFAP97	2.78E-04	6.87563	0.75617	2.767771
LOC100506476	4.48E-03	4.180425	-2.25037	2.767454
C1QTNF3	2.21E-03	4.784936	-1.48662	2.766758
EMBP1	4.91E-05	9.108083	2.603066	2.76644
TSTD3	4.55E-04	6.329029	0.224776	2.7662
PBRM1	2.89E-05	9.90322	3.15747	2.765337
LOC100506548	1.59E-03	5.082235	-1.13102	2.765225
CAND1	6.00E-06	12.6412	4.75743	2.765196
SEC23A	1.62E-04	7.514501	1.334357	2.764532
AP4E1	2.28E-05	10.27843	3.403402	2.763573
TTC7A	3.93E-05	9.434148	2.836114	2.763374
KIF13A	1.48E-04	7.627842	1.432434	2.763019
DEPDC1B	2.01E-03	4.871458	-1.38179	2.76299

KIF21A	1.40E-03	5.203958	-0.98916	2.762642
RBM48	5.03E-04	6.222763	0.117256	2.762362
NEK7	1.42E-04	7.679383	1.476605	2.762035
NBPF15	9.35E-05	8.21771	1.922534	2.761809
ECT2	2.98E-04	6.797372	0.682246	2.761724
MON2	4.85E-03	4.115163	-2.33609	2.759903
RANBP6	2.76E-04	6.883421	0.763491	2.759779
MARCH7	8.19E-05	8.395463	2.063852	2.759612
CNOT4	6.93E-05	8.622504	2.240292	2.759226
ACSL3	2.16E-05	10.36358	3.457901	2.757372
UBXN7	1.35E-03	5.235864	-0.95233	2.755977
FRYL	2.10E-05	10.41547	3.49088	2.755782
HIPK2	1.65E-04	7.490554	1.313467	2.75562
EPS15	9.71E-06	11.73764	4.27593	2.754601
TTC3P1	6.49E-05	8.713565	2.309812	2.753725
POLR2D	1.74E-03	4.99907	-1.22918	2.753565
RBM25	1.52E-05	10.95531	3.823897	2.753443
DPY19L4	1.07E-04	8.044196	1.781806	2.752419
BBX	2.61E-05	10.06403	3.264044	2.752232
MED13L	2.22E-04	7.139518	1.000355	2.752107
ZNF117	9.00E-06	11.87499	4.35181	2.751754
COQ2	1.37E-05	11.13158	3.928796	2.751378
KRIT1	1.18E-04	7.911645	1.6724	2.751339
USP47	9.95E-04	5.527719	-0.62217	2.750792
NPEPPS	4.51E-05	9.234203	2.694181	2.750668
ATM	4.10E-03	4.253262	-2.15545	2.750636
DCUN1D1	1.46E-05	11.01791	3.861363	2.749565
UBOX5	5.55E-05	8.934077	2.475286	2.749297
SLC30A7	4.31E-06	13.29856	5.083133	2.749035
CCDC43	3.50E-05	9.609573	2.958166	2.748463
SMARCA5	6.56E-06	12.46861	4.668595	2.748012
FRG1CP	2.32E-04	7.08407	0.949687	2.747948
CAMK2D	1.06E-04	8.050555	1.787013	2.747633
SHQ1	1.69E-05	10.76994	3.711578	2.747436
HIF1A	3.93E-05	9.43468	2.836488	2.746963
APOBEC3G	3.49E-05	9.613863	2.961123	2.746886
SNRK	2.89E-04	6.8325	0.715516	2.746881
ARMC1	1.36E-05	11.14502	3.936724	2.746808
ZNF25	1.98E-05	10.51209	3.551823	2.746218
CCDC144B	4.97E-03	4.095665	-2.36182	2.745483
PCYOX1	6.93E-06	12.36299	4.613516	2.745469
MRPL35	3.32E-04	6.673215	0.563514	2.7452
PMAIP1	2.09E-04	7.205915	1.060585	2.744972
CLASP2	2.24E-04	7.125983	0.988018	2.744868
SNTB2	1.23E-05	11.32113	4.039584	2.743857
ZNF181	6.87E-05	8.634251	2.2493	2.742906
RDH11	2.51E-04	6.993575	0.866268	2.742592
ATAD5	4.44E-05	9.254979	2.709071	2.742441

SIMC1	2.00E-05	10.49438	3.540698	2.742316
PMAIP1	9.15E-05	8.246186	1.945366	2.742315
DSC2	3.09E-03	4.490689	-1.85153	2.741969
MCTP1	3.35E-03	4.423028	-1.93728	2.741875
NAIP	1.79E-03	4.976389	-1.25613	2.741412
HPS3	4.97E-06	13.01001	4.942575	2.740828
SRP72	6.48E-05	8.715165	2.311027	2.74066
TFEC	2.08E-03	4.83804	-1.42215	2.740113
FAM60A	3.26E-05	9.718532	3.032839	2.740088
GHRLOS	3.22E-06	13.90649	5.367563	2.739561
MERTK	2.09E-05	10.41728	3.492028	2.739267
CCDC82	6.10E-06	12.60796	4.74043	2.739155
KIAA0922	9.98E-04	5.52446	-0.62579	2.739126
RSU1	8.11E-05	8.408248	2.073908	2.738955
TYW1	3.94E-05	9.432703	2.8351	2.738481
SDC1	7.01E-04	5.876476	-0.24312	2.738141
RNF13	7.96E-04	5.748729	-0.38005	2.737549
SLC39A6	6.46E-05	8.720588	2.315144	2.737526
GIGYF2	1.01E-04	8.114181	1.838903	2.737179
PMAIP1	9.42E-05	8.207928	1.914675	2.736975
CSGALNACT2	3.23E-05	9.730942	3.04129	2.736711
CCNE2	2.93E-03	4.537037	-1.79319	2.736704
LCLAT1	6.47E-05	8.717114	2.312507	2.736552
ATF2	2.43E-05	10.17958	3.339533	2.735182
CHD1	1.12E-04	7.984974	1.73313	2.735102
RALGAPA2	2.84E-05	9.929053	3.174711	2.734874
ZBTB1	9.82E-06	11.71604	4.263907	2.733932
KLF11	2.75E-05	9.983823	3.211112	2.733843
HPS3	6.79E-06	12.4015	4.63366	2.733327
PNPLA8	3.99E-05	9.414567	2.822349	2.733282
NEK1	3.63E-05	9.553848	2.919643	2.733256
EIF5B	6.77E-06	12.40914	4.637652	2.732956
YBX3	8.26E-04	5.711449	-0.42042	2.73199
CD44	2.03E-05	10.46728	3.523632	2.730931
RPL5	1.16E-03	5.38031	-0.78742	2.730929
UPP2	8.39E-03	3.678248	-2.92582	2.730696
GPR39	1.01E-03	5.509245	-0.64271	2.730244
SLC2A2	1.89E-04	7.328204	1.170277	2.72963
LOC105379426	1.21E-03	5.341812	-0.83108	2.728752
IL7	2.67E-04	6.921919	0.799566	2.728252
HIF1A	4.45E-05	9.25075	2.706043	2.728072
FBXL4	8.58E-05	8.332221	2.013899	2.726666
APC	7.89E-04	5.757654	-0.37041	2.725724
TSPAN18	1.09E-03	5.439066	-0.72118	2.725273
VTI1A	3.57E-05	9.580359	2.937999	2.725233
EIF5B	2.71E-05	10.00322	3.223955	2.725166
PMAIP1	6.27E-05	8.76264	2.346987	2.725147
ZFP90	8.12E-06	12.06639	4.455901	2.724878

ARID5B	4.21E-04	6.4127	0.308446	2.723921
HPS3	3.90E-06	13.50474	5.181338	2.722982
SRGAP2C	1.43E-03	5.179238	-1.01779	2.722743
EIF5B	1.50E-05	10.96787	3.831435	2.721961
CLINT1	1.71E-04	7.44764	1.275885	2.721905
ZNF107	6.43E-05	8.727381	2.320298	2.721809
PKN2	3.59E-05	9.570055	2.93087	2.721594
LOC100287497	5.88E-05	8.85279	2.414755	2.72128
SGPP1	1.38E-04	7.713782	1.505937	2.719682
CLCN3	4.43E-04	6.358808	0.254653	2.719679
PRKDC	2.42E-03	4.703013	-1.58692	2.719265
UGGT2	5.27E-04	6.171572	0.064953	2.719141
IFNAR2	1.74E-03	5.000763	-1.22717	2.718313
ADAMTSL3	4.09E-05	9.376898	2.795786	2.717643
MAP3K2	3.06E-05	9.815885	3.098835	2.717429
ACER3	1.31E-03	5.261491	-0.92285	2.717003
ARL13B	1.30E-04	7.796062	1.575624	2.716631
UEVLD	1.92E-04	7.31303	1.156754	2.716029
C2CD4B	1.04E-04	8.081726	1.812481	2.715158
TBC1D12	1.47E-03	5.155052	-1.0459	2.714719
PLXNC1	1.42E-03	5.188623	-1.00691	2.713391
IDH3A	7.12E-04	5.860497	-0.26013	2.712656
PDE4C	2.05E-05	10.45306	3.514664	2.711075
SYNCRIP	6.23E-05	8.770703	2.353076	2.710811
ACAP2	3.07E-04	6.761405	0.648035	2.709974
RAP2C	2.00E-05	10.49004	3.537969	2.709795
C3orf38	1.38E-04	7.718834	1.510235	2.709523
DIS3	1.63E-04	7.510097	1.330519	2.708949
SRSF7	5.15E-06	12.94244	4.909124	2.708357
ERO1A	2.63E-05	10.04931	3.25436	2.708017
HPS3	4.48E-06	13.22102	5.045721	2.707991
OR7E47P	5.04E-05	9.071425	2.576348	2.707762
PAPD5	4.52E-05	9.229018	2.690459	2.707745
ZNF638	2.83E-05	9.936722	3.17982	2.707533
TXNDC9	7.59E-05	8.498802	2.144717	2.707206
GRM4	8.29E-05	8.379092	2.050955	2.706414
ATXN3	9.57E-05	8.187725	1.898414	2.705521
ATG14	8.86E-03	3.63625	-2.98391	2.703274
DICER1	1.06E-03	5.465513	-0.69153	2.702861
IQGAP1	2.23E-04	7.130516	0.992151	2.701459
CNTLN	5.23E-04	6.180261	0.073855	2.70123
TBL1XR1	5.21E-05	9.024411	2.541925	2.70121
IPO8	1.97E-03	4.886882	-1.36321	2.701072
ZNF720	3.69E-05	9.531574	2.90418	2.700719
SORD	2.20E-04	7.145392	1.005702	2.70027
FN1	8.84E-03	3.638658	-2.98058	2.700125
SMIM15	2.70E-05	10.00986	3.228346	2.700021
IPMK	5.33E-05	8.991579	2.517781	2.699615

ACBD5	9.23E-06	11.8304	4.327286	2.698943
BRWD3	3.77E-05	9.496309	2.879624	2.698654
HPS3	1.06E-05	11.57564	4.185135	2.697822
LPP	6.37E-05	8.739369	2.329384	2.697673
ATAD2	5.03E-05	9.075633	2.57942	2.697169
CCPG1	7.76E-05	8.468203	2.12087	2.697114
SRP9	1.10E-04	8.006526	1.750883	2.696583
CKAP4	1.15E-03	5.383789	-0.78348	2.696337
TMEM192	3.66E-04	6.567229	0.460726	2.696058
KIRREL	1.50E-04	7.613939	1.420473	2.693509
ANKRD28	3.24E-04	6.703442	0.592586	2.69254
APOPT1	9.52E-05	8.194029	1.903492	2.692046
ZNF347	3.86E-05	9.462282	2.855843	2.69075
PDCD7	8.66E-05	8.320572	2.004659	2.690297
HORMAD1	3.47E-04	6.626314	0.518192	2.690188
ESCO1	1.60E-05	10.85941	3.766053	2.689166
NDUFB2	2.33E-05	10.2473	3.38336	2.688791
DENND4A	7.79E-06	12.14253	4.496783	2.688061
SLC4A7	1.00E-04	8.123091	1.846139	2.686707
SDHD	3.29E-05	9.705423	3.023901	2.686692
CCPG1	7.89E-05	8.444686	2.102487	2.686045
DOCK4	2.46E-03	4.688161	-1.60521	2.68584
ATAD2	9.69E-05	8.170457	1.884486	2.685688
TVP23C	3.96E-05	9.423847	2.828876	2.685277
PPP2R5A	7.53E-05	8.509602	2.153114	2.684884
FBLL1	5.59E-04	6.111041	0.002679	2.683471
ERAP1	2.00E-03	4.87405	-1.37866	2.68304
WAC	2.66E-04	6.9254	0.802821	2.681947
CCPG1	2.31E-04	7.091951	0.95691	2.681273
ARHGEF6	3.60E-04	6.585339	0.478384	2.68065
DBT	2.62E-05	10.05757	3.259795	2.680623
FRYL	1.83E-04	7.370872	1.208177	2.680188
SMIM10	1.33E-03	5.250065	-0.93598	2.679319
PPA2	1.65E-05	10.81574	3.739526	2.67917
GPR141	3.59E-04	6.587332	0.480325	2.679075
FIGNL1	4.38E-05	9.274124	2.722763	2.678959
MTRF1L	1.48E-05	10.99471	3.847507	2.678857
HTR1E	6.92E-05	8.624534	2.24185	2.678556
EML4	5.77E-05	8.878599	2.434033	2.677416
TMF1	1.71E-05	10.75193	3.700556	2.677093
ARRDC4	7.95E-04	5.749951	-0.37873	2.677093
MAN2A1	2.79E-04	6.869641	0.750537	2.676653
GK	3.55E-05	9.589381	2.944234	2.67612
NR3C1	7.05E-05	8.59934	2.222495	2.67586
SLC16A7	1.26E-03	5.301334	-0.87721	2.674895
MAP7D3	1.10E-04	8.010658	1.754281	2.673278
SETD2	9.09E-05	8.255827	1.953078	2.672392
CNST	1.73E-04	7.439236	1.268502	2.671824

PTGES3	5.14E-06	12.94322	4.90951	2.671329
PML	4.16E-05	9.35243	2.778474	2.670879
DCN	3.98E-03	4.277313	-2.12428	2.670472
MLLT10	2.68E-04	6.917643	0.795568	2.670313
EIF5B	7.82E-06	12.13704	4.493848	2.669101
MBNL1	3.36E-04	6.662483	0.553167	2.668828
CD2AP	2.14E-04	7.180326	1.03743	2.668163
CSNK1G3	1.13E-04	7.972062	1.722474	2.667162
DNM1L	7.51E-05	8.51281	2.155606	2.666972
ZNF711	6.92E-03	3.829413	-2.71873	2.665917
MGC70870	2.37E-04	7.058473	0.926184	2.665521
USP33	5.78E-05	8.875964	2.432067	2.66499
ATG4C	4.65E-05	9.188752	2.661488	2.664261
LOC105373326///I	4.99E-04	6.229467	0.12408	2.664058
CCDC25	6.35E-05	8.744261	2.333088	2.663747
CAND1	1.52E-05	10.94825	3.819661	2.66275
HIST1H3I	1.43E-05	11.0508	3.880952	2.661495
UMODL1	1.72E-04	7.440247	1.269391	2.659917
CHN2	8.20E-04	5.71883	-0.41241	2.659649
PMAIP1	3.20E-04	6.716287	0.604908	2.659595
NUCKS1	2.14E-05	10.38159	3.469366	2.659459
HAND2	1.36E-04	7.730368	1.520038	2.659257
THUMPD1	8.15E-06	12.05799	4.451371	2.659028
RHOBTB1	7.76E-04	5.774112	-0.35267	2.659002
JADE2	7.21E-05	8.568971	2.199094	2.658999
OXSR1	2.21E-05	10.32699	3.434537	2.658884
SPOCK1	1.21E-03	5.338663	-0.83466	2.658563
SBNO1	2.32E-04	7.087467	0.952802	2.657296
PMAIP1	7.71E-05	8.476332	2.127214	2.657193
CHD2	1.93E-04	7.30185	1.146773	2.657169
SLC35A5	4.36E-04	6.37576	0.271612	2.656212
ARID4A	4.12E-05	9.3661	2.788152	2.656048
ADGRD1	2.81E-04	6.86256	0.743873	2.655618
RPS21	4.23E-06	13.33561	5.100916	2.655267
SOCS6	1.13E-04	7.970156	1.7209	2.655127
TAS2R39	3.41E-03	4.407985	-1.95644	2.653521
RPL14	1.29E-05	11.23622	3.990213	2.653349
RSL1D1	5.26E-05	9.010217	2.531498	2.653044
ZC3H7A	1.12E-04	7.980694	1.7296	2.652896
SPRTN	1.40E-04	7.699025	1.493368	2.652371
ADAR	2.95E-04	6.807749	0.692089	2.651702
SLC37A3	9.00E-04	5.626476	-0.51315	2.651509
SLC25A40	5.44E-05	8.96104	2.495245	2.651468
EPG5	9.99E-04	5.523875	-0.62644	2.650437
SF3B3	3.58E-04	6.59011	0.483029	2.650297
CREBRF	2.17E-05	10.36239	3.457139	2.650143
RUBCN	7.84E-03	3.730993	-2.85321	2.649857
FRAS1	3.33E-04	6.67166	0.562016	2.649232

SNAPC3	4.62E-05	9.197812	2.668018	2.649062
CCPG1	6.94E-05	8.620828	2.239006	2.648898
LMBRD2	2.59E-04	6.958227	0.833436	2.648814
B3GALNT2	1.16E-03	5.383153	-0.7842	2.648537
C5orf24	3.70E-05	9.52457	2.89931	2.647576
CCPG1	5.30E-05	8.999209	2.5234	2.64644
RAP1B	1.05E-04	8.0684	1.801604	2.646304
PACRGL	3.64E-04	6.57161	0.465001	2.645805
KMT2C	8.72E-05	8.311014	1.997068	2.645284
LOC101927588	2.81E-05	9.948358	3.187565	2.644785
GPR26	4.01E-05	9.404371	2.815169	2.644283
FN1	3.24E-03	4.451486	-1.90113	2.644152
RPL23	5.93E-04	6.048234	-0.06244	2.643369
FAM172A	1.41E-03	5.197518	-0.99661	2.643146
ZBTB41	1.21E-04	7.884092	1.649447	2.643014
ARIH1	3.14E-04	6.737885	0.625583	2.643007
PRKCI	1.10E-03	5.433444	-0.7275	2.642784
ARRDC3	1.05E-03	5.477676	-0.67792	2.642302
DHX40	9.08E-05	8.257269	1.954231	2.642023
C3orf38	1.53E-05	10.94262	3.816275	2.641915
GJB2	1.20E-03	5.345226	-0.8272	2.641829
BLOC1S6	5.79E-04	6.074315	-0.03533	2.641611
EIF5B	1.46E-05	11.01849	3.861709	2.641481
CEP135	3.14E-04	6.736396	0.624159	2.640323
AOX1	9.08E-03	3.617362	-3.01011	2.639894
COPE	9.67E-05	8.1738	1.887184	2.639463
STX2	1.33E-04	7.760005	1.545168	2.639446
ATRX	8.12E-06	12.06558	4.455462	2.639397
RBM47	4.21E-04	6.411573	0.307324	2.638949
SLC9A2	6.38E-06	12.52201	4.696234	2.63869
CTAGE5	2.60E-05	10.07049	3.268288	2.638333
CCDC141	2.81E-04	6.863543	0.744798	2.638153
FNDC3B	1.45E-04	7.655559	1.45622	2.637989
LTBP1	2.01E-04	7.255533	1.105285	2.637698
ZNF136	1.15E-03	5.388893	-0.77771	2.637232
ANKRD11	3.19E-04	6.720524	0.608968	2.636865
KRR1	1.58E-05	10.88542	3.781798	2.636446
KRBOX4	2.91E-05	9.895917	3.152587	2.635162
SENP6	1.65E-05	10.81007	3.736072	2.634963
HPS3	1.22E-05	11.3262	4.042519	2.634705
TMEM2	8.35E-04	5.700002	-0.43286	2.634461
ABCD3	3.17E-03	4.470883	-1.87656	2.633867
DDX3Y	8.06E-06	12.07936	4.462886	2.633237
STK38L	4.56E-04	6.326093	0.221824	2.633096
PPP1R15B	7.11E-05	8.588258	2.213964	2.631778
KIAA1143	1.57E-04	7.555494	1.36998	2.631668
CD164	3.51E-04	6.612218	0.50452	2.631429
HMGB3P1	7.92E-05	8.441097	2.099677	2.631104

KIF5B	1.92E-05	10.55909	3.581254	2.630918
PRUNE1	1.08E-04	8.026358	1.767179	2.630648
XRCC5	1.85E-04	7.353392	1.192674	2.630229
GPATCH2L	5.30E-03	4.042145	-2.43274	2.629382
KPNA5	7.85E-04	5.762488	-0.3652	2.629275
ANKRD20A2	6.58E-03	3.869587	-2.66422	2.628502
SDAD1	2.10E-05	10.41228	3.488854	2.628136
NEK1	2.09E-05	10.41847	3.492783	2.627932
TCF15	1.88E-04	7.333377	1.174883	2.627685
KIAA1549L	4.47E-04	6.348452	0.244275	2.627638
ANKIB1	8.22E-05	8.39043	2.05989	2.62735
CCPG1	7.80E-05	8.461093	2.115318	2.627154
KMT2C	1.14E-04	7.955838	1.709062	2.627112
COL6A6	5.12E-04	6.202351	0.09644	2.62709
SLITRK4	5.74E-03	3.978204	-2.51801	2.626881
NBPF19	6.52E-04	5.951314	-0.16392	2.626504
HPS3	5.79E-06	12.71093	4.792918	2.626333
CCNT2	3.77E-04	6.533658	0.427889	2.62633
PAXBP1	1.35E-04	7.741074	1.529126	2.626241
ALDH9A1	5.88E-05	8.852335	2.414414	2.625989
LOC100130193	5.21E-03	4.057151	-2.41281	2.624143
LOC101929550	8.38E-06	12.0065	4.423532	2.623926
CXCL17	1.86E-04	7.348009	1.187893	2.623163
FKTN	6.87E-05	8.635209	2.250034	2.622928
CAPNS2	7.04E-04	5.87302	-0.2468	2.622403
PPME1	3.80E-04	6.52606	0.420438	2.621719
GJB2	9.54E-05	8.191562	1.901505	2.620896
TMEM131	2.87E-03	4.555663	-1.76984	2.620548
PHACTR2	6.61E-05	8.687852	2.290252	2.62035
NR3C1	1.35E-04	7.741614	1.529584	2.619612
KDM4C	5.62E-06	12.77012	4.822866	2.619186
ZBTB43	1.28E-05	11.24681	3.996388	2.61756
NEK9	1.21E-03	5.336144	-0.83752	2.617515
PTGES3	1.49E-04	7.617921	1.423901	2.61745
CBX1	4.94E-04	6.240061	0.134854	2.617437
TYW3	8.16E-05	8.399302	2.066873	2.616943
ARCN1	1.58E-04	7.549835	1.365072	2.616018
PRKAG2	1.63E-04	7.511247	1.331522	2.615994
SCML1	9.61E-05	8.181311	1.893244	2.615663
CCDC144A	1.47E-03	5.152376	-1.04901	2.615357
PGM2L1	1.39E-04	7.706148	1.499438	2.613947
PLK4	1.03E-04	8.095887	1.824022	2.613701
HNRNPA1	7.17E-05	8.575825	2.204382	2.612871
LONRF1	1.77E-05	10.68932	3.662076	2.61184
EIF4E3	6.43E-05	8.727022	2.320026	2.611732
FAM155B	8.45E-05	8.353407	2.030673	2.611518
BLZF1	9.32E-05	8.222986	1.92677	2.611361
CCDC149	4.99E-05	9.086585	2.58741	2.611123

DBF4B	6.74E-03	3.850757	-2.68974	2.610954
GJB7	2.05E-04	7.231988	1.084106	2.610275
NR3C1	1.45E-04	7.655007	1.455748	2.610009
FAM91A1	2.15E-04	7.173051	1.030833	2.60901
LAMTOR3	7.80E-04	5.768223	-0.35901	2.608443
GOLGA7	2.16E-04	7.168267	1.026492	2.607293
SERBP1	3.14E-05	9.775601	3.071609	2.607048
DNAJB14	1.49E-05	10.98881	3.843977	2.606874
ABCB10	1.15E-05	11.42895	4.101685	2.606852
CRABP1	2.07E-05	10.43635	3.504101	2.6067
LIN7C	1.26E-04	7.831865	1.605739	2.606621
ARIH1	1.88E-05	10.58982	3.600423	2.605515
SLC13A1	1.63E-04	7.509483	1.329985	2.604972
RYBP	6.51E-05	8.709203	2.306497	2.604898
PHBP19	1.24E-04	7.851713	1.622381	2.60456
CDC40	3.85E-05	9.468187	2.859976	2.604245
SLC16A7	4.67E-05	9.180581	2.655594	2.603273
PHF20	4.60E-05	9.203101	2.671827	2.602789
IPO9	2.89E-05	9.903994	3.157987	2.602419
RDH10	2.72E-04	6.901697	0.780638	2.602153
PDE4C	5.88E-05	8.851323	2.413658	2.60202
LOC100233156	1.29E-03	5.281478	-0.89993	2.601433
POP1	1.61E-05	10.85149	3.761246	2.601225
CD86	1.43E-04	7.67522	1.473047	2.600813
SLC6A2	1.86E-03	4.93922	-1.30045	2.60029
SH3BP5	9.43E-05	8.206833	1.913795	2.600162
TMEM135	3.92E-05	9.440626	2.840663	2.599029
ADAD1	6.14E-04	6.012037	-0.10019	2.59896
ZBTB38	3.76E-04	6.537563	0.431715	2.598633
NAMPT	2.48E-04	7.006016	0.877791	2.598502
CXorf38	1.72E-05	10.74346	3.695364	2.598399
CACUL1	4.25E-04	6.401752	0.297547	2.598325
SERPINB3	3.93E-03	4.288221	-2.11017	2.597967
KRT38	3.24E-03	4.452006	-1.90047	2.597872
SPAST	4.14E-05	9.357038	2.781738	2.597287
MTPN	1.63E-05	10.82849	3.747281	2.596436
STAM2	4.34E-05	9.288654	2.733137	2.595986
TMEM161B	1.84E-04	7.3637	1.20182	2.595919
CCPG1	5.15E-05	9.041553	2.554497	2.595703
HIPK1	2.43E-04	7.030974	0.900853	2.595683
CENPL	5.76E-04	6.079617	-0.02984	2.59527
GON4L	2.18E-04	7.156762	1.016043	2.595109
CA12	2.20E-04	7.146228	1.006463	2.594759
PJA2	1.07E-04	8.039141	1.777664	2.594574
CEP152	1.86E-04	7.349638	1.18934	2.594572
APLN	6.39E-05	8.735134	2.326176	2.59316
FAM204A	5.70E-04	6.08959	-0.0195	2.593121
ERI1	1.37E-05	11.12367	3.924128	2.5925

GYPE	1.93E-04	7.30097	1.145987	2.591222
RHOQ	1.45E-03	5.169793	-1.02876	2.590695
HGF	3.52E-03	4.382069	-1.98952	2.589922
OSTM1	1.61E-05	10.8505	3.760649	2.589242
RALGAPA1	1.05E-05	11.59381	4.195395	2.588793
CRAMP1	6.44E-05	8.724247	2.31792	2.588782
PPP4R2	1.92E-04	7.312554	1.156328	2.588592
UNC5B	6.08E-03	3.932178	-2.57976	2.588457
SS18L1	1.50E-05	10.96745	3.831182	2.588044
BDP1	1.51E-05	10.96676	3.83077	2.587943
F2R	4.75E-04	6.28323	0.178609	2.587935
EZR	1.83E-04	7.370345	1.207709	2.587445
TULP3	4.57E-03	4.163796	-2.27215	2.587403
NOTCH2NL	9.53E-04	5.569482	-0.5759	2.587361
RASAL2	2.94E-04	6.812306	0.696408	2.586525
HPS3	9.65E-06	11.74878	4.282124	2.586398
SEPT7P2	8.30E-04	5.705889	-0.42646	2.585834
ZNF273	5.48E-05	8.951191	2.487962	2.585209
KCTD9	2.73E-04	6.894846	0.774215	2.585023
ZNF100	3.94E-05	9.433555	2.835698	2.58479
NMD3	6.23E-04	5.997124	-0.1158	2.584645
SKP1	2.63E-05	10.05308	3.256843	2.583982
ZNF226	3.34E-05	9.678995	3.005843	2.583974
TRA2A	1.79E-03	4.974267	-1.25865	2.583591
NAA35	2.45E-05	10.16226	3.328273	2.583366
TPMT	4.13E-05	9.363414	2.786251	2.583365
VAMP3	3.30E-04	6.680468	0.5705	2.583271
CREB1	8.49E-04	5.683537	-0.45077	2.582701
ACER3	1.19E-03	5.353522	-0.81778	2.582627
RHOT1	1.27E-03	5.289693	-0.89053	2.582461
ADCY7	1.89E-04	7.328807	1.170815	2.58235
GCSAML	1.37E-04	7.72964	1.51942	2.582166
SLC35A3	6.38E-05	8.736378	2.327118	2.581939
RP9P	2.99E-04	6.792281	0.677413	2.58187
PPT1	4.32E-03	4.209325	-2.21261	2.581467
ERMN	3.92E-04	6.489477	0.384467	2.581266
NNT	1.89E-04	7.328469	1.170514	2.581174
ZFP1	1.25E-05	11.28941	4.021189	2.580587
TAF5	3.85E-05	9.468292	2.860049	2.580163
CCDC82	3.92E-06	13.49366	5.176109	2.579708
APBA3	1.76E-03	4.989157	-1.24095	2.579465
WDR89	1.83E-04	7.366284	1.204111	2.578991
FNDC3A	6.44E-04	5.963767	-0.15081	2.578523
AP5B1	1.22E-03	5.331897	-0.84236	2.578377
PLEKHM3	1.33E-04	7.760729	1.54578	2.578268
STAT1	3.51E-04	6.611698	0.504015	2.578139
MTMR6	2.28E-04	7.107984	0.971581	2.576906
CCPG1	1.08E-04	8.033595	1.773117	2.576202

LOC101927550	1.25E-04	7.841299	1.613654	2.575605
CEP120	1.00E-04	8.125928	1.848442	2.575566
KRTAP9-2	4.14E-05	9.359867	2.783741	2.575517
RAB12	2.15E-05	10.37137	3.462862	2.575475
MKRN9P	5.05E-05	9.068228	2.574012	2.575436
CAPZA2	7.93E-04	5.751871	-0.37666	2.574818
GPR180	1.03E-04	8.088247	1.817798	2.574438
SLC30A9	5.15E-05	9.039851	2.553249	2.573499
IL1R1	7.10E-05	8.588854	2.214424	2.573218
RIOK3	2.23E-03	4.777555	-1.49562	2.573208
BMI1	5.04E-06	12.98259	4.929027	2.572947
CSNK2A1	1.45E-04	7.654617	1.455413	2.57198
TWSG1	3.35E-05	9.67683	3.004361	2.571675
CLEC2D	5.61E-05	8.918738	2.463905	2.570762
LOC100505915	1.15E-05	11.44034	4.108209	2.570573
ATM	5.12E-03	4.070073	-2.39568	2.570301
UBE2D1	3.07E-03	4.498074	-1.84222	2.56954
C20orf194	8.02E-04	5.74016	-0.38931	2.569488
LARP1B	4.36E-05	9.283128	2.729193	2.569128
RAB11FIP1	9.29E-03	3.600037	-3.03419	2.568442
FAM133B	4.21E-06	13.34503	5.105429	2.568214
ABCA1	5.61E-05	8.918831	2.463975	2.568038
NELFCD	5.36E-05	8.98359	2.511893	2.567796
PPIG	1.43E-04	7.671647	1.469991	2.567469
TPMT	8.46E-03	3.672592	-2.93363	2.567281
KAT2B	5.89E-05	8.848926	2.411864	2.567003
ARID4A	9.78E-05	8.158064	1.874473	2.566302
LINC00937	1.19E-03	5.353156	-0.81819	2.565983
HIPK2	5.71E-05	8.893526	2.445157	2.565795
GPR89B	1.91E-04	7.316716	1.16004	2.565418
OSBPL8	1.69E-04	7.461815	1.288319	2.56474
PDXDC2P	1.14E-03	5.396372	-0.76926	2.563834
FGFR1OP2	5.37E-06	12.85675	4.866402	2.563176
OXR1	1.29E-03	5.281339	-0.90009	2.563038
ZNF197	1.52E-04	7.593946	1.403239	2.562885
LOC100190986	4.51E-04	6.3374	0.233185	2.562691
SOHLH2	5.07E-04	6.214011	0.108337	2.562565
SERPINB9	9.55E-03	3.57854	-3.06411	2.561644
SLFN13	1.09E-03	5.435434	-0.72526	2.561582
FAM86DP	6.07E-04	6.023831	-0.08787	2.560629
EPB41	2.21E-03	4.785271	-1.48622	2.560367
PDE4C	3.23E-04	6.705445	0.594509	2.559383
SLC35E1	1.46E-04	7.645205	1.447344	2.559295
RCC2	1.02E-03	5.503265	-0.64937	2.55908
PDE4D	2.87E-04	6.839957	0.72256	2.557612
PPP6R3	2.37E-03	4.721229	-1.56453	2.557426
CCPG1	7.62E-05	8.492287	2.139646	2.556878
SLAIN2	3.99E-04	6.471457	0.366689	2.556205

AGAP1	1.62E-05	10.84473	3.757144	2.555615
ELMO2	1.15E-05	11.43112	4.102929	2.555604
CDC40	3.15E-05	9.769186	3.067262	2.555363
LRRC47	1.87E-05	10.60198	3.607988	2.555357
RPL10	1.72E-03	5.01302	-1.21264	2.554271
RASSF5	4.58E-04	6.321094	0.216795	2.552969
HHEX	1.26E-04	7.8357	1.608958	2.55295
MED28	1.50E-04	7.60991	1.417003	2.551839
TCF4	1.25E-03	5.304558	-0.87353	2.551711
EMC2	1.54E-05	10.92979	3.808559	2.551653
TMEM156	4.87E-04	6.25639	0.151432	2.55064
PPM1K	8.13E-05	8.405039	2.071385	2.549911
EIF5B	6.78E-06	12.40426	4.635104	2.549299
CDC23	8.43E-04	5.690871	-0.44279	2.549203
PLEKHA2	1.40E-04	7.699692	1.493937	2.548456
SCUBE3	1.62E-03	5.06565	-1.15051	2.548189
UBQLN1	8.37E-06	12.01058	4.425743	2.547525
DNAJC24	9.51E-06	11.77436	4.296318	2.5468
NARS	1.74E-05	10.72284	3.682707	2.546475
LACTB	2.18E-04	7.156831	1.016106	2.546407
SDCCAG8	3.55E-04	6.599043	0.49172	2.546396
ULK4P2	2.61E-03	4.638851	-1.66617	2.545813
CCDC126	1.68E-05	10.78375	3.720021	2.545764
GPATCH11	1.47E-05	11.00123	3.851405	2.545329
ABHD2	2.49E-04	7.004607	0.876486	2.545205
WDR41	7.19E-06	12.29303	4.576739	2.544699
ZNF92	2.71E-04	6.903396	0.78223	2.544678
ATM	7.50E-03	3.76574	-2.80557	2.544606
KLHL24	9.37E-06	11.80248	4.311875	2.543859
GLG1	6.30E-05	8.755801	2.341818	2.543691
KATNBL1	2.43E-05	10.17532	3.336764	2.542415
CSNK2A1	6.78E-05	8.653173	2.263785	2.542098
PSMC6	6.99E-05	8.610647	2.231189	2.541712
ZNF732	1.80E-04	7.38501	1.220693	2.541572
YIPF4	1.31E-04	7.780809	1.562756	2.541545
HAUS3	2.62E-05	10.05478	3.257963	2.540916
CMAS	1.99E-05	10.50122	3.544995	2.540592
ZNF302	2.31E-05	10.25984	3.391436	2.540497
TMEM242	9.76E-04	5.545991	-0.60189	2.54046
TDRD3	6.75E-05	8.659096	2.268312	2.540436
ZBTB20	1.38E-05	11.11714	3.920274	2.540309
PPP4R1	2.44E-04	7.024854	0.895205	2.539104
UBR3	1.04E-04	8.079628	1.81077	2.538326
KLF5	2.25E-03	4.767296	-1.50814	2.537486
GOSR1	8.10E-05	8.410657	2.075801	2.537272
NR3C1	1.10E-04	8.00411	1.748895	2.536972
ATAD2	8.28E-05	8.379593	2.051351	2.536824
ARHGAP24	9.43E-04	5.579689	-0.56463	2.536248

DENND1B	3.38E-05	9.66253	2.994567	2.536181
EID1	2.02E-04	7.24907	1.099478	2.536115
GAPVD1	1.36E-05	11.13904	3.933196	2.535945
GALNT4	2.32E-05	10.25043	3.385373	2.535865
LCOR	7.75E-05	8.470456	2.122629	2.535019
KATNBL1	2.64E-05	10.04284	3.250104	2.534742
GAPVD1	2.06E-04	7.228286	1.080772	2.5343
SMA4	6.85E-04	5.90044	-0.21768	2.534263
FAM21C	2.36E-05	10.2266	3.369993	2.533729
IGF2BP2	2.68E-03	4.614865	-1.69595	2.533416
FAM63B	5.24E-05	9.016392	2.536036	2.532282
ORM2	3.25E-03	4.449918	-1.90312	2.532067
RDX	1.04E-04	8.082862	1.813408	2.531943
UBASH3B	1.65E-03	5.050472	-1.16839	2.531457
DGKH	9.04E-04	5.621922	-0.51815	2.53092
PDE4C	4.57E-05	9.213435	2.679263	2.530758
FAM126B	6.60E-04	5.938528	-0.1774	2.530629
ZAK	6.68E-04	5.926439	-0.19016	2.530483
CCPG1	1.45E-04	7.65735	1.457755	2.530257
MPP5	6.75E-04	5.915711	-0.20151	2.530233
APPBP2	1.94E-05	10.5433	3.571382	2.529996
RIC1	4.68E-04	6.298992	0.194527	2.529878
ATP6V1H	1.63E-04	7.511508	1.331749	2.528765
STK3	2.57E-03	4.652593	-1.64914	2.528338
TMED7	2.36E-04	7.064095	0.931352	2.528198
LAMP2	6.28E-04	5.989373	-0.12392	2.527747
N4BP2	2.29E-05	10.27396	3.400528	2.527064
NELFA	2.45E-05	10.16595	3.330674	2.526801
LNPEP	1.60E-03	5.074291	-1.14035	2.525205
MAP7	1.45E-03	5.170578	-1.02785	2.52501
FN1	8.42E-03	3.675831	-2.92916	2.524979
CCDC144A	2.89E-04	6.831501	0.714572	2.524873
ZNF644	2.99E-05	9.852417	3.123428	2.524419
ZNF207	9.79E-06	11.72171	4.267066	2.524287
SHISA9	3.31E-04	6.67662	0.566794	2.523669
C9orf66	6.25E-04	5.994782	-0.11825	2.523478
NR3C1	9.76E-05	8.160989	1.876837	2.523364
MSN	9.82E-04	5.540094	-0.60843	2.523232
ATAD2	1.17E-05	11.40604	4.088542	2.522928
ZBTB26	1.26E-04	7.828358	1.602795	2.522907
TRIQK	2.57E-04	6.965779	0.840462	2.521922
ATM	5.50E-03	4.012365	-2.47238	2.521848
SMURF2	1.64E-04	7.498549	1.320448	2.521731
PIANP	2.17E-04	7.164617	1.023179	2.521726
ABHD3	8.59E-05	8.331123	2.013028	2.521437
ATAD2	1.33E-04	7.757727	1.543239	2.520642
PER2	4.49E-05	9.239512	2.69799	2.519965
ARID4B	1.38E-05	11.1183	3.920961	2.519473

SHPRH	5.79E-04	6.073138	-0.03656	2.519298
AAK1	6.40E-05	8.732178	2.323935	2.519016
RGPD5	8.46E-04	5.686872	-0.44714	2.518164
PHF2	6.43E-04	5.964337	-0.15021	2.517489
SEPSECS	6.43E-05	8.726906	2.319938	2.516795
LINS1	6.29E-05	8.75644	2.342302	2.5167
DHX33	3.51E-04	6.612239	0.50454	2.516583
FAM217B	1.73E-05	10.72687	3.685184	2.515992
GNS	9.60E-04	5.562504	-0.58361	2.515772
ATAD2	6.48E-05	8.715626	2.311377	2.515534
WDR1	4.48E-06	13.22224	5.046314	2.515284
ZNF320	1.98E-05	10.50534	3.547584	2.514954
FAM173B	1.36E-04	7.730111	1.51982	2.51492
ANKDD1A	7.67E-05	8.483427	2.132745	2.514742
ATAD2	3.76E-05	9.500677	2.88267	2.514614
VEZF1	9.78E-05	8.159143	1.875345	2.514104
ZBTB20	1.45E-04	7.652363	1.453481	2.513655
POGZ	1.16E-05	11.42437	4.099059	2.513537
IRS1	8.76E-06	11.92538	4.379401	2.512591
GABPB1-AS1	1.20E-05	11.36079	4.062502	2.512504
CBX1	6.90E-06	12.37169	4.618077	2.511743
C4orf3	5.09E-05	9.056342	2.565324	2.511403
SLC22A13	1.13E-03	5.407216	-0.75703	2.510593
PSEN1	2.49E-03	4.680604	-1.61453	2.510572
PDE4B	9.67E-05	8.17341	1.88687	2.510226
LRIF1	1.13E-05	11.46607	4.122915	2.509987
SMAD1	1.08E-03	5.446668	-0.71265	2.509247
EREG	3.15E-03	4.474941	-1.87143	2.509184
SPATA13	1.63E-04	7.509128	1.329675	2.508978
CCDC88A	2.44E-04	7.026807	0.897007	2.50719
CAAP1	4.34E-05	9.290297	2.734309	2.507076
TPBG	2.05E-03	4.851392	-1.406	2.506883
ATAD2	7.13E-05	8.584048	2.210721	2.506704
ZNF257	1.49E-05	10.98397	3.841082	2.506137
OXGR1	7.95E-05	8.43489	2.094815	2.505528
NR3C1	1.97E-04	7.277279	1.124792	2.505185
SH3RF1	9.79E-05	8.157542	1.87405	2.504909
ATAD2	6.35E-05	8.742897	2.332055	2.504356
SLC25A25	2.29E-05	10.27048	3.398289	2.504263
CSTA	9.60E-05	8.183438	1.894958	2.504063
RC3H1	7.95E-05	8.435376	2.095196	2.503627
COX7A1	2.06E-04	7.225744	1.07848	2.502988
NUS1	3.44E-04	6.635894	0.527471	2.502838
SEMA3C	2.44E-04	7.026828	0.897027	2.502549
JAK1	3.51E-04	6.611446	0.50377	2.502253
LOC102723694	1.42E-03	5.187709	-1.00797	2.501899
MED17	2.53E-05	10.11066	3.294614	2.501746
TNMD	2.90E-04	6.829366	0.712554	2.50173

CRHR2	5.46E-05	8.957395	2.492551	2.501597
GPX7	8.00E-06	12.09347	4.470474	2.500941
RAP2C	6.64E-04	5.931707	-0.1846	2.500806
PDE4C	9.06E-05	8.25957	1.95607	2.499324
MTF1	4.55E-04	6.329195	0.224942	2.499288
OGFOD3	1.68E-05	10.77838	3.716736	2.49928
CYP26A1	4.67E-04	6.301331	0.196886	2.49921
ZNF121	8.38E-04	5.696448	-0.43672	2.499184
NPPA	4.49E-03	4.178337	-2.2531	2.498826
RAP1A	2.99E-04	6.794141	0.679179	2.498423
RABEP1	1.33E-04	7.762503	1.547282	2.498417
FAM3C	1.82E-04	7.371984	1.209162	2.498168
MAP4K4	1.85E-04	7.353245	1.192543	2.497808
ERLIN1	3.32E-04	6.673672	0.563955	2.4978
RFWD3	6.17E-05	8.785011	2.363867	2.497137
ZNF23	1.65E-05	10.81203	3.737269	2.496852
DENND4A	2.17E-05	10.35664	3.453476	2.496561
OSBPL1A	1.07E-03	5.460859	-0.69674	2.496417
IFT57	6.28E-05	8.758641	2.343965	2.496321
LNPK	4.99E-05	9.085406	2.58655	2.495988
RPS6KA5	1.01E-04	8.116103	1.840465	2.495215
FMC1	9.49E-06	11.77919	4.298994	2.494527
APBB1IP	2.41E-05	10.19121	3.347078	2.493949
CARD16	5.30E-03	4.042683	-2.43202	2.493578
ATM	8.27E-03	3.690049	-2.90954	2.493512
TCF4	8.28E-06	12.02898	4.435705	2.493473
SNAP23	8.42E-05	8.357746	2.034103	2.493462
C6orf62	6.72E-05	8.665321	2.273068	2.492578
ASAP1	8.13E-04	5.726809	-0.40376	2.491952
NRIR///CMPK2	1.84E-05	10.62985	3.625299	2.49185
TP53INP2	7.41E-04	5.820297	-0.30307	2.49177
C7orf50	1.21E-05	11.34345	4.052494	2.491435
DYNC1LI2	4.61E-05	9.200131	2.669688	2.491398
NAA35	1.73E-05	10.72836	3.686099	2.491273
TMEM87B	8.39E-05	8.362314	2.037713	2.491212
KRTAP4-9	6.89E-04	5.894226	-0.22427	2.49012
PDE5A	3.22E-04	6.708372	0.597318	2.489894
AHI1	4.93E-03	4.102211	-2.35317	2.489552
PHTF2	1.58E-05	10.88843	3.783613	2.487612
DPYD	5.36E-03	4.032842	-2.44511	2.487504
YTHDF3	3.25E-04	6.699641	0.588936	2.48709
DYNC2LI1	1.77E-04	7.405601	1.238883	2.486756
MARCH6	1.39E-05	11.09728	3.908528	2.486367
PPHLN1	8.94E-05	8.278306	1.97103	2.486295
FAM135A	1.21E-03	5.336472	-0.83715	2.485551
KLHL42	9.27E-05	8.230052	1.932439	2.485413
LONRF1	3.41E-05	9.650335	2.986203	2.4852
CRLF3	1.34E-04	7.756422	1.542134	2.484886

MFSD4B	6.34E-04	5.980197	-0.13355	2.484786
SOCS5	3.45E-04	6.630843	0.52258	2.484768
LARP4	6.82E-05	8.643947	2.256726	2.484579
ENC1	8.43E-04	5.690641	-0.44304	2.484464
DKFZP434I0714	1.08E-04	8.029154	1.769474	2.483607
ARL5A	1.36E-04	7.738296	1.526769	2.482981
QKI	4.22E-04	6.411264	0.307017	2.482845
RPS6KA3	3.65E-05	9.548071	2.915636	2.482355
CHORDC1	2.01E-05	10.4857	3.535239	2.482194
LINC01314	5.05E-05	9.067932	2.573796	2.482083
KIAA1841	4.01E-04	6.465689	0.360989	2.48161
ANKRD26	1.28E-05	11.24918	3.997774	2.481218
NR3C1	2.10E-04	7.203387	1.058301	2.481157
INSIG1	1.62E-04	7.514412	1.334279	2.480872
DONSON	3.36E-05	9.674105	3.002496	2.480773
ABCA5	7.18E-05	8.57418	2.203113	2.480593
ATPAF1	1.46E-04	7.646008	1.448033	2.4805
ZNF146	1.86E-05	10.60676	3.610963	2.480428
SLC30A6	8.87E-05	8.288642	1.979268	2.480202
TIGD6	7.85E-04	5.762521	-0.36516	2.480115
CAMSAP2	1.87E-03	4.934422	-1.30619	2.479993
USP6	9.38E-03	3.592325	-3.04492	2.479958
SOAT1	2.83E-04	6.856009	0.737702	2.478164
ATM	9.61E-03	3.573649	-3.07093	2.477498
PTPN13	2.83E-05	9.938389	3.18093	2.477044
CAV1	9.11E-03	3.614954	-3.01346	2.476997
KMT2C	6.84E-05	8.640248	2.253894	2.476983
ERRFI1	7.49E-06	12.21643	4.536185	2.476822
CYCS	2.16E-04	7.17081	1.0288	2.476277
FLJ23519///RNH1	4.76E-05	9.153037	2.635686	2.476161
B3GNT2	2.84E-05	9.932695	3.177137	2.475994
CDK9	3.96E-05	9.423633	2.828726	2.475742
UGT8	5.32E-03	4.039362	-2.43644	2.475644
ALKBH8	5.15E-04	6.195789	0.089737	2.475589
PHF6	2.20E-05	10.33841	3.441843	2.475509
PSD3	1.98E-04	7.271762	1.119848	2.475351
PPP4R3B	3.62E-05	9.560996	2.924597	2.47433
RAP2C-AS1	2.02E-04	7.247421	1.097995	2.473825
NR1D2	4.80E-04	6.271412	0.166654	2.473607
NR3C1	1.10E-04	8.00048	1.745908	2.473509
MTERF3	3.68E-05	9.535139	2.906657	2.473406
MYL10	7.94E-04	5.750703	-0.37792	2.472889
U2AF1	9.02E-05	8.26498	1.960394	2.472687
PROS1	2.88E-04	6.836043	0.718863	2.472622
BRWD1	2.17E-05	10.36108	3.456308	2.472511
VBP1	8.59E-05	8.331382	2.013234	2.472317
TP73	8.02E-03	3.714136	-2.87637	2.472057
PTGS2	3.43E-04	6.638895	0.530376	2.471836

ARID4B	1.71E-05	10.75404	3.701849	2.47164
GTF3C2	3.05E-03	4.503951	-1.83481	2.471245
DIP2A	4.27E-04	6.397903	0.293711	2.470802
ATM	6.18E-03	3.918645	-2.59798	2.470508
ATG10	7.05E-05	8.598227	2.221639	2.470077
SPTY2D1	1.19E-05	11.37334	4.069734	2.469998
PTPN9	6.92E-04	5.890263	-0.22848	2.469512
SERPINB10	7.05E-03	3.814278	-2.73932	2.469347
SLC25A24	3.77E-05	9.49883	2.881383	2.469252
FAM69A	1.45E-03	5.170488	-1.02795	2.467907
GPALPP1	1.09E-04	8.017006	1.759499	2.467855
MTMR9	6.19E-05	8.78048	2.360452	2.467685
ATM	8.06E-03	3.709734	-2.88243	2.467351
DNAJC3-AS1	3.00E-04	6.787172	0.67256	2.467252
URB1	9.31E-05	8.223939	1.927535	2.466634
CREBBP	6.00E-06	12.64166	4.757666	2.465654
TMEM98	4.03E-05	9.397893	2.810604	2.464982
SLC6A2	3.41E-03	4.407835	-1.95663	2.464595
ATAD2	2.48E-05	10.1438	3.316255	2.464067
TRAPPC8	3.04E-05	9.823281	3.103822	2.46355
CCDC126	1.46E-04	7.647218	1.44907	2.463423
SPIN4	2.21E-04	7.144631	1.00501	2.463414
CAND1	1.02E-05	11.65039	4.227206	2.462628
ZNF75A	2.48E-04	7.005927	0.877708	2.461982
C12orf65	2.43E-05	10.17727	3.338034	2.460371
ZNF429	2.49E-05	10.13627	3.311345	2.460258
DPY19L3	1.34E-04	7.753978	1.540065	2.459829
GOLIM4	6.90E-04	5.893193	-0.22537	2.459569
CPED1	2.80E-03	4.57741	-1.74264	2.459396
NACAP1	9.30E-05	8.225176	1.928528	2.458723
SLC26A3	8.61E-04	5.669558	-0.46601	2.458411
ITGA4	6.93E-05	8.6232	2.240827	2.458369
LRRC57	2.56E-05	10.09495	3.284331	2.458181
ZNF827	1.11E-03	5.425288	-0.73667	2.457447
CCDC174	2.58E-04	6.961953	0.836904	2.457188
TTC33	2.18E-03	4.79589	-1.47329	2.457055
MTF1	1.13E-04	7.9734	1.723579	2.456936
MRPL1	2.19E-05	10.34152	3.443827	2.456927
TAF5L	6.32E-05	8.749552	2.337093	2.456899
FAM175B	8.07E-05	8.414247	2.078621	2.456786
SLCO5A1	5.32E-05	8.993233	2.518999	2.456738
YBX1	2.98E-05	9.854559	3.124866	2.45644
ENY2	1.04E-03	5.484462	-0.67034	2.456368
SPTLC2	4.64E-04	6.307526	0.203132	2.455927
MTF2	1.92E-04	7.30753	1.151845	2.455447
PROSC	2.28E-03	4.757114	-1.52057	2.455145
MS4A7	1.24E-03	5.312103	-0.86492	2.454897
PDE4C	7.35E-05	8.542239	2.178428	2.453983

PDCD6IP	1.51E-04	7.602915	1.410976	2.453906
FBXW2	4.91E-03	4.104371	-2.35032	2.453497
TTC30A	1.64E-03	5.05333	-1.16502	2.45323
CSDC2	7.14E-05	8.581325	2.208622	2.452891
LNPEP	2.61E-04	6.946954	0.822937	2.452534
LCORL	4.71E-04	6.291047	0.186507	2.452425
DLG1	8.62E-05	8.32578	2.008791	2.452167
CLEC7A	1.23E-03	5.326572	-0.84842	2.45047
BMP2K	5.56E-04	6.116341	0.00815	2.450308
VPS13B	4.85E-04	6.259662	0.15475	2.449324
OPN1SW	4.99E-03	4.091275	-2.36762	2.449172
PTGS2	3.96E-04	6.478753	0.373891	2.449136
PDS5B	6.21E-04	6.000787	-0.11196	2.448875
MMS22L	1.74E-05	10.72674	3.685105	2.448329
PICALM	6.40E-04	5.970493	-0.14374	2.447751
FAM65B	1.34E-04	7.752655	1.538943	2.447558
ARL15	2.44E-03	4.696428	-1.59502	2.447548
TRA2B	1.47E-05	11.00878	3.855911	2.44677
CCPG1	1.08E-04	8.030484	1.770566	2.446709
PGGT1B	8.85E-04	5.642793	-0.49527	2.444354
TBC1D22B	3.27E-05	9.71544	3.030732	2.444245
SRGN	6.45E-05	8.721285	2.315673	2.443704
SACM1L	2.14E-05	10.38364	3.470673	2.443475
SDPR	4.04E-04	6.457299	0.352693	2.443372
PRKCI	1.65E-03	5.049104	-1.17	2.442571
PTGS2	4.47E-04	6.347391	0.243211	2.44126
ANKHD1	3.08E-04	6.757677	0.64448	2.440677
RP2	4.07E-05	9.38444	2.801113	2.440319
RBM7	1.21E-04	7.88517	1.650347	2.440294
SDHD	1.47E-04	7.638795	1.441843	2.440203
ATAD2	9.50E-05	8.197481	1.906271	2.439824
SLC16A4	2.07E-03	4.842633	-1.41659	2.439176
MAPT	4.18E-04	6.41946	0.315168	2.438841
UNK	7.11E-06	12.3155	4.588576	2.43848
LRRFIP1	3.35E-04	6.665615	0.556188	2.4381
MEX3B	6.28E-03	3.906113	-2.61486	2.437075
UMPS	1.22E-04	7.875015	1.64187	2.437056
HS2ST1	6.30E-04	5.986067	-0.12739	2.435961
SRP9	5.29E-05	9.001115	2.524803	2.43507
TMEM189-UBE2V	3.02E-05	9.836098	3.112453	2.434942
SKAP2	9.65E-05	8.176236	1.88915	2.434802
OTUD6B-AS1	1.39E-05	11.10573	3.913528	2.434768
SREK1IP1	1.08E-04	8.032433	1.772164	2.434333
FZD7	4.01E-03	4.272272	-2.1308	2.434254
VAV3	2.42E-03	4.706038	-1.5832	2.434249
ZNF552	1.32E-04	7.768372	1.552247	2.433965
CASP5	2.50E-04	6.997737	0.870125	2.433692
AEBP2	1.54E-04	7.580347	1.391494	2.4332

IKZF1	2.10E-05	10.41106	3.488085	2.432975
PRKAB2	4.99E-05	9.086868	2.587616	2.432707
SEPT10	1.12E-03	5.412124	-0.75149	2.432542
KLF3-AS1	2.02E-03	4.865954	-1.38842	2.432527
MSR1	9.00E-04	5.625739	-0.51396	2.432313
SET	4.30E-03	4.214804	-2.20547	2.432296
COPB1	2.43E-05	10.17423	3.336056	2.431217
SLC37A3	1.91E-04	7.315326	1.158801	2.430441
TRAM1L1	2.92E-04	6.818226	0.702014	2.430343
HTRA3	5.60E-03	3.99775	-2.49188	2.429911
DHFR2	2.94E-04	6.809968	0.694192	2.429572
PIK3R1	1.37E-05	11.12594	3.925469	2.42923
PCDHB18P	3.37E-04	6.659326	0.55012	2.428767
CNTN3	3.44E-05	9.636171	2.976474	2.427681
SLC16A1-AS1	2.55E-05	10.10022	3.287784	2.427444
RAI14	6.93E-03	3.828745	-2.71963	2.426766
OTUD6B-AS1	1.90E-05	10.57526	3.59135	2.426698
SAV1	2.11E-04	7.198687	1.054051	2.426002
CDK11B	6.97E-03	3.823401	-2.7269	2.425344
IMPG2	7.72E-05	8.474986	2.126164	2.425118
ACAA2	4.07E-05	9.384347	2.801047	2.424917
HBS1L	5.23E-04	6.181377	0.074997	2.423835
SCAF11	4.00E-05	9.409958	2.819105	2.423488
SNORD14E	1.35E-03	5.233276	-0.95531	2.423148
MIA3	5.77E-05	8.87832	2.433825	2.422702
CHMP5	1.76E-04	7.416714	1.248683	2.422579
ZNF606	2.18E-03	4.796729	-1.47227	2.422365
NT5E	1.12E-03	5.416333	-0.74675	2.422227
ATP5G3	4.30E-04	6.389717	0.285549	2.421103
WDR26	3.45E-05	9.633466	2.974615	2.420957
TCERG1	4.42E-04	6.359772	0.255619	2.420865
CCNA2	4.48E-04	6.344762	0.240574	2.420765
LOC101927765	5.51E-05	8.943677	2.482399	2.420487
CHML	1.44E-05	11.04576	3.877954	2.41997
REL	4.02E-04	6.463561	0.358886	2.419203
ARNTL2	1.14E-03	5.397125	-0.76841	2.418915
SREK1IP1	1.14E-05	11.44551	4.111165	2.418892
RBBP4	1.27E-03	5.29196	-0.88793	2.418614
LRRC57	1.97E-05	10.51959	3.556533	2.418276
SDAD1P1	2.40E-05	10.19577	3.350038	2.417708
CAMK2D	7.17E-05	8.575556	2.204174	2.417247
MDFIC	8.43E-05	8.355896	2.032641	2.41719
DNPEP	4.27E-04	6.398675	0.294481	2.417116
IKBIP	4.00E-04	6.468412	0.363681	2.416999
ST8SIA4	1.58E-04	7.550685	1.36581	2.416685
HECTD4	1.78E-03	4.977738	-1.25452	2.415245
ZNF451	2.81E-05	9.949612	3.188399	2.414523
SEMA6D	2.11E-04	7.19975	1.055013	2.413623

COX11	7.77E-05	8.466426	2.119483	2.413533
RBBP7	2.88E-05	9.912048	3.163367	2.413263
EIF5B	1.06E-05	11.58067	4.187978	2.413224
BPTF	1.33E-03	5.251453	-0.93439	2.413139
SRSF1	5.16E-04	6.194522	0.088442	2.412813
DNAJC8	5.56E-05	8.931421	2.473317	2.412313
LPAR1	5.30E-03	4.042924	-2.4317	2.412151
USP45	2.83E-05	9.938195	3.180801	2.412149
RXRA	1.39E-03	5.206583	-0.98612	2.411726
PARVA	5.65E-05	8.90891	2.456603	2.411326
RPL26	1.36E-04	7.737145	1.525793	2.411063
G2E3	3.87E-05	9.460406	2.854529	2.409951
FOXA1	5.25E-04	6.177101	0.070619	2.409864
USP14	2.23E-04	7.133143	0.994547	2.40844
CLEC5A	1.07E-04	8.044069	1.781702	2.408387
TOR1AIP1	2.89E-03	4.549507	-1.77755	2.407677
SLA	2.22E-04	7.137564	0.998575	2.407564
PFDN1	6.44E-04	5.964221	-0.15033	2.407264
TRIP12	4.48E-04	6.345022	0.240835	2.406172
HFE	9.97E-03	3.546035	-3.10948	2.405596
DAZAP1	4.23E-03	4.227685	-2.18869	2.404799
FGD5	1.17E-04	7.923036	1.681867	2.40477
DESI2	4.70E-03	4.140146	-2.3032	2.404211
USP25	4.95E-05	9.096907	2.594932	2.403627
NR3C1	2.05E-04	7.22879	1.081226	2.403609
SYNCRIP	1.79E-04	7.395443	1.229915	2.40329
BMPR1A	2.66E-04	6.926829	0.804155	2.403139
HCCS	2.67E-04	6.920616	0.798349	2.4031
CRBN	2.08E-04	7.211515	1.065643	2.402755
POLE	1.74E-04	7.426533	1.25733	2.402236
SLC7A13	8.35E-05	8.369018	2.043007	2.401954
STAG2	3.43E-04	6.638171	0.529675	2.401951
PUM2	1.04E-04	8.078868	1.81015	2.401631
CYCS	9.84E-03	3.555645	-3.09605	2.40156
TNFSF8	6.88E-04	5.895549	-0.22287	2.40144
METTL2B	3.24E-03	4.450538	-1.90233	2.401319
AKAP13	4.00E-04	6.469838	0.365089	2.401289
UBE2D1	3.38E-04	6.654674	0.545628	2.401045
NOL10	4.75E-05	9.157625	2.639006	2.400987
OTUD6B-AS1	1.32E-05	11.18843	3.962242	2.400661
FAM92A1	2.46E-03	4.690097	-1.60282	2.400561
BTBD7	1.80E-04	7.388388	1.22368	2.400055
FBXO3	1.33E-05	11.18005	3.957321	2.399866
CRIM1	1.12E-04	7.987266	1.735021	2.39962
HIST2H3A	5.43E-05	8.963439	2.497019	2.399103
NRCAM	9.41E-04	5.581982	-0.5621	2.398993
SLC35A3	1.83E-04	7.370323	1.20769	2.398925
OCR1	2.32E-04	7.084095	0.949711	2.398706

COLEC12	1.52E-03	5.125794	-1.08001	2.398668
LCE1A	2.20E-05	10.33441	3.439283	2.398645
MAPK8	6.58E-06	12.46402	4.666209	2.398473
SMC2	2.89E-05	9.903666	3.157768	2.397456
MORF4L2	2.82E-03	4.571986	-1.74942	2.397154
EIF5B	6.99E-05	8.610836	2.231334	2.396683
LOC100132363	5.25E-04	6.177023	0.070538	2.396201
TMEM242	2.88E-05	9.908347	3.160895	2.396165
HOXB13	8.06E-04	5.735725	-0.39411	2.395613
TAPT1	1.47E-04	7.638817	1.441861	2.394895
PVR	2.53E-03	4.663597	-1.63553	2.394735
PRRC1	9.08E-05	8.257272	1.954234	2.394627
LNPK	4.56E-04	6.326981	0.222717	2.39417
ALS2	6.17E-04	6.007917	-0.1045	2.393903
FBXL19-AS1	5.28E-03	4.046135	-2.42744	2.393422
KIF3A	2.05E-04	7.230336	1.082619	2.39279
RWDD4	3.65E-04	6.569249	0.462697	2.392538
KIAA0319	3.98E-05	9.418504	2.825119	2.391748
FAM76B	1.75E-04	7.420249	1.251797	2.390943
PRKACB	6.27E-05	8.761414	2.346061	2.390558
OTUD6B-AS1	1.73E-05	10.73166	3.688122	2.390478
GUF1	4.09E-05	9.375777	2.794993	2.39044
RRN3	5.14E-05	9.044346	2.556543	2.390254
NUDT15	4.42E-04	6.359563	0.255409	2.390211
USP40	1.71E-03	5.014832	-1.2105	2.390043
SERINC3	6.69E-04	5.924408	-0.19231	2.389817
DLG1	2.63E-05	10.05013	3.254905	2.389719
TICAM2	2.22E-03	4.781566	-1.49073	2.389431
RNF38	6.75E-05	8.659883	2.268914	2.389018
NEK3	7.80E-03	3.735115	-2.84755	2.387936
HNRNPA3	6.16E-05	8.787029	2.365387	2.387907
KAT6B	1.06E-04	8.049724	1.786333	2.387714
C3orf58	9.52E-05	8.194392	1.903784	2.387419
IL13RA1	6.14E-03	3.92384	-2.59098	2.387227
PLOD2	5.26E-04	6.173992	0.067434	2.385473
LSM11	9.11E-06	11.85309	4.339781	2.385219
MRPS11	2.45E-05	10.16325	3.328918	2.385147
LOC84843	2.87E-04	6.840742	0.723301	2.384755
LINC00622	1.93E-03	4.904705	-1.3418	2.383797
BAG3	7.17E-04	5.853902	-0.26716	2.383726
MS4A1	1.25E-03	5.308773	-0.86872	2.38359
PSME3	3.79E-04	6.52652	0.42089	2.383347
PTGS2	4.93E-04	6.243566	0.138416	2.383245
CD274	9.10E-05	8.253573	1.951276	2.382967
TMEM50B	3.55E-05	9.589101	2.944041	2.382547
VPS50	7.62E-05	8.491918	2.139359	2.382534
ATM	7.85E-03	3.730323	-2.85413	2.381922
UACA	6.52E-04	5.950328	-0.16496	2.381581

CLK1	6.99E-05	8.610089	2.23076	2.381542
DNAH7	1.97E-04	7.280275	1.127476	2.381513
DENND1B	1.25E-04	7.843894	1.61583	2.38139
GUCA2B	5.44E-05	8.962898	2.496619	2.380858
PTGS2	4.30E-04	6.388853	0.284687	2.380456
SLFN13	2.05E-03	4.852259	-1.40495	2.380402
MYCBP2	1.28E-03	5.287118	-0.89347	2.380286
RPSA	2.43E-05	10.17858	3.338885	2.379911
MRPL3	2.81E-04	6.861695	0.743058	2.379621
CXCL2	6.25E-04	5.994215	-0.11885	2.379487
PLEKHA5	1.95E-05	10.53216	3.564412	2.379311
SLC35E2	5.36E-03	4.03324	-2.44458	2.379173
PRKAR1A	4.00E-04	6.469704	0.364957	2.379059
RHOQ	3.37E-03	4.418103	-1.94355	2.378711
RAD23A	8.35E-04	5.700449	-0.43237	2.377546
SLC24A1	4.14E-03	4.244875	-2.16634	2.376309
ST13	9.00E-05	8.268297	1.963043	2.375724
MAP4K5	1.74E-04	7.429598	1.260027	2.375593
RP9	2.55E-05	10.10079	3.288156	2.37494
SCRN3	1.91E-03	4.916548	-1.32759	2.374727
LNPEP	5.81E-05	8.867509	2.425756	2.374647
RGPD6	2.24E-03	4.772966	-1.50122	2.374267
TNFAIP3	1.95E-05	10.53172	3.564131	2.374249
SP100	9.92E-06	11.6991	4.254459	2.37392
MFSD14A	4.89E-05	9.115637	2.608558	2.373641
CCNT1	8.51E-05	8.343642	2.022946	2.373636
CEP126	6.57E-04	5.943292	-0.17237	2.373588
GPAT2	2.22E-03	4.778979	-1.49388	2.373518
CLOCK	2.63E-03	4.632309	-1.67429	2.373513
SMG1P5	2.31E-05	10.258	3.390256	2.373437
RMND1	2.82E-04	6.858728	0.740263	2.373308
NLRC5	4.72E-05	9.166271	2.645258	2.372823
HRASLS5	1.87E-05	10.60393	3.609202	2.372793
CCDC54	8.47E-04	5.685722	-0.44839	2.372107
KIF27	5.45E-05	8.960655	2.494961	2.372045
PTGS2	3.84E-04	6.513863	0.408463	2.371483
PPIL4	1.82E-05	10.6453	3.634874	2.37128
KIAA1804	4.15E-03	4.242865	-2.16895	2.37097
MEX3B	7.96E-04	5.747757	-0.3811	2.370619
ERCC2	8.11E-05	8.408185	2.073858	2.370506
KAT6B	2.35E-04	7.070465	0.937204	2.370243
IFT80	1.17E-03	5.373332	-0.79532	2.369907
ERO1B	2.38E-04	7.053777	0.921864	2.369661
FOXN2	6.71E-05	8.66812	2.275205	2.369467
LMBR1	4.10E-05	9.373306	2.793247	2.369219
C18orf54	5.14E-05	9.044209	2.556442	2.36914
HNRNPA3	6.09E-05	8.802412	2.376968	2.368854
SMPDL3A	1.07E-04	8.037535	1.776348	2.368593

C12orf65	1.47E-05	11.00668	3.854661	2.368561
GLUD2	5.41E-04	6.145325	0.038008	2.368385
PDE4C	1.59E-04	7.537155	1.354065	2.368383
KCNK3	2.21E-04	7.140594	1.001334	2.36745
PLAT	4.51E-05	9.23355	2.693712	2.367427
SBDS	2.79E-05	9.959853	3.195206	2.365978
TERF1	1.94E-04	7.298839	1.144083	2.365978
SYT5	2.02E-05	10.47729	3.529941	2.365825
CRCP	5.88E-03	3.959522	-2.54304	2.365735
C1orf226	6.41E-05	8.73022	2.322451	2.365669
LOC100132006	8.04E-04	5.738578	-0.39102	2.365581
HNRNPU	3.94E-04	6.484941	0.379995	2.365459
GATAD1	7.62E-04	5.791811	-0.33363	2.365
PLCB1	6.43E-04	5.965901	-0.14857	2.364907
C8orf37	1.68E-03	5.032237	-1.18991	2.364824
PTGS2	3.59E-04	6.586741	0.47975	2.364671
BMT2	8.94E-06	11.88771	4.358786	2.364629
PDE4C	4.07E-05	9.38468	2.801282	2.364391
SETMAR	1.36E-04	7.737246	1.525878	2.364386
FAM91A1	1.18E-05	11.39501	4.082206	2.364238
YY1	8.58E-04	5.673613	-0.46159	2.36371
TVP23B	1.30E-05	11.21617	3.978493	2.363246
ZNF223	4.25E-04	6.403119	0.298908	2.362933
ZNF562	1.43E-04	7.673149	1.471276	2.361768
PYGO1	8.29E-05	8.379392	2.051192	2.361192
PTGS2	3.83E-04	6.515256	0.409832	2.361154
MEIS3P1	2.11E-03	4.826663	-1.43592	2.360835
PPM1B	7.44E-05	8.524646	2.164793	2.36054
EIF5	8.07E-05	8.414281	2.078647	2.360156
PPP1CB	4.40E-04	6.36524	0.261092	2.359908
TRAK2	7.20E-05	8.569198	2.199268	2.358899
WDFY4	2.06E-04	7.223549	1.076502	2.35877
DDX6	5.36E-04	6.154854	0.0478	2.358634
ZNF652	3.81E-03	4.314685	-2.07601	2.358492
RAB27A	1.13E-04	7.965706	1.717223	2.357737
NPAT	1.64E-05	10.81656	3.740022	2.357615
RND3	4.54E-03	4.169801	-2.26428	2.356921
RAB10	1.69E-04	7.462207	1.288663	2.356874
MPZL1	6.17E-04	6.008497	-0.10389	2.356512
AMD1	6.53E-05	8.705791	2.303904	2.356467
ATF7	6.90E-05	8.628197	2.244659	2.356372
PPP1CC	2.60E-04	6.950682	0.82641	2.356257
ACSL4	2.32E-04	7.084444	0.950031	2.355893
MSANTD2	6.10E-04	6.020029	-0.09184	2.355725
ZNF267	6.81E-04	5.906234	-0.21154	2.35449
TAF3	6.96E-05	8.615932	2.235247	2.354366
FGD6	1.64E-03	5.051898	-1.16671	2.353394
UACA	5.33E-03	4.038608	-2.43744	2.352989

OTUD6B-AS1	1.93E-05	10.54731	3.573893	2.352836
ZNF254	7.90E-05	8.443888	2.101862	2.352663
EIF2S1	2.06E-04	7.225848	1.078574	2.352585
HNRNPA0	2.07E-03	4.844191	-1.41471	2.352555
SH2D4B	1.44E-03	5.174107	-1.02375	2.352272
TFDP1	7.14E-04	5.858394	-0.26237	2.352096
NPEPPS	8.07E-04	5.734355	-0.39559	2.352074
FAM179B	2.22E-03	4.781896	-1.49033	2.351423
SPAST	3.87E-04	6.505086	0.399834	2.350958
ADRA1B	3.20E-04	6.715817	0.604458	2.350469
PCMTD2	2.60E-04	6.952013	0.82765	2.350185
MARCH1	3.38E-04	6.655341	0.546273	2.349331
WDFY1	2.86E-04	6.845091	0.727406	2.349167
ZNF12	1.31E-04	7.777329	1.559816	2.348789
CTDSPL2	2.51E-05	10.1283	3.30614	2.348786
PEX5L	1.21E-03	5.34084	-0.83218	2.348745
HRG	1.17E-03	5.371149	-0.79779	2.348531
NABP1	1.96E-04	7.283062	1.129971	2.348257
ARHGAP29	6.18E-03	3.918577	-2.59807	2.348089
PRL	3.42E-03	4.405731	-1.95931	2.347616
GBA3	3.86E-03	4.304679	-2.08891	2.346985
UBR5	6.97E-05	8.614785	2.234367	2.346731
RORA	7.68E-03	3.74758	-2.83045	2.345983
VASH1	2.26E-03	4.762772	-1.51366	2.345898
BTBD7	1.75E-05	10.70957	3.67455	2.34542
TOR1AIP1	2.32E-05	10.25012	3.385173	2.345269
CD47	5.86E-03	3.962051	-2.53965	2.345222
VMA21	8.28E-05	8.379793	2.051508	2.345096
NRL	1.95E-03	4.895595	-1.35274	2.345055
CLCN3	1.95E-03	4.895432	-1.35293	2.345004
C1orf27	5.15E-03	4.066121	-2.40091	2.344789
ANKRD17	3.16E-05	9.76435	3.063984	2.344282
ZNF587	4.63E-05	9.194681	2.665762	2.343961
SUMO1	5.56E-05	8.931157	2.473121	2.343793
TRMT10B	2.53E-05	10.11386	3.296706	2.343581
MAP2K5	1.58E-03	5.090572	-1.12123	2.343294
TAS2R9	1.74E-03	4.999242	-1.22898	2.343269
THUMPD1	2.03E-04	7.241925	1.093052	2.342957
SCP2	4.93E-05	9.104142	2.600198	2.342933
WASL	1.24E-04	7.847695	1.619015	2.342514
AP5B1	2.81E-04	6.86431	0.745521	2.342219
QSER1	3.97E-03	4.280385	-2.1203	2.341868
NUP50	2.67E-04	6.923629	0.801165	2.341776
MALT1	7.23E-05	8.56397	2.195232	2.341431
AREL1	8.84E-05	8.292718	1.982515	2.341404
MPLKIP	1.16E-04	7.94206	1.697652	2.341257
PGM3	3.44E-03	4.401381	-1.96486	2.341137
NR3C1	2.33E-04	7.077812	0.943948	2.340258

RAB5B	4.00E-04	6.469443	0.364699	2.340041
SEL1L	4.26E-04	6.39995	0.295751	2.339848
RAB22A	1.24E-04	7.85081	1.621625	2.339805
LYRM7	7.09E-05	8.590829	2.215945	2.339705
FEM1C	3.07E-04	6.761196	0.647836	2.338987
OR5P1P	6.78E-04	5.91039	-0.20714	2.338976
TTC33	5.57E-05	8.928261	2.470973	2.338326
C16orf87	4.48E-04	6.346812	0.24263	2.338246
RBL1	1.37E-03	5.221289	-0.96914	2.337712
CCT8	1.50E-05	10.9756	3.836065	2.337033
RBM23	1.66E-04	7.490262	1.313212	2.336737
CCNG1	1.07E-03	5.45322	-0.7053	2.336649
ARL5A	4.26E-04	6.39966	0.295462	2.335915
ABI1	3.66E-05	9.541793	2.911279	2.335618
FRS2	9.64E-05	8.17766	1.890299	2.335205
ZNF136	9.41E-05	8.209172	1.915675	2.335065
ELN	6.66E-05	8.677615	2.28245	2.335027
GOLT1B	1.79E-05	10.67118	3.650879	2.334769
RAMP2-AS1	7.82E-03	3.733643	-2.84957	2.33466
TDRD12	6.16E-03	3.921122	-2.59464	2.333612
SLC38A2	3.10E-05	9.79516	3.084843	2.33318
PVR	5.71E-04	6.088189	-0.02095	2.332671
ZBED1	1.54E-04	7.577452	1.388991	2.332385
RAB9B	1.13E-05	11.46184	4.1205	2.331665
C17orf80	5.07E-04	6.213904	0.108227	2.331491
RHO	4.24E-04	6.406124	0.3019	2.331223
ARIH2	1.55E-04	7.569526	1.382135	2.331081
ZBTB41	2.05E-05	10.44975	3.512571	2.330682
FGD6	9.97E-04	5.526081	-0.62399	2.32997
C22orf29///GNB1I	1.92E-03	4.911742	-1.33335	2.329928
UGCG	3.40E-03	4.411205	-1.95233	2.329505
NAPEPLD	2.75E-05	9.982559	3.210274	2.329287
RND3	2.09E-04	7.20849	1.062912	2.329104
PTGS2	5.99E-04	6.037572	-0.07354	2.328759
CAV1	1.04E-03	5.481218	-0.67397	2.328756
KIAA1614-AS1	7.80E-05	8.460276	2.114679	2.328734
OTUD6B-AS1	2.92E-05	9.886933	3.146576	2.328478
CXCL10	8.44E-03	3.673841	-2.93191	2.3283
AMN1	2.09E-04	7.206465	1.061082	2.328192
SPOP	1.10E-04	8.000073	1.745572	2.328049
ESR1	2.11E-03	4.824992	-1.43795	2.327937
TNFSF15	6.18E-03	3.919657	-2.59661	2.32761
CXCL12	7.28E-03	3.789393	-2.77325	2.32726
MAGOHB	1.41E-04	7.690258	1.485891	2.327195
CDC14C	3.13E-04	6.742489	0.629983	2.327065
UGDH	2.59E-04	6.959124	0.834271	2.327005
NACA2	4.34E-03	4.20674	-2.21598	2.326707
SRSF10	5.85E-05	8.859256	2.41959	2.326521

HIF1A-AS2///HIF1	2.33E-04	7.082004	0.947793	2.325762
FAM60A	1.30E-05	11.221	3.981317	2.32571
MNT	4.41E-05	9.266591	2.717379	2.325648
GORASP2	2.43E-04	7.029579	0.899566	2.325623
PTGS2	5.30E-04	6.166431	0.059682	2.325575
LINC01399	6.16E-04	6.008564	-0.10383	2.325434
NAPB	7.47E-04	5.812004	-0.31195	2.325269
CCDC112	4.28E-05	9.309628	2.748081	2.324962
SLC16A1-AS1	1.09E-03	5.440581	-0.71948	2.324534
MSR1	5.18E-03	4.060705	-2.4081	2.324214
NUSAP1	3.71E-04	6.550352	0.444234	2.32361
STAG1	7.30E-05	8.550308	2.184672	2.323275
ATF1	2.96E-04	6.80561	0.690061	2.323103
ZNF273	2.60E-05	10.07072	3.268439	2.322392
RNASEH1	3.51E-05	9.607626	2.956825	2.321056
CSNK2A1	2.05E-05	10.45103	3.513382	2.320956
CAPN8	5.13E-03	4.068386	-2.39791	2.320834
HPF1	2.42E-03	4.703473	-1.58635	2.32067
SLK	4.08E-05	9.378741	2.797088	2.320606
KIF20B	3.76E-04	6.53556	0.429752	2.320562
TCF12	4.55E-05	9.218498	2.682903	2.320479
CRYBG3	8.79E-04	5.649794	-0.48761	2.319309
MDM2	7.34E-04	5.830438	-0.29222	2.319118
SPRED1	5.85E-03	3.963417	-2.53782	2.318975
PSIP1	2.75E-04	6.887654	0.767466	2.318447
LOC100129406	1.33E-03	5.249726	-0.93637	2.318215
LNPK	4.90E-05	9.112444	2.606238	2.3179
PRKAR1A	1.31E-04	7.784744	1.566077	2.317357
EID1	1.59E-04	7.53892	1.355598	2.317302
KRT19	7.34E-05	8.543808	2.179643	2.317125
MDM2	7.59E-04	5.795541	-0.32962	2.316926
RPL3L	1.03E-04	8.090621	1.819732	2.316543
AKT3	3.30E-03	4.436633	-1.91998	2.316441
INHBB	8.29E-04	5.70725	-0.42498	2.316402
ZNF17	5.47E-04	6.132947	0.02527	2.315951
COMMD5	1.02E-03	5.503424	-0.64919	2.315651
HIF1A-AS2///HIF1	2.46E-04	7.015465	0.88653	2.315389
IGSF6	2.39E-03	4.714292	-1.57305	2.315389
BRD4	1.59E-03	5.08211	-1.13116	2.314811
UBLCP1	4.81E-04	6.269629	0.164848	2.314695
PROSER1	7.68E-04	5.783927	-0.3421	2.314296
DPYD	1.15E-04	7.944367	1.699563	2.313845
SRSF11	3.19E-05	9.752714	3.056088	2.313819
OTUD6B-AS1	2.13E-05	10.39031	3.474913	2.313798
PMS2	9.56E-05	8.188892	1.899354	2.313794
LHCGR	1.63E-04	7.509835	1.330291	2.313692
PARN	5.63E-05	8.91259	2.459338	2.313453
RTN3	1.49E-03	5.140214	-1.06318	2.313149

IL1R1	4.52E-04	6.336654	0.232436	2.312741
ZYG11B	5.68E-04	6.09292	-0.01606	2.312267
PCDH1	7.50E-05	8.514982	2.157293	2.311448
SUMO1	9.22E-05	8.23608	1.937271	2.311256
ACVR2A	1.91E-03	4.914453	-1.3301	2.310998
PDE4C	5.20E-05	9.025759	2.542915	2.31086
C20orf96	3.24E-04	6.703567	0.592706	2.310607
KMT5A	9.51E-05	8.195589	1.904748	2.310378
THOC1	2.94E-05	9.877843	3.140488	2.309963
CRNKL1	1.16E-04	7.935447	1.692169	2.309951
TNFAIP6	8.60E-04	5.670918	-0.46453	2.30986
RAB9B	7.86E-04	5.760699	-0.36713	2.30979
HEG1	6.41E-04	5.967794	-0.14658	2.309315
OR10P1	7.75E-03	3.74075	-2.83981	2.309307
CLK4	1.58E-04	7.546481	1.362163	2.308924
FCAR	6.18E-03	3.919216	-2.59721	2.308658
PIP4K2A	1.13E-03	5.405221	-0.75928	2.308367
NR2C1	1.76E-04	7.412391	1.244872	2.308216
LOC100132790	1.17E-03	5.373547	-0.79507	2.307932
RSBN1	2.99E-05	9.850981	3.122463	2.307696
LOC100130476	7.26E-05	8.559013	2.191402	2.307226
AASDHPPT	1.21E-04	7.885045	1.650242	2.30708
CHUK	5.35E-04	6.156736	0.049733	2.307041
VPS50	1.07E-05	11.55519	4.173577	2.305902
ZNF121	6.85E-04	5.900439	-0.21768	2.305677
HBP1	3.24E-04	6.702633	0.591809	2.305371
DPY19L1	4.25E-04	6.402759	0.298549	2.305305
ADGRL4	5.75E-04	6.081735	-0.02764	2.304524
HSP90AB1	1.63E-04	7.506469	1.327357	2.303332
NUS1	4.06E-04	6.451287	0.346743	2.302721
HIF1A-AS2///HIF1	3.14E-04	6.736613	0.624367	2.302121
UHRF1	9.07E-04	5.61844	-0.52197	2.3018
SLC30A5	2.76E-04	6.881941	0.762101	2.301477
SFPQ	4.39E-05	9.273062	2.722005	2.301235
SRP19	2.56E-05	10.09196	3.282375	2.301191
ST13	1.22E-04	7.872261	1.63957	2.301148
TMEM37	1.75E-03	4.993515	-1.23577	2.300794
SESN1	1.44E-04	7.66019	1.460187	2.300568
OTUD6B-AS1	1.81E-05	10.65211	3.63909	2.300457
YEATS2	4.19E-03	4.236119	-2.17772	2.300279
FAM13A	8.50E-04	5.682335	-0.45208	2.300153
SUMO1	1.07E-04	8.040951	1.779147	2.299323
KIF21A	4.82E-04	6.266847	0.162031	2.299007
B3GNT4	2.90E-04	6.828376	0.711618	2.298942
LOC105379499	1.65E-03	5.049329	-1.16974	2.298924
ZNF430	2.80E-04	6.868725	0.749675	2.29794
DCUN1D4	1.25E-05	11.28545	4.018886	2.296714
PICALM	2.26E-03	4.766034	-1.50968	2.296347

SUMO2	2.39E-05	10.20044	3.353064	2.296288
APIP	4.73E-05	9.163303	2.643112	2.295992
BCAP29	6.90E-04	5.892759	-0.22583	2.295874
HSP90AB3P	1.04E-04	8.077422	1.808971	2.295698
WHAMM	1.70E-05	10.75983	3.705395	2.294574
PGC	5.23E-05	9.018596	2.537655	2.294469
AK4	2.65E-03	4.623499	-1.68522	2.294433
TTC3	8.59E-05	8.330744	2.012728	2.294366
CLIP4	7.73E-05	8.472706	2.124385	2.294198
SLC35B3	4.24E-04	6.404298	0.300082	2.293792
CXCL12	3.13E-03	4.480188	-1.8648	2.293399
C4orf19	2.15E-05	10.37511	3.465243	2.293032
LOC100506990	3.65E-04	6.567786	0.46127	2.291736
C5orf24	5.20E-04	6.185592	0.079311	2.291354
RFK	1.68E-04	7.46923	1.294816	2.291254
DIMT1	4.47E-04	6.348073	0.243895	2.29011
WAPL	1.16E-04	7.940435	1.696305	2.290077
PIK3CG	1.21E-04	7.881396	1.647198	2.28956
SCIN	1.60E-05	10.85936	3.766022	2.289545
THAP10	8.80E-03	3.641461	-2.97669	2.289503
FBLIM1	3.52E-03	4.381561	-1.99017	2.289422
ARF4	4.10E-05	9.37435	2.793985	2.289394
ZFAND5	2.28E-04	7.107277	0.970935	2.288881
PPP6R3	4.40E-04	6.364081	0.259932	2.288603
NFXL1	1.93E-04	7.303255	1.148028	2.288497
TMEM35A	1.52E-04	7.591723	1.40132	2.288292
CPA4	5.44E-03	4.021184	-2.46063	2.287919
MARCH1	9.06E-04	5.619936	-0.52033	2.287434
TCF12	2.61E-04	6.946967	0.822948	2.287385
HELB	2.32E-03	4.741693	-1.53944	2.28709
JAG1	2.77E-03	4.586293	-1.73155	2.286111
SEPT14	8.88E-05	8.285903	1.977086	2.286093
SMAD2	1.82E-04	7.371361	1.20861	2.285668
RRAGB	1.47E-04	7.634369	1.438042	2.285468
NEMF	1.92E-05	10.55735	3.580171	2.284973
MCL1	6.49E-04	5.955987	-0.159	2.284913
WNK1	1.31E-04	7.78031	1.562335	2.283678
LPCAT2	4.12E-03	4.248561	-2.16155	2.283511
CDV3	5.91E-05	8.844752	2.40874	2.282497
ZMYM5	2.18E-04	7.161338	1.020201	2.28189
TNKS	2.43E-04	7.029226	0.89924	2.281655
MCTP1	3.78E-03	4.321046	-2.06782	2.281476
SYK	8.41E-04	5.693891	-0.4395	2.281335
PGAP1	3.77E-05	9.499701	2.88199	2.280989
CSNK2A1	1.33E-04	7.75937	1.54463	2.279865
PIK3CA	3.63E-04	6.576816	0.470079	2.279564
EDARADD	8.09E-04	5.732155	-0.39797	2.279458
RIC8B	2.13E-04	7.186981	1.043459	2.27938

CREBBP	1.90E-04	7.319591	1.162604	2.279371
NREP	1.14E-03	5.395533	-0.77021	2.278917
ZFR	1.61E-04	7.525589	1.34401	2.278775
PCBD2	4.10E-05	9.373968	2.793715	2.27835
TTC3	6.56E-05	8.698592	2.298429	2.277676
MAB21L2	9.59E-04	5.563899	-0.58207	2.277193
L3HYPDH	1.83E-03	4.952082	-1.28509	2.277184
AMOTL2	5.79E-03	3.971955	-2.52638	2.277083
UTP14A	6.82E-04	5.904339	-0.21355	2.276976
SMPDL3A	6.83E-03	3.839887	-2.70449	2.276967
VHL	1.32E-04	7.773516	1.556595	2.276829
YME1L1	1.33E-05	11.17576	3.954806	2.276112
RTN3	1.00E-03	5.522756	-0.62768	2.275377
PPP2R5E	4.39E-05	9.271336	2.720772	2.275369
IER3IP1	6.96E-05	8.616388	2.235598	2.275257
TMEM38B	3.50E-04	6.61663	0.508802	2.274176
VPS26A	5.92E-05	8.843182	2.407564	2.273954
EIF5B	6.08E-05	8.804789	2.378755	2.27385
GPR82	3.58E-03	4.366575	-2.00935	2.273814
TTC30A	1.24E-03	5.312808	-0.86411	2.273777
KIAA1109	2.00E-05	10.4914	3.538827	2.273567
SMIM11A	5.78E-05	8.875998	2.432092	2.272853
MFHAS1	2.72E-03	4.602318	-1.71157	2.272503
MORF4L2	1.33E-03	5.249735	-0.93636	2.272178
STK4	9.33E-05	8.221047	1.925214	2.271867
MCM9	9.84E-05	8.150691	1.868509	2.271459
NPEPPS	1.55E-04	7.574372	1.386327	2.270863
PREPL	6.45E-05	8.723162	2.317098	2.270479
SP100	5.21E-05	9.022699	2.540668	2.270257
ABCB10	5.42E-05	8.96677	2.49948	2.270215
SRRM1	2.43E-05	10.17952	3.339491	2.270054
SLC46A3	1.72E-04	7.44532	1.273847	2.269869
LCE3D	3.47E-03	4.392154	-1.97664	2.269351
RAP2A	1.33E-04	7.759641	1.54486	2.268793
P2RY12	3.42E-03	4.405952	-1.95903	2.268512
ZNF431	2.61E-04	6.949379	0.825196	2.267855
ATG4C	1.02E-04	8.097917	1.825674	2.267813
HOXD9	2.20E-03	4.788436	-1.48236	2.267805
MCTP2	1.56E-03	5.10183	-1.10804	2.267425
USP6NL	1.67E-03	5.035616	-1.18592	2.267393
MXRA5	2.72E-04	6.899374	0.778461	2.266847
PAXBP1-AS1	3.93E-03	4.287957	-2.11051	2.266068
CACNG8	2.04E-03	4.85408	-1.40275	2.265923
KCNAB1	5.94E-03	3.951223	-2.55417	2.265769
KMT5B	4.99E-05	9.084995	2.58625	2.265744
NTRK3	2.50E-04	6.996011	0.868525	2.26501
HNRNPH1	7.01E-04	5.876061	-0.24357	2.263992
METTL4	2.76E-04	6.882318	0.762455	2.263822

PNISR	1.95E-05	10.53217	3.564413	2.263248
PLAGL1	6.17E-04	6.007148	-0.10531	2.26278
CAMK2N1	8.47E-05	8.349394	2.027499	2.262601
ADAMTSL4	8.81E-03	3.640626	-2.97785	2.262381
UFL1	2.18E-05	10.35292	3.451103	2.26236
NMD3	4.09E-04	6.44357	0.339098	2.261589
GPR1-AS	9.28E-04	5.59567	-0.54701	2.261514
SUMO1	7.67E-05	8.483621	2.132896	2.261289
MINPP1	8.26E-04	5.71145	-0.42042	2.261124
CCDC69	3.03E-04	6.778893	0.664689	2.260756
ANKRD36B	1.32E-04	7.773283	1.556398	2.260638
SLC25A5	7.78E-04	5.771439	-0.35555	2.260589
SERBP1	2.02E-04	7.250521	1.100782	2.260499
GNPTAB	2.64E-04	6.935653	0.812395	2.260122
FAM65C	4.56E-03	4.165184	-2.27033	2.259914
NCKAP5	1.87E-05	10.60005	3.606791	2.259307
ADAM10	5.76E-04	6.07921	-0.03026	2.25887
RGS12	1.57E-03	5.096584	-1.11418	2.258835
TSHZ2	1.09E-03	5.438174	-0.72219	2.258181
PDK4	2.39E-03	4.713684	-1.5738	2.257635
TBC1D32	2.59E-04	6.958104	0.833322	2.257352
ABHD13	6.10E-05	8.800055	2.375195	2.257205
MICU3	4.65E-03	4.149184	-2.29132	2.257047
EDDM3B	7.96E-03	3.719682	-2.86875	2.256957
NCOA7	8.53E-04	5.679178	-0.45552	2.256698
CD46	2.69E-03	4.611718	-1.69987	2.256624
EP300	2.30E-03	4.749502	-1.52988	2.256513
FBXO38	1.32E-04	7.775053	1.557894	2.256292
SKI	1.67E-04	7.476892	1.301523	2.256136
SLC2A3	8.32E-04	5.703742	-0.42879	2.254439
ZNF295-AS1	2.35E-03	4.731874	-1.55147	2.254377
DNA2	4.67E-04	6.302033	0.197595	2.25433
USP12	8.29E-05	8.378313	2.050341	2.25406
CUEDC1	3.00E-03	4.517369	-1.81791	2.253803
HMGCR	3.13E-04	6.740689	0.628263	2.253673
KLHL7	1.24E-04	7.851589	1.622277	2.252745
CRCP	7.81E-05	8.45944	2.114026	2.252487
FBN2	3.32E-04	6.67484	0.56508	2.252159
LOC100505622	6.55E-04	5.945977	-0.16954	2.251929
YWHAZ	6.72E-04	5.920399	-0.19655	2.251848
LOC101928907	6.06E-04	6.02704	-0.08452	2.251606
ZSCAN12	2.39E-04	7.049725	0.918135	2.251311
OR51D1	2.24E-04	7.126895	0.98885	2.25065
SPAG9	9.16E-05	8.245869	1.945112	2.250492
CAPRIN1	1.34E-04	7.748792	1.53567	2.24992
GEN1	2.15E-04	7.176679	1.034124	2.249745
CXCL11	5.62E-03	3.995391	-2.49503	2.249285
SMURF1	1.03E-04	8.091948	1.820814	2.249275

PGRMC1	4.56E-04	6.326982	0.222718	2.249246
PACRGL	8.05E-03	3.711078	-2.88058	2.249065
STK32B	6.63E-03	3.862871	-2.67332	2.24898
HIF1A-AS2///HIF1	5.76E-04	6.078443	-0.03105	2.248941
SLC6A6	2.05E-03	4.850644	-1.4069	2.248479
SPAG9	8.14E-03	3.702274	-2.8927	2.248419
OTUD6B-AS1	3.34E-05	9.683507	3.008929	2.247656
DYRK1A	1.63E-05	10.82765	3.746771	2.247501
DSTYK	3.76E-04	6.535714	0.429904	2.247412
ALDH1L1	1.14E-03	5.398512	-0.76685	2.24702
C10orf76	2.56E-04	6.972736	0.846929	2.246383
NOX1	3.13E-04	6.741208	0.628758	2.246153
RCOR1	9.12E-05	8.250952	1.949179	2.245785
MARCH7	5.53E-04	6.122123	0.014115	2.245498
NETO2	4.51E-05	9.231165	2.692001	2.245246
CCNA2	1.91E-04	7.315312	1.158789	2.244904
DCAF4	2.84E-03	4.564462	-1.75883	2.244877
C1R	8.21E-03	3.695657	-2.90181	2.244377
ZNF273	9.82E-03	3.557616	-3.0933	2.244359
SMC5	2.44E-05	10.17061	3.333708	2.244237
LRRC20	1.32E-03	5.253167	-0.93242	2.244189
TMEM38B	3.52E-05	9.599689	2.95135	2.243869
MAP7D2	1.45E-03	5.1662	-1.03293	2.243684
MCM9	5.73E-04	6.084438	-0.02484	2.242966
SLC39A9	1.34E-03	5.241516	-0.94582	2.242921
LPGAT1	7.49E-05	8.515245	2.157497	2.242914
POLK	1.27E-04	7.818273	1.594321	2.242536
ZBTB33	6.36E-05	8.740934	2.330569	2.242417
CTSS	1.21E-03	5.339917	-0.83323	2.242052
ZNF44	9.28E-05	8.227717	1.930566	2.241916
CSNK2A1	2.54E-05	10.1069	3.292157	2.241722
NUBPL	1.22E-03	5.329457	-0.84514	2.241506
MECOM	7.70E-04	5.781795	-0.3444	2.241448
PPP6C	5.72E-05	8.891758	2.44384	2.24123
BAZ2B	3.18E-04	6.72449	0.612767	2.24102
FAM102A	1.89E-03	4.926386	-1.31581	2.24081
EIF2B3	1.39E-04	7.70873	1.501637	2.240635
ITSN1	5.00E-03	4.090377	-2.36881	2.240405
GCA	7.58E-04	5.797352	-0.32767	2.240397
LOC100506544	1.82E-03	4.961135	-1.27429	2.240279
PIK3CB	3.37E-04	6.65634	0.547238	2.239974
HEATR5A	1.87E-04	7.342295	1.182815	2.239271
ERCC8	2.77E-05	9.968627	3.201033	2.239177
PTGER4	1.06E-03	5.462816	-0.69455	2.238839
ASIC2	1.01E-04	8.112543	1.837572	2.238761
TTC14	1.80E-04	7.389171	1.224372	2.238631
STRN	6.83E-05	8.643334	2.256256	2.238073
RBL2	4.12E-05	9.366534	2.788459	2.237818

CCNA2	5.64E-04	6.101642	-0.00703	2.237794
CREBL2	7.07E-04	5.867676	-0.25249	2.237545
APBB1IP	8.70E-05	8.314055	1.999484	2.237353
GUSBP3	4.98E-04	6.231931	0.126587	2.237265
GNA12	6.75E-03	3.848894	-2.69227	2.236973
RNASE3	1.08E-03	5.449441	-0.70954	2.236541
CNTNAP2	2.04E-04	7.238871	1.090304	2.23648
HIF1A-AS2///HIF1	4.22E-04	6.410645	0.306401	2.236081
ROPN1L	3.40E-04	6.649526	0.540655	2.235842
SUMO1	1.94E-04	7.30043	1.145504	2.235177
ARHGAP15	1.97E-04	7.277798	1.125257	2.235095
RRM2B	5.80E-05	8.870608	2.428069	2.234807
RFX7	2.95E-03	4.531732	-1.79986	2.234346
CCNJ	8.31E-05	8.375343	2.047998	2.234159
EIF4B	9.15E-04	5.609985	-0.53126	2.23414
PHF6	2.22E-04	7.138732	0.999639	2.233624
CELF4	2.60E-04	6.952493	0.828097	2.233524
CEP350	8.85E-05	8.291802	1.981785	2.233253
ARHGAP1	1.39E-04	7.704762	1.498257	2.232585
TLR7	1.85E-03	4.942136	-1.29697	2.232304
SYK	9.00E-04	5.625952	-0.51372	2.231908
LDLR	5.84E-04	6.065284	-0.04471	2.231884
CCDC30	1.04E-04	8.081115	1.811983	2.231613
PROK2	8.31E-05	8.375808	2.048366	2.231345
ILDR1	1.48E-03	5.15062	-1.05105	2.230623
TNFAIP3	4.34E-05	9.28807	2.73272	2.230569
ZNF407	1.62E-04	7.519923	1.339078	2.230418
C8orf34	4.22E-04	6.411536	0.307287	2.230315
OTUD6B-AS1	4.23E-05	9.32754	2.760817	2.230057
SAFB2	5.47E-04	6.133594	0.025936	2.229836
PM20D2	1.67E-04	7.476826	1.301465	2.229811
GOLT1B	1.04E-04	8.074063	1.806229	2.228891
RAB43	8.52E-05	8.342406	2.021967	2.228499
PER3	1.02E-03	5.50504	-0.64739	2.228467
M6PR	6.37E-05	8.73867	2.328854	2.228417
DHFR2	4.16E-04	6.425802	0.321469	2.228361
ZNF157	6.56E-04	5.944169	-0.17145	2.227924
TLR10	2.56E-04	6.969541	0.84396	2.227621
TLR4	7.96E-04	5.748642	-0.38014	2.227539
LOC100131662	6.12E-04	6.015943	-0.09611	2.227024
ZFX	6.40E-04	5.96946	-0.14483	2.226961
GTF2I	2.35E-04	7.068235	0.935157	2.226675
CXCL1	1.38E-05	11.11843	3.921033	2.226667
SCP2	1.83E-03	4.955365	-1.28118	2.226642
SPATA3	1.13E-03	5.40272	-0.7621	2.226561
P2RY13	5.33E-03	4.038218	-2.43796	2.226403
GALR2	9.20E-05	8.239167	1.939744	2.226312
HERPUD2	1.49E-03	5.143025	-1.0599	2.226149

USP8	3.71E-04	6.551589	0.445445	2.225321
SERAC1	1.33E-03	5.252519	-0.93316	2.225296
BCL6	4.51E-04	6.33827	0.234059	2.225227
TUG1	1.77E-04	7.408587	1.241517	2.224853
ZC3H18	3.96E-04	6.478434	0.373577	2.224498
PDCD6IP	1.28E-03	5.287356	-0.8932	2.224343
GGNBP2	7.33E-05	8.545837	2.181213	2.224168
BTF3	2.37E-04	7.058671	0.926366	2.224016
TRMT13	5.35E-05	8.985386	2.513217	2.223965
TSSK3	4.68E-04	6.297849	0.193374	2.223677
TP73	2.50E-04	6.995929	0.86845	2.223645
ZNF43	9.87E-05	8.147266	1.865736	2.223634
TMPRSS11B	1.05E-04	8.065933	1.799589	2.222681
ENOX2	3.93E-05	9.434449	2.836326	2.222191
NUCKS1	9.27E-05	8.230034	1.932424	2.221481
CRYGB	7.25E-04	5.841998	-0.27986	2.22105
ZMYM6	1.14E-03	5.39484	-0.77099	2.220745
ZNF546	4.40E-05	9.268637	2.718843	2.220593
WDFY3	1.86E-03	4.941116	-1.29819	2.220575
HAL	1.08E-03	5.445017	-0.7145	2.220345
TCN1	7.86E-04	5.760321	-0.36753	2.219339
CYP51A1	7.53E-05	8.508145	2.151981	2.21924
C2orf49	1.56E-04	7.563584	1.37699	2.218564
FBXO22	6.60E-05	8.691127	2.292747	2.218546
PAK1IP1	9.70E-05	8.170047	1.884154	2.218461
TFCP2	1.27E-05	11.25737	4.002546	2.218258
GATS	1.44E-04	7.661097	1.460964	2.218185
LPAR3	5.53E-04	6.121467	0.013438	2.217655
CRBN	4.15E-04	6.429007	0.324652	2.21628
CACNB2	3.90E-05	9.446854	2.845032	2.216163
CAMK2D	1.40E-04	7.700632	1.494738	2.215614
BTBD3	1.92E-03	4.909412	-1.33615	2.21513
FAM162B	1.70E-04	7.456049	1.283264	2.214924
PAXBP1	7.18E-05	8.574324	2.203224	2.214812
C1GALT1	3.21E-05	9.741287	3.048325	2.214771
BCKDHB	2.77E-04	6.877829	0.758237	2.214692
RB1	1.08E-04	8.023984	1.765231	2.214544
DCK	3.09E-05	9.800393	3.088378	2.213977
ZNF345	5.34E-05	8.9898	2.51647	2.21383
SETD6	3.58E-05	9.575718	2.934789	2.21373
DHX33	3.21E-04	6.71338	0.602121	2.213715
MRGPRX4	7.36E-03	3.781066	-2.78462	2.213647
DYNLT3	8.92E-04	5.634887	-0.50393	2.213643
MTHFD2L	6.76E-04	5.913765	-0.20357	2.213279
KIAA0907	2.11E-05	10.40658	3.485239	2.213103
TXNRD1	7.48E-04	5.811203	-0.31281	2.213081
CCPG1	3.67E-04	6.562051	0.45567	2.213012
TXLNA	3.09E-04	6.757113	0.643942	2.212906

ADAM9	1.45E-03	5.167084	-1.03191	2.212756
ZFX	2.36E-04	7.066283	0.933363	2.212538
ITSN2	1.75E-03	4.994144	-1.23503	2.211894
HIF1A-AS2///HIF1	3.40E-04	6.647757	0.538946	2.211236
LAX1	3.25E-04	6.699471	0.588773	2.21101
THOC2	1.13E-04	7.973095	1.723327	2.21095
ASCC3	2.81E-04	6.865129	0.746291	2.210933
ZNF236	7.97E-04	5.746571	-0.38238	2.210688
ZNF791	7.26E-03	3.791308	-2.77063	2.210617
CTHRC1	1.91E-03	4.915483	-1.32887	2.210003
STAB2	1.20E-04	7.898271	1.661268	2.209577
YWHAE	3.35E-04	6.664331	0.55495	2.209499
COL4A3BP	8.65E-03	3.655237	-2.95762	2.209469
DNAJC25	1.40E-05	11.09601	3.907779	2.208991
HIF1A-AS2///HIF1	5.08E-04	6.210809	0.105072	2.208912
CLDN6	1.07E-04	8.036523	1.775518	2.208837
GPX3	5.21E-04	6.183807	0.077484	2.208566
SAMD9	1.79E-04	7.393121	1.227863	2.208468
ZMYM1	2.51E-05	10.12439	3.303588	2.208468
ATP10D	9.52E-05	8.19356	1.903114	2.208172
CRTAP	8.68E-04	5.661455	-0.47486	2.207364
TRMT1L	3.75E-05	9.505529	2.886053	2.207235
ANKRD11	4.27E-05	9.312459	2.750096	2.207182
SPN	2.82E-05	9.941515	3.183012	2.207057
ANKRD36BP2	4.02E-05	9.403234	2.814368	2.207006
ERN1	5.38E-04	6.149959	0.042771	2.206746
CDK19	9.47E-05	8.201347	1.909382	2.206542
FAM228A	1.13E-03	5.404824	-0.75972	2.205829
KIAA0040	7.15E-03	3.803837	-2.75354	2.205184
MINA	4.28E-04	6.396101	0.291915	2.205137
CD55	1.05E-03	5.470982	-0.68541	2.20502
ZNF44	5.88E-04	6.058173	-0.0521	2.204577
OR1D2	7.36E-03	3.780992	-2.78472	2.204236
G3BP1	1.05E-04	8.064201	1.798173	2.204221
YWHAQ	1.25E-03	5.308398	-0.86915	2.203926
RHOQ	1.62E-03	5.063929	-1.15254	2.2038
ZSWIM6	3.29E-04	6.68594	0.575766	2.203538
VTA1	2.72E-04	6.902291	0.781194	2.203509
LOC105379704	3.20E-04	6.715348	0.604007	2.203266
LOC643802	7.18E-03	3.80085	-2.75761	2.20292
SAMD13	1.54E-04	7.579146	1.390456	2.202584
HCCS	5.54E-05	8.935459	2.476311	2.202405
GCC2	2.29E-05	10.2709	3.398556	2.20217
SERPINE1	5.45E-03	4.019689	-2.46262	2.201934
TUBE1	1.83E-04	7.369535	1.206992	2.201842
PAPD4	3.95E-04	6.481513	0.376614	2.201592
SYK	9.64E-04	5.558688	-0.58783	2.2011
PRAC1	7.22E-04	5.846859	-0.27468	2.200979

DUOXA2	1.66E-04	7.486967	1.310333	2.200734
SPIN1	2.85E-04	6.845625	0.727909	2.200137
CD200R1	9.27E-05	8.229453	1.931959	2.199986
COL4A1	2.85E-03	4.56018	-1.76419	2.19995
CCNYL1	1.40E-04	7.69829	1.492741	2.199747
KAT6A	3.62E-04	6.578852	0.472064	2.199363
BCL11A	2.74E-04	6.893864	0.773294	2.198811
CREG1	9.92E-04	5.530961	-0.61857	2.198705
LARP7	2.93E-05	9.883945	3.144575	2.198306
LOC105378997	1.09E-04	8.020232	1.762149	2.197909
PCF11	1.47E-03	5.157187	-1.04341	2.197893
SNAPC1	1.16E-04	7.936683	1.693194	2.196763
VNN1	3.52E-04	6.609642	0.502019	2.196382
HNRNPR	2.77E-05	9.970343	3.202171	2.196371
AASDH	2.25E-05	10.30257	3.418901	2.196246
POGLUT1	4.52E-05	9.230294	2.691375	2.195981
ZNF740	3.62E-04	6.577496	0.470741	2.195792
TAF4B	6.37E-04	5.974559	-0.13947	2.19573
CLEC5A	5.83E-04	6.067317	-0.0426	2.195633
SLC38A11	8.49E-03	3.669845	-2.93743	2.195507
ZDHHC17	5.76E-04	6.079568	-0.02989	2.195416
TMEM106A	2.29E-04	7.102976	0.967001	2.195177
B3GNT5	3.56E-03	4.370765	-2.00398	2.195172
PSG2	2.57E-04	6.964372	0.839154	2.195067
MAP9	9.96E-05	8.134648	1.855515	2.194867
RABL6	2.62E-04	6.942302	0.818599	2.19481
NFYC-AS1	7.05E-04	5.871305	-0.24863	2.194777
HNRNPLL	2.26E-04	7.11512	0.978102	2.194597
C20orf197	2.05E-04	7.234198	1.086097	2.194545
LIMD1	1.22E-03	5.332402	-0.84178	2.194411
SNX19	9.34E-04	5.589336	-0.55399	2.193972
SOD2	5.04E-05	9.072682	2.577266	2.193821
OLFML1	9.84E-05	8.151166	1.868893	2.193476
LOC101929122	9.60E-04	5.562827	-0.58326	2.19245
USP44	8.33E-03	3.684184	-2.91763	2.192395
TAS2R19	6.99E-05	8.610454	2.23104	2.192122
GPR155	1.36E-04	7.733458	1.522662	2.192051
ZC3H11A	1.26E-04	7.834785	1.608191	2.191791
PYROXD1	7.97E-05	8.432547	2.092979	2.191492
P2RY1	8.96E-05	8.27439	1.967905	2.191375
FAM98A	4.70E-05	9.173813	2.650708	2.191285
AP4S1	1.44E-03	5.171821	-1.0264	2.190931
SYK	1.10E-03	5.426501	-0.73531	2.190861
RPS10P7	5.50E-04	6.127058	0.019203	2.190763
SMC4	9.13E-05	8.250045	1.948454	2.190503
CCSAP	1.36E-03	5.231643	-0.95719	2.190489
LUC7L3	1.92E-04	7.308655	1.152849	2.19039
LYRM2	2.70E-04	6.909358	0.787815	2.188771

TNKS	1.16E-04	7.93809	1.694361	2.187765
DBF4B	7.44E-05	8.524974	2.165048	2.1876
TES	1.21E-04	7.882953	1.648497	2.187431
STMN2	4.30E-03	4.213171	-2.2076	2.187009
EIF4EBP2	6.32E-05	8.750267	2.337633	2.18696
XPO1	2.35E-05	10.23156	3.373197	2.186844
TMEM230	5.29E-04	6.169205	0.062527	2.186757
TBL1XR1	6.57E-05	8.696488	2.296828	2.186583
RFPL1	7.17E-04	5.853974	-0.26709	2.18603
ZNF253	7.00E-05	8.609426	2.23025	2.185777
PRR4	1.81E-04	7.380511	1.216712	2.185745
SAMSN1	7.62E-05	8.492195	2.139574	2.185641
KCNQ1	1.58E-03	5.085708	-1.12694	2.185009
CARD8	1.81E-04	7.378836	1.215229	2.184384
RABGAP1L	4.15E-03	4.242961	-2.16882	2.184252
RPL32P3	1.96E-04	7.282377	1.129358	2.184227
TTF2	6.79E-03	3.844897	-2.69769	2.184185
KYNU	2.40E-03	4.713303	-1.57427	2.184047
HIPK2	1.43E-03	5.184351	-1.01186	2.183995
BTN2A2	6.34E-04	5.97896	-0.13485	2.183227
ST13	1.95E-04	7.290316	1.136463	2.18313
GPATCH2L	4.59E-04	6.320664	0.216363	2.183123
PTPRR	2.84E-03	4.563644	-1.75985	2.183092
DEK	6.24E-05	8.768635	2.351515	2.182902
LOX	2.41E-03	4.706396	-1.58276	2.182875
EIF4E3	3.64E-04	6.572107	0.465486	2.182763
ZCCHC7	1.08E-04	8.034027	1.773472	2.182191
CDK12	3.22E-05	9.735698	3.044525	2.181692
CDCA7	6.79E-04	5.908722	-0.20891	2.180917
RPL36AP40	1.62E-05	10.84594	3.757884	2.180692
WBP4	1.69E-04	7.464934	1.291053	2.180562
HSD17B12	1.88E-05	10.59235	3.601996	2.18052
MIER3	3.10E-04	6.75107	0.638177	2.180366
SLC4A7	2.20E-04	7.14593	1.006192	2.179955
SUMO1	2.47E-04	7.011979	0.883307	2.179937
RNASE4	2.68E-03	4.614615	-1.69627	2.179012
TRIB1	2.57E-03	4.650884	-1.65126	2.178368
VN1R1	2.12E-03	4.823225	-1.44009	2.177974
LILRB2	5.51E-04	6.126204	0.018322	2.17761
SPG20	8.20E-04	5.718377	-0.4129	2.177487
HIPK1	2.53E-03	4.665153	-1.63361	2.177302
CALCRL	4.52E-03	4.17255	-2.26068	2.177048
GLIS3	6.95E-05	8.618854	2.237491	2.177018
ZNF281	3.91E-04	6.493534	0.388464	2.176577
LIAS	1.76E-04	7.411865	1.244408	2.176255
KRTAP4-8	2.04E-04	7.237205	1.088804	2.175837
NSUN7	1.08E-03	5.450458	-0.7084	2.174892
ZBTB34	1.01E-04	8.116085	1.84045	2.174206

TULP3	1.94E-04	7.298868	1.144109	2.173567
PAK1IP1	1.80E-04	7.38617	1.221718	2.17351
GNB4	1.06E-03	5.465649	-0.69138	2.173297
ELF1	2.01E-04	7.257487	1.10704	2.173249
MS4A15	6.72E-05	8.665085	2.272888	2.173033
RERE	9.01E-05	8.266897	1.961924	2.172578
MAGOHB	2.62E-05	10.05853	3.260431	2.172439
SRI	1.85E-04	7.352675	1.192037	2.172162
RAB2A	7.70E-05	8.478035	2.128541	2.17181
HAUS6	9.90E-05	8.14274	1.862071	2.1718
EPB41L3	7.10E-04	5.863925	-0.25648	2.17146
MOB4	1.53E-05	10.94366	3.816903	2.170488
LRRC2-AS1	1.11E-04	7.989381	1.736765	2.170484
NDUFC2	1.93E-05	10.5483	3.574509	2.170301
PRRG4	4.78E-04	6.274976	0.170262	2.170096
THRAP3	1.74E-04	7.432221	1.262334	2.16988
RYK	1.97E-04	7.281271	1.128367	2.169826
CELF4	3.62E-03	4.358193	-2.02009	2.169738
FANCM	2.54E-04	6.977837	0.851668	2.169728
GLTSCR1L	1.81E-05	10.65721	3.642249	2.169525
GBP3	3.28E-04	6.688116	0.577859	2.169164
ABI2	4.81E-05	9.140365	2.626506	2.168951
SYK	1.34E-03	5.240276	-0.94725	2.16877
OXTR	1.96E-04	7.283166	1.130064	2.168344
GTF3C4	9.91E-05	8.141198	1.860823	2.168093
LOC100132790	7.72E-04	5.778503	-0.34794	2.167751
ACSM5	1.51E-04	7.605074	1.412836	2.167414
CCNA2	2.96E-04	6.805692	0.690139	2.167293
SUMO1	7.12E-05	8.585724	2.212013	2.167162
CUL4B	4.91E-05	9.110029	2.604481	2.165603
METTL15	5.88E-05	8.852043	2.414196	2.165593
GPR34	9.95E-03	3.547373	-3.10761	2.164109
LOC100129503	3.37E-03	4.41869	-1.9428	2.163823
TMEM184C	1.74E-03	5.000901	-1.22701	2.163735
FAM168A	4.84E-05	9.128794	2.618114	2.16346
MTIF2	8.40E-05	8.361406	2.036995	2.1634
COG3	2.48E-03	4.68354	-1.61091	2.16324
ANKRD33B	4.94E-05	9.100014	2.597194	2.163133
TSC22D2	1.03E-03	5.498477	-0.65471	2.162832
DSE	9.47E-05	8.200554	1.908744	2.162742
SEC62	2.79E-05	9.959168	3.194751	2.162546
RSAD2	4.71E-04	6.292297	0.18777	2.162409
RALGAPA1	7.53E-05	8.509036	2.152674	2.161798
CWC25	3.17E-05	9.761725	3.062204	2.161038
TRGV7	6.14E-04	6.012319	-0.0999	2.160425
NCOA4	2.08E-03	4.838174	-1.42198	2.160107
PPP2R2C	2.54E-04	6.979588	0.853293	2.159461
HBP1	1.62E-04	7.51497	1.334766	2.159415

NUP50	2.91E-04	6.82193	0.70552	2.159346
WDR33	7.60E-05	8.495734	2.142329	2.159257
USP27X	7.18E-04	5.851974	-0.26922	2.159177
PRDX3	1.87E-03	4.933579	-1.3072	2.15898
HIF1A-AS2///HIF1	6.83E-04	5.903914	-0.214	2.158155
AGO4	1.36E-03	5.228372	-0.96096	2.157604
ANKRD20A2	2.71E-03	4.604119	-1.70933	2.157024
MFI2-AS1	6.45E-05	8.72181	2.316072	2.156774
CYB5B	5.89E-05	8.849495	2.41229	2.156572
DMC1	1.10E-04	8.00345	1.748352	2.15639
OTUD1	2.33E-04	7.081721	0.947534	2.155918
RNF141	3.44E-05	9.636923	2.976991	2.155898
VN1R2	2.63E-03	4.630722	-1.67625	2.155592
HNRNPR	1.79E-04	7.394267	1.228876	2.154746
TRMT11	7.96E-05	8.433713	2.093893	2.154606
ZNF626	4.45E-05	9.2534	2.707941	2.154031
SLC16A6	2.65E-04	6.928632	0.80584	2.15362
PRDM13	4.37E-04	6.371589	0.267442	2.153617
MS4A5	7.53E-05	8.508843	2.152524	2.153422
CYP1A2	1.11E-03	5.423518	-0.73866	2.153414
PAK1IP1	1.85E-03	4.945596	-1.29284	2.152611
RND3	3.19E-05	9.749469	3.053885	2.152355
PLBD1	5.84E-05	8.861542	2.421298	2.152249
DTX3L	4.63E-04	6.30927	0.20489	2.151387
PLAT	6.46E-05	8.720711	2.315237	2.151147
VN1R5	3.83E-04	6.515824	0.410389	2.150987
ZNF160	8.35E-05	8.368695	2.042753	2.150844
CCL2	1.27E-04	7.818036	1.594122	2.150447
NCL	4.30E-03	4.213195	-2.20756	2.149951
PDE7B	2.38E-05	10.21314	3.361291	2.149315
PLPP4	1.16E-03	5.382795	-0.78461	2.149091
NCR2	8.40E-05	8.360216	2.036055	2.149057
BTBD7	9.40E-04	5.583698	-0.56021	2.148599
SLC8A1-AS1///SL	2.45E-03	4.694157	-1.59782	2.14818
KDM5B	3.81E-05	9.482349	2.869878	2.14817
SYK	1.45E-03	5.168552	-1.0302	2.148056
ETNK1	2.49E-05	10.13632	3.311379	2.146819
FAM200A	2.59E-05	10.07832	3.273428	2.146797
CSNK2A1	8.60E-05	8.329925	2.012078	2.146713
LOC730961	1.72E-03	5.009105	-1.21728	2.146433
SLC44A3	1.94E-04	7.298616	1.143884	2.146417
LYRM4	5.02E-05	9.078506	2.581517	2.146346
LOC55338	1.14E-03	5.393525	-0.77248	2.146308
DSP	1.40E-04	7.696554	1.491261	2.145915
ZNF566	4.76E-04	6.281225	0.176582	2.145644
SUGP2	5.15E-05	9.040598	2.553797	2.145433
TMEM87B	3.91E-04	6.492583	0.387527	2.145318
HIST1H3F	5.65E-03	3.990762	-2.50122	2.145163

SUCLA2	5.53E-05	8.93796	2.478164	2.144588
SUMO1	1.54E-04	7.582517	1.393369	2.144562
ABHD13	4.60E-05	9.204291	2.672684	2.144365
EHMT1-IT1	1.30E-04	7.793723	1.573652	2.144275
SEPT7	1.48E-05	10.98994	3.844654	2.14427
AIMP1	1.87E-05	10.60425	3.609402	2.142952
CSDE1	1.21E-04	7.879698	1.645781	2.142856
SLC30A8	2.17E-04	7.161675	1.020507	2.142849
HIF1A-AS2///HIF1	7.84E-04	5.763083	-0.36455	2.142812
B3GNT6	1.68E-03	5.030299	-1.1922	2.142718
SLC9A1	2.49E-03	4.678672	-1.61691	2.141765
CHMP2B	8.63E-04	5.667697	-0.46804	2.14115
ZBTB26	2.59E-05	10.07897	3.273854	2.140835
MTM1	1.16E-03	5.381616	-0.78594	2.14067
NHSL1	4.83E-03	4.117904	-2.33247	2.14013
TMEM169	1.01E-03	5.517283	-0.63377	2.140052
KCTD12	2.61E-03	4.637528	-1.66781	2.139608
TKTL2	5.76E-03	3.975923	-2.52106	2.13954
GPD2	1.64E-04	7.503628	1.32488	2.13938
SYK	1.61E-03	5.071516	-1.14361	2.139162
PUM1	7.58E-05	8.500399	2.145959	2.138729
SPRED2	5.64E-03	3.992388	-2.49904	2.138623
KRTAP3-1	1.42E-04	7.677944	1.475375	2.138496
JAM3	3.31E-04	6.677842	0.567971	2.1382
CLDN11	7.28E-03	3.789954	-2.77248	2.138005
RBPJ	1.79E-04	7.393169	1.227905	2.137766
ZFYVE16	5.63E-04	6.103545	-0.00507	2.137757
ASAH1	5.02E-04	6.224562	0.119087	2.137033
PAK1IP1	1.26E-04	7.83067	1.604737	2.136625
RIOK3	5.09E-04	6.209807	0.104049	2.136382
SGO1	1.04E-03	5.483099	-0.67187	2.136339
SERINC5	5.24E-03	4.051271	-2.42061	2.136243
CCL2	3.73E-04	6.545567	0.439552	2.135787
SYK	1.43E-03	5.183065	-1.01335	2.135065
IKZF5	2.79E-05	9.96158	3.196353	2.134898
LYN	4.88E-04	6.254052	0.14906	2.134678
PGK1	2.80E-03	4.577678	-1.7423	2.13461
PLS3	2.59E-03	4.64408	-1.65969	2.134337
NT5C2	1.03E-04	8.094205	1.822652	2.134311
PSMD3	8.63E-05	8.325028	2.008194	2.134006
ARL6IP1	7.43E-05	8.527778	2.167223	2.133653
GAB1	1.16E-03	5.376596	-0.79162	2.133169
SMIM14	8.01E-05	8.425258	2.087264	2.133038
SLC24A2	4.46E-03	4.183219	-2.24671	2.133023
UBL7-AS1	2.71E-04	6.904306	0.783083	2.132687
FBXL3	6.35E-05	8.743232	2.332309	2.132501
LYN	4.91E-04	6.246543	0.141439	2.132285
NLN	1.50E-03	5.134244	-1.07014	2.131764

CT55	6.93E-05	8.621946	2.239864	2.131439
UCHL5	1.45E-04	7.651844	1.453036	2.1305
NUDT10	7.06E-05	8.597583	2.221144	2.130385
MOB1A	9.54E-04	5.568688	-0.57678	2.130063
TAB3	1.01E-04	8.118894	1.842732	2.129711
TIFA	1.06E-03	5.467608	-0.68918	2.129533
UBE2B	1.28E-04	7.813468	1.59028	2.128626
ORC5	1.37E-04	7.724324	1.514903	2.128604
NAA30	1.39E-05	11.10194	3.91129	2.1286
MTNR1A	3.76E-03	4.325914	-2.06155	2.12858
OR8B8	3.80E-04	6.523745	0.418167	2.127865
RBBP4	2.72E-04	6.899964	0.779013	2.127763
UBE3B	6.39E-05	8.735199	2.326225	2.127719
SASS6	4.94E-05	9.101785	2.598483	2.127507
TBC1D12	2.98E-03	4.522589	-1.81135	2.126921
DDHD1	7.23E-05	8.564222	2.195426	2.12686
CNGB1	2.51E-03	4.672433	-1.62461	2.126755
OR1A2	8.55E-03	3.664211	-2.94521	2.126709
GCLM	3.93E-05	9.43485	2.836608	2.126335
CCDC57	5.34E-03	4.036762	-2.43989	2.126304
GINM1	4.29E-05	9.305803	2.745358	2.125744
TUFT1	4.98E-05	9.089272	2.589369	2.125741
YTHDC2	2.29E-04	7.10149	0.965642	2.125635
TMEM64	8.07E-05	8.414814	2.079066	2.12563
LPP	1.21E-03	5.340375	-0.83271	2.125412
SYK	1.63E-03	5.057869	-1.15967	2.125306
VPS53	7.25E-04	5.842078	-0.27978	2.125078
SLC30A7	5.03E-05	9.075098	2.579029	2.125016
DIEXF	2.32E-05	10.24796	3.383782	2.124788
C17orf58	5.11E-04	6.205332	0.099484	2.124686
LRRC40	1.91E-05	10.57147	3.588984	2.124515
ATP8B4	2.07E-03	4.843162	-1.41595	2.124118
ZWILCH	5.83E-04	6.06742	-0.04249	2.124062
AJUBA	5.52E-04	6.124599	0.016668	2.123769
PCGF3	6.63E-05	8.68467	2.287828	2.123382
LRRN3	7.09E-04	5.865825	-0.25446	2.122793
IFI27	5.33E-04	6.159602	0.052676	2.12279
EPHA3	9.28E-03	3.600541	-3.03349	2.122507
COQ10B	1.61E-03	5.069994	-1.1454	2.122363
OR13D1	1.67E-04	7.481644	1.30568	2.122329
ZNF486	6.75E-05	8.658375	2.267762	2.122208
FDXACB1	7.02E-05	8.605795	2.22746	2.121928
CHDH	6.80E-03	3.84275	-2.70061	2.121009
LOC149373	7.37E-03	3.779415	-2.78687	2.120658
HERC2	1.40E-03	5.19909	-0.99479	2.120087
GEMIN4	1.51E-05	10.9648	3.829594	2.120005
RBM14	4.64E-03	4.151167	-2.28872	2.119868
SLC15A2	1.58E-04	7.548052	1.363525	2.119754

CLASP2	1.38E-04	7.713059	1.505322	2.119699
PPFIA1	4.69E-04	6.295298	0.1908	2.118795
TMEM168	2.20E-04	7.14969	1.009613	2.118792
SPTBN4	8.85E-05	8.290896	1.981064	2.118758
LINC01497	4.69E-03	4.143014	-2.29943	2.118734
UBE2Q2P1	2.50E-04	6.997132	0.869564	2.117996
KRT6B	3.64E-04	6.572592	0.465959	2.117681
COL14A1	1.65E-03	5.050673	-1.16815	2.11753
LINC00593	2.96E-03	4.528979	-1.80331	2.117452
CLDN12	3.25E-03	4.448202	-1.9053	2.11704
GNB5	1.01E-03	5.510663	-0.64113	2.116999
ZNF625	8.55E-05	8.337861	2.018368	2.116904
PLEK	2.60E-04	6.950895	0.826608	2.116284
OGT	9.42E-05	8.208448	1.915093	2.116162
CAMTA1	9.32E-04	5.591762	-0.55132	2.115823
SMIM2	3.33E-04	6.671773	0.562125	2.115443
LIN7C	1.49E-04	7.615946	1.422201	2.115392
TM9SF2	7.58E-04	5.797536	-0.32748	2.114748
LOC221272	4.37E-04	6.373624	0.269477	2.11472
DTWD1	2.45E-04	7.022789	0.893297	2.114562
LYN	4.64E-04	6.308007	0.203617	2.114529
DAAM2	8.32E-04	5.704421	-0.42805	2.114235
SH3D21	1.70E-04	7.454754	1.282128	2.114015
TMEM87B	1.01E-04	8.115068	1.839623	2.113591
COX6A1	1.37E-04	7.723887	1.514532	2.1133
LRP11	6.40E-04	5.96978	-0.14449	2.113152
C1RL	6.93E-04	5.887801	-0.23109	2.113135
VEGFA	3.45E-03	4.398213	-1.9689	2.113081
MCFD2	6.23E-04	5.998401	-0.11446	2.113059
COL15A1	2.86E-03	4.55942	-1.76514	2.112657
MFSD14B	1.06E-04	8.056323	1.791733	2.112493
PLG	3.41E-03	4.408462	-1.95583	2.112383
DR1	9.27E-04	5.597214	-0.54531	2.112303
LYN	5.14E-04	6.199804	0.093839	2.112095
NNMT	2.02E-03	4.864512	-1.39016	2.111983
C4orf33	3.77E-04	6.534456	0.428671	2.111089
IL18	2.37E-03	4.723499	-1.56174	2.111058
ZMYM2	1.67E-05	10.78902	3.72324	2.110954
TMEM181	3.00E-04	6.790201	0.675438	2.11075
PRRC2C	1.06E-04	8.056803	1.792125	2.110721
PRDM12	8.31E-04	5.704735	-0.42771	2.110506
C17orf47	4.84E-04	6.263666	0.158809	2.110329
GARS	1.53E-04	7.585282	1.395758	2.110307
UHRF1BP1	5.86E-03	3.961042	-2.541	2.110147
PPP3R2	2.80E-04	6.8667	0.74777	2.110097
TRAM1	1.78E-04	7.400123	1.234048	2.109992
ACSM2B	3.04E-04	6.773913	0.65995	2.109978
HSF2	1.89E-04	7.330591	1.172402	2.109476

SLF2	1.31E-04	7.784333	1.565731	2.109445
ALPK1	6.67E-04	5.927943	-0.18857	2.109354
LOC101928504	1.06E-04	8.05279	1.788842	2.10911
OGFRL1	2.00E-04	7.25959	1.108928	2.109052
SLC30A7	1.06E-03	5.467468	-0.68934	2.10887
LYN	8.85E-04	5.642368	-0.49573	2.108824
PFN1P2	3.88E-04	6.50281	0.397595	2.108693
EVI2A	2.10E-03	4.82879	-1.43335	2.108524
DCTN6	2.04E-04	7.239252	1.090647	2.108507
SLC24A2	1.03E-03	5.48951	-0.66471	2.108381
LRRC58	2.26E-03	4.763731	-1.51249	2.108343
HDX	1.17E-03	5.369042	-0.80018	2.10833
TMEFF1	6.87E-04	5.896599	-0.22175	2.107474
RPL21	3.85E-05	9.466815	2.859016	2.107447
VTN	3.83E-03	4.310005	-2.08205	2.107068
PSMD1	1.74E-03	4.997635	-1.23088	2.106945
RWDD3	1.71E-05	10.75552	3.702754	2.106902
DUSP10	9.13E-05	8.250207	1.948584	2.106847
CD86	2.56E-04	6.972329	0.846551	2.106421
FAM177A1	5.49E-05	8.949886	2.486996	2.106129
THAP5	1.87E-04	7.343038	1.183476	2.106053
LYN	6.52E-04	5.950274	-0.16502	2.105718
SUN5	8.97E-04	5.629714	-0.5096	2.105607
DCT	2.18E-04	7.156363	1.01568	2.105578
UNC93A	2.19E-04	7.152767	1.012411	2.105289
JAZF1	4.42E-05	9.263838	2.715411	2.105279
HMGN2	1.74E-05	10.71971	3.680786	2.10484
TRIM2	5.75E-04	6.080323	-0.0291	2.104742
SZT2	2.25E-04	7.123597	0.985841	2.104455
DAPK1	3.44E-03	4.400497	-1.96599	2.103954
KNCN	3.74E-03	4.329817	-2.05653	2.103658
USP32	1.32E-03	5.254155	-0.93128	2.102541
USP13	1.98E-04	7.271392	1.119516	2.101833
QPCTL	4.36E-03	4.203012	-2.22085	2.101754
NCKAP1	7.39E-04	5.822687	-0.30051	2.101625
HOXD13	2.24E-03	4.772839	-1.50137	2.101494
RND3	6.25E-03	3.909618	-2.61014	2.10143
KANSL1L	1.47E-03	5.158504	-1.04188	2.10113
FBN2	2.46E-03	4.690475	-1.60236	2.101042
SERPINE1	2.40E-03	4.713134	-1.57447	2.100932
PTCD2	5.12E-05	9.048353	2.559477	2.100463
PHF20	5.32E-05	8.992623	2.51855	2.100157
CCR2	4.53E-03	4.170654	-2.26316	2.100133
LYN	3.85E-04	6.510921	0.405572	2.099947
ZNF701	5.90E-04	6.053801	-0.05664	2.099867
F5	4.23E-04	6.407988	0.303756	2.0992
SUMO1	3.52E-04	6.608515	0.500925	2.099168
NEK4	1.96E-04	7.287962	1.134357	2.099013

MARCH1	8.71E-04	5.658247	-0.47836	2.098996
RASGRP1	3.43E-03	4.402004	-1.96406	2.098813
UBR5-AS1	6.31E-05	8.753305	2.339931	2.098726
SPICE1	3.32E-05	9.688853	3.012585	2.098474
SOSTDC1	8.83E-05	8.294109	1.983622	2.098051
CXorf38	1.39E-04	7.705143	1.498582	2.098045
IPO7	2.44E-03	4.695989	-1.59556	2.097955
TCAF1	2.60E-04	6.951585	0.827251	2.09789
SHISA7	9.49E-05	8.198796	1.907329	2.097463
BMPR1A	2.74E-04	6.892627	0.772133	2.096635
PIGX	3.87E-04	6.505997	0.400731	2.096006
NIPBL-AS1	1.30E-04	7.789727	1.570282	2.095791
SNRPG	6.62E-05	8.686054	2.288883	2.09577
FOXA2	2.46E-05	10.16049	3.327125	2.095749
SLCO2B1	1.63E-03	5.060985	-1.156	2.095681
ADAM21	1.51E-04	7.601889	1.410091	2.094961
RIC8B	8.79E-05	8.300869	1.989002	2.094285
MIS18BP1	2.05E-04	7.229265	1.081653	2.094238
LMNB1	4.94E-03	4.099135	-2.35724	2.094192
EDC3	7.73E-04	5.778077	-0.3484	2.094175
FRAS1	3.80E-03	4.315863	-2.0745	2.094113
GLO1	1.21E-04	7.885933	1.650984	2.093734
RIF1	4.70E-05	9.173816	2.650709	2.093463
RTTN	8.83E-05	8.29449	1.983925	2.09283
YIPF6	2.05E-04	7.231294	1.083482	2.092661
SLC35F5	7.28E-04	5.838465	-0.28364	2.092446
ERO1A	1.34E-04	7.749713	1.536451	2.092406
METTL15	3.41E-05	9.650491	2.986309	2.092144
ITIH6	8.82E-05	8.295758	1.984935	2.092141
TAF9B	3.89E-04	6.499867	0.3947	2.092042
SMIM12	1.18E-03	5.358876	-0.8117	2.091503
BET1	1.24E-03	5.312191	-0.86482	2.091285
LRCH1	8.12E-04	5.728432	-0.40201	2.090638
ICE2	1.31E-03	5.262789	-0.92136	2.090194
ITGAV	1.69E-04	7.466358	1.292301	2.089995
CXorf36	1.69E-03	5.027395	-1.19563	2.089927
LYN	5.87E-04	6.059304	-0.05092	2.089762
HPGD	6.51E-04	5.95198	-0.16322	2.089549
CARD16	5.05E-04	6.217276	0.111665	2.08952
TRIM34	3.27E-04	6.691757	0.58136	2.089475
DLC1	7.22E-03	3.795632	-2.76473	2.089409
OK/SW-CL.58	6.37E-03	3.895142	-2.62967	2.089257
TWISTNB	3.51E-05	9.607526	2.956756	2.089203
TNS3	2.19E-03	4.793477	-1.47623	2.088498
ZRANB1	3.33E-04	6.672138	0.562476	2.08849
TMED5	4.81E-04	6.270164	0.16539	2.087972
GPAT3	2.67E-03	4.61769	-1.69244	2.087132
GCSH	4.01E-05	9.405264	2.815799	2.086823

ZNF225	2.16E-03	4.802878	-1.46479	2.086503
UHMK1	2.29E-04	7.099547	0.963864	2.086406
IFI16	4.86E-03	4.112764	-2.33925	2.086336
RB1CC1	2.98E-04	6.795722	0.68068	2.08623
FBXO9	5.38E-04	6.150966	0.043806	2.085338
LOC105372881	1.82E-04	7.371286	1.208543	2.085239
GPRASP2	8.84E-04	5.643469	-0.49453	2.084131
RAD51AP1	1.11E-04	7.991512	1.738521	2.083969
UBAP2	2.22E-04	7.13551	0.996703	2.083844
SUMO1	2.11E-04	7.198082	1.053505	2.08353
BAGE	7.94E-03	3.721919	-2.86567	2.08333
AP3M1	2.60E-04	6.954738	0.830188	2.08294
CREBL2	3.23E-05	9.731084	3.041386	2.082095
FAM161B	4.29E-03	4.215508	-2.20455	2.082093
WWTR1	8.60E-04	5.671521	-0.46387	2.081822
ZNF107	6.91E-04	5.891265	-0.22741	2.081261
HIF1A-AS2///HIF1	6.53E-04	5.948724	-0.16665	2.080973
TMEM123	1.72E-04	7.442157	1.271069	2.080887
SYK	1.51E-03	5.133296	-1.07125	2.080503
RMND5A	3.94E-05	9.430516	2.833564	2.079963
CALR	3.18E-04	6.721319	0.609729	2.079721
PCYOX1	7.71E-04	5.780632	-0.34565	2.079596
HACD1	8.35E-04	5.700363	-0.43246	2.079581
ADD3	3.86E-04	6.507568	0.402275	2.07866
SLC16A14	5.10E-05	9.055305	2.564565	2.078281
PCNX3	8.72E-04	5.65777	-0.47888	2.078212
DYM	2.69E-03	4.610098	-1.70189	2.078174
MRE11A	3.12E-03	4.484347	-1.85954	2.077891
RABGAP1L	4.02E-04	6.46449	0.359805	2.077835
RAB2B	1.48E-04	7.624898	1.429903	2.0777
OCRL	6.23E-04	5.998313	-0.11455	2.077605
SETD7	7.02E-04	5.875878	-0.24376	2.077177
OPN3	6.23E-04	5.99759	-0.11531	2.076912
ARHGAP15	1.21E-04	7.885595	1.650701	2.075367
WT1	5.67E-03	3.987934	-2.505	2.075296
GEN1	8.39E-05	8.362957	2.038221	2.075103
ASPH	1.05E-03	5.474116	-0.6819	2.075092
MYCT1	2.47E-03	4.685602	-1.60836	2.075079
ZNRF3	2.04E-03	4.854152	-1.40267	2.074363
CCDC113	8.08E-05	8.412889	2.077554	2.074308
MFAP3	1.30E-04	7.795191	1.57489	2.074175
CHM	2.84E-04	6.851359	0.733319	2.073802
TMEM97	1.53E-03	5.115329	-1.09224	2.073739
MYT1	5.98E-04	6.040552	-0.07043	2.07329
GOLGA2	1.62E-04	7.519379	1.338605	2.073198
GLIPR2	2.27E-03	4.759847	-1.51723	2.073066
ATP6V1A	5.45E-03	4.02024	-2.46188	2.072783
SEH1L	1.04E-04	8.075033	1.807021	2.072492

OR1L3	1.32E-04	7.771987	1.555303	2.072466
FAM174A	3.71E-04	6.551815	0.445666	2.072001
FAM208B	2.49E-03	4.678513	-1.61711	2.071984
KMT2A	3.34E-04	6.667277	0.55779	2.07188
APOL4	5.05E-03	4.082527	-2.37919	2.071836
CHD9	1.81E-04	7.383022	1.218933	2.071827
MED13	2.64E-04	6.932955	0.809877	2.071494
CCDC138	2.00E-03	4.871585	-1.38163	2.071479
TAL2	6.56E-04	5.943746	-0.1719	2.071211
SLC25A13	6.16E-03	3.922378	-2.59295	2.070954
XRCC2	7.44E-04	5.815638	-0.30806	2.070924
SRPK2	7.53E-05	8.508183	2.152011	2.070872
LINC01128	5.85E-05	8.85783	2.418524	2.070866
CCL2	1.27E-04	7.817883	1.593993	2.070536
TMX3	4.81E-04	6.269367	0.164583	2.070096
CYR61	8.84E-04	5.644141	-0.49379	2.069968
GAPT	4.98E-04	6.231933	0.12659	2.069866
OR1A1	2.47E-03	4.687089	-1.60653	2.06985
IGK///IGKV3-20///	5.60E-03	3.997784	-2.49184	2.06972
YIPF6	3.14E-04	6.738047	0.625737	2.06961
CTPS2	1.93E-04	7.304665	1.149287	2.069592
SEPT7	6.21E-05	8.775029	2.35634	2.069332
AGPS	1.39E-03	5.205073	-0.98787	2.068965
CEP57	2.27E-05	10.2882	3.409678	2.068923
C9orf40	3.95E-05	9.426833	2.830975	2.06874
XDH	4.06E-04	6.452149	0.347596	2.068365
PIGN	1.13E-03	5.402404	-0.76245	2.068318
RSL24D1	5.26E-04	6.174354	0.067804	2.067899
PAK1IP1	2.92E-04	6.818425	0.702203	2.06789
HEPH	6.48E-03	3.88169	-2.64785	2.067729
C4orf32	3.43E-04	6.636978	0.52852	2.067535
GCLC	5.28E-04	6.170431	0.063784	2.067533
FGD5-AS1	5.71E-05	8.894243	2.445691	2.067456
UBQLN1	4.62E-05	9.198025	2.668172	2.067165
LY6K	6.92E-05	8.625581	2.242653	2.066932
NUDCD2	1.73E-04	7.433532	1.263488	2.066466
CEACAM6	7.73E-04	5.777684	-0.34882	2.066455
FAM13B	5.29E-05	9.00231	2.525682	2.066175
ERI2	1.01E-03	5.510715	-0.64107	2.066018
ADAR	9.57E-04	5.566133	-0.5796	2.065972
GUSBP3	3.50E-04	6.616378	0.508558	2.065741
CDKAL1	6.33E-04	5.981435	-0.13225	2.065711
CEP83-AS1	5.26E-03	4.049352	-2.42316	2.065591
TMEM9B	2.35E-04	7.071531	0.938183	2.065375
KIAA1211	3.94E-04	6.484569	0.379629	2.065358
ADK	6.90E-04	5.893345	-0.22521	2.065296
MFSD8	1.60E-04	7.531618	1.349252	2.06526
ID2	4.76E-04	6.280591	0.175941	2.065176

CAV1	3.58E-03	4.366409	-2.00956	2.06445
C5orf22	4.30E-04	6.3897	0.285532	2.064049
CSF2	7.81E-05	8.458756	2.113492	2.063983
TRDMT1	4.41E-04	6.363018	0.258868	2.063789
SEZ6	6.73E-03	3.85115	-2.68921	2.063743
SYNE3	5.52E-05	8.941835	2.481035	2.063102
ZHX1	1.52E-04	7.596503	1.405446	2.062744
ZFAS1	8.57E-05	8.334528	2.015727	2.062607
SUZ12	3.30E-03	4.436967	-1.91956	2.062591
ARIH1	3.35E-04	6.664827	0.555428	2.062529
CCDC88A	3.58E-03	4.366129	-2.00992	2.062378
PPP2CB	1.01E-03	5.510498	-0.64131	2.062125
NCEH1	6.48E-04	5.956576	-0.15838	2.061913
GOLT1A	1.27E-03	5.289794	-0.89041	2.061719
LAMTOR3	3.00E-04	6.789041	0.674336	2.061693
SESTD1	1.14E-04	7.959959	1.712471	2.060646
DBF4	6.44E-04	5.962752	-0.15188	2.060538
RNF183	2.87E-03	4.555955	-1.76947	2.060472
TNRC18	1.92E-04	7.309738	1.153816	2.06047
KBTBD7	4.16E-04	6.425308	0.320979	2.060466
HLF	7.63E-04	5.790537	-0.335	2.060439
DAZAP1	4.95E-05	9.096441	2.594592	2.060366
FUBP1	2.25E-05	10.3004	3.417509	2.060335
TM9SF3	7.54E-05	8.507412	2.151412	2.059747
RIMS1	3.17E-04	6.726304	0.614504	2.058889
LYN	8.00E-04	5.742636	-0.38663	2.058751
HNRNPA3	3.01E-05	9.840886	3.115675	2.058711
SERINC1	9.22E-05	8.235907	1.937133	2.058475
ZMYM5	1.08E-04	8.034313	1.773706	2.058472
IRF8	2.65E-04	6.930418	0.807508	2.058459
RNF6	9.80E-03	3.559259	-3.09101	2.058294
CTAGE6	2.47E-04	7.013971	0.885149	2.05822
API5	3.92E-04	6.491269	0.386233	2.057943
OR2J2	2.49E-03	4.677822	-1.61796	2.057606
PGLYRP4	6.73E-04	5.918703	-0.19834	2.057011
OTUD1	7.77E-05	8.46588	2.119057	2.056983
CUL3	4.59E-04	6.319007	0.214695	2.056945
AVIL	4.47E-04	6.347067	0.242886	2.056401
GNG10	3.13E-03	4.481674	-1.86292	2.05636
KCNQ2	3.46E-04	6.62763	0.519468	2.056043
DCLRE1B	5.68E-03	3.986473	-2.50695	2.055757
EIF4A2	2.01E-04	7.254122	1.104018	2.055432
LOC101926940	3.68E-04	6.560878	0.454524	2.055293
FAM21C	2.46E-04	7.015247	0.886328	2.055066
NRP2	4.34E-03	4.206694	-2.21604	2.054907
ACAD11	4.16E-04	6.425389	0.321059	2.054766
CCNA2	1.48E-03	5.151427	-1.05012	2.054645
PUS10	1.21E-03	5.339929	-0.83322	2.054168

LSG1	3.60E-03	4.361823	-2.01544	2.053265
NPTN	1.25E-03	5.311544	-0.86555	2.053121
REPS2	3.40E-03	4.411405	-1.95208	2.052963
BCLAF1	2.16E-05	10.36432	3.45837	2.052895
HOXB6	6.01E-03	3.940904	-2.56803	2.052603
HPRT1	6.07E-05	8.808019	2.381184	2.052414
SIKE1	9.77E-05	8.15979	1.875868	2.051745
REST	1.38E-03	5.217581	-0.97342	2.051499
RND3	6.88E-03	3.834095	-2.71236	2.051468
NHLRC2	3.22E-05	9.737222	3.045562	2.051364
ESR1	1.68E-03	5.032664	-1.18941	2.051204
ZNF799	8.34E-05	8.370675	2.044316	2.050701
DPYSL3	2.32E-04	7.084769	0.950328	2.050679
RPRD1A	8.38E-05	8.363344	2.038527	2.050421
CREB5	1.49E-03	5.140531	-1.06281	2.050238
PPARA	1.47E-03	5.157174	-1.04343	2.04992
FAM114A2	2.48E-05	10.14273	3.315556	2.049787
THRAP3	1.60E-03	5.0759	-1.13846	2.049713
AGAP3	5.61E-04	6.10705	-0.00144	2.049209
PHF21A	7.27E-04	5.839785	-0.28223	2.049133
WDR82	1.48E-03	5.146153	-1.05626	2.048753
CTAGE5	1.10E-04	8.002547	1.747609	2.048529
C5AR1	1.15E-04	7.947875	1.70247	2.047785
TRMT61B	6.13E-04	6.01378	-0.09837	2.047161
CCNA2	4.23E-05	9.327379	2.760703	2.046966
RND3	1.46E-03	5.165033	-1.03429	2.046861
LAMP2	7.63E-05	8.490338	2.138128	2.046632
CAMK1G	4.32E-04	6.384888	0.280729	2.046584
RIPPLY3	8.64E-05	8.322676	2.006328	2.046563
MSANTD4	1.05E-04	8.064126	1.798112	2.046269
SELENBP1	7.02E-03	3.818361	-2.73376	2.046233
LOC105379252///I	6.76E-04	5.913095	-0.20428	2.045951
CCND1	8.09E-03	3.706602	-2.88674	2.045449
PFKFB3	5.81E-03	3.967942	-2.53175	2.045423
SC5D	2.92E-04	6.818172	0.701963	2.045406
AZI2	1.51E-03	5.131494	-1.07335	2.045357
GNAO1	9.30E-03	3.598842	-3.03585	2.045332
NT5DC1	3.82E-05	9.479935	2.868191	2.045233
MAVS	2.33E-03	4.739536	-1.54208	2.045133
NDRG1	3.01E-04	6.785913	0.671364	2.045008
FOXRED2	1.58E-04	7.545596	1.361394	2.044605
RFX7	1.61E-04	7.521576	1.340518	2.044451
AHNAK	1.41E-03	5.192656	-1.00224	2.043765
BFAR	1.73E-04	7.434447	1.264292	2.043581
PATJ	2.05E-03	4.849604	-1.40816	2.04338
HAVCR2	2.29E-03	4.751945	-1.52689	2.043024
GINS4	3.21E-04	6.713885	0.602605	2.042808
CXorf23	2.75E-05	9.983672	3.211011	2.042763

SLC7A8	8.21E-04	5.717598	-0.41375	2.042706
DLAT	9.06E-05	8.260031	1.956439	2.042446
DDX18	4.12E-05	9.366709	2.788582	2.042409
UBE2E2	3.29E-03	4.43873	-1.91732	2.042307
RBMX	4.16E-03	4.240728	-2.17172	2.042074
CPEB4	1.44E-04	7.661858	1.461615	2.041867
RNF214	2.52E-04	6.990879	0.863769	2.041864
SYAP1	8.22E-05	8.389354	2.059042	2.041784
TP73	5.81E-04	6.070801	-0.03898	2.041652
ST3GAL1	3.19E-04	6.72025	0.608706	2.041619
SNHG6	1.58E-04	7.543742	1.359785	2.041587
OR4C1P	5.93E-04	6.04917	-0.06146	2.041018
GCH1	3.96E-04	6.479745	0.37487	2.040818
ZNF880	5.06E-04	6.215573	0.109929	2.0407
BACE2	4.17E-03	4.239173	-2.17375	2.040446
PFKFB3	8.92E-03	3.631247	-2.99085	2.039855
FRMD4B	2.58E-03	4.646787	-1.65633	2.039336
NBPF1	2.77E-03	4.584977	-1.73319	2.038709
TBL1XR1	3.18E-05	9.755319	3.057857	2.038611
SRPK2	1.04E-04	8.078811	1.810103	2.038594
TGS1	5.82E-04	6.067787	-0.04211	2.038538
PSMD10	5.76E-05	8.881832	2.436443	2.038272
TRIM7	9.05E-04	5.620941	-0.51922	2.038111
IL18	4.10E-03	4.254022	-2.15446	2.037991
GLUD1	8.85E-04	5.642492	-0.4956	2.037572
TFCP2L1	7.75E-03	3.7405	-2.84016	2.036517
TAF1	1.12E-03	5.412395	-0.75119	2.036368
CCDC88C	1.42E-04	7.680156	1.477265	2.03614
MTFR1	1.47E-03	5.155803	-1.04502	2.036068
ENO1-AS1	8.85E-04	5.642323	-0.49578	2.035795
L3MBTL4	2.93E-04	6.81596	0.699869	2.03567
PSIP1	2.98E-03	4.524036	-1.80953	2.035446
SGK1	1.27E-03	5.292104	-0.88777	2.035125
SDAD1	1.27E-04	7.817852	1.593967	2.034801
ARHGEF26	2.93E-03	4.537604	-1.79248	2.034205
CAPRIN2	5.73E-05	8.888691	2.441556	2.034063
KAT6B	1.05E-04	8.062581	1.79685	2.033589
DPP6	1.55E-03	5.108946	-1.0997	2.033532
CSHL1	1.08E-03	5.443607	-0.71608	2.033365
FKBP5	1.34E-03	5.238933	-0.94879	2.032962
SLC2A13	2.60E-04	6.952065	0.827699	2.032923
RASA1	1.08E-03	5.448042	-0.71111	2.032815
FAM200B	9.34E-04	5.589053	-0.5543	2.032598
SGIP1	1.62E-03	5.065301	-1.15092	2.032235
COX11	1.05E-04	8.060663	1.795281	2.032152
RAB14	3.32E-04	6.674805	0.565047	2.032141
DUSP5P1	5.05E-03	4.081857	-2.38008	2.031895
RGMA	7.39E-03	3.777539	-2.78944	2.031693

RCOR2	1.71E-03	5.016545	-1.20847	2.031673
HOXC10	2.41E-03	4.708316	-1.5804	2.031299
TANC1	2.01E-03	4.867884	-1.38609	2.031171
LRTOMT	1.08E-04	8.031948	1.771767	2.030058
DENND2A	4.81E-04	6.269157	0.164371	2.02989
PSME3	1.99E-03	4.876974	-1.37514	2.029699
CYP27B1	1.20E-04	7.890589	1.654866	2.029284
LZTFL1	6.53E-05	8.705088	2.303369	2.029141
IDH3A	9.13E-04	5.612099	-0.52894	2.029076
GORAB	9.88E-05	8.145186	1.864053	2.028883
BMP2K	2.73E-03	4.598132	-1.71678	2.028602
CREBZF	5.94E-05	8.838184	2.403821	2.028545
ST3GAL6	6.53E-03	3.875484	-2.65624	2.028459
UBE2O	5.84E-05	8.860939	2.420847	2.028372
OSGIN2	6.01E-04	6.035187	-0.07603	2.028173
ATP6AP2	6.45E-04	5.962434	-0.15221	2.027938
ZFAND5	9.02E-04	5.623821	-0.51606	2.027601
MOGAT1	2.92E-04	6.819709	0.703418	2.026948
MAN1A2	5.51E-05	8.94315	2.482009	2.026866
TM4SF1	8.40E-03	3.678221	-2.92586	2.026747
NCK1	2.18E-04	7.15791	1.017087	2.026274
PEG3	2.65E-03	4.625685	-1.68251	2.026055
ZNFX1	5.86E-04	6.060751	-0.04942	2.026022
DDHD1	1.94E-04	7.297151	1.142574	2.025935
PSPH	3.89E-03	4.297074	-2.09873	2.025902
CAPRIN1	3.70E-04	6.55539	0.449161	2.025354
GPR160	1.66E-03	5.042661	-1.1776	2.024893
TAAR8	1.47E-03	5.158047	-1.04241	2.024514
ZNF112	5.82E-03	3.966931	-2.53311	2.024453
ZNF549	2.34E-03	4.733907	-1.54898	2.024349
UPF3A	2.08E-04	7.215038	1.068824	2.024019
RINT1	3.19E-05	9.752215	3.05575	2.023903
ZNF697	2.69E-03	4.612305	-1.69914	2.023849
ZNF780A	5.52E-03	4.009831	-2.47576	2.023814
PRNP	9.91E-05	8.140982	1.860648	2.023271
SPTLC1	1.11E-04	7.998757	1.744489	2.022737
TMEM95	7.39E-03	3.777805	-2.78908	2.022698
TRIM66	6.38E-05	8.737741	2.328151	2.022505
RND3	1.07E-03	5.454841	-0.70348	2.022371
RAD50	1.29E-03	5.280679	-0.90085	2.022347
RBM26-AS1	6.16E-03	3.921217	-2.59451	2.022277
TSC22D1-AS1	1.22E-04	7.877077	1.643592	2.022264
RMND5A	3.62E-04	6.577084	0.47034	2.021979
KIT	9.70E-03	3.566685	-3.08064	2.02186
PLEKHA3	5.30E-04	6.165992	0.059233	2.021374
PPP1R21	2.02E-04	7.250851	1.101078	2.021219
BEND3	9.62E-05	8.180782	1.892817	2.02109
HSP90AA1	2.47E-04	7.013629	0.884833	2.020831

NR4A1	2.82E-04	6.858347	0.739905	2.0207
DNAH2	4.22E-03	4.229485	-2.18635	2.020682
ST3GAL6	7.30E-03	3.786995	-2.77652	2.020593
LRP4	2.05E-03	4.849547	-1.40823	2.02041
FOXD3	5.29E-05	9.000778	2.524554	2.020388
SH3GL2	7.72E-04	5.77844	-0.34801	2.01949
ZNF507	5.37E-03	4.031828	-2.44646	2.019344
PRNP	8.11E-05	8.40771	2.073485	2.018885
IRF2BP2	7.39E-04	5.823076	-0.3001	2.018837
CFAP221	2.43E-03	4.699824	-1.59084	2.018791
TDRG1	1.97E-04	7.2775	1.12499	2.018747
RBM48	2.66E-05	10.0338	3.244149	2.018687
CPNE3	4.69E-04	6.295589	0.191093	2.018394
ATAD5	3.67E-04	6.5629	0.456499	2.018303
FMNL2	8.34E-04	5.701469	-0.43126	2.018258
IL33	1.73E-03	5.006437	-1.22044	2.018045
HERC2P7	5.86E-04	6.061531	-0.04861	2.017885
TCTE3	9.07E-04	5.618149	-0.52229	2.017826
LOC100507022	4.64E-04	6.307217	0.202821	2.017704
CCT6A	9.75E-05	8.162386	1.877966	2.017634
PBX3	4.33E-04	6.381208	0.277055	2.01721
SLC6A19	1.25E-04	7.844888	1.616663	2.017037
MLEC	2.32E-03	4.740566	-1.54082	2.016716
ZNF273	1.24E-04	7.848288	1.619512	2.016542
LRRK2	2.59E-03	4.64445	-1.65923	2.016421
C3orf14	8.70E-04	5.660095	-0.47634	2.016004
WTAP	1.33E-04	7.763562	1.548178	2.015784
PTK2	5.25E-04	6.176833	0.070344	2.0157
CD93	7.55E-03	3.761243	-2.81173	2.01521
CD300E	2.59E-03	4.644656	-1.65897	2.014852
ZNF669	1.40E-04	7.700077	1.494265	2.014716
TTC17	8.45E-04	5.689035	-0.44479	2.014622
STX3	9.74E-04	5.548618	-0.59898	2.014453
CCBE1	5.85E-04	6.063047	-0.04703	2.014414
SOX4	2.18E-04	7.158246	1.017392	2.013642
ZNF302	2.44E-05	10.17286	3.335165	2.013472
SEPT10	9.24E-04	5.599972	-0.54227	2.013252
PTTG1IP	3.33E-03	4.429446	-1.92912	2.013059
DCLRE1C	6.09E-05	8.803236	2.377588	2.012968
GNA11	8.69E-03	3.651868	-2.96228	2.012645
METAP1	1.93E-04	7.301641	1.146586	2.012303
RNF182	8.70E-03	3.650571	-2.96408	2.01194
PPM1F	1.52E-03	5.121743	-1.08474	2.011771
MXD3	1.28E-03	5.286875	-0.89375	2.011728
ZKSCAN1	3.74E-04	6.541308	0.435383	2.011684
MAP10	1.03E-04	8.084689	1.814897	2.011386
MPP3	2.34E-03	4.733041	-1.55004	2.011338
PLGLB1	3.60E-03	4.362086	-2.0151	2.011312

PRNP	8.26E-05	8.382908	2.053964	2.011145
SRSF10	5.34E-05	8.98759	2.514842	2.011084
ZNF252P	5.81E-03	3.968999	-2.53034	2.010734
GART	2.29E-05	10.27423	3.400703	2.010326
CBWD5	7.10E-04	5.863026	-0.25744	2.010283
POGZ	6.88E-05	8.632216	2.24774	2.010178
RNF170	2.52E-04	6.988699	0.861747	2.010103
KTI12	1.46E-04	7.64342	1.445812	2.00965
FBXO28	1.05E-04	8.063774	1.797825	2.009388
ZNF614	1.72E-03	5.012914	-1.21277	2.008947
KERA	1.70E-03	5.02138	-1.20275	2.008743
GK	1.24E-04	7.852687	1.623197	2.008629
CCNA2	4.77E-04	6.278922	0.174253	2.008291
CD164	1.11E-04	7.996372	1.742525	2.008216
ST3GAL6	7.22E-03	3.796192	-2.76397	2.008203
PSTK	7.00E-04	5.878225	-0.24127	2.007924
CAMLG	8.40E-05	8.360432	2.036226	2.007864
CCND1	5.34E-03	4.037108	-2.43943	2.007859
RAB38	1.14E-03	5.393106	-0.77295	2.007513
C12orf75	4.85E-05	9.126518	2.616462	2.007216
PHC1	4.09E-04	6.443215	0.338746	2.005813
LINC00674	7.52E-04	5.804843	-0.31964	2.005644
SPTBN1	3.69E-03	4.342277	-2.04051	2.004624
IGDCC4	2.76E-03	4.589548	-1.72749	2.004575
BNIP3L	4.34E-04	6.378959	0.274808	2.004241
LINC00665	1.26E-04	7.833921	1.607465	2.004228
TMEM71	4.85E-04	6.261155	0.156264	2.004183
SLC25A46	2.21E-04	7.14373	1.004189	2.004161
TCTEX1D2	3.64E-05	9.551706	2.918157	2.003794
ZNF236	8.04E-05	8.419413	2.082677	2.003368
LINC01578	1.24E-03	5.312563	-0.86439	2.003275
SFRP2	1.60E-04	7.535596	1.35271	2.003027
EIF4E	2.92E-04	6.818442	0.702218	2.00291
PRNP	7.94E-05	8.436714	2.096244	2.002738
QPCT	5.97E-04	6.041297	-0.06966	2.002382
TMEM67	2.14E-03	4.811692	-1.45409	2.002165
ATMIN	3.47E-04	6.624082	0.516029	2.002011
INSIG2	6.50E-05	8.711403	2.308169	2.001965
EIF4B	6.23E-05	8.770593	2.352993	2.001767
ZNF277	2.95E-04	6.809519	0.693767	2.001647
MZT1	4.28E-04	6.395147	0.290964	2.001458
OSGEPL1	5.20E-04	6.187209	0.080965	2.001261
RABEP1	2.07E-04	7.221549	1.074698	2.001102
AFF2	2.56E-05	10.09619	3.285143	2.001002
TUBB1	3.12E-03	4.483311	-1.86085	2.000727
PBLD	2.25E-04	7.119547	0.982144	2.000541
MAN1A2	1.93E-04	7.30246	1.147318	2.000497
RXFP2	2.30E-03	4.748176	-1.5315	2.000241

KLHL15		3.42E-05	9.644638	2.982292	2.000028
