# Supplementary material for: Research on the Mechanism of Liuwei Dihuang Decoction for Osteoporosis Based on Systematic Biological Strategies
Source: Evid Based Complement Alternat Med. 2022 Sep 22;2022:7017610. doi: 10.1155/2022/7017610 (PMC9522519; doi:10.1155/2022/7017610)
Supplement: Supplementary Materials — Table S1-1: components meeting the screening criteria. Table S1-2: compound targets for each compound of LDD. Table S2: osteoporosis genes. Table S3: enrichment analysis of clusters based on Gene Ontology (GO) annotation of LDD-osteoporosis PPI network. Table S4: pathway enrichment analysis of LDD-osteoporosis PPI network. Table S5: Reactome pathways of LDD-osteoporosis PPI network. Table S6: Human Transcriptomics Data. Table S7: the biological processes of Human Transcriptomics Data Network. Table S8: the Reactome pathways of Human Transcriptomics Data Network. Table S9: the signaling pathways of Human Transcriptomics Data Network. Table S10: the biological processes of protein arrays data network. Table S11: the Reactome pathways of protein arrays data network. Table S12: the signaling pathways of protein arrays data network. [file 7017610.f1.zip › 7017610.f1/Table S7.pdf]

Table S7 The Biological Processes of Human Transcriptomics Data Network

Term	Biological processes	Count	%
GO:0098609	cell-cell adhesion	58	0.016763
GO:0006511	ubiquitin-dependent protein catabolic process	39	0.011271
GO:0000042	protein targeting to Golgi	10	0.00289
GO:0030033	microvillus assembly	9	0.002601
GO:0048008	platelet-derived growth factor receptor signaling pathway	11	0.003179
GO:0061028	establishment of endothelial barrier	8	0.002312
GO:0007067	mitotic nuclear division	46	0.013294
GO:0048009	insulin-like growth factor receptor signaling pathway	7	0.002023
GO:0048705	skeletal system morphogenesis	10	0.00289
GO:0016525	negative regulation of angiogenesis	15	0.004335
GO:2001275	positive regulation of glucose import in response to insulin stin	6	0.001734
GO:0000186	activation of MAPKK activity	12	0.003468
GO:0043508	negative regulation of JUN kinase activity	6	0.001734
GO:0034976	response to endoplasmic reticulum stress	16	0.004624
GO:0000086	G2/M transition of mitotic cell cycle	25	0.007225
GO:0006890	retrograde vesicle-mediated transport, Golgi to ER	17	0.004913
GO:0034454	microtubule anchoring at centrosome	4	0.001156
GO:0050856	regulation of T cell receptor signaling pathway	4	0.001156
GO:0050999	regulation of nitric-oxide synthase activity	8	0.002312
GO:0019827	stem cell population maintenance	12	0.003468
GO:0035329	hippo signaling	8	0.002312
GO:0010575	positive regulation of vascular endothelial growth factor produc	8	0.002312
GO:0001525	angiogenesis	36	0.010404
GO:0043124	negative regulation of I-kappaB kinase/NF-kappaB signaling	10	0.00289
GO:0034140	negative regulation of toll-like receptor 3 signaling pathway	3	8.67E-04
GO:0048146	positive regulation of fibroblast proliferation	12	0.003468
GO:0072593	reactive oxygen species metabolic process	9	0.002601
GO:0043123	positive regulation of I-kappaB kinase/NF-kappaB signaling	27	0.007803

PValue	Genes	Fold Enrichment	Bonferroni
3.76E-06	HSP90AB	1.86500248	0.017995
1.73E-04	USP6, USI	1.867299281	0.565513
1.73E-04	PACS1, R(	4.357031655	0.5671
2.76E-04	KLF5, MA	4.613327635	0.735942
9.83E-04	PLAT, ZF.	3.305334359	0.991351
0.001154091	EZR, F2RI	4.357031655	0.996214
0.001201818	SNX18, H.	1.616318195	0.996994
0.002995169	IGF2R, PL	4.357031655	0.999999
0.008108506	ZFAND5,	2.723144785	1
0.00933775	COL4A3, I	2.108241124	1
0.011510838	OSBPL8, (	4.021875374	1
0.013108606	LAMTOR.	2.273233907	1
0.016241766	AIDA, HII	3.734598562	1
0.021821347	CREBRF,	1.859000173	1
0.022565963	HAUS3, S	1.590157538	1
0.0227242	COPA, KI	1.806574101	1
0.023058622	KIF3A, NI	5.809375541	1
0.023058622	BCL10, CI	5.809375541	1
0.023814463	ACVR2A,	2.681250249	1
0.024012635	EIF4E, NII	2.091375195	1
0.029058455	PJA2, NPI	2.581944685	1
0.029058455	CXCL17, I	2.581944685	1
0.033608577	EMCN, C/	1.406754615	1
0.03433568	CASP8, A:	2.178515828	1
0.036434743	F2RL1, UI	8.714063311	1
0.040365446	BMI1, WA	1.936458514	1
0.041117405	P2RX7, NI	2.240759137	1
0.045064305	BCL10, M	1.461364655	1
