# Supplementary material for: Research on the Mechanism of Liuwei Dihuang Decoction for Osteoporosis Based on Systematic Biological Strategies
Source: Evid Based Complement Alternat Med. 2022 Sep 22;2022:7017610. doi: 10.1155/2022/7017610 (PMC9522519; doi:10.1155/2022/7017610)
Supplement: Supplementary Materials — Table S1-1: components meeting the screening criteria. Table S1-2: compound targets for each compound of LDD. Table S2: osteoporosis genes. Table S3: enrichment analysis of clusters based on Gene Ontology (GO) annotation of LDD-osteoporosis PPI network. Table S4: pathway enrichment analysis of LDD-osteoporosis PPI network. Table S5: Reactome pathways of LDD-osteoporosis PPI network. Table S6: Human Transcriptomics Data. Table S7: the biological processes of Human Transcriptomics Data Network. Table S8: the Reactome pathways of Human Transcriptomics Data Network. Table S9: the signaling pathways of Human Transcriptomics Data Network. Table S10: the biological processes of protein arrays data network. Table S11: the Reactome pathways of protein arrays data network. Table S12: the signaling pathways of protein arrays data network. [file 7017610.f1.zip › 7017610.f1/Table S8.pdf]

Table S8 The Reactome Pathways of Human Transcriptomics Data Network

Pathway identifier	Pathway name	Entities ratio	PValue	FDR
R-HSA-446353	Cell-extracellular matrix in	0.001340766	0.021318905	0.828656
R-HSA-1266695	Interleukin-7 signaling	0.002187566	0.024177376	0.828656
R-HSA-3899300	SUMOylation of transcript	0.003104933	0.027367079	0.828656
R-HSA-4090294	SUMOylation of intracellu	0.002822666	0.028254891	0.828656
R-HSA-2173788	Downregulation of TGF-be	0.001975866	0.029485859	0.828656
R-HSA-8875555	MET activates RAP1 and I	9.17E-04	0.029507883	0.828656
R-HSA-428359	Insulin-like Growth Factor-	9.17E-04	0.029507883	0.828656
R-HSA-1059683	Interleukin-6 signaling	0.001199633	0.03331133	0.828656
R-HSA-2470946	Cohesin Loading onto Chro	7.06E-04	0.033769101	0.828656
R-HSA-447115	Interleukin-12 family signa	0.006774398	0.038539411	0.828656
R-HSA-6811440	Retrograde transport at the	0.003881166	0.038755779	0.828656
R-HSA-8950505	Gene and protein expressio	0.005151365	0.038921538	0.828656
R-HSA-264876	Insulin processing	0.002117	0.043444065	0.828656

Genes

ITGB1;FBLIM1;PARVA;RSU1;ACTG1;LIMS1;ARHGEF6

HIST1H3A;HIST2H3A;CISH;IL7;IRS1;HGF;HIST1H3I;PIK3R1;BRWD1;HIST1H3C;JAK1;HIST1H[illegible]

DDX17;NCOA2;NPM1;CREBBP;PCGF2;BMI1;PHC3;PIAS2;HIPK2;PCM1;SUMO1;SUMO2;EP30[illegible]

NR5A1;NR4A2;AR;RXRA;SUMO1;SUMO2;RORA;NR3C1;ESR1;NR2C1;PIAS2;SF1

PPP1CB;SMAD2;PPP1CC;USP15;STAG1;SMURF2;MTMR4;UCHL5;TGFBR1;TGFBR2

RAP1B;RAP1A;HGF;GAB1;RAPGEF1;CRKL

IGF2BP2;CD44

STAT1;PTPN11;IL6ST;JAK1

NIPBL;STAG1;STAG2;PDS5B;WAPL

RALA;ARF1;SERPINB2;STAT1;TALDO1;MSN;RAP1B;MTAP;CANX;TCP1;PDCD4;IL6ST;PPIA;[illegible]

NSF;USP6NL;NAPB;NAA30;SCOC;RIC1;TMF1;STX16;GCC2;IGF2R;GOLGA4;VTI1A;NAA35;R[illegible]

RAP1B;RALA;ARF1;MTAP;SERPINB2;TCP1;PDCD4;TALDO1;MSN;PPIA

ERO1A;SLC30A7;ERO1B;SLC30A6;SLC30A5;KIF5B;MYO5A;RAB27A;EXOC6;EXOC5

AB6A;RAB9B;VAMP3
