# Supplementary material for: Research on the Mechanism of Liuwei Dihuang Decoction for Osteoporosis Based on Systematic Biological Strategies
Source: Evid Based Complement Alternat Med. 2022 Sep 22;2022:7017610. doi: 10.1155/2022/7017610 (PMC9522519; doi:10.1155/2022/7017610)
Supplement: Supplementary Materials — Table S1-1: components meeting the screening criteria. Table S1-2: compound targets for each compound of LDD. Table S2: osteoporosis genes. Table S3: enrichment analysis of clusters based on Gene Ontology (GO) annotation of LDD-osteoporosis PPI network. Table S4: pathway enrichment analysis of LDD-osteoporosis PPI network. Table S5: Reactome pathways of LDD-osteoporosis PPI network. Table S6: Human Transcriptomics Data. Table S7: the biological processes of Human Transcriptomics Data Network. Table S8: the Reactome pathways of Human Transcriptomics Data Network. Table S9: the signaling pathways of Human Transcriptomics Data Network. Table S10: the biological processes of protein arrays data network. Table S11: the Reactome pathways of protein arrays data network. Table S12: the signaling pathways of protein arrays data network. [file 7017610.f1.zip › 7017610.f1/Table S9.pdf]

Table S9 The Signaling Pathways of Human Transcriptomics Data Network

Term	Signaling pathways	Count	%	PValue	Genes	Fold Enri
hsa04141	Protein processing in endoplasmic reticul	37	0.010693	6.27E-05	HSP90AB	1.986878
hsa04350	TGF-beta signaling pathway	20	0.00578	0.001587	E2F4, LTE	2.160761
hsa04070	Phosphatidylinositol signaling system	21	0.006069	0.004381	PIK3CG, I	1.944685
hsa04722	Neurotrophin signaling pathway	24	0.006936	0.005332	PIK3CG, Y	1.81504
hsa04919	Thyroid hormone signaling pathway	23	0.006647	0.006497	PIK3CG, F	1.81504
hsa04510	Focal adhesion	34	0.009826	0.016494	CAV1, XL	1.497848
hsa04550	Signaling pathways regulating pluripoten	24	0.006936	0.031449	BMI1, PIK	1.555748
hsa04068	FoxO signaling pathway	23	0.006647	0.035054	PIK3CG, T	1.557683
hsa04915	Estrogen signaling pathway	18	0.005202	0.040933	HSP90AB	1.650036
hsa04910	Insulin signaling pathway	23	0.006647	0.046595	PIK3CG, F	1.512533
hsa04064	NF-kappa B signaling pathway	16	0.004624	0.05108	BCL10, IL	1.669002

Bonferroni
0.01747
0.360012
0.708787
0.777409
0.83983
0.990659
0.999874
0.999956
0.999992
0.999998
1
